# Protecting group free glycosylation: one-pot stereocontrolled access to 1,2-*trans* glycosides and (1→6)-linked disaccharides of 2-acetamido sugars†

**DOI:** 10.1039/d2sc00222a

**Published:** 2022-03-17

**Authors:** Xin Qiu, Anna L. Garden, Antony J. Fairbanks

**Affiliations:** School of Physical and Chemical Sciences, University of Canterbury Private Bag 4800 Christchurch 8140 New Zealand antony.fairbanks@canterbury.ac.nz; Department of Chemistry, University of Otago Dunedin 9054 New Zealand; The MacDiarmid Institute for Advanced Materials and Nanotechnology, Victoria University of Wellington Wellington 6140 New Zealand; Biomolecular Interaction Centre, University of Canterbury Private Bag 4800 Christchurch 8140 New Zealand

## Abstract

Unprotected 2-acetamido sugars may be directly converted into their oxazolines using 2-chloro-1,3-dimethylimidazolinium chloride (DMC), and a suitable base, in aqueous solution. Freeze drying and acid catalysed reaction with an alcohol as solvent produces the corresponding 1,2-*trans*-glycosides in good yield. Alternatively, dissolution in an aprotic solvent system and acidic activation in the presence of an excess of an unprotected glycoside as a glycosyl acceptor, results in the stereoselective formation of the corresponding 1,2-*trans* linked disaccharides without any protecting group manipulations. Reactions using aryl glycosides as acceptors are completely regioselective, producing only the (1→6)-linked disaccharides.

## Abstract 2

Un-protected 2-acetamido sugars are stereoselectively converted into 1,2-*trans* glycosides and (1→6)-linked disaccharides without any protecting groups. Reaction proceeds *via* intermediate oxazolines which react with acceptors under acid catalysis.

## Graphical abstract

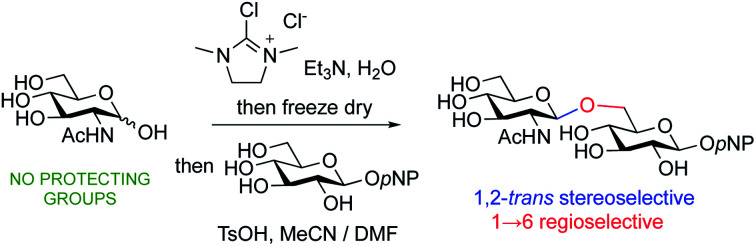

## Introduction

Nature assembles di- and oligosaccharides from unprotected monosaccharide building blocks by the use of highly selective glycosyl transferase enzymes.^1^ In many cases these require pre-activation of one component (the glycosyl donor) by first conversion to a glycosyl nucleoside di- (or mono) phosphate derivative. Using these activated substrates, glycosyl transferases, due to their evolution into highly substrate specific enzymes, are able to achieve glycosylation with complete control of both regio- and stereochemistry, and in water as the solvent.

Cloning, expression and purification of recombinant glycosyl transferases has therefore provided highly effective tools that may be used in a synthetic fashion to produce di- and oligosaccharide materials.^2^ However, the donor substrates required for these enzymes are expensive and unstable. Furthermore, only a limited number of enzymes are currently commercially available, and the effort required to clone, express and purify a new enzyme from scratch is daunting. Additionally, the exquisite specificity of these enzymes often precludes their more widespread application, since they are highly specific to the formation of one particular type of glycosidic linkage.

The majority of oligosaccharide construction is therefore still undertaken by chemical synthesis, and this is unlikely to change in the near future. However, such methods are logistically extremely demanding. Reaction control can only be achieved after multiple protecting group manipulations of both donor and acceptor components. Indeed, despite advances in glycosylation methodology,^3^ for example improvement of reaction efficiency by which the monosaccharide units are actually linked,^4^ the vast majority of the effort expended during the synthesis of an oligosaccharide synthesis involves the production of selectively protected monosaccharide building blocks to act as donors and acceptors.

In order to circumvent these considerable logistical impediments, the direct chemical glycosylation of unprotected carbohydrates has become an area of significant interest.^5^ In 2009, Shoda^6^ and co-workers first introduced the dehydrating reagent 2-chloro-1,3-dimethylimidazolinium chloride (DMC)^7^ into the carbohydrate field, and revealed its remarkable ability to selectively activate the anomeric hydroxyl group of unprotected reducing sugars in aqueous solution. A series^8^ of highly useful protecting group-free processes based on the use of DMC and analogues^9^ have since been developed, including: the synthesis of glycosyl oxazolines;^6^ the production of 1,6-anhydro sugars;^10^ the synthesis of glycosyl azides^11^ and their one-pot conjugation to other species *via* “click” chemistry;^12^ the synthesis of aryl^13^ and pyridyl thioglycosides,^14^ and the direct glycosylation of cysteine residues of peptides.^15^ DMC activation has been expanded to allow the direct synthesis of glycosyl thiols,^16^ cyanomethyl thioglycosides,^17^ glycosyl thiosulfates,^18^ glycosyl dithiocarbamates,^19^ and can even effect selective acetylation of the anomeric hydroxyl group of unprotected sugars in aqueous solution.^20^ In these examples, high levels of 1,2-*trans* stereocontrol are generally observed, due to the intermediacy of either 1,2-anhydro sugars^21^ or glycosyl oxazolines.

More recently interest has turned to intermolecular glycosylation of oxygen nucleophiles, including the one-step synthesis of *para*-nitrophenyl (PNP) and other aryl glycosides of both 2-hydroxy and 2-acetamido sugars directly from the corresponding unprotected reducing sugar.^22^ An obvious next step into the further development of DMC-activation processes is an investigation into the possibility of disaccharide formation, ideally using both completely unprotected donors and acceptors.^23^ Such protecting group free chemical glycosylation would be unprecedented,^24,25^ and would in some respects be analogous to the ability of glycosyl transferases to assemble di- and oligosaccharides; *i.e.* synthesis without the requirement for any protracted reaction sequences involving protecting group manipulations. However, significant hurdles to overcome are avoidance of both self-condensation and hydrolysis of the glycosyl donor, and control of both the regio- and stereochemistry of the newly formed glycosidic linkage. Together these represent formidable obstacles.

In this study we chose to investigate the potential glycosylation of unprotected 2-acetamido sugar donors, using glycosyl acceptors that were unprotected other than at their anomeric centres. Disaccharides of 2-acetamido sugars are components of many biologically important oligosaccharides and glycoconjugates. For example the GlcNAcβ(1→6)Gal and GalNAcβ(1→6)Gal disaccharides are component parts of proteoglycans, glycolipids, and the blood group oligosaccharides,^26^ whilst GlcNAcβ(1→6)GlcNAc is the component disaccharide of lipid A (or endotoxin),^27^ a constituent part of the lipopolysaccharides that form the outer membranes of most Gram-negative bacteria. The production of these biologically important disaccharides by chemical means^28^ was therefore the subject of extensive synthetic effort in the latter decades of the 20th century. Invariably the multi-step routes used to access them encompassed numerous protecting group manipulations in addition to the required glycosylation reaction. However, several of these syntheses were achieved using the ‘oxazoline method’^29^ – namely use of a glycosyl oxazoline as the glycosyl donor, which was activated to reaction with an acceptor by treatment with an acid. It was therefore envisaged that since unprotected 2-acetamido sugars can be converted into their corresponding pyranose oxazolines by DMC-mediated reaction,^6^ (a very useful method for producing unprotected oxazolines as donors for enzyme catalysed glycosylations),^30^ then perhaps oxazolines made in this fashion could then be used for chemical disaccharide formation, opening up a realistic possibility of protecting group free disaccharide formation *via* chemical means in the case of 2-acetamido sugars.

## Results and discussion

**Scheme 1:**
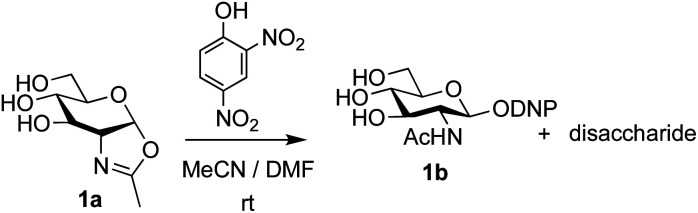
Unexpected disaccharide formation during attempted conversion of GlcNAc oxazoline 1a into its β-DNP glucoside 1b.

The possibility of disaccharide formation *via* intermediate unprotected glycosyl oxazolines made by the DMC-method was first revealed during the attempted formation of the β-di-nitrophenyl (DNP) glycoside of GlcNAc (Scheme 1). Here, using the previously developed method,^22*b*^ namely conversion of GlcNAc 2a to its oxazoline 1a by treatment with DMC/Et_3_N in water, freeze drying, dissolution of the residue in an appropriate anhydrous aprotic solvent system (*e.g.* a 10 : 1 mixture of MeCN and DMF) and addition of the phenol, unexpectedly led to disaccharide side products. It was therefore concluded that, in addition to direct reaction with the oxazoline to form the desired glycoside 1b, di-nitrophenol (p*K*_a_ in water ∼4.1)^31^ was capable of catalysing self-reaction of the GlcNAc oxazoline, leading to disaccharide formation. As previously mentioned, acid catalysis was one of the first examples of the ‘oxazoline method’ of glycosylation. There are multiple reports of this type of process in the older literature, for example using *p*-toluenesulfonic acid (TsOH) as the acid,^32^ though they were not always particularly high yielding. Though largely superseded by alternative processes, there have also been several attempts at increasing the efficiency of the oxazoline method in more recent years.^33,34^ Most relevantly, Bundle and co-workers reported the production of β-glycosides of GlcNAc *via* an intermediate furanosyl oxazoline, temporarily protected *in situ* as the 5,6-acetonide, which was reacted with an alcohol as solvent and either *p*-toluenesulfonic or camphorsulfonic acids.^35^

A study was therefore initiated in order to investigate the possible utility of this acid catalysed disaccharide formation in a more general sense. In particular the question arose as to whether this method of glycosylation could be applied using both unprotected oxazolines as the donors and unprotected glycosyl acceptors. Reaction between GlcNAc 2a as the glycosyl donor and the β-pNP-glucoside 3a, made in one step as previously described,^22*a*^ as the glycosyl acceptor was investigated as a model system (Table 1). Firstly, GlcNAc was converted to its oxazoline by treatment with DMC/Et_3_N in water, and the crude reaction mixture freeze dried. The residue was dissolved in a 10 : 1 mixture of MeCN and DMF as an anhydrous aprotic solvent system. An excess of acceptor 3a (initially 10 equiv.) was then added; an excess was deemed necessary so that glycosylation of the acceptor 3a would outcompete any self-reaction of GlcNAc oxazoline. The addition of an acid was then used to initiate glycosylation; reactions were monitored by TLC and run until all oxazoline had reacted.

**Table 1:** Reaction development with GlcNAc 2a as donor, and β-pNP glucoside 3a as acceptor

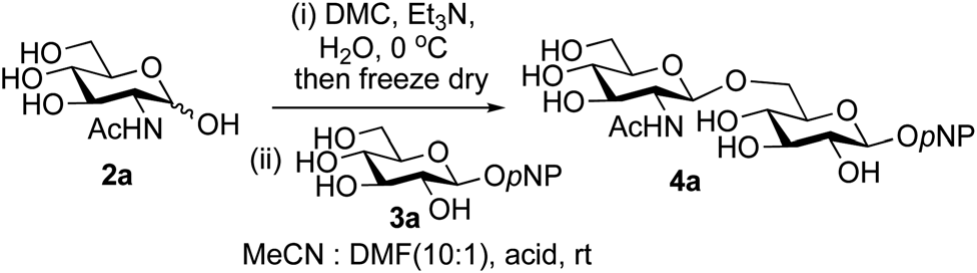				
Entry^a^	Acid (equiv.)	Time/h	Acceptor 3a/equiv.	Isolated yield of 4a (%)
1	DNPOH (6)	24	10	37
2	DNPOH (6)	24	5	39
3	DNPOH (6)	24	3	32
4	DNPOH (1)	24	5	29
5	PPTS (6)	1	5	39
6	PPTS (1)	4	5	22
7	Pyridinium triflate (6)	1	5	39
8	Pyridinium triflate (1)	4	5	18
9	TsOH (6)	3	5	56
10	TsOH (1)	3	5	50
11	CSA (1)	1	5	46
12^b^	TsOH (1)	3	5	31

a Reaction conditions: (i) DMC, Et_3_N, H_2_O, 0 °C, 1 h, then freeze-dry; 3a, MeCN/DMF (10 : 1), 4 Å powdered molecular sieves, acid, rt.

b Two-step process in which the oxazoline was specifically purified before glycosylation.

Firstly, the ability of di-nitrophenol (DNPOH) to cause oxazoline opening and glycosylation was investigated. The use of a large excess of DNPOH (6 equiv.) and 10 equiv. of the acceptor 3a led to the production of the GlcNAcβ(1→6)Glc disaccharide 4a as the only reaction product, in 37% yield (Table 1, entry 1) after 24 h. Notably glycosylation was completely regioselective for the primary 6-hydroxyl group of the acceptor.^36^ The product regiochemistry was confirmed by HMBC correlations between C-1b and H-6a/6a′ and H-1b and C-6a/6a′ (see ESI†). Although this 37% yield perhaps seems somewhat modest at first, this single step synthesis should be compared with other syntheses of protected versions of the GlcNAcβ(1→6)Glc disaccharide, which typically require at least seven synthetic steps.^28^

Reduction of the amount of acceptor 3a used to 5 equivalents actually resulted in a slight increase (39%) in product yield (Table 1, entry 2); again, reaction required 24 h to reach completion. However, the use of only 3 equivalents of 3a resulted in decreased isolated product yield (32%, Table 1, entry 3), so 5 equivalents of acceptor were used in subsequent reactions. A reduction in the amount of DNPOH used to only 1 equivalent correspondingly led to a significant reduction in yield (Table 1, entry 4, 29%).

Next alternative acids were investigated. Firstly, the use of 6 equivalents of pyridinium *p*-toluenesulfonate (PPTS) led to a faster reaction (complete after 1 h at rt) that was equally effective to DNPOH (39% yield, Table 1 entry 5), but the use of only 1 equiv. of PPTS resulted in a significantly decreased yield of 4a (22% yield, Table 1 entry 6) and required 4 h to reach completion. Pyridinium triflate showed a similar profile of effectiveness (Table 1 entries 7 & 8). However, the use of *p*-toluenesulfonic acid (TsOH) was found to produce superior results: 6 equivalents of TsOH gave 4a in 56% yield after 3 h at rt, whilst the use of only 1 equivalent of TsOH still gave 4a in a comparable 50% yield. The use of 1 equivalent of camphorsulfonic acid (CSA) also produced 4a in similar yield after 1 h at rt (Table 1, entry 11, 46%). However, given the lower cost of TsOH, the use of one equivalent of this acid was selected for all other applications. Finally, for comparative purposes a two-step process, in which the oxazoline was specifically purified before reaction with acceptor 3a, was investigated (Table 1, entry 12). Notably the yield of product obtained in this case, (31%) was considerably lower than the equivalent one-step process (50%, Table 1, entry 10), indicating that oxazoline purification is not advantageous.

Application of the process to the one-pot stereoselective synthesis of β-alkyl glycosides of GlcNAc using some simple alcohols was investigated next (Table 2).^37^ Initial studies using only 5 equivalents of *n*-pentenol as the acceptor and performing the reaction in an MeCN/DMF mixture produced the desired β-pentenyl glycoside 5a in 48% yield (Table 2, entry 1). However, a process emulating Fischer glycosylation, in which the alcohol was itself used as the solvent, was found to be more efficient. The optimised procedure involved freeze-drying of the crude reaction mixture following DMC-mediated oxazoline formation in water. Then the residue was dissolved in the appropriate alcohol and stirred with 4 Å powdered molecular sieves for 30 min at room temperature, before oxazoline opening was initiated by the addition of 1 equivalent of TsOH (based on GlcNAc starting material). After stirring for 3 h at rt, the reaction was complete (as monitored by TLC), and the corresponding β-alkyl glycosides 5a–c were isolated by column chromatography in good yield (73–76%, Table 2, entries 1–3). The process was also applicable to a tertiary alcohol acceptor, *tert*-butanol (Table 2, entry 4), although the yield of the product β-*tert*-butyl glycoside 5d was decreased (45%). This yield is comparable to that reported by Bundle (51%, *β* : *α*, 4 : 1),^35^ but in contrast our reaction was completely stereoselective. Despite this slightly lower yield, ready protection of the anomeric centre of GlcNAc as an acid cleavable *tert*-butyl glycoside may find useful application in synthesis. Furthermore, the procedure was also applicable to other unprotected 2-acetamido sugars; thus the benzyl glycosides of GalNAc 2b and ManNAc 2c were produced stereoselectively as the β- and α-glycosides 5e and 5f respectively (Table 2, entries 4 and 5). Interestingly the slightly lower yields mirror the generally lower yields observed for the DMC-mediated formation of the oxazolines of these monosaccharides.^6^ Attention then turned to the use of unprotected glycoside acceptors in order to delineate the scope of the reaction (Table 3). Firstly, using GlcNAc 2a as the glycosyl donor, a range of different unprotected glycosides (3a–i, 5d) were trialled as acceptors. The previously optimised reaction using the pNP-glucoside acceptor 3a gave the GlcNAcβ(1→6)Glc disaccharide 4a in 49% yield on a larger scale (Table 3, entry 1). Next, the use of the *manno* configured pNP-acceptor 3b gave the corresponding known GlcNAcβ(1→6)Man disaccharide 4b^38^ in a similar yield (46%), again in a reaction that was completely regioselective. This one-step synthesis compares very favourably with the previous reported^38^ convergent synthesis of 4b which required a total 6 steps (24% overall yield based on the linear sequence using the acceptor; see ESI Scheme S1†). However, surprisingly, glycosylation of the methyl mannopyranoside 3c under identical conditions was not completely regioselective, and gave a mixture of 3 regioisomeric disaccharides, in which the β(1→6) isomer 4c ^38^ predominated (50% combined yield, entry 3) and from which 4c ^38^ could be readily purified by chromatography and isolated in pure form (25% isolated yield).

**Table 2:** One-pot production of alkyl glycosides^a^

Entry	Donor	Alcohol	Product	Isolated yield (%)
1	GlcNAc 2a	*n*-Pentenol	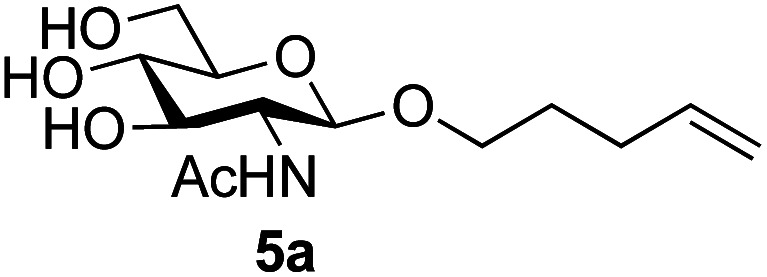	73, 48^b^
2	GlcNAc 2a	Benzyl alcohol	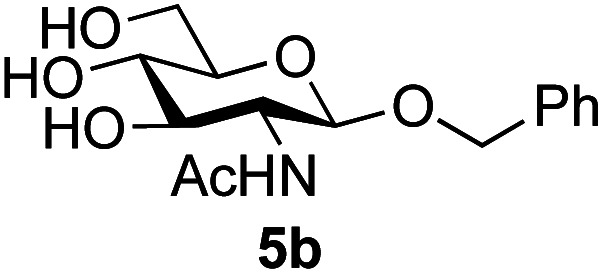	75
3	GlcNAc 2a	Isopropanol	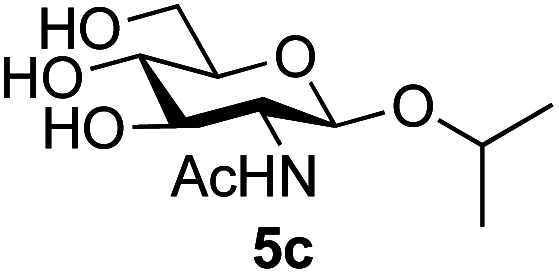	76
4	GlcNAc 2a	*tert*-Butanol	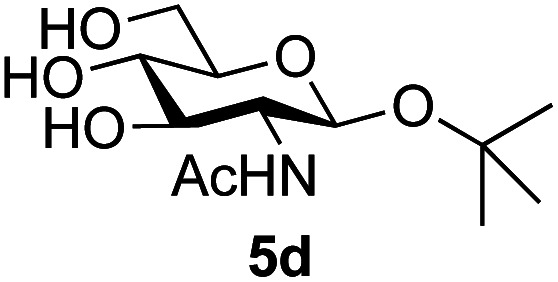	45
5	GalNAc 2b	Benzyl alcohol	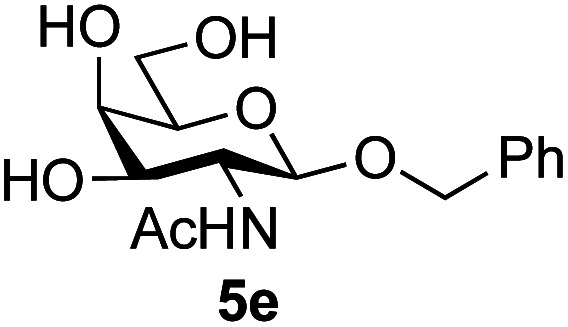	43
6	ManNAc 2c	Benzyl alcohol	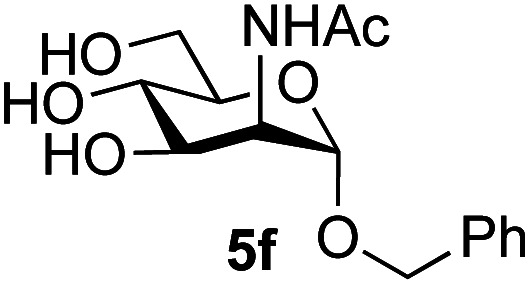	57

a Reaction conditions: (i) DMC, Et_3_N, H_2_O, 0 °C, 1 h, then freeze-dry; (ii) alcohol as solvent, TsOH (1 equiv.), 4 Å powdered molecular sieves, rt, 3 h.

b Reaction performed in MeCN/DMF in the presence of 5 equiv. of alcohol as acceptor.

**Table 3:** Disaccharide formation using unprotected 2-acetamido sugars as donors, and unprotected glycosides as acceptors^a^

Entry	Donor	Acceptor	Product	Isolated yield of (1→6) linked disaccharide (%)	Regioselectivity/total combined yield
1	GlcNAc 2a	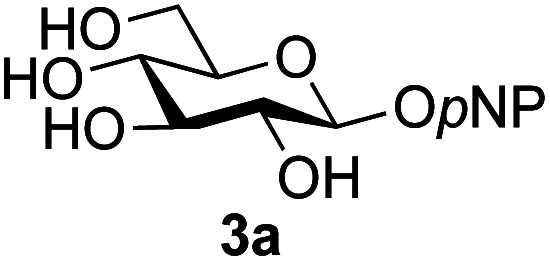	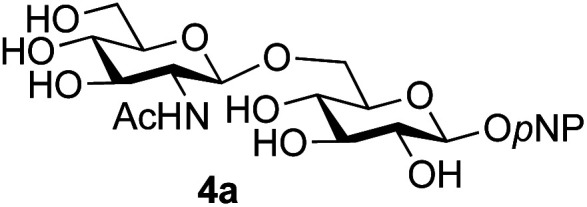	49	(1→6) only
2	GlcNAc 2a	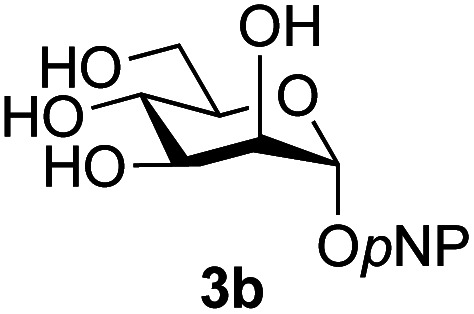	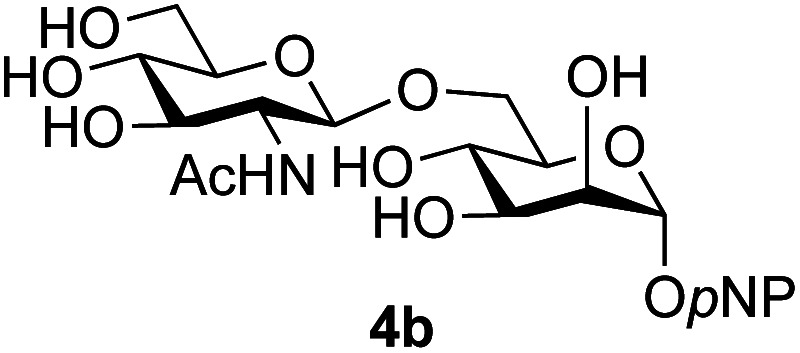	46	(1→6) only
3	GlcNAc 2a	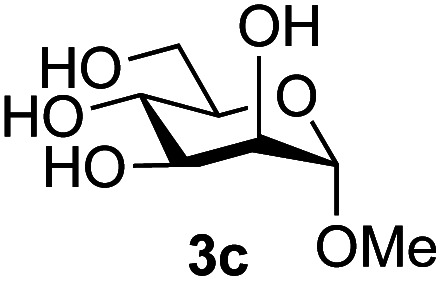	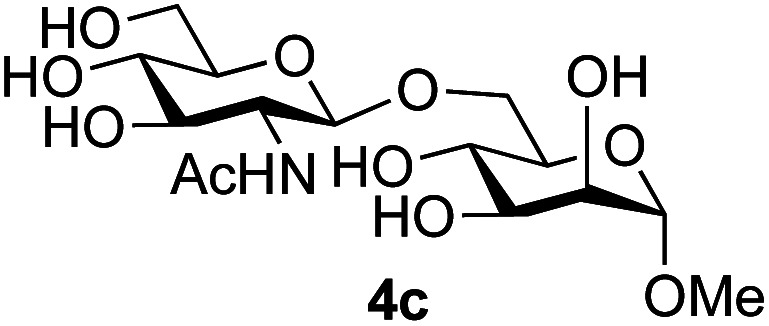	25	3 regioisomers/50%
4	GlcNAc 2a	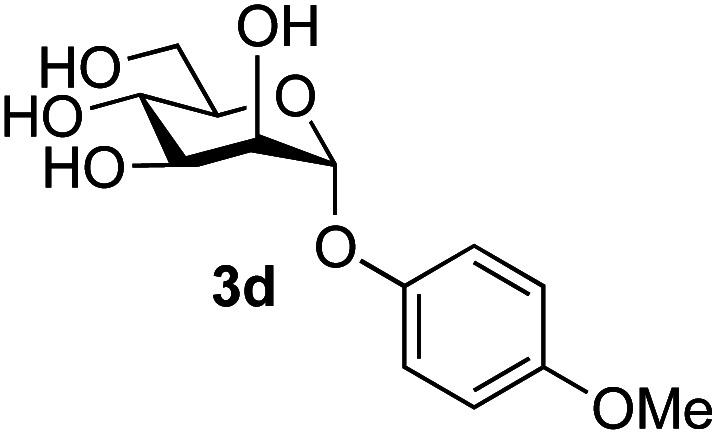	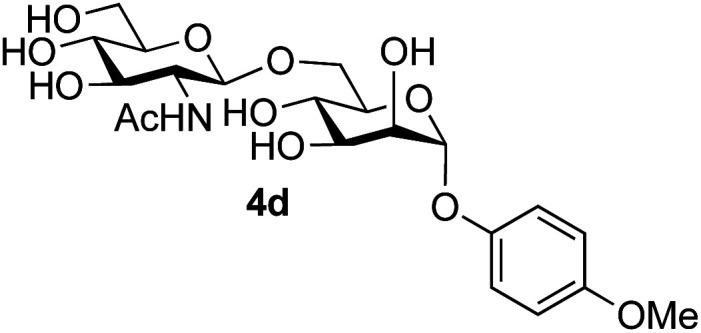	40^e^	(1→6) only^e^
5	GlcNAc 2a	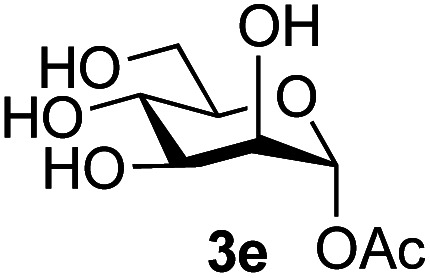	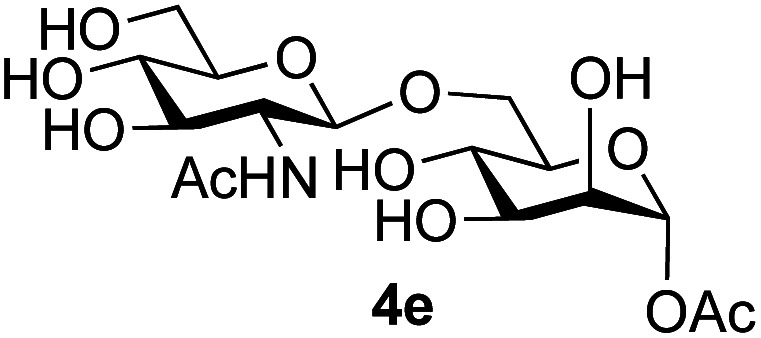	36	3 regioisomers/48%
6	GlcNAc 2a	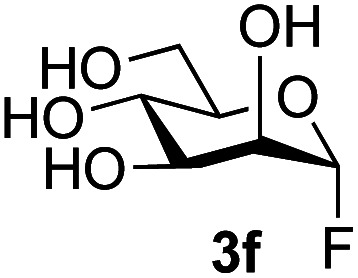	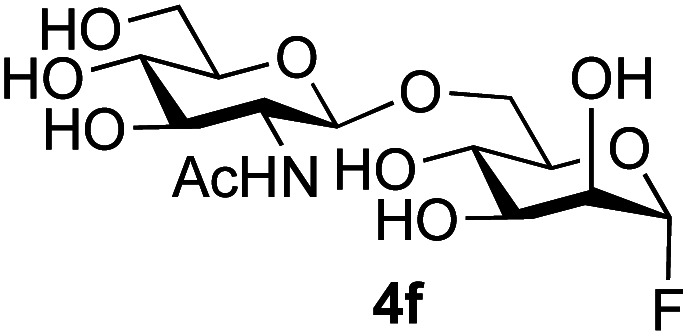	33	3 regioisomers/55%
7	GlcNAc 2a	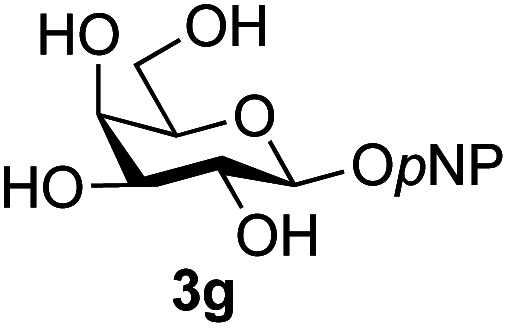	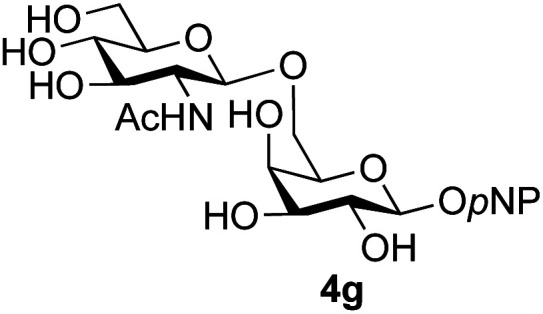	32^a^	2 regioisomers/46%^a^
				44^b^	(1→6) only^b^
8	GlcNAc 2a	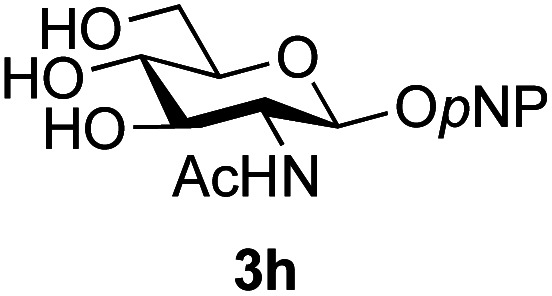	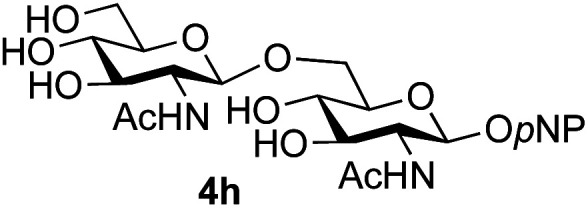	39^e^	(1→6) only^e^
9	GlcNAc 2a	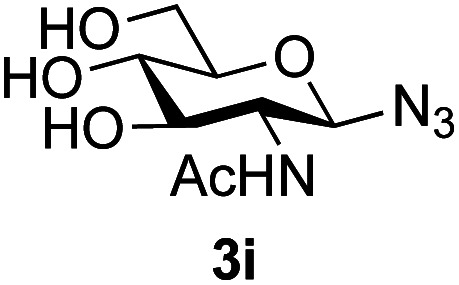	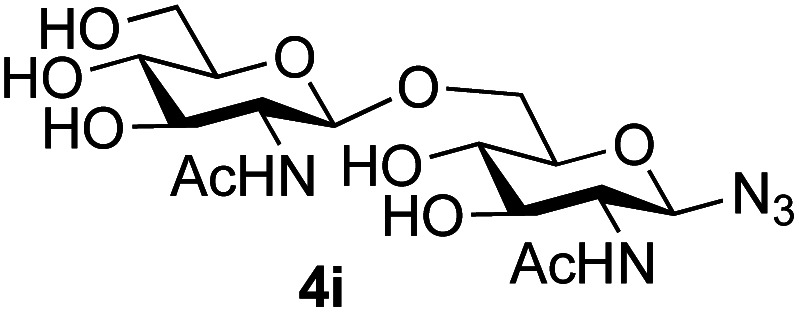	31^a^	3 regioisomers/62%^a^
				37^d^	3 regioisomers/54%^d^
10	GlcNAc 2a	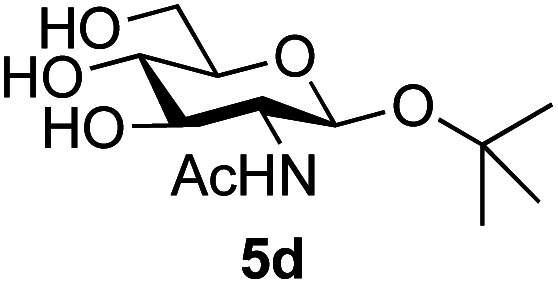	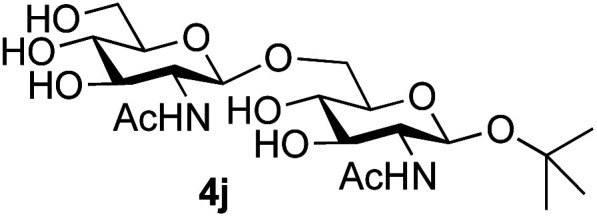	39^a^	3 regioisomers/68%^a^
				26^b^	3 regioisomers/52%^b^
11	GlcNAc 2a	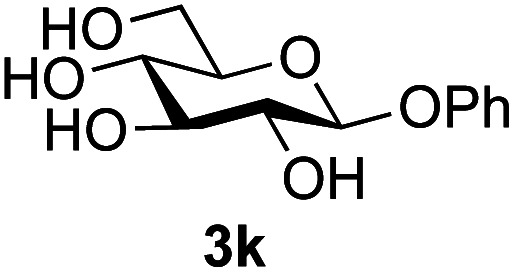	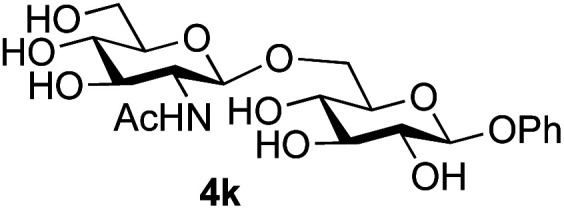	45	(1→6) < 1% of any other regioisomer
12	GlcNAc 2a	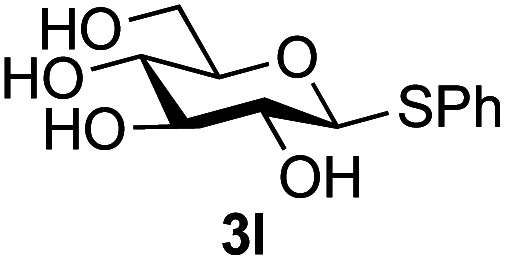	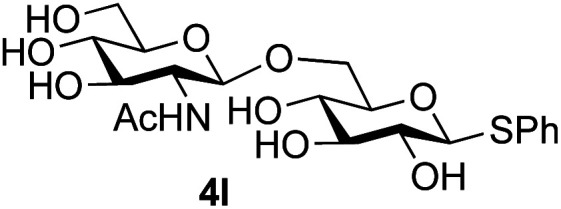	34^a^	3 regioisomers/47%^a^
				39^b^	3 regioisomer/58%^b^
13	GlcNAc 2a	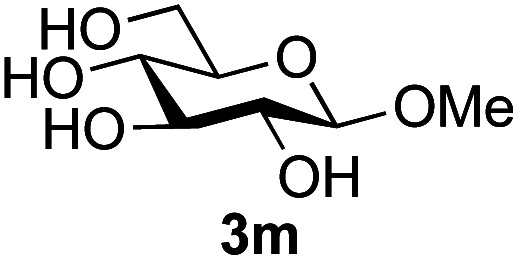	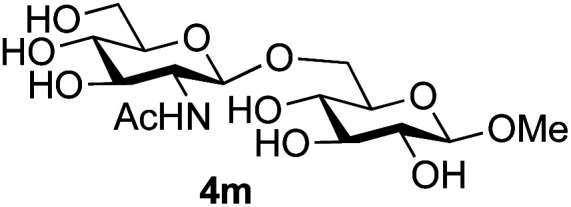	18	3 regioisomers/51%
14	GalNAc 2b	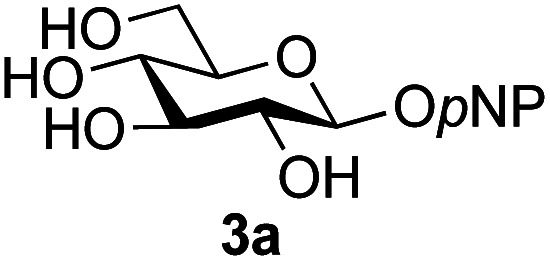	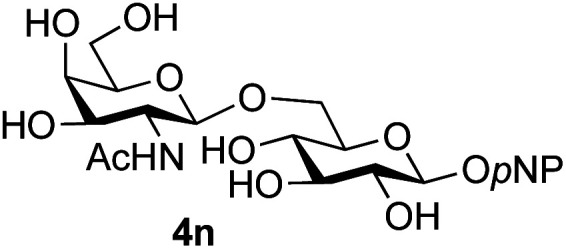	40	(1→6) only
15	GalNAc 2b	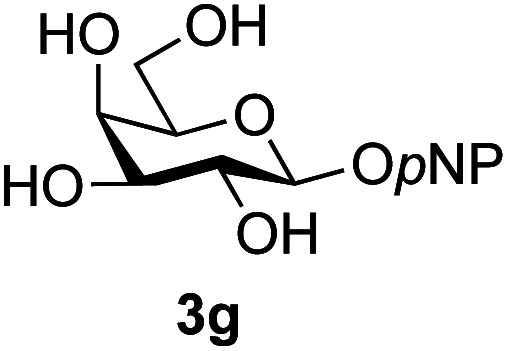	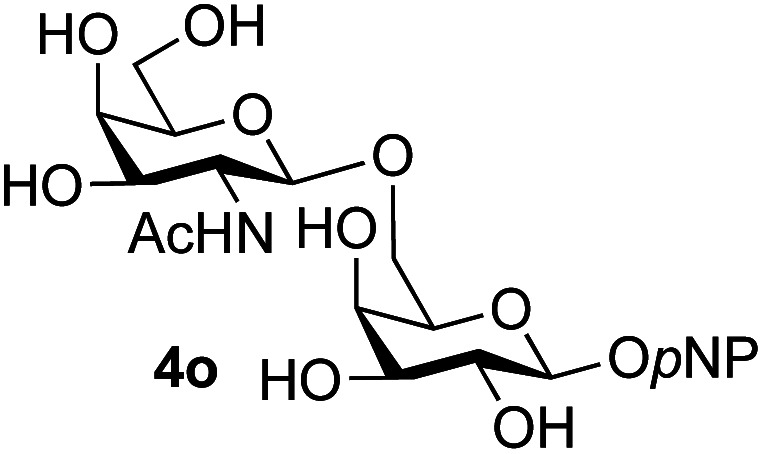	26^a^	2 regioisomers/40%^a^
				30^c^	(1→6) only^c^
16	ManNAc 2c	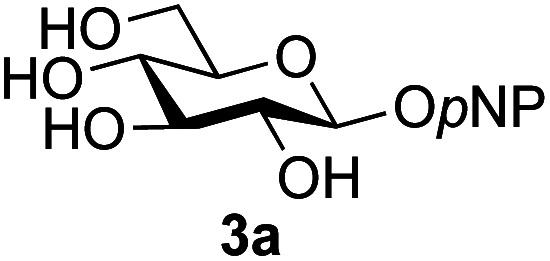	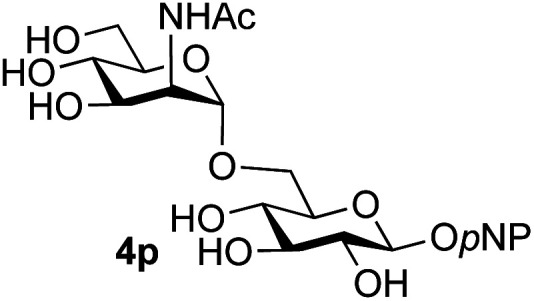	15	(1→6)/trace amount of another regioisomer

a Reaction conditions: (i) DMC 1, Et_3_N, H_2_O, 0 °C, 1 h, then freeze-dry; acceptor (3a–i or 5d), MeCN/DMF (10 : 1), 4 Å powdered molecular sieves, TsOH, rt, 3 h.

b Reaction performed at 0 °C.

c Reaction performed at −10 °C.

d Reaction performed at −16 °C for 24 h.

e Reaction performed in DMF as solvent.

The reason for the observed reduction in regioselectivity was probed further. Evidence of the importance of the use of an aryl glycoside as the acceptor was the observation that glycosylation of the *para*-methoxyphenyl mannoside 3d was also completely regioselective (40% yield, entry 4; reaction performed in DMF as the sole solvent for reasons of acceptor solubility). In contrast, reaction of the unprotected glycosyl acetate 3e (entry 5) and fluoride 3f (entry 6) were non-selective, and produced a mixture of 3 regioisomers (48% and 55% combined yields respectively) in which the (1→6)-linked disaccharides 4e and 4f predominated (36% and 33% isolated yields respectively). Use of the pNP-galactoside acceptor 3g (Table 3, entry 7) at rt was also not completely regioselective (46% total yield), but again the (1→6) disaccharide 4g ^39^ predominated (32% isolated yield). However, in this case, lowering the reaction temperature to 0 °C resulted in a completely regioselective reaction, and formation of the GlcNAcβ(1→6)Gal disaccharide 4g as the sole glycosylation product in 44% yield. Again, this one-step synthesis compares very favourably with previous reported^39^ 8-step convergent synthesis of 4g (13% yield based on the linear sequence using the acceptor; see ESI, Scheme S2†). Next the use of the pNP-glycoside of GlcNAc 3h as acceptor (Table 3, entry 8) gave the GlcNAcβ(1→6)GlcNAc disaccharide 4h ^40^ as a single regioisomer in 39% yield. This particular reaction is notable as the product is the constituent disaccharide of lipid A. This one-step synthesis therefore compares extremely favourably with previous methods^40^ of production of this disaccharide (6 or 8 steps, ∼15% yield based on the linear sequence using the acceptor; see ESI Scheme S3†). Further evidence that the complete (1→6)-regioselectivity was acceptor dependent was that the glycosylation of the GlcNAc glycosyl azide 3i to give 4i^41^ was also not completely regioselective (entry 9), even when using the lowest reaction temperature available (−16 °C; 24 h required to reach completion). Reasoning that the origin of the high (1→6)-regioselectivity was perhaps due to the steric bulk of the anomeric substituent of the acceptor, the use of the *tert*-butyl glycoside 5d was investigated. Surprisingly reaction of 5d did not result in completely regioselective reaction (entry 10), though again the (1→6)-disaccharide product 4j could be readily obtained in pure form by chromatography.

Further insight was sought into the origin of the reaction regioselectivity. To this point reactions of all the aryl glycoside acceptors had proven completely regioselective, albeit in the case of galactoside 3b requiring reaction at 0 °C, whereas glycosylation of all other glycosides had not been completely selective. Interestingly, reactions of aryl glycosides comprising both electron withdrawing (*e.g.*3b) and electron donating (*e.g.*3d) aromatic substituents had proven equally regioselective. The origin of regioselectivity of glycosylation of an unprotected, or partially protected, acceptor is complex, as it may depend on both acceptor conformation, and electronic effects.^42^ Although glycosyl acceptor reactivity has recently become an area of interest, it has undoubtedly been under-examined in comparison with the extensive studies into glycosyl donor reactivity and selectivity. For comparative and modelling (*vide infra*) purposes the outcomes of reactions of GlcNAc 2b with the β-*gluco* configured phenyl 3k, thiophenyl 3l and methyl 3m glycosides were examined. Reaction of phenyl glycoside 3k was again completely regioselective (entry 11), but in contrast reaction of thiophenyl glycoside 3l was not (entry 12); a mixture of three regioisomers was formed, even at 0 °C. Similarly, reaction of the methyl glycoside 3m gave a mixture of three regioisomers (entry 13).

**Fig. 1:**
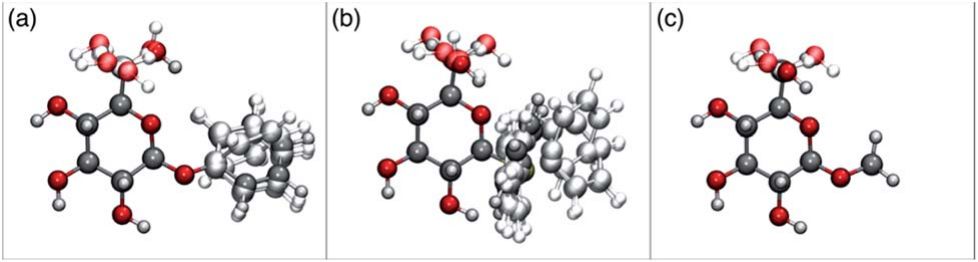
Composite structures of low energy conformations of glycosyl acceptors; (a) OPh glycoside 3k; (b) SPh glycoside 3l; (c) OMe glycoside 3m.

In order to possibly gain insight into these differences in reaction outcome, molecular structures for each of the acceptors 3k, 3l and 3m were explored using computational methods (see ESI†). First, low energy conformers were identified using the Tinker software^43^ by performing a general conformational search, where the energies were calculated using the MMFF94 force field.^44^ The geometries and energies of these low energy conformers were then refined using density functional theory (DFT) to obtain the final structural, energetic and electronic information (see ESI, Tables S3–S5†). Composite images comprising superpositions of the low energy conformations (namely those within 10 kJ mol^−1^ of the lowest energy conformer) of glycosides 3k,^45^3l and 3m^46^ are shown in Fig. 1 (for full details see ESI, Fig. S1–S3†).

The only significant differences observed were that the phenyl ring of SPh glycoside 3l was found to adopt different conformations than in the corresponding OPh glycoside 3k, in line with previous observations,^47^ presumably as result of the greater C–S bond length and differences in the endo anomeric effect. Low energy conformers the of methyl glycoside 3m comprised rotamers around the C5–C6 bond. No exceptional conformations or data were observed for the OPh glycoside 3k which may have explained the high preference for selective reaction of the 6-OH group. Furthermore, calculations revealed no differences in charge distribution at the reacting centre (O6) for any conformers of the three acceptors (Tables S3–S5†). Thus unfortunately, computational analysis did not provide any clear-cut rationalisation for the observed differences in acceptor regioselectivity.

Next, we investigated if the relative solubilities of the glycosyl acceptors may play a part in altering the regioselectivity of the glycosylation reaction.^48^ Thus, we undertook an assessment of acceptor solubility for several acceptors (3e, 3f, 3k, 3l, 3m) by NMR in a mixture of CD_3_CN and d7-DMF (10 : 1) at concentrations that matched those used for the glycosylation reactions, in the presence of glycosyl oxazoline 1a (see ESI†). Although we found that methyl glycoside acceptor 3m was marginally less soluble than OPh acceptor 3k, all of the other acceptors studied displayed comparable solubility. We therefore concluded that acceptor solubility under the reaction conditions was not a significant contributing factor to reaction regioselectivity.

The lack of complete regioselectivity using alkyl glycoside acceptors intimated that extension of the procedure to trisaccharide synthesis would not be straightforward. Indeed attempted reaction using a pNP-maltoside as acceptor produced a mixture of two trisaccharides, each containing a GlcNAcβ(1→6) linkage (ESI, Table S1†). Other attempted trisaccharide synthesis resulted in non-regioselective reactions (ESI, Table S2†).

Finally, attention turned to the use of other 2-acetamido sugars as donors. Using GalNAc 2b as the glycosyl donor the reaction outcome was found to closely mirror the results found using GlcNAc as donor. Firstly (Table 3, entry 14) the use of the pNP-glucoside acceptor 3a produced the GalNAcβ(1→6)Glc disaccharide 4k as the sole product in 40% yield. Next (entry 15), whilst the use of the pNP-galactoside 3g as acceptor at rt was not completely regioselective, lowering the reaction temperature to −10 °C resulted in completely selective reaction and gave the GalNAcβ(1→6)Gal disaccharide 4l as the sole product in 30% yield. Finally, the use of ManNAc 2c as donor was investigated using acceptor 3a. Although the product yield was significantly lower (15%, entry 16), the 1,2-*trans* α(1→6) linked disaccharide 4m was isolated as the reaction product, demonstrating wider applicability of the basic process, though indicating that in this case further reaction optimisation is required.

## Conclusions

A pragmatic method has been developed for the glycosylation of unprotected 2-acetamido sugars, allowing their direct conversion into either 1,2-*trans* alkyl glycosides, or (1→6)-linked disaccharides. In all cases the transformation is completely stereoselective, though absolute control of regioselectivity of disaccharide formation is dependent on the acceptor used. Whilst the major reaction product is invariably the (1→6)-linked disaccharide, reactions using aryl *O*-glycoside acceptors are completely regioselective. This simple glycosylation process supersedes traditional synthetic methods to access this type of material, which require multiple protecting group manipulations, and protracted reaction sequences.

## Data availability

All data is provided the ESI,† including detailed synthetic procedures, compound characterization, and copies of NMR spectra.

## Author contributions

X. Q. performed all of the synthetic chemistry and compound characterisation. A. L. G. performed all of the modelling and computational studies. A. J. F. conceived and directed the project, and wrote the paper, with input from X. Q. and A. L. G.

## Conflicts of interest

There are no conflicts to declare.

## Supplementary Material

SC-013-D2SC00222A-s001

## References

[cit1] Lairson L. L., Henrissat B., Davies G. J., Withers S. G. (2008). Annu. Rev. Biochem..

[cit2] Liang D.-M., Liu J.-H., Wu H., Wang B.-B., Zhu H.-J., Qiao J.-J. (2015). Chem. Soc. Rev..

[cit3] Liu H., Hansen T., Zhou S. Y., Wen G. E., Liu X. X., Zhang Q. J., Codée J. D. C., Schmidt R. R., Sun J. S. (2019). Org. Lett..

[cit4] Guberman M., Seeberger P. H. (2019). J. Am. Chem. Soc..

[cit5] Downey A. M., Hocek M. (2017). Beilstein J. Org. Chem..

[cit6] Noguchi M., Tanaka T., Gyakushi H., Kobayashi A., Shoda S.-i. (2009). J. Org. Chem..

[cit7] Isobe T., Ichikawa T. (1999). J. Org. Chem..

[cit8] Fairbanks A. J. (2021). Carbohydr. Res..

[cit9] Noguchi M., Fujieda T., Huang W. C., Ishihara M., Kobayashi A., Shoda S.-I. (2012). Helv. Chim. Acta.

[cit10] Tanaka T., Huang W. C., Noguchi M., Kobayashi A., Shoda S.-I. (2009). Tetrahedron Lett..

[cit11] Tanaka T., Nagai H., Noguchi M., Kobayashi A., Shoda S.-I. (2009). Chem. Commun..

[cit12] Lim D., Brimble M. A., Kowalczyk R., Watson A. J. A., Fairbanks A. J. (2014). Angew. Chem., Int. Ed..

[cit13] Tanaka T., Matsumoto T., Noguchi M., Kobayashi A., Shoda S.-I. (2009). Chem. Lett..

[cit14] Yoshida N., Noguchi M., Tanaka T., Matsumoto T., Aida N., Ishihara M., Kobayashi A., Shoda S.-I. (2011). Chem.–Asian J..

[cit15] Novoa A., Barluenga S., Serba C., Winssinger N. (2013). Chem. Commun..

[cit16] Köhling S., Exner M. P., Nojoumi S., Schiller J., Budisa N., Rademann J. (2016). Angew. Chem., Int. Ed. Engl..

[cit17] Alexander S. R., Fairbanks A. J. (2016). Org. Biomol. Chem..

[cit18] Meguro Y., Noguchi M., Li G., Shoda S.-I. (2017). Org. Lett..

[cit19] Li G., Noguchi M., Kashiwagura H., Tanaka Y., Serizawa K., Shoda S.-I. (2016). Tetrahedron Lett..

[cit20] Lim D., Fairbanks A. J. (2017). Chem. Sci..

[cit21] Serizawa K., Noguchi M., Li G., Shoda S.-I. (2017). Chem. Lett..

[cit22] Qiu X., Fairbanks A. J. (2020). Org. Lett..

[cit23] Dimakos V., Taylor M. S. (2018). Chem. Rev..

[cit24] Pelletier G., Zwicker A., Allen C. L., Schepartz A., Miller S. J. (2016). J. Am. Chem. Soc..

[cit25] Shimada N., Sugimoto T., Noguchi M., Ohira C., Kuwashima Y., Takahashi N., Sato N., Makino K. (2021). J. Org. Chem..

[cit26] Essentials of Glycobiology, ed. A. Varki, R. D. Cummings, J. D. Esko, H. H. Freeze, P. Stanley, C. R. Bertozzi, G. W. Hart and M. E. Etzler, Cold Spring Harbor Laboratory Press: Cold Spring Harbor, 2nd edn, 2009 20301239

[cit27] Raetz C. R. H., Whitfield C. (2002). Annu. Rev. Biochem..

[cit28] Beau J.-M., Boyer F. D., Norsikian S., Urban D., Vauzeilles B., Xolin A. (2018). Eur. J. Org. Chem..

[cit29] Banoub J., Boullanger P., Lafont D. (1992). Chem. Rev..

[cit30] Li C., Wang L.-X. (2018). Chem. Rev..

[cit31] Kortüm G. , Vogel W. and Andrussow K., Dissociation Constants of Organic Acids in Aqueous Solution, Butterworths, 1961

[cit32] Zurabyan S. E., Volosyuk T. P., Khorlin A. J. (1969). Carbohydr. Res.

[cit33] Wittmann V., Lennartz D. (2002). Eur. J. Org. Chem..

[cit34] Blatter G., Beau J. M., Jacquinet J. C. (1994). Carbohydr. Res..

[cit35] Cai Y., Ling C.-C., Bundle D. R. (2005). Org. Lett..

[cit36] Excoffier G., Gagnaire D., Utile J. P., Vignon M. (1972). Tetrahedron Lett..

[cit37] Gudmundsdottir A., Nitz M. (2008). Org. Lett..

[cit38] Rana S. S., Barlow J. J., Matta K. L. (1981). Carbohydr. Res..

[cit39] Matta K. L., Barlow J. J. (1977). Carbohydr. Res..

[cit40] Zurabyan S. E., Volosyuk T. P., Khorlin A. J. (1969). Carbohydr. Res..

[cit41] Fialová P., Carmona A. T., Robina I., Ettrich R., Sedmera P., Přikrylová V., Petrásková-Hušáková L., Křen V. (2005). Tetrahedron Lett..

[cit42] van der Vorm S., Hansen T., van Hengst J. M. A., Overkleeft H. S., van der Marel G. A., Codée J. D. C. (2019). Chem. Soc. Rev..

[cit43] Rackers J. A., Wang Z., Lu C., Laury M. L., Lagardère L., Schnieders M. J., Piquemal J. P., Ren P., Ponder J. W. (2018). J. Chem. Theory Comput..

[cit44] Halgren T. A. (1995). J. Comput. Chem..

[cit45] Jones P. G., Sheldrick G. M., Clegg W., Kirby A. J., Glenn R. (1982). Z. Kristallogr. - New Cryst. Struct..

[cit46] Tvaroška I., Taravel F. R., Utille J. P., Carver J. P. (2002). Carbohydr. Res..

[cit47] Ohlsson J., Sundin A., Nilsson U. J. (2003). Chem. Commun..

[cit48] We thank a referee for suggesting these experiments

