# Supplementary material for: Protecting group free glycosylation: one-pot stereocontrolled access to 1,2-trans glycosides and (1→6)-linked disaccharides of 2-acetamido sugars
Source: Chem Sci. 2022 Mar 17;13(14):4122–30. doi: 10.1039/d2sc00222a (PMC8985506; doi:10.1039/d2sc00222a)

Electronic Supplementary Material (ESI) for Chemical Science.
This journal is © The Royal Society of Chemistry 2022

SUPPORTING INFORMATION

Protecting group free glycosylation: one step stereocontrolled access to 1,2-*trans* glycosides and (1→6)-linked disaccharides of 2-acetamido sugars

Xin Qiu,[a] Anna L. Garden,[b] and Antony J Fairbanks*[a,c]

a) School of Physical and Chemical Sciences, University of Canterbury, Private Bag 4800, Christchurch, 8140, New Zealand

b) Department of Chemistry, University of Otago, Dunedin 9054, New Zealand and The MacDiarmid Institute for Advanced Materials and Nanotechnology, Victoria University of Wellington, Wellington 6140, New Zealand.

c) Biomolecular Interaction Centre, University of Canterbury, Private Bag 4800, Christchurch, 8140, New Zealand

Table of contents

1. Previous routes to GlcNAcβ(1→6) linked disaccharides: Schemes S1-S3 .... S2
2. Attempted trisaccharide synthesis; Tables S1, and S2 .... S4
3. Molecular modelling; Figures S1, S2, and S3, and Tables S3, S4, and S5 .... S5
4. Acceptor solubility studies S9
5. General experimental procedures S16
6. Experimental and characterization of compounds S18
7. Experimental and characterization of (1→3)-linked isomers S34
8. Experimental and characterization of trisaccharides S36
9. Experimental references S37
10. NMR spectra of compounds S381

Scheme S1: Previous synthetic routes to GlcNAcβ(1→6)ManαO*p*NP disaccharide **4b** and corresponding methyl glycoside

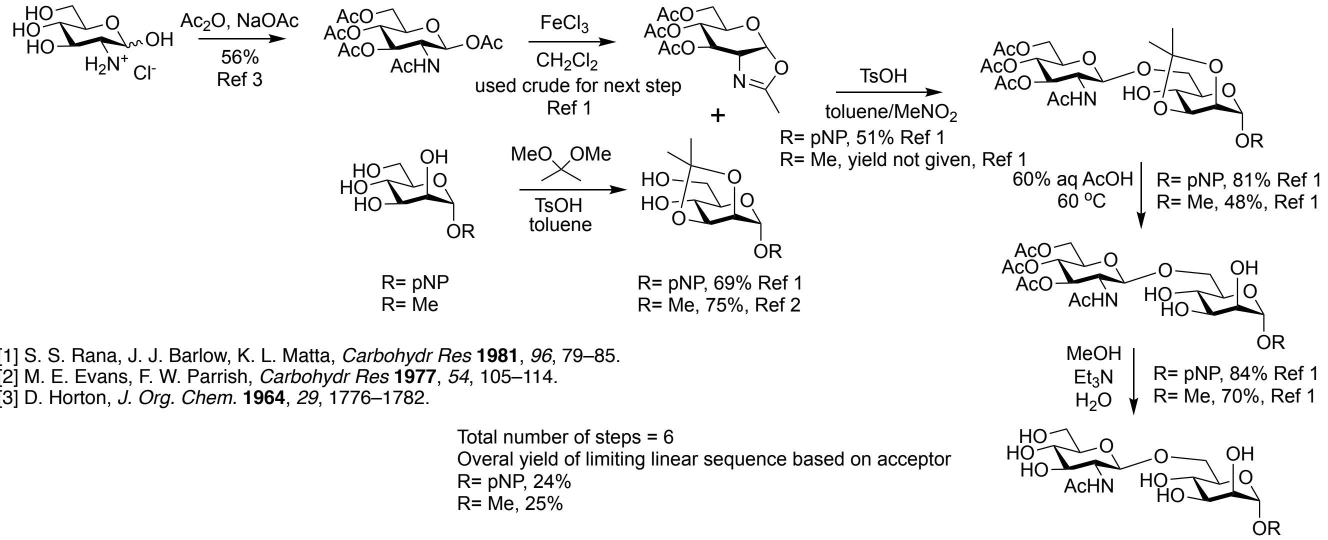

[1] S. S. Rana, J. J. Barlow, K. L. Matta, *Carbohydr Res* **1981**, *96*, 79–85.
[2] M. E. Evans, F. W. Parrish, *Carbohydr Res* **1977**, *54*, 105–114.
[3] D. Horton, *J. Org. Chem.* **1964**, *29*, 1776–1782.

Scheme S2: Previous synthetic route to GlcNAcβ(1→6)GalβO*p*NP disaccharide **4g**

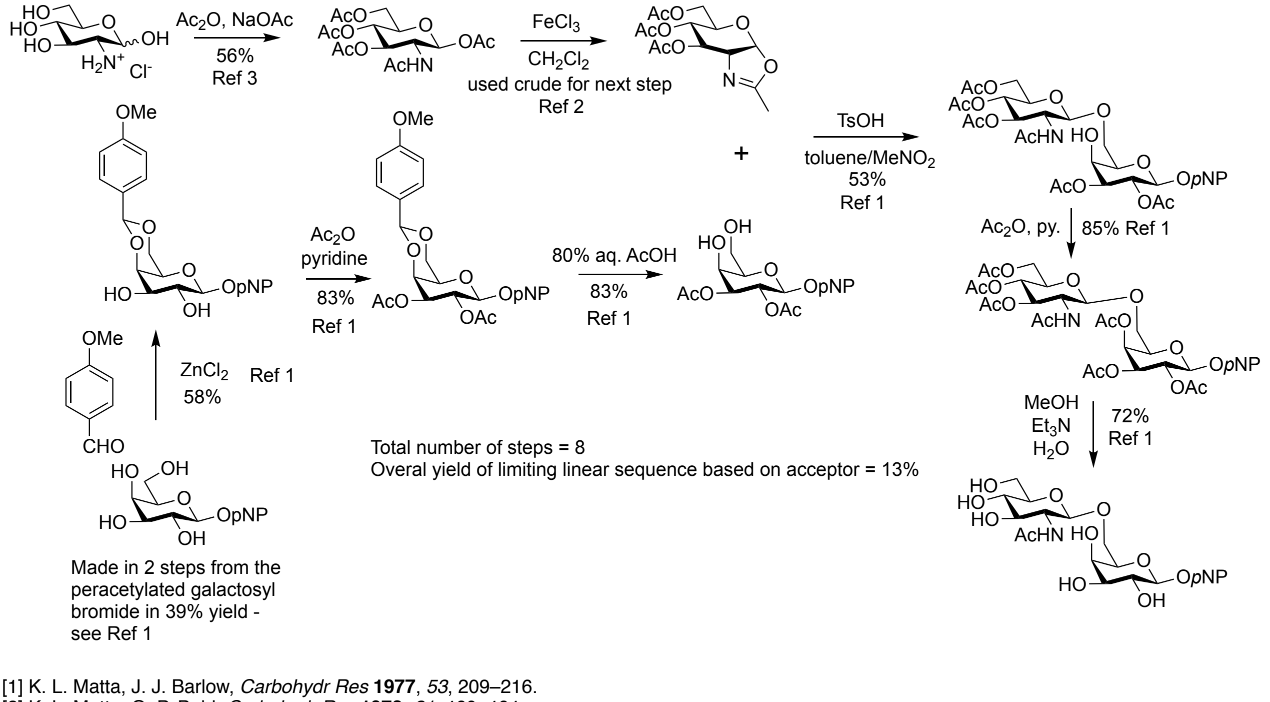

[1] K. L. Matta, J. J. Barlow, *Carbohydr Res* **1977**, *53*, 209–216.
[2] K. L. Matta, O. P. Bahl, *Carbohydr Res* **1972**, *21*, 460–464.
[3] D. Horton, *J. Org. Chem.* **1964**, *29*, 1776–1782.

Scheme S3: Previous synthetic routes to GlcNAcβ(1→6)GlcNAcβOpNP disaccharide **4h**

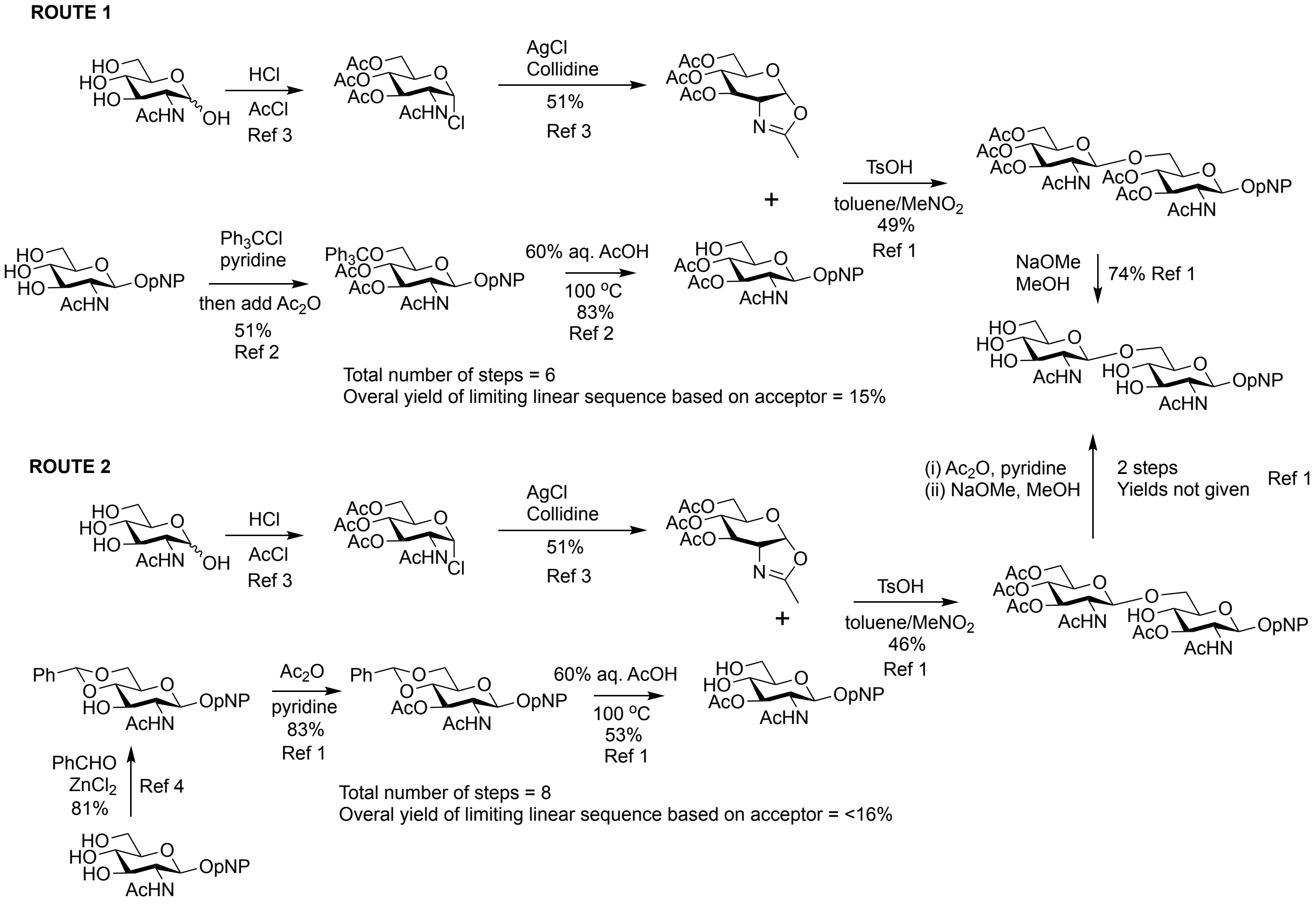

[1] S. E. Zurabyan, T. P. Volosyuk, A. J. Khorlin, *Carbohydr Res* **1969**, *9*, 215–220.
[2] T. Osawa, *Carbohydr Res* **1966**, *1*, 435–443.
[3] A.Ya. Khorlin, M. L. Shul'man ,S. E. Zurabyan, I. M. Privalova, Yu. L. Kopaveich, *Izv. Akad. Nauk SSSR, Ser. Khim.* **1968**, 2094.
[4] R. W. Jeanloz, E. Walker, P. Sinaÿ, *Carbohydr Res* **1968**, *6*, 184–196.

Table S1: Attempted trisaccharide synthesis using *p*NP-maltoside as the acceptor.

Entry	Donor	Acceptor	Products	Yield
1	GlcNAc	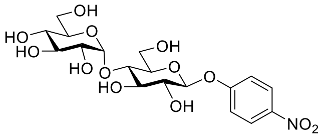	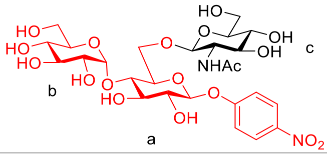	29%
			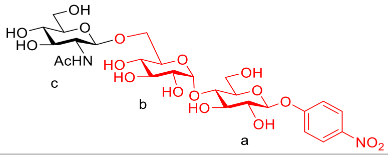	15%
2[a]	GlcNAc	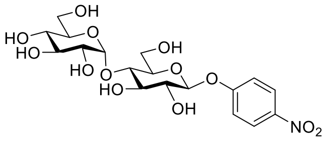	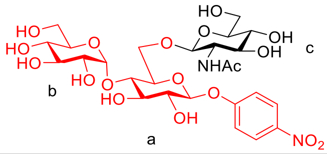	19%
			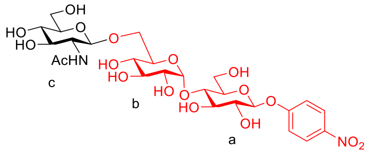	11%

a: The reaction was performed at -10 °C.

Table S2: Attempted trisaccharide synthesis using either a (1→6) linked disaccharide acceptor, or a disaccharide donor.

Entry	Donor	Acceptor	Results
1	GlcNAc	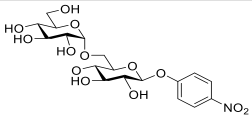	25%, as a mixture of regioisomers
2	GlcNAc	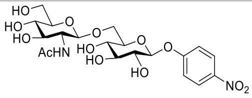	20%, as a mixture of regioisomers
3	Man-β(1-4)GlcNAc[1]	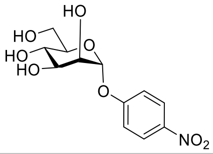	21%, as a mixture of regioisomers
4	Man-β(1-4)GlcNAc[1]	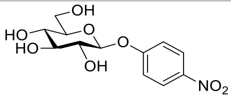	39%, as a mixture of regioisomers
5[a]	Man-β(1-4)GlcNAc[1]	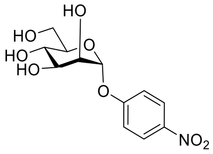	22%, as a mixture of regioisomers

a: The reaction was performed at -10 °C.

Molecular Modelling Studies

Conformational searching was performed using the Tinker software [1] (module SCAN) with the MMFF94 force field [2a-e]. All conformers within ~40 kJ mol^{-1} of the lowest energy structure were retained for refinement with density functional theory (DFT).

DFT calculations were performed using the ORCA program version 4.0 [3a,b]. Structures were fully optimized using the PW6B95 functional [4] with the ma-def2-TZVP basis set, which includes diffuse functions.[5] D3 dispersion corrections with Becke-Johnson damping were also applied.[6] SCF iterations were considered converged when the energy change was less than 1×10^{-8} a.u. The geometry was considered optimized when the following tolerances were met:

maximum gradient = 3×10^{-4} a.u.
RMS gradient = 1×10^{-4} a.u.
maximum displacement = 4×10^{-3} a.u.
RMS displacement = 2×10^{-3} a.u.

To reduce numerical error in the DFT integration, more grid points were used for both the angular and radial grids *via* the keyword “Grid6” for the SCF iterations and “Grid7” for the final energy evaluation.
Partial charges on selected atoms were calculated using Löwdin population analysis [7].

Modelling references

[1] Rackers, J. A., Wang, Z., Lu, C., Laury, M. L., Lagardère, L., Schnieders, M. J., Piquemal, J. P., Ren, P., & Ponder, J. W. Tinker 8: Software Tools for Molecular Design. *J. Chem. Theory Comput.* **2018**, *14*, 5273–5289. https://doi.org/10.1021/acs.jctc.8b00529

[2a] T. A. Halgren, "Merck Molecular Force Field. I. Basis, Form, Scope, Parametrization, and Performance of MMFF94", *J. Comput. Chem*. **1995**, *17*, 490-519.

[2b] T. A. Halgren, "Merck Molecular Force Field. II. MMFF94 van der Waals and Electrostatic Parameters for Intermolecular Interactions", *J. Comput. Chem.* **1995**, *17*, 520-552.

[2c] T. A. Halgren, "Merck Molecular Force Field. III. Molecular Geometries and Vibrational Frequencies for MMFF94", *J. Comput. Chem*. **1995**, *17*, 553-586.

[2d] T. A. Halgren and R. B. Nachbar, "Merck Molecular Force Field. IV. Conformational Energies and Geometries for MMFF94", *J. Comput. Chem.* **1995**, *17*, 587-615.

[2e] T. A. Halgren, "Merck Molecular Force Field. V. Extension of MMFF94 Using Experimental Data, Additional Computational Data, and Empirical Rules", *J. Comput. Chem.* **1995**, *17*, 616-641.

[3a] Neese, F., The ORCA program system, *Wiley Interdiscip. Rev.: Comput. Mol. Sci.* **2012**, 2, 73-78.

[3b] Neese, F. Software update: the ORCA program system, version 4.0, *Wiley Interdiscip. Rev.: Comput. Mol. Sci.* **2017**, *8*, e1327.

[4] Y. Zhao and D. G. Truhlar, *J. Phys. Chem. A* **2005**, *109*, 5656–5667

[5] Zheng, J., Xu, X. & Truhlar, D.G. Minimally augmented Karlsruhe basis sets. *Theor. Chem. Acc* **2011**, *128,* 295–305. https://doi.org/10.1007/s00214-010-0846-z

[6] S. Grimme, S. Ehrlich and L. Goerigk, *J. Comput. Chem.* **2011**, *32*, 1456–1465

[7] Per-Olov Löwdin , "On the Non-Orthogonality Problem Connected with the Use of Atomic Wave Functions in the Theory of Molecules and Crystals", *J. Chem. Phys.* **1950**, *18*, 365-375. https://doi.org/10.1063/1.1747632

Figure S1 Low energy conformations of OPh glycosyl acceptor **3k**

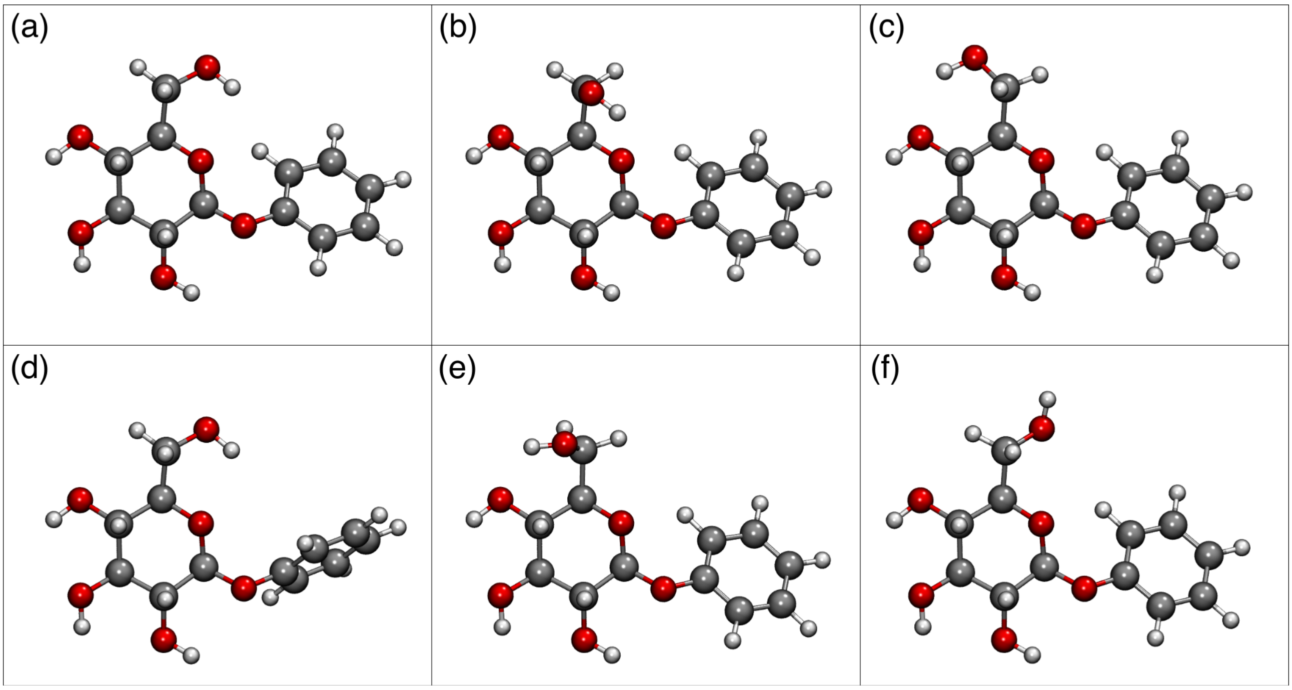

Table S3 Data for low energy conformations of OPh glycosyl acceptor **3k**

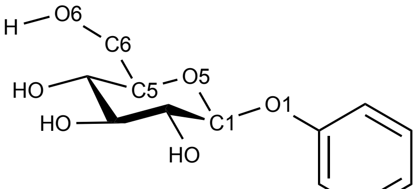

3k

Conformer	Relative E / kJ mol^{-1}	O5-C1-O1 bond angle	O5-C1 bond length	C1-O1 bond length	C5-C6 bond length	Partial charge on O6
a	0.00	109.3	1.400	1.384	1.509	-0.12
b	1.43	109.2	1.401	1.386	1.512	-0.11
c	3.38	109.5	1.402	1.385	1.517	-0.12
d	8.20	109.9	1.400	1.389	1.508	-0.11
e	9.71	109.8	1.401	1.386	1.520	-0.11
f	9.93	109.8	1.399	1.386	1.502	-0.12

Figure S2 Low energy conformations of SPh glycosyl acceptor **3l**

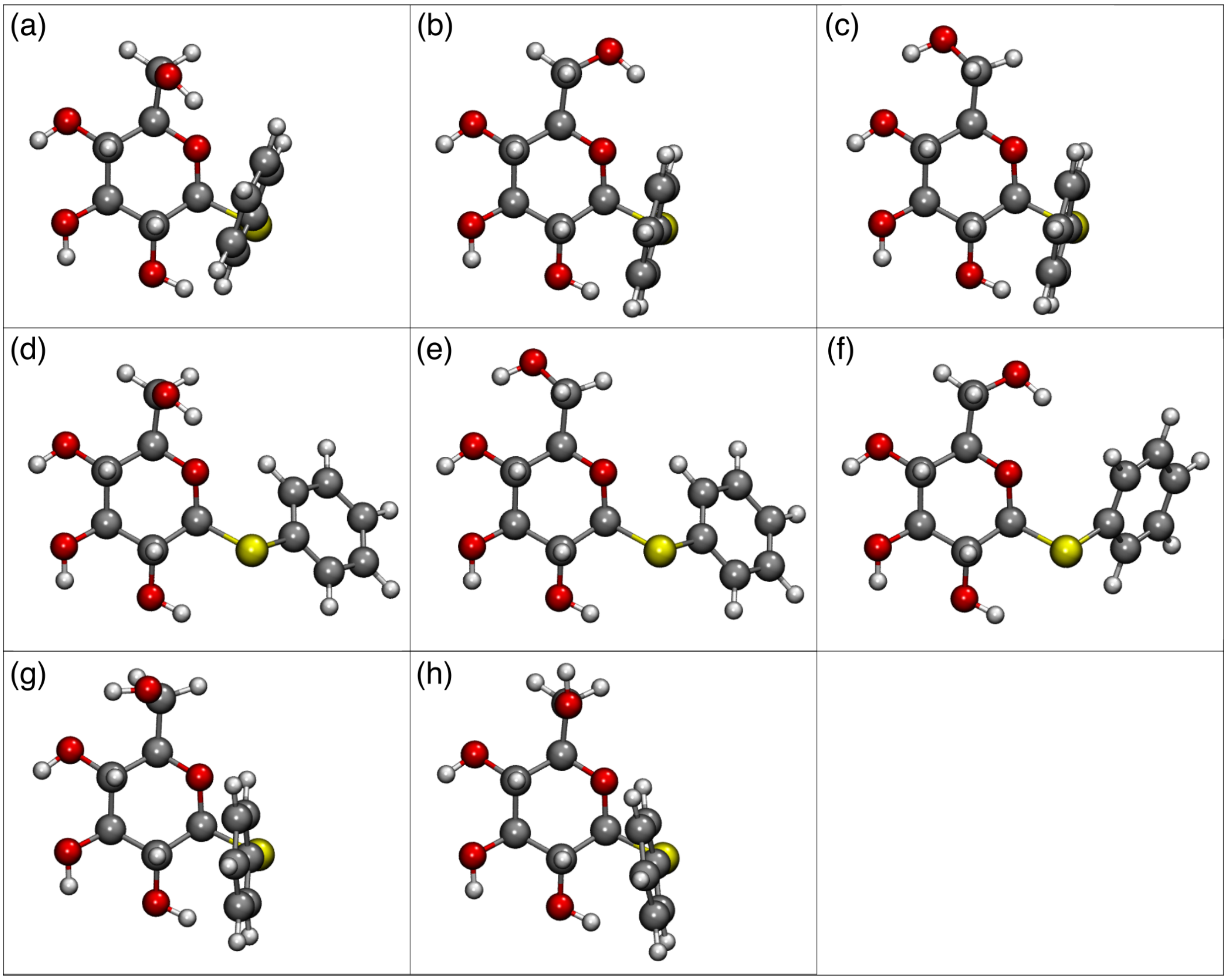

Table S4 Data for low energy conformations of SPh glycosyl acceptor **3l**

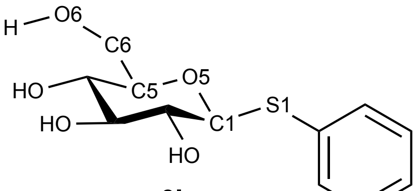

3l

Conformer	Relative E / kJ mol^{-1}	O5-C1-S1 bond angle	O5-C1 bond length	C1-S1 bond length	C5-C6 bond length	Partial charge on O6
a	0.00	109.3	1.397	1.816	1.5133	-0.12
b	0.84	110.5	1.401	1.808	1.507	-0.12
c	2.62	110.5	1.401	1.809	1.517	-0.12
d	3.08	110.0	1.405	1.799	1.512	-0.11
e	4.97	110.3	1.406	1.799	1.517	-0.12
f	5.67	109.9	1.403	1.803	1.507	-0.12
g	5.80	110.3	1.401	1.808	1.516	-0.12
h	9.59	110.0	1.399	1.808	1.506	-0.12

Figure S3 Low energy conformations of OMe glycosyl acceptor **3m**

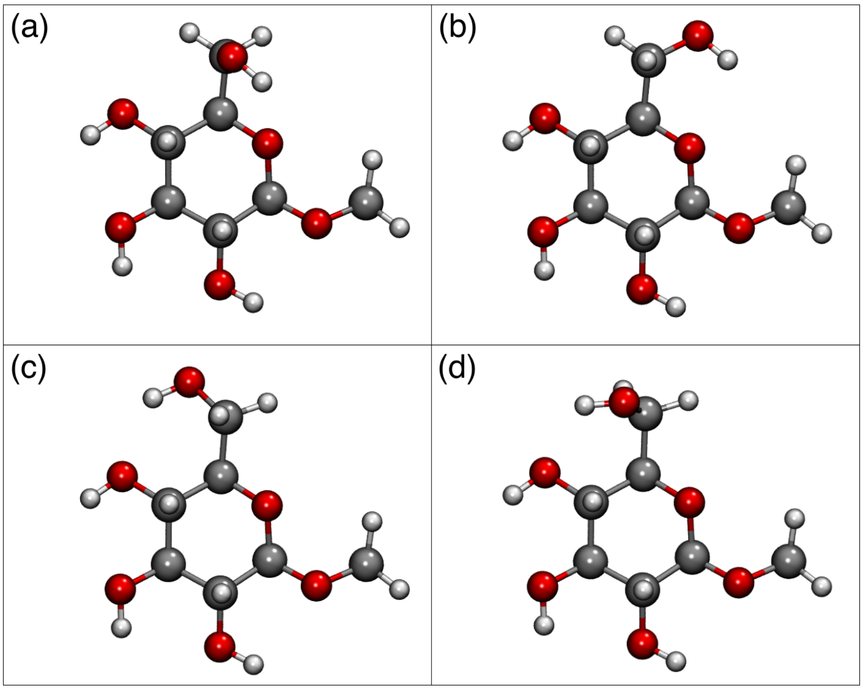

Table S5 Data for low energy conformations of OMe glycosyl acceptor **3m**

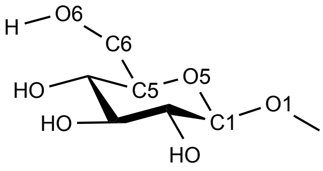

3m

Conformer	Relative E / kJ mol^{-1}	O5-C1-O1 bond angle	O5-C1 bond length	C1-O1 bond length	C5-C6 bond length	Partial charge on O6
a	0.00	109.5	1.406	1.377	1.512	-0.12
b	0.61	109.8	1.407	1.376	1.508	-0.12
c	1.30	109.7	1.407	1.376	1.517	-0.12
d	7.22	109.9	1.406	1.377	1.519	-0.12

NMR Solubility studies on selected glycosyl acceptors under the reaction conditions

Two sets of investigations were undertaken using pre-made oxazoline as follows:

a) *N*-Acetyl-D-glucosamine (12 mg, 54 μmol) and triethylamine (70 μL, 0.48 mmol) were stirred in water (0.3 mL) at 0 °C. DMC (27 mg, 0.16 mmol) was added, and the solution was stirred for 30 min. The solution was then diluted by the addition of water (0.6 mL), and freeze-dried. The residue was dissolved in a mixture of MeCN-d_3 (1.2 mL) and DMF-d_7 (0.12 mL). The solution was divided into 3 samples; each sample contained oxazoline (4 mg, 18 μmol, 1 equiv.). To these three samples were added

Phenyl β-D-glucopyranoside **3k** (23 mg, 90 μmol, 5 equiv.)

Phenyl β-D-thioglucopyranoside **3l** (25 mg, 90 μmol, 5 equiv.)

Methyl β-D-glucopyranoside **3m** (17 mg, 90 μmol, 5 equiv.)

Each resulting solution was then analysed by ^{1}H NMR, and the acceptor solubility assessed by comparison of the integrals of the H1 protons for the acceptor and glycosyl oxazoline.

b) *N*-Acetyl-D-glucosamine (16 mg, 72 μmol) and triethylamine (93 μL, 0.65 mmol) were stirred in water (0.4 mL) at 0 °C. DMC (36 mg, 0.22 mmol) was added, and the solution was stirred for 30 min. The solution was then diluted by the addition of water (0.8 mL), and freeze-dried. The residue was dissolved in a mixture of MeCN-d_3 (1.6 mL) and DMF-d_7 (0.16 mL) to form an oxazoline solution.

Phenyl β-D-glucopyranoside **3k** (23 mg, 90 μmol, 5 equiv.) was dissolved in the oxazoline solution (440 μL).

Acetyl α-D-mannopyranoside **3e** (25.4 mg, 114 μmol, 5 equiv.) was dissolved in the oxazoline solution (559 μL).

α-D-Mannopyranosyl fluoride **3f** (15.3 mg, 84 μmol, 5 equiv.) was dissolved in the oxazoline solution (408 μL).

Each resulting solution was then analysed by ^{1}H NMR, and the acceptor solubility assessed by comparison of the integrals of the H1 protons for the acceptor and glycosyl oxazoline.

a) Solubility of acceptor **3k**

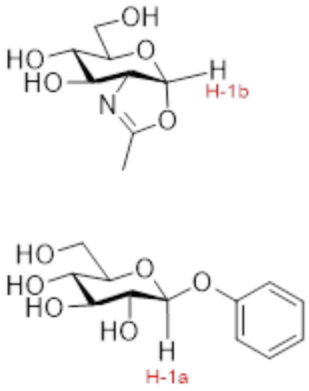

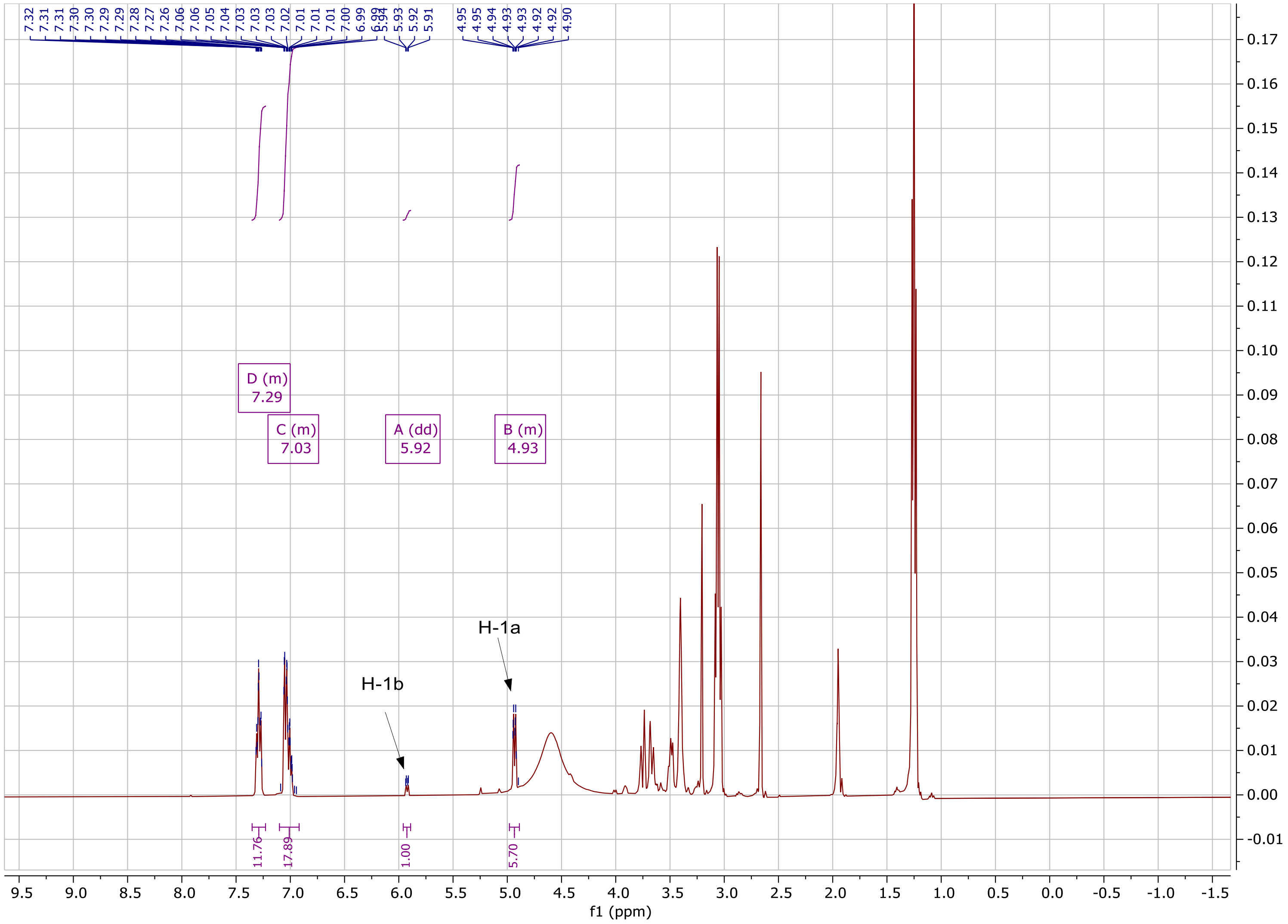

a) Solubility of acceptor **3l**

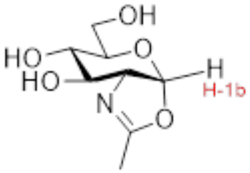

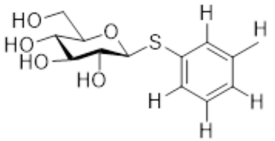

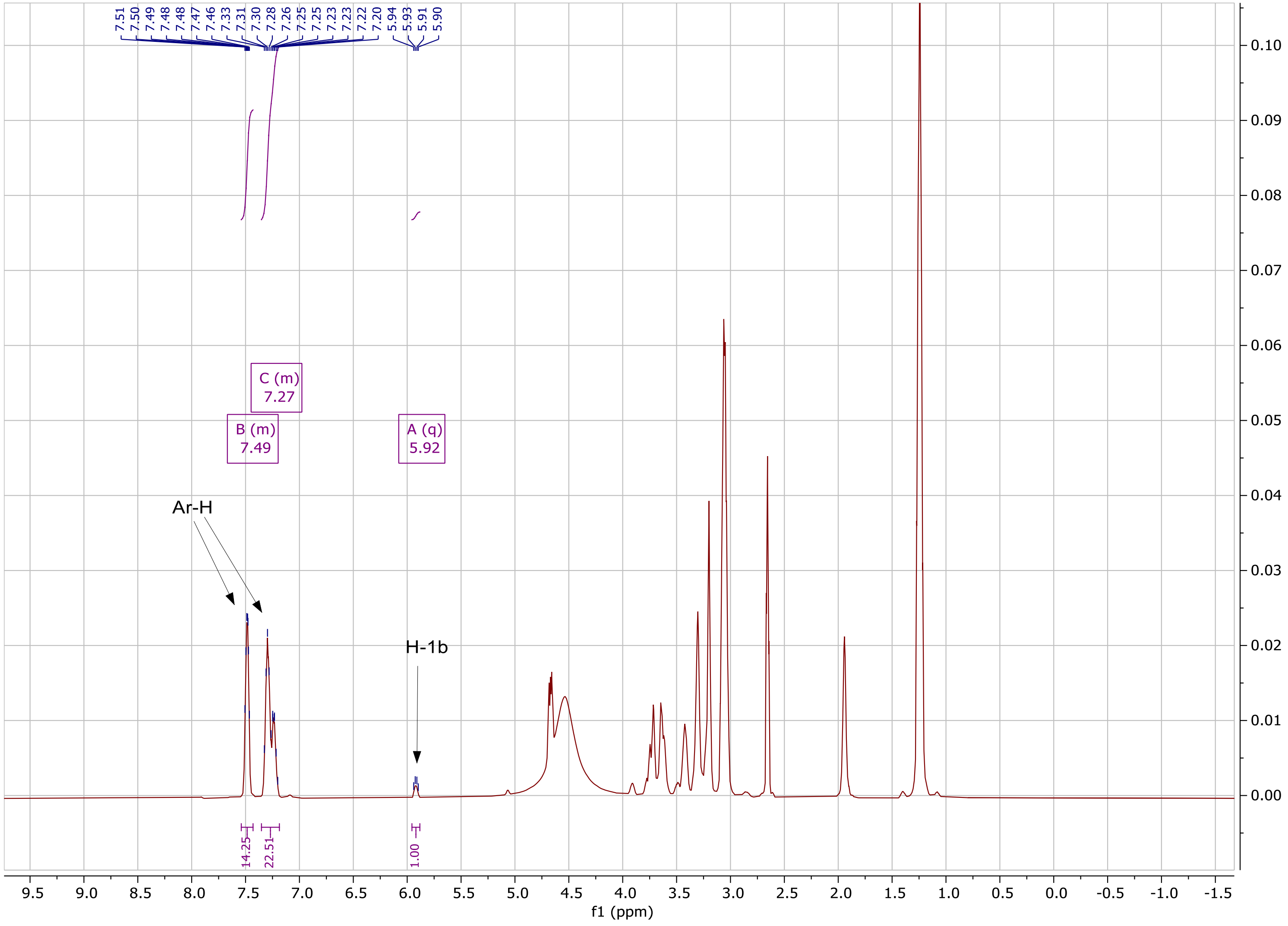

a) Solubility of acceptor **3l**

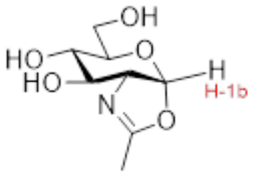

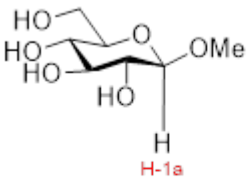

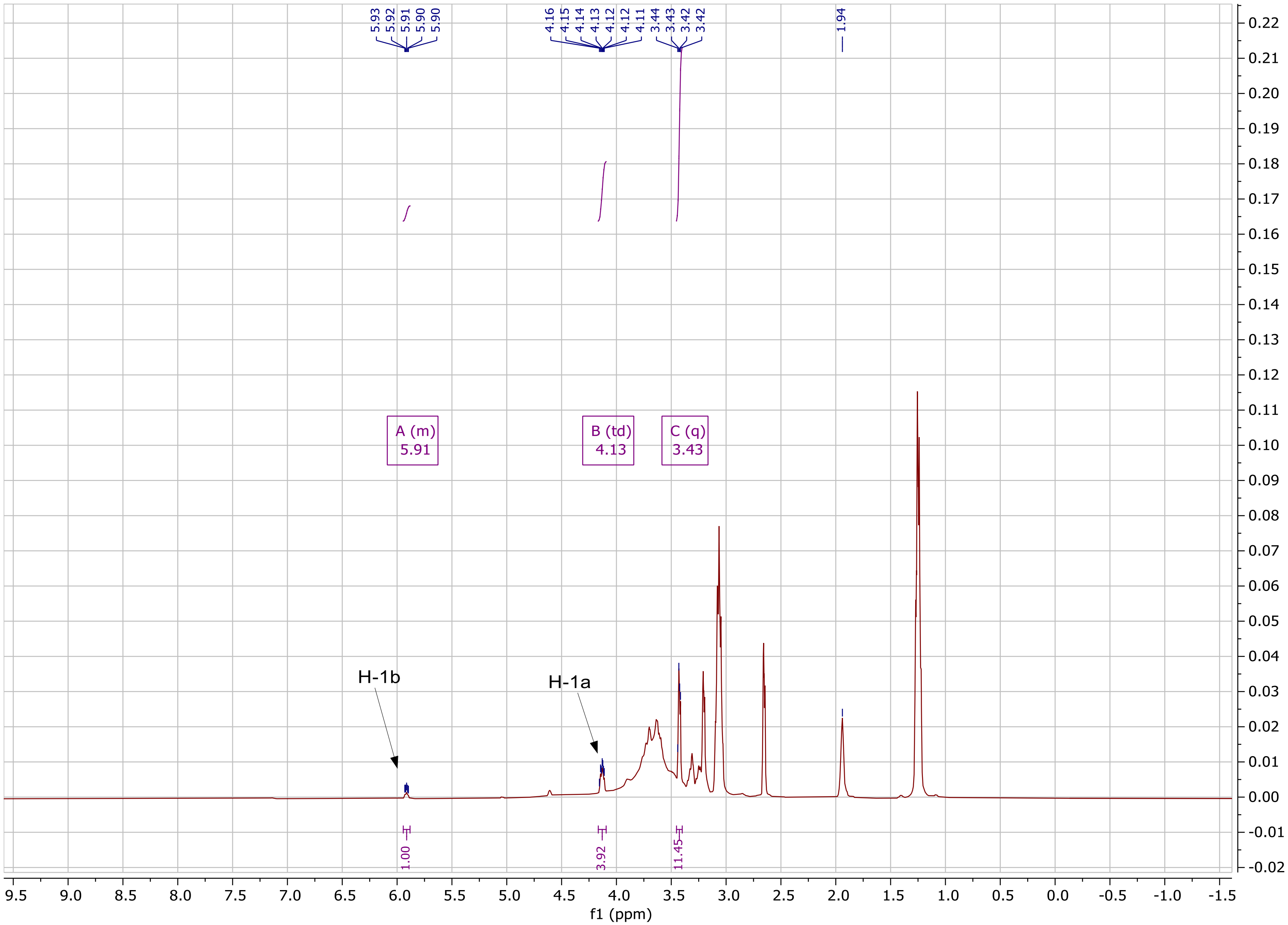

b) Solubility of acceptor **3k**

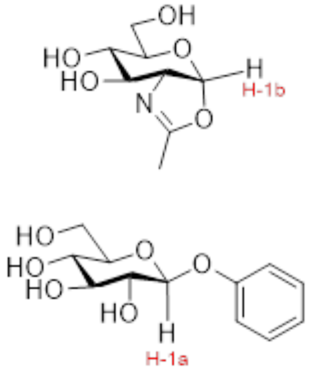

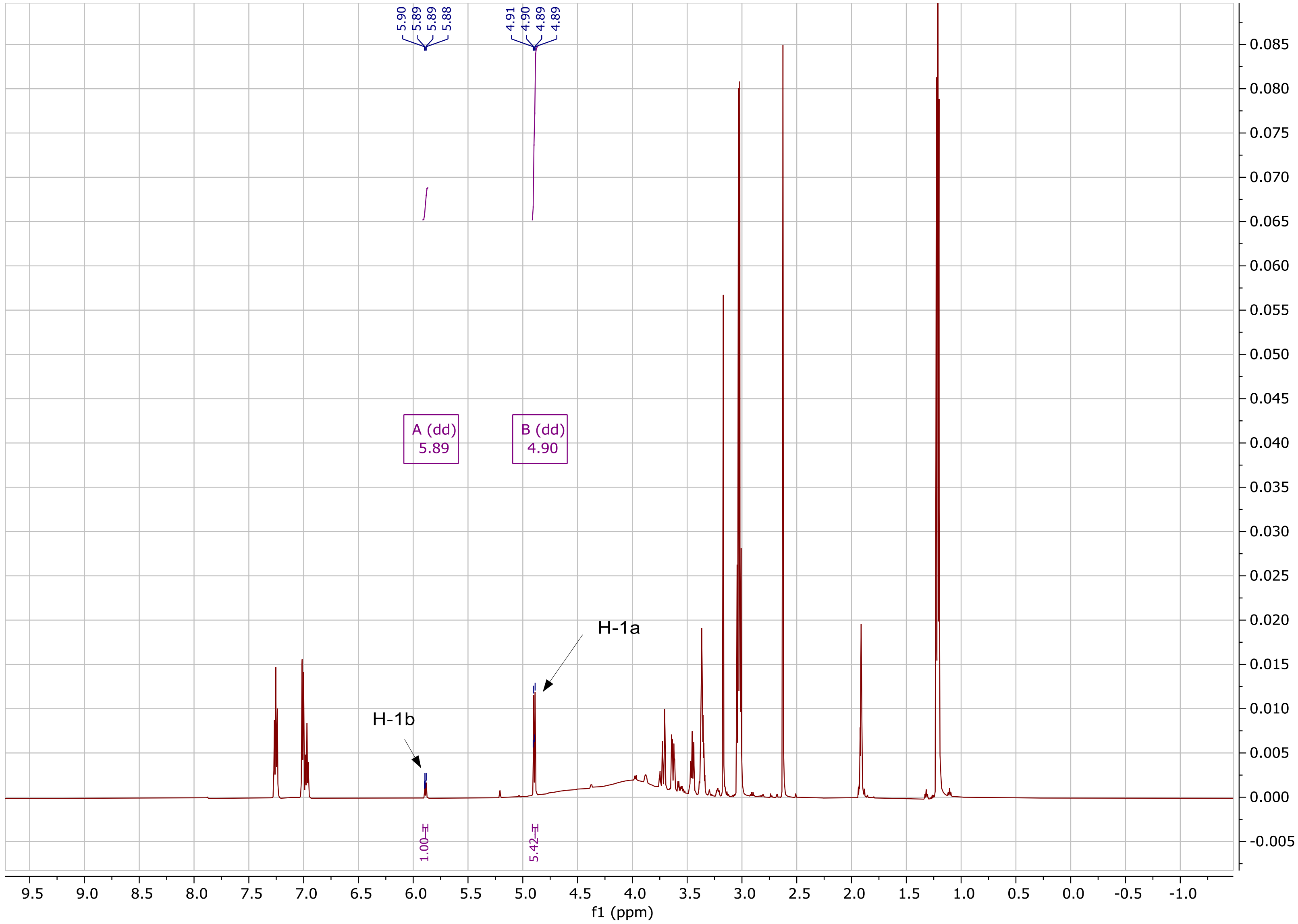

b) Solubility of acceptor **3e**

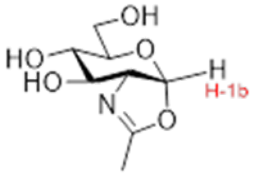

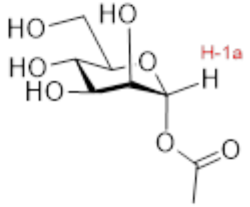

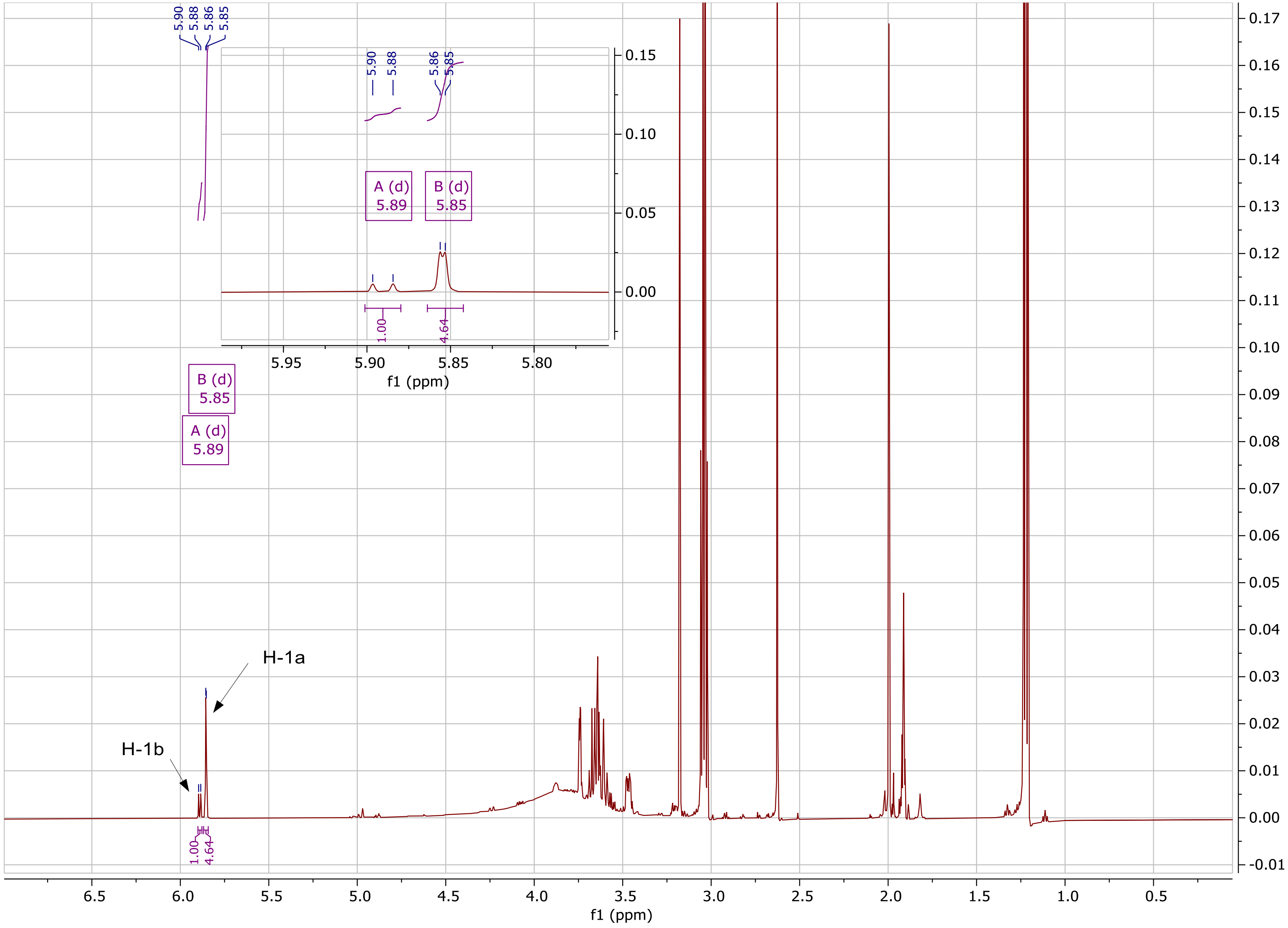

b) Solubility of acceptor **3f**

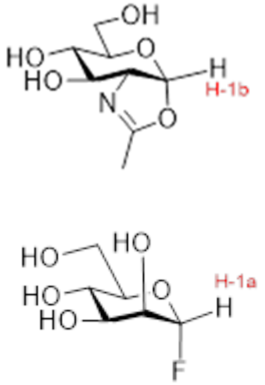

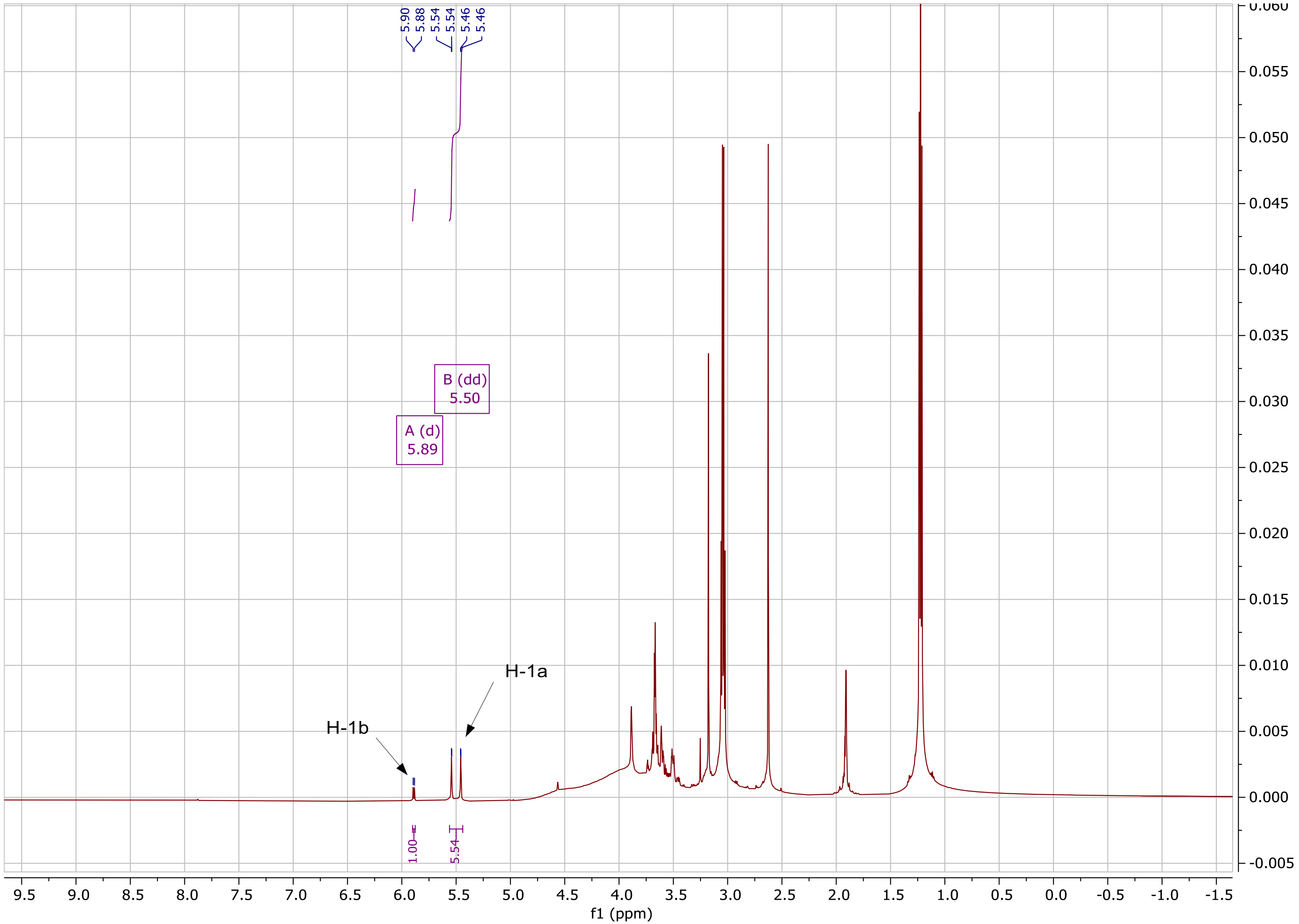

General Experimental

Reactions conducted at 0 °C were cooled by means of an ice bath. Reactions conducted at -10 °C or -16 °C were cooled using a Julabo FP45 cryostat. Solvent was removed under reduced pressure using a Buchi™ rotary evaporator. Anhydrous solvents were dried using a custom-built 'Grubbs' Inert Solvent Purification System, using alumina columns (see *Organometallics* **1996**, *15*, 1518). Molecular sieves were 4Å powered, and were dried by heating under vacuum for activation prior to use. Other reagents were used as supplied without further purification unless otherwise stated. Thin Layer Chromatography (t.l.c.) was carried out on Merck Silica Gel $60F_{254}$ aluminium-backed plates. Visualisation of the plates was achieved using a UV lamp (λ_{max} = 254 or 365 nm), and/or ammonium molybdate (5% in 2M H_2SO_4), and/or aniline-diphenylamine-85% phosphoric acid (4 mL :4 g :20 mL) in acetone (96 mL). Flash column chromatography was carried out using Sorbsil C60 40/60 silica. Melting points were recorded on an Electrothermal® melting point apparatus. Proton and carbon nuclear magnetic resonance (δ_H, δ_C) spectra were recorded on JEOL ECZ400S and JEOL ECZ600R spectrometers. All chemical shifts are quoted on the δ-scale in ppm using residual solvent as an internal standard. ^{1}H and ^{13}C spectra were assigned using COSY, DEPT, HSQC, HMQC, HMBC and TOCSY. High resolution mass spectra were recorded a Bruker maXis 3G UHR-TOF mass spectrometer using electrospray ionization (ESI) or chemical ionization (CI) techniques as stated. M/z values are reported in Daltons. Optical rotations were measured on a Rudolph Research Analytical AUTOPOL IV automatic polarimeter with a Ceramic Quartz cell with a path length of 50 mm and inner diameter of 5 mm, and are quoted in units of $°.cm^2.g^{-1}$.

General procedure A: glycosylation of alcohols (see Table 2)

N-Acetyl-D-glucosamine **2a** (typically 40 mg, 0.18 mmol, 1 equiv.) and triethylamine (0.22 mL, 1.59 mL, 9 equiv.) were stirred in water (typically 1 mL) at 0 °C. DMC (88 mg, 0.54 mmol, 3 equiv.) was added, and the solution was stirred for 30 min. After this time, t.l.c. ($CHCl_3$:MeOH, 2:1) indicated the consumption of starting material (R_f 0.3) and the formation of a major product (R_f 0.5). The solution was then diluted by the addition of water (typically 2 mL), and freeze-dried. The residue was dissolved in the alcohol (typically 4.4 mL) and activated powdered molecular sieves (typically 1.6 g) were added. The mixture was then stirred under nitrogen at room temperature for 30 min. Dry TsOH (31 mg, 0.18 mmol, 1 equiv.) was added,

and the mixture was stirred at room temperature for a further 3 h. After this time, t.l.c. ($CHCl_3$:MeOH, 2:1) indicated the complete consumption of oxazoline (R_f 0.5) and the formation of a major product (R_f 0.6). Sodium bicarbonate (15 mg, 0.18 mmol, 1 equiv.) and H_2O (typically 1 mL) was added, and the solution was diluted with MeOH (typically 50 mL), and filtered through a pad of Celite®. The filtrate was concentrated, 35% w/w aqueous ammonium hydroxide (typically ~10 mL) was added, and the mixture then concentrated *in vacuo* to remove triethylamine. This procedure was repeated a second time. The residue was dissolved in MeOH (typically 20 mL) and pre-absorbed on to a pad of silica and then purified by flash column chromatography (typical solvent system $CHCl_3$:MeOH, 10:1) to give the pure b-glycoside product **5a-d**.

General procedure B: disaccharide synthesis (see Table 3)

The unprotected glycosyl donor **2a-c** (typically 40 mg, 0.18 mmol, 1 equiv.) and triethylamine (0.22 mL, 1.59 mmol, 9 equiv.) were stirred in water (typically 1 mL) at 0 °C. DMC (88 mg, 0.54 mmol, 3 equiv.) was added, and the solution was stirred for 30 min. The solution was then diluted by the addition of water (typically 2 mL), and freeze-dried. The residue was dissolved in a mixture of dry MeCN (typically 4 mL) and dry DMF (typically 0.4 mL). The glycosyl acceptor **3a-1, 5d** (0.9 mmol, 5 equiv.) was then added, and the mixture was sonicated for 1 min until it became clear. Activated powdered molecular sieves (typically 1.6 g) were then added, and the solution was stirred under nitrogen at room temperature for 30 min. After this time, t.l.c. (typical solvent system $CHCl_3$:MeOH, 2:1) indicated the presence of oxazoline (R_f 0.5). Dry TsOH (31 mg, 0.18 mmol, 1 equiv.) was added, and the mixture was stirred at the specified temperature (rt, 0 °C, or -10 °C) for a further 3 h. After this time, t.l.c. ($CHCl_3$:MeOH, 2:1) indicated the consumption of oxazoline (R_f 0.5) and the formation of a major product (typically R_f 0.1). Sodium bicarbonate (15 mg, 0.18 mmol, 1 equiv.) and H_2O (typically 1 mL) were added, the solution was diluted with MeOH (50 mL), and then filtered through a pad of Celite®. The filtrate was concentrated, dissolved in MeOH (typically 20 mL), pre-absorbed onto a pad of silica, and purified by flash column chromatography ($CHCl_3$:MeOH, 6:1 to 3:1) to give the pure disaccharide product.

n-Pentenyl 2-acetamido-2-deoxy-β-D-glucopyranoside 5a[2]

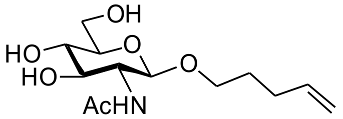

Method 1: General Procedure A, with *N*-acetyl-D-glucosamine **2a** (40 mg, 0.18 mmol), triethylamine (0.22 mL, 1.59 mmol), DMC (88 mg, 0.54 mmol) in water (1 mL), and then *n*-pentenol (4.4 mL), powdered molecular sieves (1.6 g), and then TsOH (31 mg, 0.18 mmol). Purification by flash column chromatography ($CHCl_3$:MeOH, 10:1) gave *n*-pentenyl 2-acetamido-2-deoxy-β-D-glucopyranoside **5a** (38 mg, 73%) as a white solid, m.p. 160-165 °C (MeOH) [lit 184 °C][2]; $[\alpha]_D^{22}$ -28 (*c*, 0.1 in H_2O) [lit. $[\alpha]_D^{25}$ -27 (*c*, 0.9 in MeOH)][2]; δ_H (400 MHz, D_2O)[2] 1.56-1.65 (2H, m, $OCH_2\mathit{CH_2}CH_2$), 2.00 (3H, s, $NHCOC\mathit{H}_3$), 2.01-2.08 (2H, m, $OCH_2CH_2C\mathit{H_2}$), 3.37-3.41 (2H, m, H-4, H-5), 3.46-3.52 (1H, m, H-3), 3.56 (1H, dt, *J* 10.3 Hz, 6.5 Hz, $OC\mathit{H}_aH_b$), 3.64 (1H, dd, $J_{2,3}$ 10.3 Hz, $J_{1,2}$ 8.5 Hz, H-2), 3.67-3.73 (1H, m, H-6), 3.82-3.90 (2H, m, H-6', $OCH_a\mathit{H}_b$), 4.46 (1H, $J_{1,2}$ 8.5 Hz, H-1), 4.95-5.05 (2H, m, $\mathit{CH_2}$=CH), 5.84 (1H, m, CH_2=C*H*). HRMS (ESI) m/z: $[M + Na]^+$ Calcd. For $C_{13}H_{23}NO_6Na$ 312.1418; Found 312.1421.

Method 2: Following Procedure A with minor modication, with *N*-Acetyl-D-glucosamine **2a** (40 mg, 0.18 mmol), triethylamine (0.22 mL, 1.59 mmol), DMC (88 mg, 0.54 mmol) in water (1 mL), and then a mixtute of MeCN (4 mL) and DMF (0.4 mL) as the reaction solvent, and *n*-pentenol (92 µL, 0.9 mmol), powdered molecular sieves (1.6 g), and then TsOH (31 mg, 0.18 mmol). Purification by flash column chromatography ($CHCl_3$:MeOH, 10:1) gave *n*-pentenyl 2-acetamido-2-deoxy-β-D-glucopyranoside **5a** (25 mg, 48% identical to the material described above).

Benzyl 2-acetamido-2-deoxy-β-D-glucopyranoside 5b[2-3]

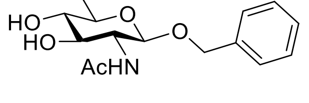

General Procedure A, with *N*-acetyl-D-glucosamine **2a** (40 mg, 0.18 mmol), triethylamine (0.22 mL, 1.59 mmol), DMC (88 mg, 0.54 mmol) in water (1 mL), and then benzyl alcohol (4.4 mL), powdered molecular sieves (1.6 g), and TsOH (31 mg, 0.18 mmol). Purification by flash column chromatography ($CHCl_3$:MeOH, 10:1) gave benzyl 2-acetamido-2-deoxy-β-D-glucopyranoside **5b** (42 mg, 75%) as a white solid, m.p. 183-185 °C (MeOH) [lit. 191-192 °C][3]; $[\alpha]_D^{22}$ -21.6 (*c*, 0.5 in H_2O) [lit. $[\alpha]_D^{20}$ -29 (*c*, 0.93 in H_2O)];[3] δ_H (400 MHz, D_2O)[2] 1.88 (3H, s,

NHCOCH_3), 3.37-3.45 (3H, m, H-3, H-4, H-5), 3.61-3.68 (1H, at, *J* 9.0 Hz, H-2), 3.69-3.75 (1H, dd, $J_{6,6'}$ 12.7 Hz, $J_{5,6}$ 4.5 Hz, H-6), 3.90 (1H, ad, *J* 12.0 Hz, H-6'), 4.47 (1H, d, $J_{1,2}$ 8.5 Hz, H-1), 4.62 (1H, d, *J* 12.2 Hz, OCH_aH$_b$), 4.84 (1H, d, *J* 12.2 Hz, OCH$_a$CH_b), 7.30-7.44 (5H, m, Ar-H). HRMS (ESI) m/z: $[M + Na]^+$ Calcd. For $C_{15}H_{21}NO_6Na$ 334.1261; Found 334.1259.

Isopropyl 2-acetamido-2-deoxy-β-D-glucopyranoside 5c[2, 4]

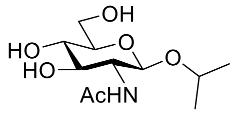

General Procedure A with *N*-acetyl-D-glucosamine **2a** (40 mg, 0.18 mmol), triethylamine (0.22 mL, 1.59 mmol), DMC (88 mg, 0.54 mmol) in water (1 mL), and then isopropanol (4.4 mL), powdered molecular sieves (1.6 g), and TsOH (31 mg, 0.18 mmol). Purification by flash column chromatography ($CHCl_3$:MeOH, 10:1) gave isopropyl 2-acetamido-2-deoxy-β-D-glucopyranoside **5c** (36 mg, 76%) as a white solid, m.p. 196-198 °C (MeOH); $[\alpha]_D^{22}$ -36 (*c*, 0.2 in H_2O) [lit. $[\alpha]_D^{23}$ -5 (*c*, 0.48 in MeOH)];[4] δ_H (400 MHz, D_2O)[2] 1.08 (3H, d, *J* 6.1 Hz, OCHCH_3), 1.15 (3H, d, *J* 6.2 Hz, OCHCH_3), 1.99 (3H, s, NHCOCH_3), 3.33-3.43 (2H, m, H-4, H-5), 3.45-3.51 (1H, dd, $J_{2,3}$ 10.2 Hz, $J_{1,2}$ 8.1 Hz, H-3), 3.54-3.61 (1H, dd, $J_{1,2}$ 8.4 Hz, $J_{2,3}$ 10.2Hz, H-2), 3.68 (1H, dd, $J_{6,6'}$ 12.3 Hz, $J_{5,6}$ 5.3 Hz, H-6), 3.87 (1H, dd, $J_{6,6'}$ 12.3 Hz, $J_{5,6'}$ 1.9 Hz, H-6'), 3.97 (1H, sept, *J* 6.1 Hz, OCH(CH$_3$)$_2$), 4.54 (1H, d, $J_{1,2}$ 8.4 Hz, H-1). HRMS (ESI) m/z: $[M + Na]^+$ Calcd. For $C_{11}H_{21}NO_6Na$ 286.1261; Found 286.1266.

***t*-Butyl 2-acetamido-2-deoxy-β-D-glucopyranoside 5d**[2]

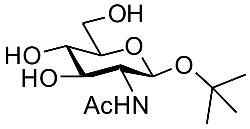

General Procedure A with *N*-acetyl-D-glucosamine **2a** (40 mg, 0.18 mmol), triethylamine (0.22 mL, 1.59 mmol), DMC (88 mg, 0.54 mmol) in water (1 mL), then *t*-butanol (4.4 mL), powdered molecular sieves (1.6 g), TsOH (31 mg, 0.18 mmol). Purification by flash column chromatography ($CHCl_3$:MeOH, 10:1) gave *t*-butyl 2-acetamido-2-deoxy-β-D-glucopyranoside **5d** (23 mg, 45%) as a white solid, m.p. 140-150 °C (MeOH), $[\alpha]_D^{22}$ -12 (*c*, 0.2 in H_2O); δ_H (400 MHz, D_2O)[2] 1.17 (9H, s, 3 × CH_3), 1.99 (3H, s, NHCOCH_3), 3.35 (1H, dd, $J_{4,5}$ 9.8 Hz, $J_{3,4}$ 8.0 Hz, H-4),

3.38-3.43 (1H, m, H-5), 3.51 (1H, dd, $J_{2,3}$ 10.4 Hz, $J_{3,4}$ 8.0 Hz, H-3), 3.57 (1H, dd, $J_{2,3}$ 10.4 Hz, $J_{1,2}$ 8.0 Hz, H-2), 3.66 (1H, dd, $J_{6,6'}$ 12.3 Hz, $J_{5,6}$ 5.8 Hz, H-6), 3.84 (1H, dd, $J_{6,6'}$ 12.3 Hz, $J_{5,6'}$ 2.2 Hz, H-6'), 4.63 (1H, d, $J_{1,2}$ 8.0 Hz, H-1). HRMS (ESI) m/z: $[M + Na]^+$ Calcd. For $C_{12}H_{23}NO_6Na$ 300.1418; Found 300.1420.

Benzyl 2-acetamido-2-deoxy-β-D-galactopyranoside 5e[5-6]

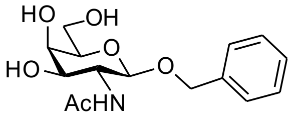

General Procedure A, with *N*-acetyl-D-galactosamine **2b** (40 mg, 0.18 mmol), triethylamine (0.22 mL, 1.59 mmol), DMC (88 mg, 0.54 mmol) in water (1 mL), and then benzyl alcohol (4.4 mL), powdered molecular sieves (1.6 g), and TsOH (31 mg, 0.18 mmol). Purification by flash column chromatography ($CHCl_3$:MeOH, 10:1) gave benzyl 2-acetamido-2-deoxy-β-D-galactopyranoside **5e** (24 mg, 43%) as a white solid, m.p. 185-195 °C (MeOH, dec) [lit. 210-212 °C (MeOH-Et_2O)][5][lit. 195-197 °C (2-propanol)][6]; $[\alpha]_D^{22}$ -5.2 (*c*, 0.5 in H_2O) [lit. $[\alpha]_D^{23}$ -3.4 (*c*, 0.5 in H_2O)][5]; δ_H (400 MHz, D_2O)[6] 1.86 (3H, s, NHCOCH_3), 3.55-3.65 (2H, m, H-3, H-5), 3.73 (1H, d, $J_{6,6'}$ 11.7 Hz, $J_{5,6}$ 4.4 Hz, H-6), 3.76-3.88 (3H, m, H-2, H-4, H-6'), 4.41 (1H, d, $J_{1,2}$ 8.5 Hz, H-1), 4.63 (1H, d, *J* 12.2 Hz, OCH_aH$_b$), 4.83 (1H, under water peak, OCH$_a$$H_b$), 7.30-3.46 (5H, m, Ar-H).

Benzyl 2-acetamido-2-deoxy-α-D-mannopyranoside 5f[7-8]

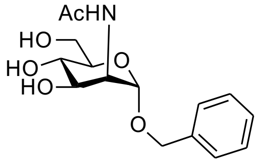

General Procedure A, with *N*-acetyl-D-mannosamine **2c** (40 mg, 0.18 mmol), triethylamine (0.22 mL, 1.59 mmol), DMC (88 mg, 0.54 mmol) in water (1 mL), and then benzyl alcohol (4.4 mL), powdered molecular sieves (1.6 g), and TsOH (31 mg, 0.18 mmol). Purification by flash column chromatography ($CHCl_3$:MeOH, 12:1) gave benzyl 2-acetamido-2-deoxy-α-D-mannopyranoside **5f** (32 mg, 57%) as a colorless oil; $[\alpha]_D^{22}$ +56.4 (*c*, 0.5 in H_2O) [lit. $[\alpha]_D^{20}$ +75 (*c*, 1.1 in MeOH)][7]; δ_H (400 MHz, CD_3OD)[8] 2.00 (3H, s, NHCOCH_3), 3.55-3.65 (2H, m, H-4, H-5), 3.78-3.83 (2H, H-6, H-6'), 3.96 (1H, aq, *J* 4.9 Hz, H-3), 4.35 (1H, ad, *J* 4.6 Hz, H-2), 4.51 (1H, d, *J* 11.7 Hz, OCH_aH$_b$), 4.72 (1H, d, *J* 11.7 Hz, OCH$_a$$H_b$), 4.78 (1H, brs, H-1), 7.26-7.40 (5H, m, Ar-H).

***p*-Nitrophenyl 2-acetamido-2-deoxy-β-D-glucopyranosyl-(1→6)-β-D-glucopyranoside 4a**

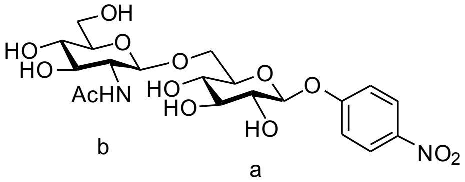

General Procedure B with *N*-acetyl-D-glucosamine **2a** (100 mg, 0.45 mmol), triethylamine (0.56 mL, 4.1 mmol), DMC (223 mg, 1.36 mmol) in water (2.5 mL), and then MeCN (10 mL), DMF (1 mL), *p*-nitrophenyl β-D-glucopyranoside **3a** (683 mg, 2.26 mmol), powdered molecular sieves (4 g), and TsOH (82 mg, 0.45 mmol) at rt. Purification by flash column chromatography ($CHCl_3$:MeOH, 6:1 until the excess **3a** had been eluted, then $CHCl_3$:MeOH, 3:1) gave *p*-nitrophenyl 2-acetamido-2-deoxy-β-D-glucopyranosyl-(1→6)-β-D-glucopyranoside **4a** (111 mg, 49%) as a white solid, m.p. 167-175 °C (MeOH, dec); $[\alpha]_D^{23}$ -95 (*c*, 0.4 in H_2O); ν_{max} (neat) 3304 (s, OH), 1620, 1572 (2 × s, amide), 1512, 1346 (2 × s, N=O) cm^{-1}; δ_H (400 MHz, D_2O) 1.78 (3H, s, NHCOCH_3), 3.35-3.50 (4H, m, H-4a, H-3b, H-4b, H-5b), 3.53-3.62 (2H, m, H-2a, H-3a), 3.64-3.74 (3H, m, H-2b, H-6a, H-6b), 3.81 (1H, ddd, $J_{4a,5a}$ 9.9 Hz, $J_{5a,6a}$ 5.7 Hz, $J_{5a,6a'}$ 1.9 Hz, H-5a), 3.86 (1H, dd, $J_{6b,6b'}$ 12.2 Hz, $J_{5b,6b}$ 1.8 Hz, H-6b'), 4.20 (1H, dd, $J_{6a,6a'}$ 11.2 Hz, $J_{5a,6a'}$ 1.9 Hz, H-6a), 4.47 (1H, d, $J_{1b,2b}$ 8.4 Hz, H-1b), 5.22 (1H, d, $J_{1a,2a}$ 7.4 Hz, H-1a), 7.19 (2H, d, *J* 9.3 Hz, Ar-H), 8.23 (2H, d, *J* 9.3 Hz, Ar-H); δ_C (150 MHz, D_2O) 22.0 (q, NHCOCH_3), 55.4 (d, C-2b), 60.7 (t, C-6b), 68.2 (t, C-6a), 69.4, 69.9, 73.9, 75.9 (4 × d, C-4a, C-3b, C-4b, C-5b), 72.6, 75.3 (2 × d, C-2a, C-3a), 74.8 (d, C-5a), 99.3 (d, C-1a), 101.2 (d, C-1b), 116.4, 126.2 (2 × d, Ar-C), 142.6 161.7 (2 × s, Ar-C), 174.4 (s, C=O). HRMS (ESI) m/z: $[M + Na]^+$ Calcd. For $C_{20}H_{28}N_2O_{13}Na$ 527.1484; Found 527.1473.

p-Nitrophenyl 2-acetamido-2-deoxy-β-D-glucopyranosyl-(1→6)-α-D-mannopyranoside 4b[9]

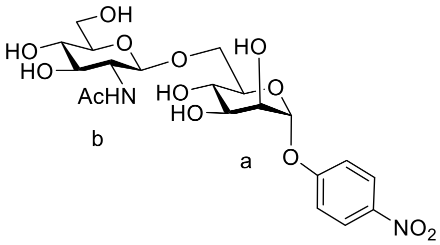

General Procedure B with *N*-acetyl-D-glucosamine **2a** (40 mg, 0.18 mmol), triethylamine (0.22 mL, 1.59 mmol), DMC (88 mg, 0.54 mmol) in water (1 mL), and then MeCN (4 mL), DMF (0.4 mL), *p*-nitrophenyl α-D-mannopyranoside **3b** (270 mg, 0.89 mmol), powdered molecular sieves (1.6 g), and TsOH (31 mg, 0.18 mmol) at rt. Purification by flash column chromatography ($CHCl_3$:MeOH, 6:1 until the excess **3b** had been eluted, then $CHCl_3$:MeOH, 3:1) gave *p*-nitrophenyl 2-acetamido-2-deoxy-β-D-glucopyranosyl-(1→6)-α-D-mannopyranoside **4b** (42 mg, 47%) as a white solid, m.p. 185-190 °C (MeOH/$CHCl_3$, dec) [lit. 189-191 °C][9], $[\alpha]_D^{22}$ +25.2 (*c*, 0.5 in H_2O) [lit. $[\alpha]_D$ +71.5 (*c*, 1 in DMSO)][9]; ν_{max} (neat) 3302 (s, OH), 1630 (s, amide), 1512, 1345 (s, N=O) cm^{-1}; δ_H (400 MHz, D_2O) 1.98 (3H, s, NHCOCH_3), 3.30 (1H, dd, $J_{3b,4b}$ 8.6 Hz, $J_{4b,5b}$ 9.8 Hz, H-4b), 3.37 (1H, dd, $J_{4b,5b}$ 9.8 Hz, $J_{5b,6b}$ 5.9 Hz, $J_{5b,6b'}$ 2.1 Hz, H-5b), 3.46 (1H, dd, $J_{2b,3b}$ 10.3 Hz, $J_{3b,4b}$ 8.7 Hz, H-3b), 3.56-3.65 (2H, m, H-2b, H-6b), 3.70-3.78 (3H, m, H-4a, H-5a, H-6a), 3.83 (1H, dd, $J_{6b,6b'}$ 12.3 Hz, $J_{5b,6b'}$ 2.1 Hz, H-6b'), 3.98-4.06 (2H, m, H-3a, H-6a'), 4.14 (1H, dd, $J_{2a,3a}$ 3.4 Hz, $J_{1a,2a}$ 1.9 Hz, H-2a), 4.47 (1H, d, $J_{1b,2b}$ 8.4 Hz, H-1b), 5.69 (1H, d, $J_{1a,2a}$ 1.9 Hz, H-1a), 7.23 (2H, d, *J* 9.3 Hz, Ar-H), 8.23 (2H, d, *J* 9.3 Hz, Ar-H); δ_C (150 MHz, D_2O) 22.2 (q, NHCOCH_3), 55.5 (d, C-2b), 60.8 (t, C-6b), 66.5, (d, C-4a), 68.5 (t, C-6a), 69.6 (d, C-2a), 70.0 (d, C-4b), 70.3 (d, C-3a), 72.5 (d, C-5a),73.9 (d, C-3b), 75.9 (d, C-5b), 97.9 (d, C-1a), 101.3 (d, C-1b), 116.8, 126.1 (2 × d, Ar-C), 142.3, 161.0 (2 × s, Ar-C), 174.5 (s, C=O). HRMS (ESI) m/z: $[M + Na]^+$ Calcd. For $C_{20}H_{28}N_2O_{13}Na$ 527.1484; Found 527.1486.

Methyl 2-acetamido-2-deoxy-β-D-glucopyranosyl-(1→6)-α-D-mannopyranoside 4c[9-10]

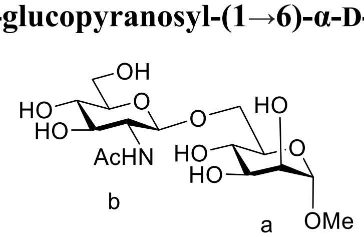

General Procedure B with *N*-acetyl-D-glucosamine **2a** (40 mg, 0.18 mmol), triethylamine (0.22 mL, 1.59 mmol), DMC (88 mg, 0.54 mmol) in water (1 mL), and then MeCN (4 mL),

DMF (0.4 mL), methyl α-D-mannopyranoside **3c** (175 mg, 0.9 mmol), powdered molecular sieves (1.6 g), and TsOH (31 mg, 0.18 mmol) at rt. Purification by flash column chromatography ($CHCl_3$:MeOH, 5:1 until the excess **3c** had been eluted, then $CHCl_3$:MeOH, 3:1) gave methyl 2-acetamido-2-deoxy-β-D-glucopyranosyl-(1→6)-α-D-mannopyranoside **4c** (18 mg, 25%) as a white solid, m.p. 155-162 °C (MeOH, dec); $[\alpha]_D^{22}$ +8 (*c*, 0.3 in H_2O) [lit. $[\alpha]_D^{24}$ +11.2 (*c*, 1 in H_2O)];[9] δ_H (400 MHz, D_2O)[10] 1.99 (3H, s, NHCOCH_3), 3.34 (3H, s, OCH_3), 3.33-3.45 (2H, m, H-4b, H-5b), 3.47-3.58 (2H, m, H-4a, H-3b), 3.64-3.74 (5H, m, H-3a, H-5a, H-6a, H-2b, H-6b), 3.86-3.92 (2H, m, H-2a, H-6b'), 4.15 (1H, ad, *J* 9.2 Hz, H-6a'), 4.51 (1H, d, $J_{1b,2b}$ 8.5 Hz, H-1b), 4.68 (1H, d, $J_{1a,2a}$ 1.7 Hz, H-1a). HRMS (ESI) m/z: $[M + Na]^+$ Calcd. For $C_{15}H_{27}NO_{11}Na$ 420.1476; Found 420.1479.

_p_-Methoxyphenyl 2-acetamido-2-deoxy-β-D-glucopyranosyl-(1→6)-α-D-mannopyranoside 4d

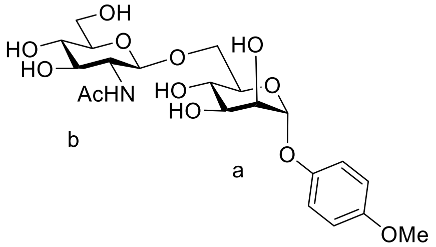

General Procedure B with *N*-acetyl-D-glucosamine **2a** (20 mg, 0.09 mmol), triethylamine (0.11 mL, 0.80 mmol), DMC (44 mg, 0.27 mmol) in water (0.5 mL), and then DMF (2.2 mL), *p*-methoxyphenyl α-D-mannopyranoside **3d** (129 mg, 0.45 mmol), powdered molecular sieves (800 mg), and TsOH (16 mg, 0.09 mmol) at rt. Purification by flash column chromatography ($CHCl_3$:MeOH, 6:1 until the excess **3d** had been eluted, then $CHCl_3$:MeOH, 3:1) gave *p*-methoxyphenyl 2-acetamido-2-deoxy-β-D-glucopyranosyl-(1→6)-α-D-mannopyranoside **4d** (18 mg, 40%) as a white solid, m.p. 168-174 °C (MeOH/$CHCl_3$, dec); $[\alpha]_D^{22}$ +18 (*c*, 0.7 in H_2O); ν_{max} (neat) 3313 (s, OH), 1640, 1563 (2 × s, amide) cm^{-1}, δ_H (600 MHz, D_2O) 1.95 (3H, s, NHCOCH_3), 3.33-3.43 (2H, m, H-4b, H-5b), 3.47 (1H, at, *J* 9.2 Hz, H-3b), 3.63-3.68 (2H, m, H-2b, H-6b), 3.70 (1H, ad, *J* 10.0 Hz, H-4a), 3.74-3.78 (2H, m, H-6a), 3.79 (3H, s, OCH_3), 3.84-3.89 (2H, m, H-5a, H-6b'), 3.98 (1H, dd, $J_{3a,4a}$ 9.5 Hz, $J_{2a,3a}$ 3.4 Hz, H-3a), 4.04 (1H, ad, *J* 11.5 Hz, H-6a'), 4.11-4.15 (1H, m, H-2a), 4.49 (1H, d, $J_{1b,2b}$ 8.5 Hz, H-1b), 5.44 (1H, s, H-1a), 6.97 (1H, d, *J* 8.9 Hz, Ar-H), 7.10 (1H, d, *J* 9.1 Hz, Ar-H); δ_C (150 MHz, D_2O) 22.2 (q, NHCOCH_3), 55.5 (d, C-2b), 55.9 (q, OCH_3), 60.8 (t, C-6b), 66.8 (d, C-4a), 68.6 (t, C-6a), 69.9, 70.0, 75.9 (3 × d, C-2a, C-4b, C-5b), 70.5 (d, C-3a), 72.4 (d,

C-5a), 74.0 (d, C-3b), 99.3 (d, C-1a), 101.2 (d, C-1b), 115.2, 118.9 (2 × d, Ar-C), 149.8, 154.7 (2 × s, Ar-C), 174.6 (s, C=O). HRMS (ESI) m/z: $[M + Na]^+$ Calcd. For $C_{21}H_{31}NO_{12}Na$ 512.1738; Found 512.1732.

Acetyl 2-acetamido-2-deoxy-β-D-glucopyranosyl-(1→6)-α-D-mannopyranoside 4e

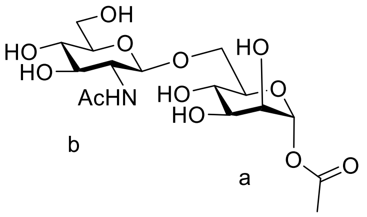

General Procedure B with *N*-acetyl-D-glucosamine **2a** (40 mg, 0.18 mmol), triethylamine (0.22 mL, 1.59 mmol), DMC (88 mg, 0.54 mmol) in water (1 mL), and then MeCN (4 mL), DMF (0.4 mL), acetyl α-D-mannopyranoside **3e** (200 mg, 0.9 mmol), powdered molecular sieves (1.6 g), and TsOH (31 mg, 0.18 mmol) at rt. Purification by flash column chromatography ($CHCl_3$:MeOH, 5:1 until the excess **3e** had been eluted, then $CHCl_3$:MeOH, 3:1) gave acetyl 2-acetamido-2-deoxy-β-D-glucopyranosyl-(1→6)-α-D-mannopyranoside **4e** (27 mg, 36%) as a colorless syrup; $[\alpha]_D^{22}$ +2 (*c*, 0.4 in H_2O); ν_{max} (neat) 3276 (s, OH), 1731 (s, C=O), 1640, 1556 (2 × s, amide) cm^{-1}; δ_H (600 MHz, D_2O) 2.02 (3H, s, NHCOCH_3), 2.12 (3H, s, $OCOCH_3$), 3.38-3.45 (2H, m, H-4b, H-5b), 3.52 (1H, dd, *J* 10.5 Hz, 8.0 Hz, H-3b), 3.62-3.75 (4H, m, H-4a, H-6a, H-2b, H-6b), 3.79 (1H, ddd, $J_{4a,5a}$ 10.0 Hz, $J_{5a,6a}$ 5.6 Hz, $J_{5a,6a'}$ 1.7 Hz, H-5a), 3.85 (1H, dd, $J_{3a,4a}$ 9.7 Hz, $J_{2a,3a}$ 3.4 Hz, H-3a), 3.90 (1H, dd, $J_{6b,6b'}$ 12.3 Hz, $J_{5b,6b'}$ 1.7 Hz, H-6b'), 3.94 (1H, dd, $J_{2a,3a}$ 3.4 Hz, $J_{1a,2a}$ 2.0 Hz, H-2a), 4.09 (1H, dd, $J_{6a,6a'}$ 11.4 Hz, $J_{5a,6a'}$ 1.7 Hz, H-6a'), 4.51 (1H, d, $J_{1b,2b}$ 8.5 Hz, H-1b), 5.92 (1H, d, $J_{1a,2a}$ 2.0 Hz, H-1a); δ_C (150 MHz, D_2O) 20.3 (q, OCO*C*H_3), 22.2 (q, NHCO*C*H_3), 55.5 (d, C-2b) 66.2 (d, C-4a), 60.8 (t, C-6b), 68.7 (t, C-6a), 69.0 (d, C-2a), 70.0, 70.2 (2 × d, C-3a, C-4b), 73.8 (2 × d, C-3b, C-5a), 75.9 (d, C-5b), 93.6 (d, C-1a), 101.6 (d, C-1b), 172.2 (s, O*C*OCH_3), 174.7 (s, NH*C*OCH_3). HRMS (ESI) m/z: $[M + Na]^+$ Calcd. For $C_{16}H_{27}NO_{12}Na$ 448.1425; Found 448.1431.

2-Acetamido-2-deoxy-β-D-glucopyranosyl-(1→6)-α-D-mannopyranosyl fluoride 4f

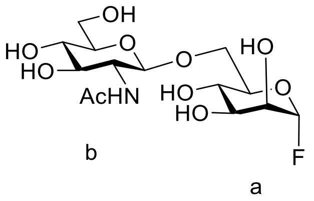

General Procedure B with *N*-acetyl-D-glucosamine **2a** (24 mg, 0.11 mmol), triethylamine (0.14 mL, 0.99 mmol), DMC (54 mg, 0.33 mmol) in water (0.6 mL), and then MeCN (2.4 mL), DMF (0.24 mL), α-D-mannopyranosyl fuoride **3f** (98 mg, 0.54 mmol), powdered molecular sieves (800 mg), and TsOH (19 mg, 0.11 mmol) at rt. Purification by flash column chromatography ($CHCl_3$:MeOH, 6:1 until the excess **3f** had been eluted, then $CHCl_3$:MeOH, 3:1) gave 2-acetamido-2-deoxy-β-D-glucopyranosyl-(1→6)-α-D-mannopyranosyl fluoride **4f** (14 mg, 33%) as a colorless oil; $[\alpha]_D^{22}$ -2 (*c*, 0.3 in H_2O); ν_{max} (neat) 3277 (s, OH), 1639, 1560 (2 × s, amide) cm^{-1}; δ_H (400 MHz, D_2O) 2.01 (3H, s, NHCOCH_3), 3.37-3.46 (2H, m, H-4b, H-5b), 3.48-3.55 (1H, m, H-3b), 3.60-3.67 (1H, m, H-4a), 3.67-3.75 (2H, m, H-2b, H-6b), 3.78-3.86 (3H, m, H-3a, H-5a, H-6a), 3.89 (1H, ad, *J* 12.2 Hz, H-6b'), 4.05-4.07 (1H, m, H-2a), 4.13 (1H, ad, *J* 10.8 Hz, H-6a'), 4.57 (1H, d, *J* 8.4 Hz, H-1b), 5.57 (1H, dd, $J_{H,F}$ 49.2 Hz, $J_{1a,2a}$ 1.4 Hz); δ_H (100 MHz, D_2O) 22.1 (q, NHCOCH_3), 55.5 (d, H-2b), 60.7 (t, C-6b), 65.9 (d, C-4a), 68.4 (dd, $J_{C,F}$ 36.4 Hz, C-2a), 68.9 (t, C-6a), 69.7, 69.8, 75.8 (3 × d, C-3a, C-4b, C-5b), 73.7 (d, C-3b), 74.1 (dd, $J_{C,F}$ 2.5 Hz, C-5a), 101.9 (d, C-1b), 107.7 (dd, $J_{C,F}$ 221 Hz, C-1a), 174.8 (s, C=O). HRMS (ESI) m/z: $[M + Na]^+$ Calcd. For $C_{14}H_{24}FNO_{10}Na$ 408.1276; Found 408.1273.

***p*-Nitrophenyl 2-acetamido-2-deoxy-β-D-glucopyranosyl-(1→6)-β-D-galactopyranoside 4g**[11-12]

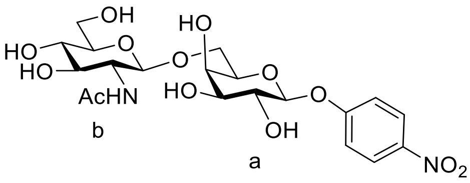

Method 1: General Procedure B with *N*-acetyl-D-glucosamine **2a** (40 mg, 0.18 mmol), triethylamine (0.22 mL, 1.59 mmol), DMC (88mg, 0.54 mmol) in water (1 mL), and then MeCN (4 mL), DMF (0.4 mL), *p*-nitrophenyl β-D-galactopyranoside **3g** (270 mg, 0.9 mmol), powdered molecular sieves (1.6 g), and TsOH (31 mg, 0.18 mmol) at rt. Purification by flash column chromatography ($CHCl_3$:MeOH, 6:1 until the excess **3g** had been eluted, then $CHCl_3$:MeOH,

3:1) gave *p*-nitrophenyl 2-acetamido-2-deoxy-β-D-glucopyranosyl-(1→6)-β-D-galactopyranoside **4g** (29 mg, 32%) as a white solid m.p. 195-200 °C (MeOH/$CHCl_3$, dec) [lit. 240-243 °C, dec][12]; $[\alpha]_D^{22}$ -85.3 (*c*, 0.3 in H_2O) [lit. $[\alpha]_D^{23}$ -99.4 (*c*, 0.5 in H_2O)][12]; νmax (neat) 3331 (s, OH), 1642, 1555 (2 × s, amide), 1512, 1346 (2 × s, N=O) cm^{-1}; δ_H (600 MHz, DMSO-d6) 1.64 (3H, s, NHCOCH_3), 3.03-3.12 (2H, m, H-4b, H-5b), 3.25 (1H, at, *J* 9.3 Hz, H-3b), 3.38-3.45 (3H, m, H-3a, H-2b, H-6b) 3.55-3.61 (2H, H-2a, H-6a), 3.64 (1H, ad, *J* 3.2 Hz, H-4a), 3.69 (1H, ad, *J* 11.6 Hz, H-6b'), 3.79-3.86 (2H, m, H-5a, H-6a'), 4.30 (1H, d, $J_{1b,2b}$ 8.4 Hz, H-1b), 4.95 (1H, d, $J_{1a,2a}$ 7.7 Hz, H-1a), 7.22 (2H, dd, *J* 9.2 Hz, 1.9 Hz, Ar-H), 8.23 (2H, dd, *J* 9.2 Hz, 1.9 Hz, Ar-H); δ_C (150 MHz, DMSO-d6)[11] 23.1 (q, NHCOCH_3), 55.6 (d, C-2b), 61.3 (t, C-6b), 68.6 (d, C-4a), 68.7 (t, C-6a), 70.1 (d, C-2a), 70.9, 77.1 (2 × d, C-4b, C-5b), 73.0 (d, C-3a), 73.9 (d, C-5a), 74.4 (d, C-3b), 100.8 (d, C-1a), 101.4 (d, C-1b), 116.9, 126.2 (2 × d, Ar-C), 141.9, 162.9 (2 × s, Ar-C), 169.6 (s, C=O). HRMS (ESI) m/z: $[M + Na]^+$ Calcd. For $C_{20}H_{28}N_2O_{13}Na$ 527.1484; Found 527.1484.

Method 2: General Procedure B with *N*-acetyl-D-glucosamine **2a** (20 mg, 0.09 mmol), triethylamine (0.11 mL, 0.80 mmol), DMC (44 mg, 0.27 mmol) in water (0.5 mL), and then MeCN (2 mL), DMF (0.2 mL), *p*-nitrophenyl β-D-galactopyranoside **3g** (135 mg, 0.45 mmol), powdered molecular sieves (800 mg), and TsOH (16 mg, 0.09 mmol) at 0 °C. Purification by flash column chromatography ($CHCl_3$:MeOH, 6:1 until the excess **3g** had been eluted, then $CHCl_3$:MeOH, 3:1) gave *p*-nitrophenyl 2-acetamido-2-deoxy-β-D-glucopyranosyl-(1→6)-β-D-galactopyranoside **4g** (20 mg, 44% identical to the material described above).

***p*-Nitrophenyl 2-acetamido-2-deoxy-β-D-glucopyranosyl-(1→6)-2-acetamido-2-deoxy-β-D-glucopyranoside 4h**

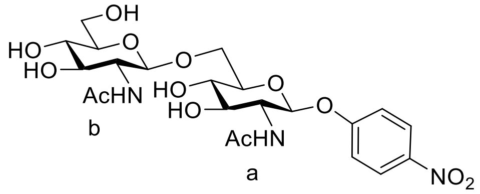

General Procedure B with *N*-acetyl-D-glucosamine **2a** (40 mg, 0.18 mmol), triethylamine (0.22 mL, 1.59 mmol), DMC (88 mg, 0.54 mmol) in water (1 mL), and then DMF (4.4 mL), *p*-nitrophenyl 2-acetamido-2-deoxy-β-D-glucopyranoside **3h** (310 mg, 0.89 mmol), powdered molecular sieves (1.6 g), and TsOH (31 mg, 0.18 mmol) at rt. Purification by flash column

chromatography ($CHCl_3$:MeOH, 6:1 until the excess **3h** had been eluted, then $CHCl_3$:MeOH, 3:1) gave *p*-nitrophenyl 2-acetamido-2-deoxy-β-D-glucopyranosyl-(1→6)-2-acetamido-2-deoxy-β-D-glucopyranoside **4h** (39 mg, 39%) as a white solid, m.p. 183-186 °C (MeOH, dec); $[\alpha]_D^{22}$ -24 (*c*, 0.3 in H_2O); νmax (neat) 3282 (s, OH), 1621, 1554 (2 × s, amide) 1519, 1349 (2 × s, N=O) cm^{-1}; δ_H (600 MHz, D_2O) 1.80 (3H, s, NHCOCH_3), 1.98 (3H, s, NHCOCH_3), 3.39-3.44 (2H, m, H-5b, H-4b), 3.47-3.52 (2H, m, H-4a, H-3b), 3.62-3.72 (4H, m, H-3a, H-2b, H-6b), 3.75 (1H, dd, $J_{6a,6a'}$ 11.3 Hz, $J_{5a,6a}$ 6.0 Hz, H-6a), 3.83 (1H, ddd, $J_{4a,5a}$ 10.1 Hz, $J_{5a,6a}$ 6.0 Hz, $J_{5a,6a'}$ 1.9 Hz, H-5a), 3.88 (1H, d, $J_{6b,6b'}$ 12.4 Hz, $J_{5b,6b'}$ 1.7 Hz, H-6b'), 3.98 (1H, dd, $J_{2a,3a}$ 10.5 Hz, $J_{1a,2a}$ 8.5 Hz, H-2a), 4.23 (1H, dd, $J_{6a,6a'}$ 11.3 Hz, $J_{5a,6a'}$ 1.9 Hz, H-6a'); 4.51 (1H, d, $J_{1b,2b}$ 8.5 Hz, H-1b), 5.29 (1H, dd, $J_{1a,2a}$ 8.5 Hz, H-1a), 7.15 (2H, d, *J* 9.2 Hz, Ar-H), 8.23 (1H, d, *J* 9.2 Hz, Ar-H); δ_C (150 MHz, D_2O) 22.0, 22.1 (2 × q, 2 × NHCOCH_3), 55.2 (d, C-2a), 55.4 (d, C-2b), 60.7 (t, C-6b), 68.4 (t, C-6a), 69.8 (d, C-4a), 69.9, 75.9 (2 × d, C-4b, C-5b), 73.3 (d, C-3a), 73.9 (d, C-3b), 74.9 (d, C-5a), 98.5 (d, C-1a), 101.3 (d, C-1b), 116.5, 126.3 (2 × d, Ar-C), 142.7, 161.7 (2 × s, Ar-C), 174.4, 175.0 (2 × s, 2 × C=O). HRMS (ESI) m/z: $[M + Na]^+$ Calcd. For $C_{22}H_{31}N_3O_{13}Na$ 568.1749; Found 568.1755.

2-Acetamido-2-deoxy-β-D-glucopyranosyl-(1→6)-2-acetamido-2-deoxy-β-D-glucopyranosyl azide 4i[13-14]

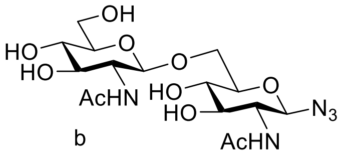

Method 1: General Procedure B with *N*-acetyl-D-glucosamine **2a** (30 mg, 0.14 mmol), triethylamine (0.17 mL, 1.22 mmol), DMC (67 mg, 0.41 mmol) in water (0.75 mL), and then MeCN (3 mL), DMF (0.3 mL), 2-acetamido-2-deoxy-β-D-glucopyranosyl azide **3i** (166 mg, 0.67 mmol), powdered molecular sieves (1.2 g), and TsOH (23 mg, 0.14 mmol) at rt. Purification by flash column chromatography ($CHCl_3$:MeOH, 6:1 until the excess **3i** had been eluted, then $CHCl_3$:MeOH, 3:1) gave 2-acetamido-2-deoxy-β-D-glucopyranosyl-(1→6)-2-acetamido-2-deoxy-β-D-glucopyranosyl azide **4i** (19 mg, 31%) as a white solid m.p. 181-184 °C (MeOH/$CHCl_3$, dec) [lit.218-219 °C][14]; $[\alpha]_D^{22}$ -12 (*c*, 0.2 in H_2O) [lit. $[\alpha]_D^{20}$ -43 (*c* 0.49 in 70% MeOH in H_2O)][14]; νmax (neat) 3355 (s, OH), 2115 (s, N=N=N), 1670, 1636, 1534 (3 × s, amide) cm^{-1}; δ_H (400 MHz, D_2O)[13] 2.02 (3H, s, NHCOCH_3), 2.03 (3H, s, NHCOCH_3), 3.38-3.47 (3H, m, H-4a, H-4b, H-5b), 3.50-3.57

(2H, m, H-3a, H-3b), 3.59-3.80 (5H, m, H-2a, H-5a, H-6a, H-2b, H-6b), 3.90 (1H, dd, $J_{6b,6b'}$ 12.3 Hz, $J_{5b,6b'}$ 1.7 Hz, H-6b'), 4.17 (1H, dd, $J_{6a,6a'}$ 11.6 Hz, $J_{5a,6a'}$ 1.9 Hz, H-6a'), 4.53 (1H, d, $J_{1b,2b}$ 8.4 Hz, H-1b), 4.73 (1H, d, $J_{1a,2a}$ 9.2 Hz, H-1a); δ_C (150 MHz, D_2O) 22.1, 22.2 (2 × q, 2 × NHCOCH_3), 55.0 (d, C-2a), 55.5 (d, C-2b), 60.7 (t, C-6b), 68.4 (t, C-6a), 69.5, 69.9 (2 × d, C-4a, C-4b), 73.6, 73.7 (2 × d, C-3a, C-3b), 75.8 (d, C-5b), 76.7 (d, C-5a), 88.7 (d, C-1a), 101.7 (d, C-1b), 174.7, 174.8 (2 × s, 2 × C=O). HRMS (ESI) m/z: $[M + Na]^+$ Calcd. For $C_{16}H_{27}N_5O_{10}Na$ 472.1650; Found 472.1646.

Method 2: General Procedure B with *N*-acetyl-D-glucosamine **2a** (66 mg, 0.3 mmol), triethylamine (0.37 mL, 2.69 mmol), DMC (147 mg, 0.9 mmol) in water (1.65 mL), and then MeCN (6.6 mL), DMF (0.66 mL), 2-acetamido-2-deoxy-β-D-glucopyranosyl azide **3i** (366 mg, 1.49 mmol), powdered molecular sieves (2.6 g), and TsOH (53 mg, 0.29 mmol) at -16 °C for 24 h. Purification by flash column chromatography ($CHCl_3$:MeOH, 6:1 until the excess **3i** had been eluted, then $CHCl_3$:MeOH, 3:1) gave 2-acetamido-2-deoxy-β-D-glucopyranosyl-(1→6)-2-acetamido-2-deoxy-β-D-glucopyranosyl azide **4i** (49 mg, 37% identical to the material described above).

***t*-Butyl 2-acetamido-2-deoxy-β-D-glucopyranosyl-(1→6)-2-acetamido-2-deoxy-β-D-glucopyranoside 4j**

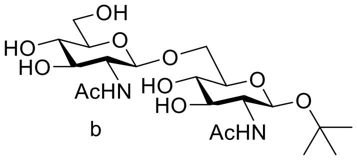

Method 1: General Procedure B with *N*-acetyl-D-glucosamine **2a** (30 mg, 0.14 mmol), triethylamine (0.17 mL, 1.22 mmol), DMC (67 mg, 0.41 mmol) in water (0.75 mL), and then MeCN (3 mL), DMF (0.3 mL) *t*-butyl 2-acetamido-2-deoxy-β-D-glucopyranoside **5d** (188 mg, 0.68 mmol), powdered molecular sieves (1.2 g), and TsOH (24 mg, 0.14 mmol) at rt. Purification by flash column chromatography ($CHCl_3$:MeOH, 5:1 until the excess **5d** had been eluted, then $CHCl_3$:MeOH, 3:1) gave *t*-butyl 2-acetamido-2-deoxy-β-D-glucopyranosyl-(1→6)-2-acetamido-2-deoxy-β-D-glucopyranoside **4j** (26 mg, 39%) as a white solid, m.p. 176-185 °C (MeOH/$CHCl_3$, dec); $[\alpha]_D^{22}$ -17 (*c*, 0.4 in H_2O); ν_{max} (neat) 3300 (s, OH), 1640, 1563 (2 × s, amide) cm^{-1}; δ_H (600 MHz, D_2O) 1.19 (9H, s, 3 × CH_3), 2.02 (3H, s, NHCOCH_3), 2.03 (3H, s, NHCOCH_3), 3.33 (1H, at, *J* 8.1 Hz, H-4a), 3.41-3.48 (2H, m, H-4b, H-5b), 3.50-3.60 (4H, m, H-2a, H-3a, H-5a, H-3b), 3.64 (1H, m, H-6a), 3.68-3.78 (2H, m, H-2b, H-6b), 3.92 (1H, ad, *J* 12.2 Hz, H-6b'), 4.19 (1H, ad, *J* 11.0 Hz,

H-6a'), 4.53 (1H, d, $J_{1b,2b}$ 9.9 Hz, H-1b), 4.65 (1H, d, $J_{1a,2a}$ 7.1 Hz, H-1a). δ_C (150 MHz, D_2O) 22.3, 22.4 (2 × q, NHCO*C*H_3), 27.7 (3 × q, CH_3), 55.5 (d, C-2b), 56.0 (d, C-2a), 60.8 (t, C-6b), 68.7 (t, C-6a), 70.0 (d, C-4b), 70.2 (d, C-4a), 73.8, 74.0 (2 × d, C-3a, C-5a), 74.3 (d, C-3b), 75.9 (d, C-5b), 77.3 (s, *C*CH_3), 95.6 (d, C-1a), 101.1 (d, C-1b), 174.5, 174.6 (2 × s, 2 × C=O). HRMS (ESI) m/z: [M + Na]$^+$ Calcd. For $C_{20}H_{36}N_2O_{11}Na$ 503.2211; Found 503.2214.

Method 2: General Procedure B with *N*-acetyl-D-glucosamine **2a** (14 mg, 0.06 mmol), triethylamine (0.08 mL, 0.57 mmol), DMC (31 mg, 0.19 mmol) in water (0.35 mL), and then MeCN (1.4 mL), DMF (0.14 mL), *t*-butyl 2-acetamido-2-deoxy-β-D-glucopyranoside **5d** (85 mg, 0.31 mmol), powdered molecular sieves (800 mg), and TsOH (11 mg, 0.06 mmol) at 0 °C. Purification by flash column chromatography ($CHCl_3$:MeOH, 5:1 until the excess **5d** had all been eluted, and then $CHCl_3$:MeOH, 3:1) gave *t*-butyl 2-acetamido-2-deoxy-β-D-glucopyranosyl-(1→6)-2-acetamido-2-deoxy-β-D-glucopyranoside **4j** (8 mg, 26% identical to the material described above).

Phenyl 2-acetamido-2-deoxy-β-D-glucopyranosyl-(1→6)-β-D-glucopyranoside 4k

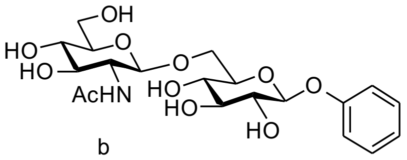

General Procedure B with *N*-acetyl-D-glucosamine **2a** (40 mg, 0.18 mmol), triethylamine (0.22 mL, 1.59 mmol), DMC (88 mg, 0.54 mmol) in water (1 mL), and then MeCN (4 mL), DMF (0.4 mL), phenyl β-D-glucopyranoside **3k** (231 mg, 0.9 mmol), powdered molecular sieves (1.6 g), and TsOH (31 mg, 0.18 mmol) at rt. Purification by flash column chromatography ($CHCl_3$:MeOH, 6:1 until the excess **3k** had been eluted, then $CHCl_3$:MeOH, 3:1) gave a mixture of two isomers (0.8 mg, 1%) and phenyl 2-acetamido-2-deoxy-β-D-glucopyranosyl-(1→6)-β-D-glucopyranoside **4k** (37 mg, 45%) as a white solid m.p. 208-215 °C (MeOH, dec); $[\alpha]_D^{22}$ -51 (*c*, 0.2 in H_2O); ν_{max} (neat) 3268 (s, OH), 1635, 1557 (2 × s, amide) cm^{-1}; δ_H (400 MHz, D_2O) 1.81 (3H, s, NHCOC*H*$_3$), 3.33-3.60 (6H, m, H-2a, H-3a, H-4a, H-3b, H-4b, H-5b), 3.63-3.76 (4H, m, H-5a, H-6a, H-2b, H-6b), 3.86 (1H, dd, $J_{6b,6b'}$ 12.2 Hz, $J_{5b,6b'}$ 2.0 Hz, H-6b'), 4.18 (1H, ad, J 9.6 Hz, H-6a'), 4.47 (1H, d, $J_{1b,2b}$ 8.5 Hz, H-1b), 5.09 (1H, d, $J_{1a,2a}$ 7.5 Hz, H-1a), 7.06-7.15 (3H, m, Ar-H), 7.34-7.40 (2H, m, Ar-H). δ_C (150 MHz, D_2O) 22.1 (q, NHCO*C*H_3), 55.4 (d, C-2b), 60.7 (t, C-6b), 68.3 (t, C-6a), 74.8 (d, C-5a), 69.5, 69.9, 72.8, 73.9, 75.5, 75.9 (6 × d, C-2a, C-3a, C-4a, C-3b, C-4b, C-5b), 100.0 (d, C-

1a), 101.2 (d, C-1b), 116.5, 123.4, 130.1 (3 × d, Ar-C), 156.6 (s, Ar-C), 174.5 (s, C=O). HRMS (ESI) m/z: $[M + Na]^+$ Calcd. For $C_{20}H_{29}NO_{11}$ 482.1633; Found 482.1636.

Phenyl 2-acetamido-2-deoxy-β-D-glucopyranosyl-(1→6)-β-D-thioglucopyranoside 4l

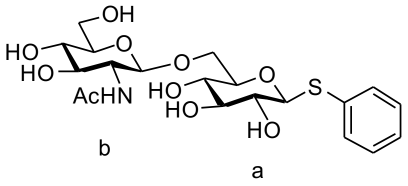

General Procedure B with *N*-acetyl-D-glucosamine **2a** (40 mg, 0.18 mmol), triethylamine (0.22 mL, 1.59 mmol), DMC (88 mg, 0.54 mmol) in water (1 mL), and then MeCN (4 mL), DMF (0.4 mL), phenyl β-D-thioglucopyranoside **3l** (246 mg, 0.9 mmol), powdered molecular sieves (1.6 g), and TsOH (31 mg, 0.18 mmol) at rt. Purification by flash column chromatography ($CHCl_3$:MeOH, 6:1 until the excess **3l** had been eluted, then $CHCl_3$:MeOH, 3:1) gave a mixture of two isomers (11 mg, 13%) and phenyl 2-acetamido-2-deoxy-β-D-glucopyranosyl-(1→6)-β-D-thioglucopyranoside **4l** (29 mg, 34%) as a colorless oil; $[\alpha]_D^{22}$ -52 (*c*, 0.1 in 50% MeOH in H_2O); ν_{max} (neat) 3314 (s, OH), 1645, 1557 (2 × s, amide) cm^{-1}; δ_H (600 MHz, D_2O) 1.93 (3H, s, NHCOCH_3), 3.31 (1H, at, *J* 9.4 Hz, H-2a), 3.34-3.45 (3H, m, H-3a, H-4a, H-5b), 3.46-3.51 (2H, m, H-3b, H-4b), 3.58 (1H, $J_{4a,5a}$ 10.0 Hz, $J_{5a,6a}$ 5.4 Hz, $J_{5a,6a'}$ 1.9 Hz, H-5a), 3.67-3.75 (3H, m, H-6a, H-2b, H-6b), 3.88 (1H, dd, $J_{6b,6b'}$ 12.3 Hz, $J_{5b,6b'}$ 1.9 Hz, H-6b'), 4.12 (1H, dd, $J_{6a,6a'}$ 11.6 Hz, $J_{5a,6a'}$ 1.9 Hz, H-6a'), 4.49 (1H, d, $J_{1b,2b}$ 8.5 Hz, H-1b), 4.77 (1H, under water peak, H-1a), 7.34-7.43 (3H, m, Ar-H), 7.51-7.54 (2H, m, Ar-H). δ_C (150 MHz, D_2O) 22.3 (q, NHCOCH$_3$), 55.5 (d, C-2b), 60.7 (t, C-6b), 68.5 (t, C-6a), 71.7 (d, C-2a), 69.3, 69.9, 73.9, 75.9, 77.2 (5 × d, C-3a, C-4a, C-3b, C-4b, C-5b), 78.6 (d, C-5a), 87.5 (d, C-1a), 101.4 (d, C-1b), 128.1, 129.5, 131.4 (3 × d, Ar-C), 132.3 (s, Ar-C), 174.6 (s, C=O). HRMS (ESI) m/z: $[M + Na]^+$ Calcd. For $C_{20}H_{29}NO_{10}S$ 498.1404; Found 498.1411.

Method 2: General Procedure B with *N*-acetyl-D-glucosamine **2a** (30 mg, 0.14 mmol), triethylamine (0.17 mL, 1.22 mmol), DMC (67 mg, 0.41 mmol) in water (0.75 mL), and then MeCN (3 mL), DMF (0.3 mL), phenyl β-D-thioglucopyranoside **3l** (185 mg, 0.68 mmol), powdered molecular sieves (1.2 g), and TsOH (24 mg, 0.14 mmol) at 0 °C. Purification by flash column chromatography ($CHCl_3$:MeOH, 6:1 until the excess **3l** had been eluted, then $CHCl_3$:MeOH, 3:1 gave a mixture of two isomers (12 mg, 19%) and phenyl 2-acetamido-2-deoxy-β-D-

glucopyranosyl-(1→6)-β-D-thioglucopyranoside **4l** (25 mg, 39% identical to the material described above).

Methyl 2-acetamido-2-deoxy-β-D-glucopyranosyl-(1→6)-β-D-glucopyranoside 4m

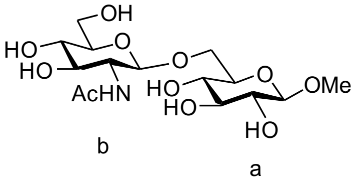

General Procedure B with *N*-acetyl-D-glucosamine **2a** (40 mg, 0.18 mmol), triethylamine (0.22 mL, 1.59 mmol), DMC (88 mg, 0.54 mmol) in water (1 mL), and then MeCN (4 mL), DMF (0.4 mL), methyl β-D-glucopyranoside **3m** (175 mg, 0.9 mmol), powdered molecular sieves (1.6 g), and TsOH (31 mg, 0.18 mmol) at rt. Purification by flash column chromatography ($CHCl_3$:MeOH, 5:1 until the excess **3m** had been eluted, then $CHCl_3$:MeOH, 3:1) gave a mixture of two isomers (24 mg, 33%) and methyl 2-acetamido-2-deoxy-β-D-glucopyranosyl-(1→6)-β-D-glucopyranoside **4m** (13 mg, 18%) as a colorless oil; $[\alpha]_D^{22}$ -2 (*c*, 0.1 in H_2O); ν_{max} (neat) 3275 (s, OH), 1635, 1553 (2 × s, amide) cm^{-1}; δ_H (400 MHz, D_2O) 2.00 (3H, s, NHCOCH_3), 3.18 (1H, at, *J* 8.7 Hz, H-2a), 3.26-3.55 (9H, m, H-3a, H-4a, H-5a, H-3b, H-4b, H-5b, OCH_3), 3.62-3.78 (4H, m, H-6a, H-2b, H-6b), 3.88 (1H, ad, *J* 12.4 Hz, H-6b'), 4.16 (1H, ad, *J* 11.1 Hz, H-6a'), 4.31 (1H, d, $J_{1a,2a}$ 8.0 Hz, H-1a), 4.48 (1H, d, $J_{1b,2b}$ 8.5 Hz, H-1b). δ_C (100 MHz, D_2O) 55.4, 69.6, 69.9, 73.0, 73.7, 74.5, 75.7, 75.8 (8 × d, C-3a, C-4a, C-5a, C-3b, C-4b, C-5b), 57.0 (q, OCH_3), 60.6 (t, C-6b), 68.4 (t, C-6a), 101.5 (d, C-1b), 103.2 (d, C-1a), 174.5 (s, C=O). HRMS (ESI) m/z: $[M + Na]^+$ Calcd. For $C_{15}H_{27}NO_{11}$ 420.1476; Found 420.1477.

***p*-Nitrophenyl 2-acetamido-2-deoxy-β-D-galactopyranosyl-(1→6)-β-D-glucopyranoside 4n**

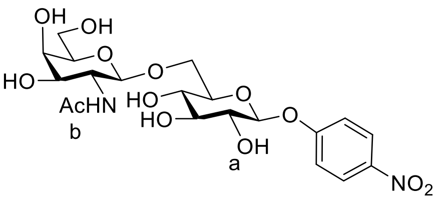

General Procedure B with *N*-acetyl-D-galactosamine **2b** (40 mg, 0.18 mmol), triethylamine (0.22 mL, 1.59 mmol), DMC (88 mg, 0.54 mmol) in water (1 mL), and then MeCN (4 mL), DMF (0.4 mL), *p*-nitrophenyl β-D-glucopyranoside **3a** (270 mg, 0.89 mmol), powdered molecular

sieves (1.6 g), and TsOH (31 mg, 0.18 mmol) at rt. Purification by flash column chromatography ($CHCl_3$:MeOH, 6:1 until the excess **3a** had been eluted, then $CHCl_3$:MeOH, 3:1) gave *p*-nitrophenyl 2-acetamido-2-deoxy-β-D-galactopyranosyl-(1→6)-β-D-glucopyranoside **4n** (37 mg, 40%) as a white solid, m.p. 220-228 °C (MeOH, dec); $[\alpha]_D^{22}$ -59 (*c*, 0.2 in H_2O); ν_{max} (neat) 3320 (s, OH), 1654, 1559 (2 × s, amide) 1512, 1353 (2 × s, N=O) cm^{-1}; δ_H (600 MHz, DMSO-d6) 1.66 (3H, s, NHCOCH_3), 3.07 (1H, at, *J* 9.2 Hz, H-4a), 3.26-3.29 (2H, m, H-2a, H-3a), 3.32 (1H, at, *J* 6.3 Hz, H-5b), 3.41 (1H, dd, $J_{2b, 3b}$ 10.6 Hz, $J_{3b,4b}$ 3.2 Hz, H-3b), 3.48 (1H, dd, $J_{6a,6a'}$ 11.5 Hz, $J_{5a,6a}$ 7.7 Hz, H-6a), 3.50-3.54 (2H, m, H-6b, H-6b'), 3.65 (1H, ad, *J* 3.2 Hz, H-4b), 3.68 (1H, ddd, $J_{4a,5a}$ 9.8 Hz, $J_{5a,6a}$ 7.7 Hz, $J_{5a,6a'}$ 1.9 Hz, H-5a), 3.72 (1H, dd, $J_{2b,3b}$ 10.6 Hz, $J_{1b,2b}$ 8.4 Hz, H-2b), 3.96 (1H, dd, $J_{6a,6a'}$ 11.5 Hz, $J_{5a,6a'}$ 1.9 Hz, H-6a'), 4.43 (1H, d, $J_{1b,2b}$ 8.5 Hz, H-1b), 5.17 (1H, d, $J_{1a,2a}$ 7.6 Hz, H-1a), 7.21 (2H, d, *J* 9.3 Hz, Ar-H), 8.25 (2H, d, *J* 9.3 Hz, Ar-H); δ_C (150 MHz, DMSO-d6) 23.1 (q, NHCO*C*H_3), 52.4 (d, C-2b), 60.7 (t, C-6b), 67.7 (d, C-4b), 69.6 (t, C-6a), 70.2 (d, C-4a), 71.7 (d, C-3b), 73.1 (d, C-2a), 75.3, 75.4 (2 × d, C-5a, C-5b), 76.4 (d, C-3a), 100.2 (d, C-1a), 102.4 (d, C-1b), 117.0, 126.2 (2 × d, Ar-C), 141.9, 162.7 (2 × s, Ar-C), 170.0 (s, C=O). HRMS (ESI) m/z: $[M + Na]^+$ Calcd. For $C_{20}H_{28}N_2O_{13}Na$ 527.1484; Found 527.1480.

_p_-Nitrophenyl 2-acetamido-2-deoxy-β-D-galactopyranosyl-(1→6)-β-D-galactopyranoside 4o

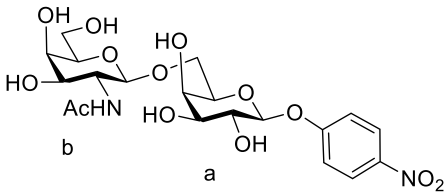

Method 1: General Procedure B with *N*-acetyl-D-galactosamine **2b** (40 mg, 0.18 mmol), triethylamine (0.22 mL, 1.59 mmol), DMC (88 mg, 0.54 mmol) in water (1 mL), and then MeCN (4 mL), DMF (0.4 mL), *p*-nitrophenyl β-D-galactopyranoside **3g** (270 mg, 0.89 mmol), powdered molecular sieves (1.6 g), and TsOH (31 mg, 0.18 mmol) at rt. Purification by flash column chromatography ($CHCl_3$:MeOH, 6:1 until the excess **3g** had been eluted, then $CHCl_3$:MeOH, 3:1) gave *p*-nitrophenyl 2-acetamido-2-deoxy-β-D-galactopyranosyl-(1→6)-β-D-galactopyranoside **4o** (24 mg, 26%) as a white solid, m.p. 192-198 °C (MeOH, dec); $[\alpha]_D^{22}$ -42 (*c*, 0.1 in H_2O); ν_{max} (neat) 3305 (s, OH), 1620, 1572 (2 × s, amide) 1512, 1347 (2 × s, N=O) cm^{-1}; δ_H (400 MHz, D_2O) 1.73 (3H, s, NHCOCH_3), 3.60-3.91 (9H, m, H-2a, H-3a, H-6a, H-2b, H-3b, H-4b, H-5b, H-6b, H-6b'), 3.96 (1H, ad, *J* 3.2 Hz, H-4a), 4.03-4.09 (2H, m, H-5a, H-6a'), 4.43 (1H, d, $J_{1b,2b}$ 8.5 Hz, H-1b),

5.17 (1H, d, $J_{1a,2a}$ 7.6 Hz, H-1a), 7.21 (2H, d, *J* 9.3 Hz, Ar-H), 8.25 (2H, d, *J* 9.3 Hz, Ar-H); δ_C (150 MHz, D_2O) 22.0 (q, NHCOCH_3), 52.4 (d, C-2b), 67.8, 70.2, 71.2, 72.2, 75.2 (5 × d, C-2a, C-3a, C-3b, C-4b, C-5b), 61.0 (t, C-6b), 68.5 (d, C-4a), 68.83 (t, C-6a), 74.0 (d, C-5a), 100.0 (d, C-1a), 101.7 (d, C-1b), 116.3, 126.3 (2 × d, Ar-C), 142.5, 161.9 (2 × s, Ar-C), 174.6 (s, C=O). HRMS (ESI) m/z: $[M + Na]^+$ Calcd. For $C_{20}H_{28}N_2O_{13}Na$ 527.1484; Found 527.1483.

Method 2: General Procedure B with *N*-acetyl-D-galactosamine **2b** (40 mg, 0.18 mmol), triethylamine (0.22 mL, 1.59 mmol), DMC (88 mg, 0.54 mmol) in water (1 mL), and then MeCN (4 mL), DMF (0.4 mL), *p*-nitrophenyl β-D-galactopyranoside **3g** (270 mg, 0.89 mmol), powdered molecular sieves (1.6 g), and TsOH (31 mg, 0.18 mmol) at -10 °C. Purification by flash column chromatography ($CHCl_3$:MeOH, 6:1 until the excess **3g** had been eluted, then $CHCl_3$:MeOH, 3:1) gave *p*-nitrophenyl 2-acetamido-2-deoxy-β-D-galactopyranosyl-(1→6)-β-D-galactopyranoside **4o** (27 mg, 30% identical to the material described above).

***p*-Nitrophenyl 2-acetamido-2-deoxy-α-D-mannopyranosyl-(1→6)-β-D-glucopyranoside 4p**

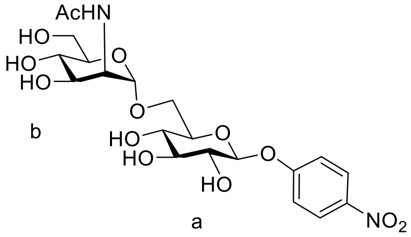

General Procedure B with *N*-acetyl-D-mannosamine **2c** (40 mg, 0.18 mmol), triethylamine (0.22 mL, 1.59 mmol), DMC (88 mg, 0.54 mmol) in water (1 mL), and then MeCN (4 mL), DMF (0.4 mL), *p*-nitrophenyl β-D-glucopyranoside **3a** (270 mg, 0.89 mmol), powdered molecular sieves (1.6 g), and TsOH (31 mg, 0.18 mmol) at rt. Purification by flash column chromatography ($CHCl_3$:MeOH, 6:1) gave *p*-nitrophenyl 2-acetamido-2-deoxy-α-D-mannopyranosyl-(1→6)-β-D-glucopyranoside **4p** (14 mg, 15%) as a colorless solid, m.p. 166-172 °C (MeOH, dec); $[\alpha]_D^{22}$ -43.3 (*c*, 0.3 in H_2O); νmax (neat) 3277 (s, OH), 1650, 1555 (2 × s, amide), 1513, 1344 (2 × s, N=O) cm^{-1}; δ_H (400 MHz, CD_3OD) 2.00 (3H, s, NHCOCH_3), 3.39 (1H, at, *J* 9.3 Hz, H-4a), 3.47-3.51 (2H, m, H-2a, H-3a), 3.54-3.58 (2H, m, H-4b, H-5b), 3.68-3.74 (2H, m, H-5a, H-6b, H-6b'), 3.81-3.84 (2H, ad, *J* 3.9 Hz, H-6a, H-6a'), 3.90-3.96 (1H, dd, $J_{2b,3b}$ 4.8 Hz, $J_{3b,4b}$ 9.1 Hz, H-3b), 4.34 (1H, dd, $J_{1b,2b}$ 1.6 Hz, $J_{2b,3b}$ 4.8 Hz, H-2b), 4.75 (1H, d, $J_{1b,2b}$ 1.6 Hz, H-1b), 5.07 (1H, d, $J_{1a,2a}$ 7.3 Hz, H-1a), 7.24 (2H, d, *J* 9.3 Hz, Ar-H), 8.26 (2H, d, *J* 9.3 Hz, Ar-H). δ_C (150 MHz, CD_3OD) 22.6 (q, NHCOCH_3), 54.3 (d, C-2b), 62.1 (2 × t, C-6b), 67.4 (2 × t, C-6a), 68.1 (d, C-4b), 70.8 (d, C-3b), 71.3 (d, C-4a), 74.0

(C-5b), 74.7 (d, C-3a), 77.9 (d, C-2a), 100.2 (d, C-1b), 101.5 (d, C-2b), 117.7, 126.8 (2 × d, Ar-C), 144.0, 163.8 (2 × s, Ar-C), 174.0 (s, C=O). HRMS (ESI) m/z: $[M + Na]^+$ Calcd. For $C_{20}H_{28}N_2O_{13}Na$ 527.1484; Found 527.1477.

Charaterisation of β (1→3)-linked disaccharides in the cases where *p*NP-galactoside 3g was used as the acceptor

***p*-Nitrophenyl 2-acetamido-2-deoxy-β-D-glucopyranosyl-(1→3)-β-D-galactopyranoside[11, 15]**

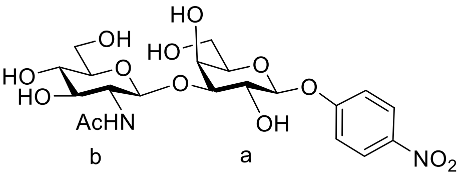

General Procedure B with *N*-acetyl-D-glucosamine **2a** (40 mg, 0.18 mmol), triethylamine (0.22 mL, 1.59 mmol), DMC (88 mg, 0.54 mmol) in water (1 mL)and then MeCN (4 mL), DMF (0.4 mL), *p*-nitrophenyl β-D-galactopyranoside **3g** (270 mg, 0.89 mmol), powdered molecular sieves (1.6 g), TsOH (31 mg, 0.18 mmol) at rt. Purification by flash column chromatography gave *p*-nitrophenyl 2-acetamido-2-deoxy-β-D-glucopyranosyl-(1→6)-β-D-galactopyranoside **4g** (29 mg, 32%) identical to the material described above, and *p*-nitrophenyl 2-acetamido-2-deoxy-β-D-glucopyranosyl-(1→3)-β-D-galactopyranoside (13 mg, 14%) as a white solid m.p. 230-235 °C (MeOH, dec) [lit 272 °C, dec][15]; $[\alpha]_D^{22}$ -28 (*c*, 0.1 in H_2O) [lit. $[\alpha]_D$ -36.6 (*c*, 0.4 in H_2O)][15]; νmax (neat) 3321 (s, OH), 1642, 1555 (2 × s, amide), 1516, 1346 (s, N=O) cm^{-1}; δ_H (600 MHz, D_2O) 2.01 (3H, s, NHCOCH_3), 3.40-3.47 (2H, m, H-4b, H-5b), 3.54 (1H, dd, $J_{3b,4b}$ 10.3 Hz, $J_{2b,3b}$ 7.9 Hz, H-3b), 3.71-3.77 (4H, m, H-6a, H-2b, H-6b, H-6b'), 3.83 (1H, dd, $J_{2a,3a}$ 9.8 Hz, $J_{3a,4a}$ 3.2 Hz, H-3a), 3.85-3.91 (3H, m, H-2a, H-5a, H-6a'), 4.21 (1H, ad, J 3.1 Hz, H-4a), 4.7 (1H, d, $J_{1b,2b}$ 8.5 Hz, H-1b), 5.16 (1H, d, $J_{1a,2a}$ 7.5 Hz, H-1a), 7.21 (2H, d, J 9.3 Hz, Ar-H), 8.23 (2H, d, J 9.3 Hz, Ar-H); δ_C (150 MHz, D_2O)[11] 22.2 (q, NHCOCH_3), 55.7 (d, C-2b), 60.5, 60.6 (2 × t, C-6a, C-6b), 68.2 (d, C-4a), 69.5 (d, C-2a), 69.7, 75.7 (2 × d, C-4b, C-5b), 73.6 (d, C-3b), 75.2 (d, C-5a), 81.7 (d, C-3a), 100.1 (d, C-1a), 102.9 (d, C-1b), 116.5, 126.1 (2 × d, Ar-C), 142.6, 161.9 (2 × s, Ar-C), 175.0 (s, C=O). HRMS (ESI) m/z: $[M + Na]^+$ Calcd. For $C_{20}H_{28}N_2O_{13}Na$ 527.1484; Found 527.1470.

_p_-Nitrophenyl 2-acetamido-2-deoxy-β-D-galactopyranosyl-(1→3)-β-D-galactopyranoside

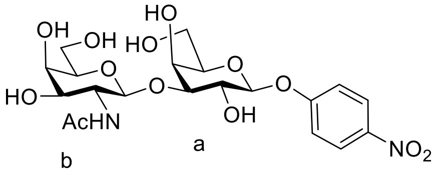

General Procedure B with *N*-acetyl-D-galactosamine **2b** (40 mg, 0.18 mmol), triethylamine (0.22 mL, 1.59 mmol), DMC (88 mg, 0.54 mmol) in water (1 mL), then MeCN (4 mL), DMF (0.4 mL), *p*-nitrophenyl β-D-galactopyranoside **3g** (270 mg, 0.89 mmol), powdered molecular sieves (1.6 g), and TsOH (31 mg, 0.18 mmol) at rt. Purification by flash column chromatography (gradient elution $CHCl_3$:MeOH, 6:1 to 3:1) gave *p*-nitrophenyl 2-acetamido-2-deoxy-β-D-galactopyranosyl-(1→6)-β-D-galactopyranoside **4o** (24 mg, 26% identical to the material described above) and *p*-nitrophenyl 2-acetamido-2-deoxy-β-D-galactopyranosyl-(1→3)-β-D-galactopyranoside (13 mg, 14%) as a white solid m.p. 241-245 °C (MeOH); $[\alpha]_D^{22}$ -14 (*c*, 0.1 in H_2O); νmax (neat) 3331 (s, OH), 1642, 1555 (2 × s, amide), 1516, 1345 (s, N=O) cm^{-1}; δ_H (600 MHz, D_2O) 2.01 (3H, s, NHCOCH_3), 3.65 (1H, dd, $J_{2b,3b}$ 7.9 Hz, $J_{3b,4b}$ 4.4 Hz, H-3b), 3.70-3.80 (6H, m, H-6a, H-6a', H-4b, H-6b, H-6b'), 3.84 (1H, dd, $J_{2a,3a}$ 9.8 Hz, $J_{3a,4a}$ 3.1 Hz, H-3a), 3.85-3.91 (3H, m, H-2a, H-5a, H-5b), 3.93 (1H, dd, $J_{2b,3b}$ 10.9 Hz, $J_{1b,2b}$ 8.5 Hz, H-2b), 4.21 (1H, ad, *J* 3.1 Hz, H-4a), 4.63 (1H, d, $J_{1b,2b}$ 8.5 Hz, H-1b), 5.16 (1H, d, $J_{1a,2a}$ 7.4 Hz, H-1a), 7.21 (2H, d, *J* 9.3 Hz, Ar-H), 8.23 (2H, d, *J* 9.3 Hz, Ar-H); δ_C (150 MHz, D_2O) 22.2 (q, NHCO*C*H_3), 52.6 (d, C-2b), 60.6, 61.0 (2 × t, C-6a, C-6b), 68.3 (d, C-4a), 67.8, 69.6, 75.2 (3 × d, C-2a, C-5a, C-5b), 70.7 (C-4b) 75.0 (d, C-3b), 81.5 (d, C-3a), 100.0 (d, C-1a), 103,4 (d, C-1b), 116.5, 126.1 (2 × d, Ar-C), 142.5, 161.9 (2 × s, Ar-C), 175.2 (s, C=O). HRMS (ESI) m/z: $[M + Na]^+$ Calcd. For $C_{20}H_{28}N_2O_{13}Na$ 527.1484; Found 527.1479.

p-Nitrophenyl 2-acetamido-2-deoxy-β-D-glucopyranosyl-(1→6)[-α-D-glucopyranosyl-(1→4)]-β-D-glucopyranoside T1 and p-nitrophenyl 2-acetamido-2-deoxy-β-D-glucopyranosyl-(1→6)-α-D-glucopyranosyl-(1→4)-β-D-glucopyranoside T2

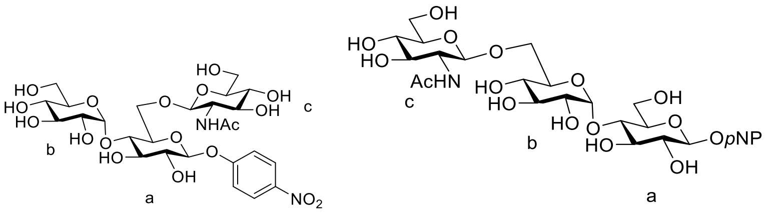

General Procedure B with *N*-acetyl-D-glucosamine **2a** (40 mg, 0.18 mmol), triethylamine (0.22 mL, 1.59 mmol), DMC (88 mg, 0.54 mmol) in water (1 mL), and then MeCN (4 mL), DMF (0.4 mL), p-nitrophenyl β-D-maltoside (419 mg, 0.90 mmol), powdered molecular sieves (1.6 g), and TsOH (31 mg, 0.18 mmol) at rt. Purification by flash column chromatography ($CHCl_3$:MeOH, 3:1) gave the product as a mixture of two regioisomers (58 mg, 48%). Further purification by Semi-Prep HPLC (column: Phenomenex Luna 5U C18 100 Å (250 × 10 mm × 10 µm); eluent: linear gradient of MeCN using a isocratic method, 11% MeCN; flow rate: 2.5 $mLmin^{-1}$ over 25 min; detection: UV 280 nm) gave p-nitrophenyl 2-acetamido-2-deoxy-β-D-glucopyranosyl-(1→6)[-α-D-glucopyranosyl-(1→4)]-β-D-glucopyranoside **T1** (35 mg, 29%) as a colorless syrup and p-nitrophenyl 2-acetamido-2-deoxy-β-D-glucopyranosyl-(1→6)-α-D-glucopyranosyl-(1→4)-β-D-glucopyranoside **T2** (18 mg, 15%) as a colorless syrup.

T1: HPLC: tR = 14.25 min; $[\alpha]_D^{22}$ -35.5 (*c*, 0.4 in H_2O); ν_{max} (neat) 3298 (s, OH), 1645, 1592 (2 × s, amide), 1515, 1345 (2 × s, N=O) cm^{-1}; δ_H (400 MHz, D_2O) 1.83 (3H, s, NHCOCH_3), 3.31-3.45 (3H, m, H-5b, H-4c, H-5c), 3.47 (1H, at, *J* 9.4 Hz, H-3c), 3.56 (1H, dd, $J_{2b,3b}$ 9.9 Hz, $J_{1b,2b}$ 3.7 Hz, H-2b), 3.58-3.90 (11H, m, H-2a, H-3a, H-4a, H-6a, H-3b, H-4b, H-6b, H-6b', H-2c, H-6c, H-6c'), 3.95 (1H, add, *J* 9.5 Hz, 4.5 Hz, H-5a), 4.20 (1H, dd, $J_{6a,6a'}$ 11.1 Hz, $J_{5a,6a'}$ 2.0 Hz, H-6a'), 4.51 (1H, d, $J_{1c,2c}$ 8.4 Hz, H-1c), 5.14 (1H, d, $J_{1b,2b}$ 3.5 Hz, H-1b), 5.24 (1H, d, $J_{1a,2a}$ 7.8 Hz, H-1a), 7.19 (2H, dd, *J* 9.3 Hz, 1.4 Hz, Ar-H), 8.23 (2H, dd, *J* 9.3 Hz, 1.3 Hz, Ar-H). δ_C (150 MHz, D_2O) 22.1 (q, NHCO*C*H_3), 55.5 (d, C-2c), 60.6 (2 × t, C-6b, C-6c), 67.8 (t, C-6a), 69.3, 69.8 (2 × d, C-5b, C-4c), 72.0 (d, C-2b), 72.3, 72.9 (3 × d, C-2a, C-3b, C-4b), 73.4 (d, C-5a), 73.9 (d, C-3c), 75.3 (d, C-3a), 75.8 (d, C-5c), 78.8 (d, C-4a), 99.1 (d, C-1a), 100.9, 101.0 (2 × d, C-1b, C-1c), 116.4, 126.2 (2 × d, Ar-C), 142.6, 161.7 (2 × s, Ar-C), 174.3 (s, C=O). HRMS (ESI) m/z: $[M + H]^+$ Calcd. For $C_{26}H_{39}N_2O_{18}$ 667.2192; Found 667.2194.

T2: HPLC: tR = 15.56 min; $[\alpha]_D^{22}$ -19 (*c*, 0.4 in H_2O); ν_{max} (neat) 3298 (s, OH), 1644, 1592 (2 × s, amide), 1515, 1344 (2 × s, N=O) cm^{-1}; δ_H (400 MHz, D_2O) 2.02 (3H, s, NHCOCH_3), 3.34-3.55 (5H, m, H-2b, H-3b, H-4b, H-4c, H-5c), 3.59-3.96 (13H, m, H-2a, H-3a, H-4a, H-5a H-6a, H-6a', H-5b, H-6b, H-2c, H-3c, H-6c, H-6c'), 4.09 (1H, ad, *J* 10.0 Hz, H-6b'), 4.50 (1H, $J_{1c,2c}$ 8.4 Hz, H-1c), 5.22 (1H, d, $J_{1a,2a}$ 7.8 Hz, H-1a), 5.37 (1H, d, $J_{1b,2b}$ 3.8 Hz, H-1b), 7.19 (2H, d, *J* 9.3 Hz, Ar-H), 8.21 (2H, *J* 9.3 Hz, Ar-H). δ_C (150 MHz, D_2O) 22.3 (q, NHCO*C*H_3), 60.5, 60.7 (2 × t, C-6a, C-6c), 68.1 (t, C-6b), 55.5, 69.2, 69.9, 71.5, 71.6, 72.6, 72.8, 73.8, 74.9, 75.9, 76.5 (12 × d, C-2a, C-3a, C-4a, C-5a, C-2b, C-3b, C-4b, C-5b, C-2c, C-3c, C-4c, C-5c), 99.3 (d, C-1a), 99.7 (C-1b), 101.5 (C-1c), 116.5, 126.1 (2 × d, Ar-C), 142.6, 161.7 (2 × s, Ar-C), 174.5 (s, C=O). HRMS (ESI) m/z: $[M + H]^+$ Calcd. For $C_{26}H_{39}N_2O_{18}$ 667.2192; Found 667.2208.

References:

1. Paramasivam, S.; Fairbanks, A. J., *Carbohydr. Res.* **2019,** *477*, 11-19.
2. Cai, Y.; Ling, C.-C.; Bundle, D. R., *Org. Lett.* **2005,** *7*, 4021-4024.
3. Arita, H.; Fukukawa, K.; Matsushima, Y., *Bull. Chem. Soc. Jpn.* **1972,** *45*, 3614-3619.
4. Pistia-Brueggeman, G.; Hollingsworth, R. I., *Carbohydr. Res.* **2003,** *338*, 455-458.
5. Matta, K. L.; Johnson, E. A.; Barlow, J. J., *Carbohydr. Res.* **1973,** *26*, 215-218.
6. Rochepeau-Jobron, L.; Jacquinet, J.-C., *Carbohydr. Res.* **1997,** *305*, 181-191.
7. Miyai, K.; Jeanloz, R. W., *Carbohydr. Res.* **1972,** *21*, 45-55.
8. Morozzi, C.; Sedláková, J.; Serpi, M.; Avigliano, M.; Carbajo, R.; Sandoval, L.; Valles-Ayoub, Y.; Crutcher, P.; Thomas, S.; Pertusati, F., *J. Med. Chem.* **2019,** *62*, 8178-8193.
9. Rana, S. S.; Barlow, J. J.; Matta, K. L., *Carbohydr. Res.* **1981,** *96*, 79-85.
10. Lycknert, K.; Edblad, M.; Imberty, A.; Widmalm, G., *Biochemistry* **2004,** *43*, 9647-9654.
11. Murata, T.; Hattori, T.; Amarume, S.; Koichi, A.; Usui, T., *Eur. J. Biochem.* **2003,** *270*, 3709-3719.
12. Matta, K. L.; Barlow, J. J., *Carbohydr. Res.* **1977,** *53*, 209-216.
13. Fialová, P.; Carmona, A. T.; Robina, I.; Ettrich, R.; Sedmera, P.; Přikrylová, V.; Petrásková-Hušáková, L.; Křen, V., *Tetrahedron Lett.* **2005,** *46*, 8715-8718.
14. Zurabyan, S.; Volosyuk, T.; Khorlin, A., *Carbohydr. Res.* **1969,** *9*, 215-220.
15. Abbas, S. A.; Matta, K. L., *Carbohydr. Res.* **1983,** *124*, 115-121.

_n_-Pentenyl 2-acetamido-2-deoxy-β-D-glucopyranoside 5a, ^{1}H NMR (400 MHz, D_2O)

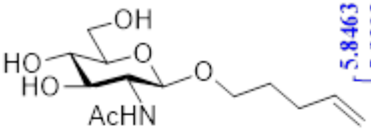

5a

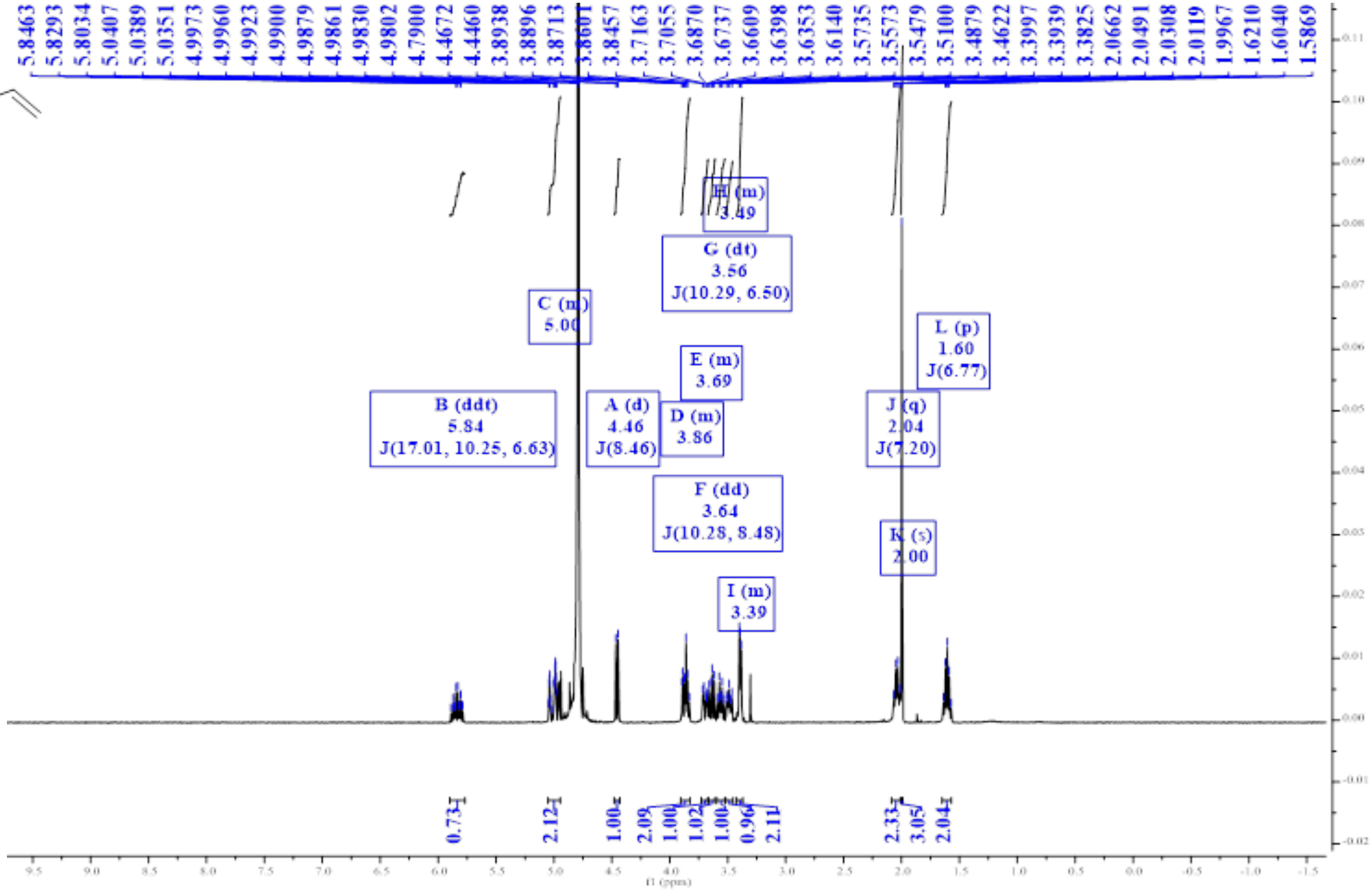

Benzyl 2-acetamido-2-deoxy-β-D-glucopyranoside 5b, ^{1}H NMR (400 MHz, D_2O)

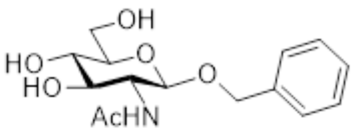

5b

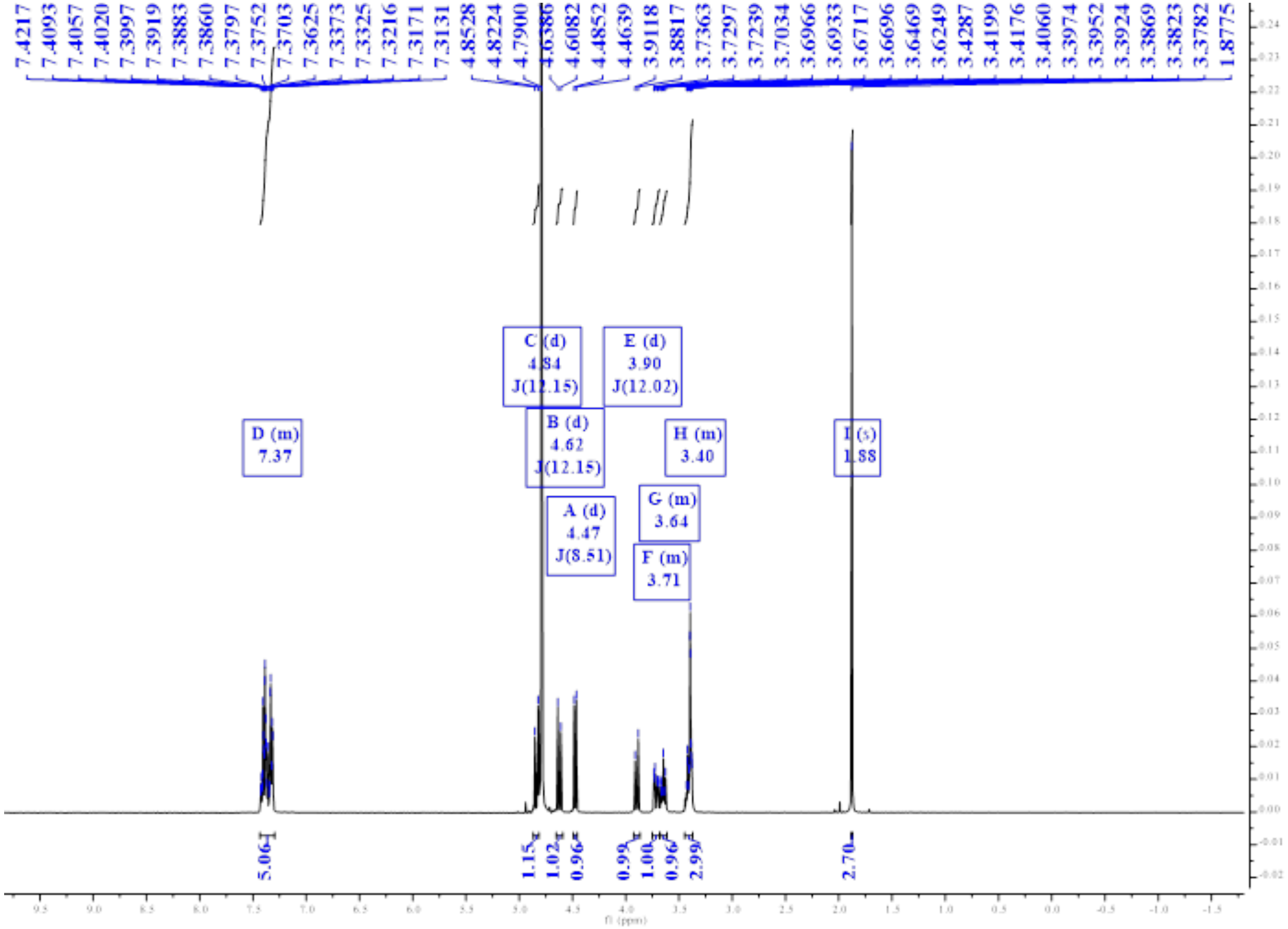

Isopropyl 2-acetamido-2-deoxy-β-D-glucopyranoside 5c, ^{1}H NMR (400 MHz, D_2O)

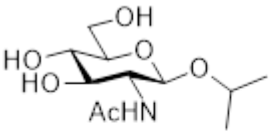

5c

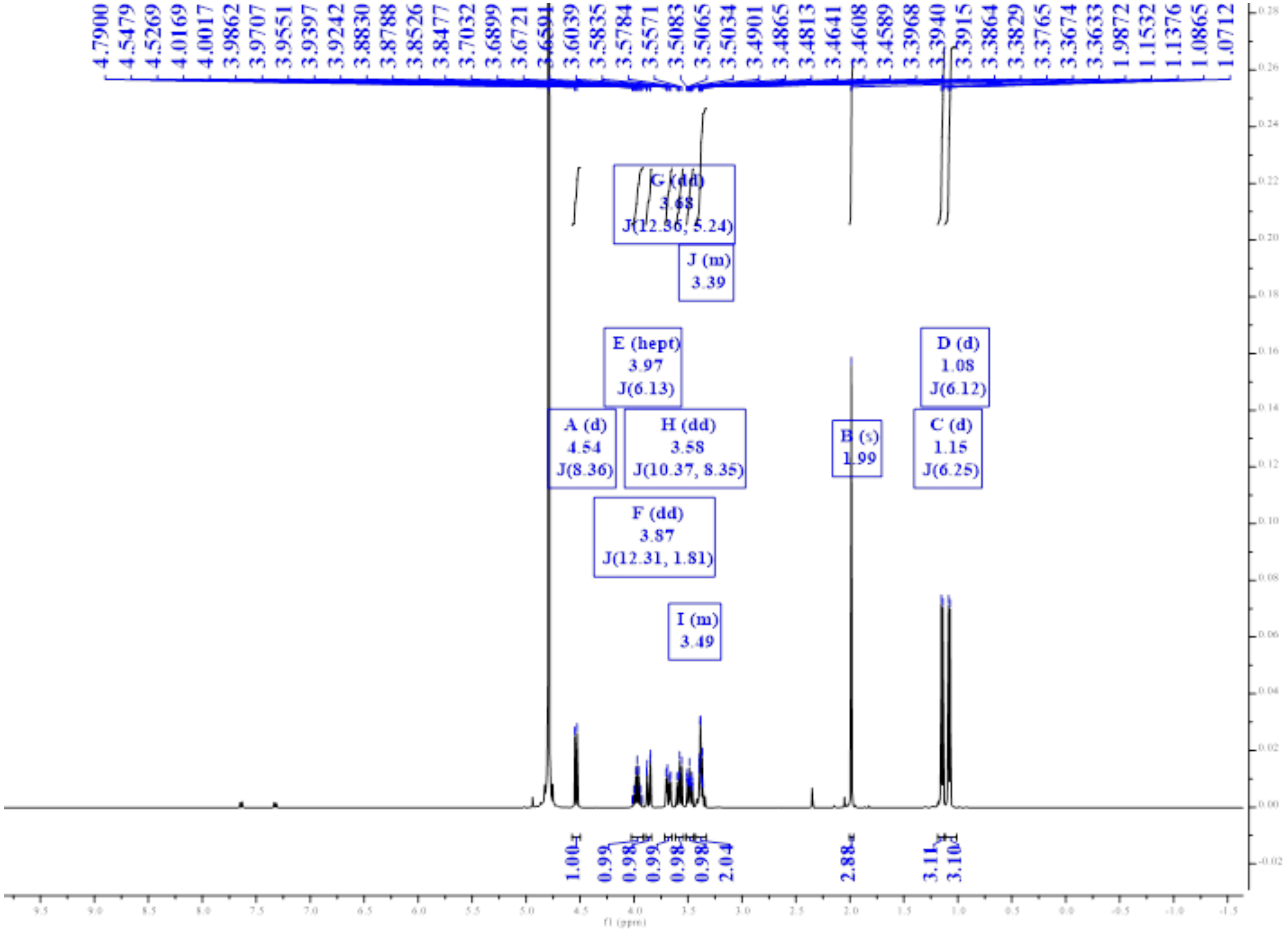

***t*-Butyl 2-acetamido-2-deoxy-β-D-glucopyranoside 5d**, ^{1}H NMR (400 MHz, D_2O)

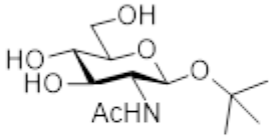

5d

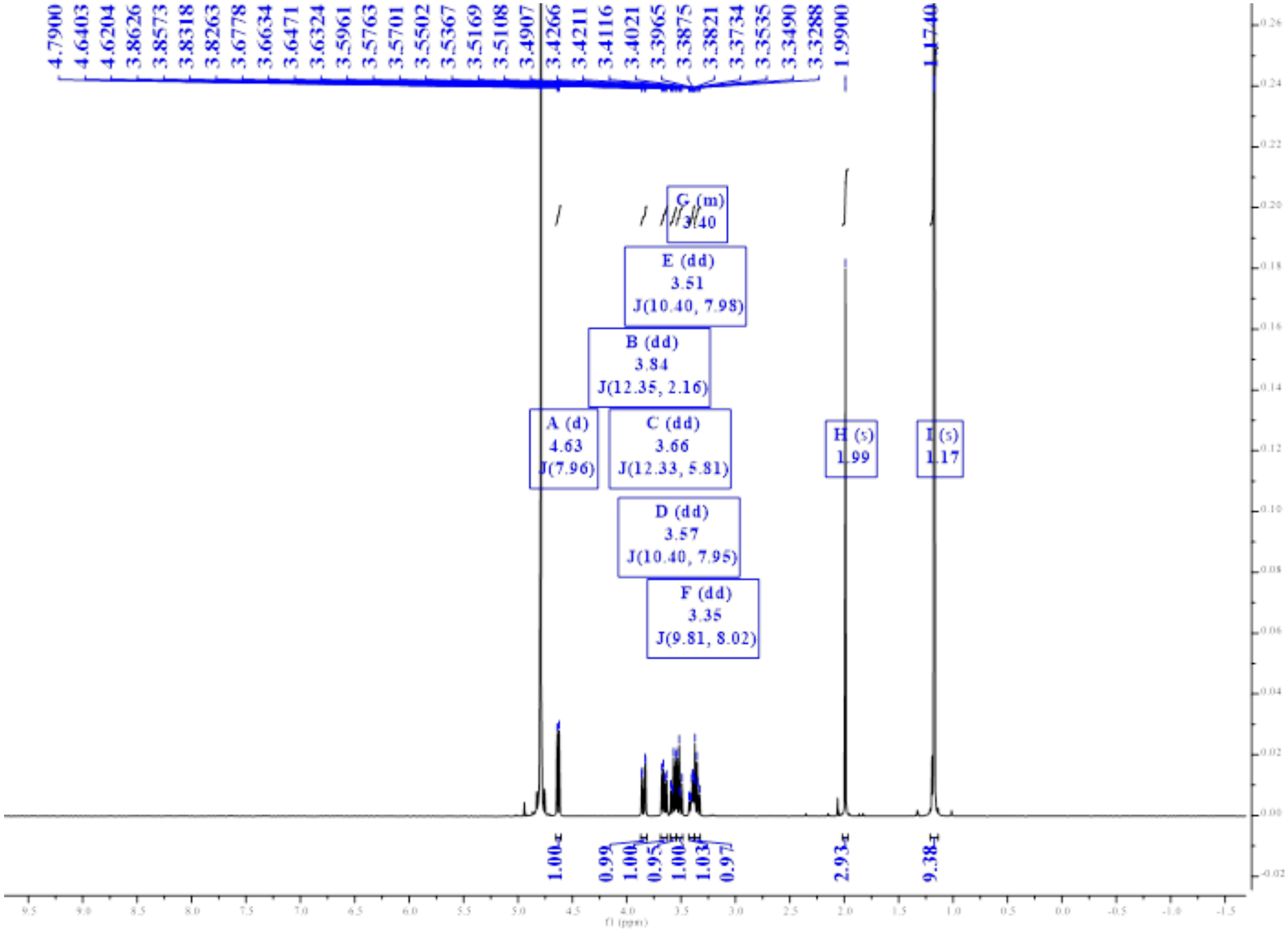

Benzyl 2-acetamido-2-deoxy-β-D-galactopyranoside 5e, ^{1}H NMR (400 MHz, D_2O)

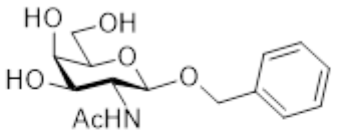

5e

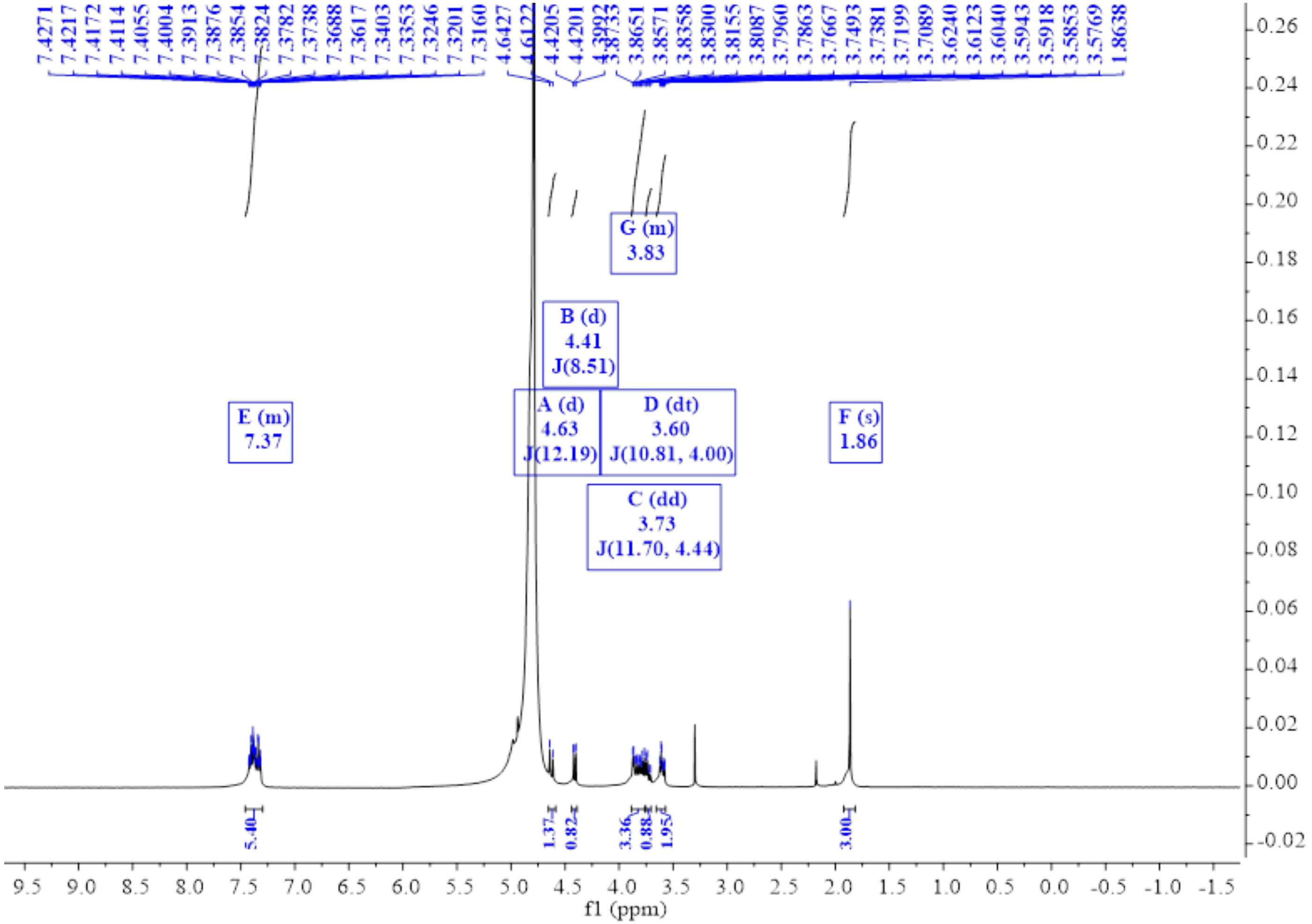

Benzyl 2-acetamido-2-deoxy-α-D-mannopyranoside 5f, (400 MHz, CD_3OD)

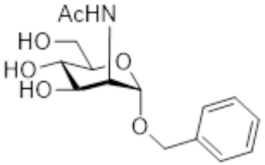

5f

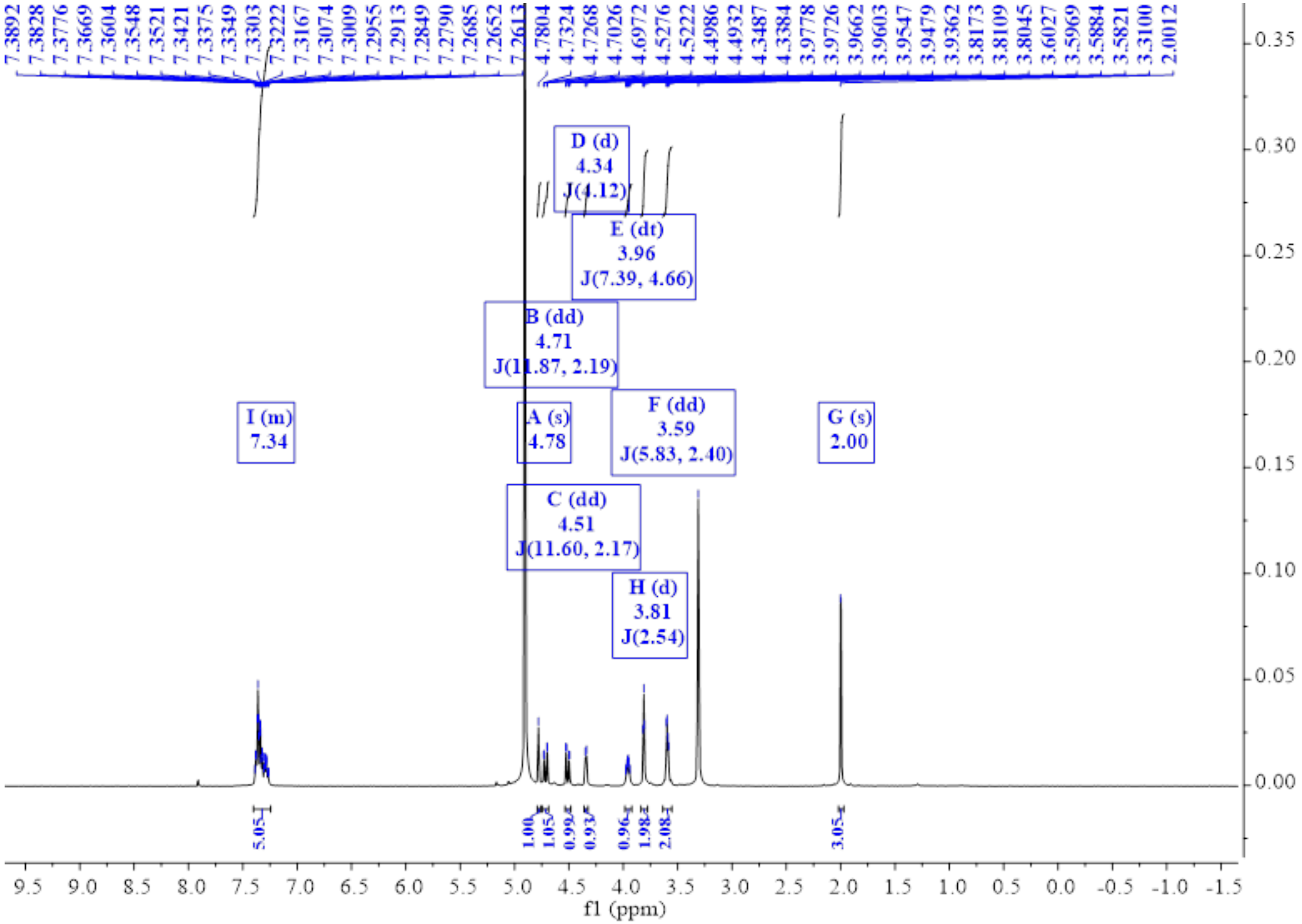

***p*-Nitrophenyl 2-acetamido-2-deoxy-β-D-glucopyranosyl-(1→6)-β-D-glucopyranoside 4a**, ^{1}H NMR (400 MHz, D_2O)

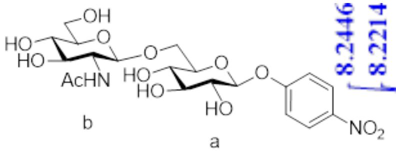

4a

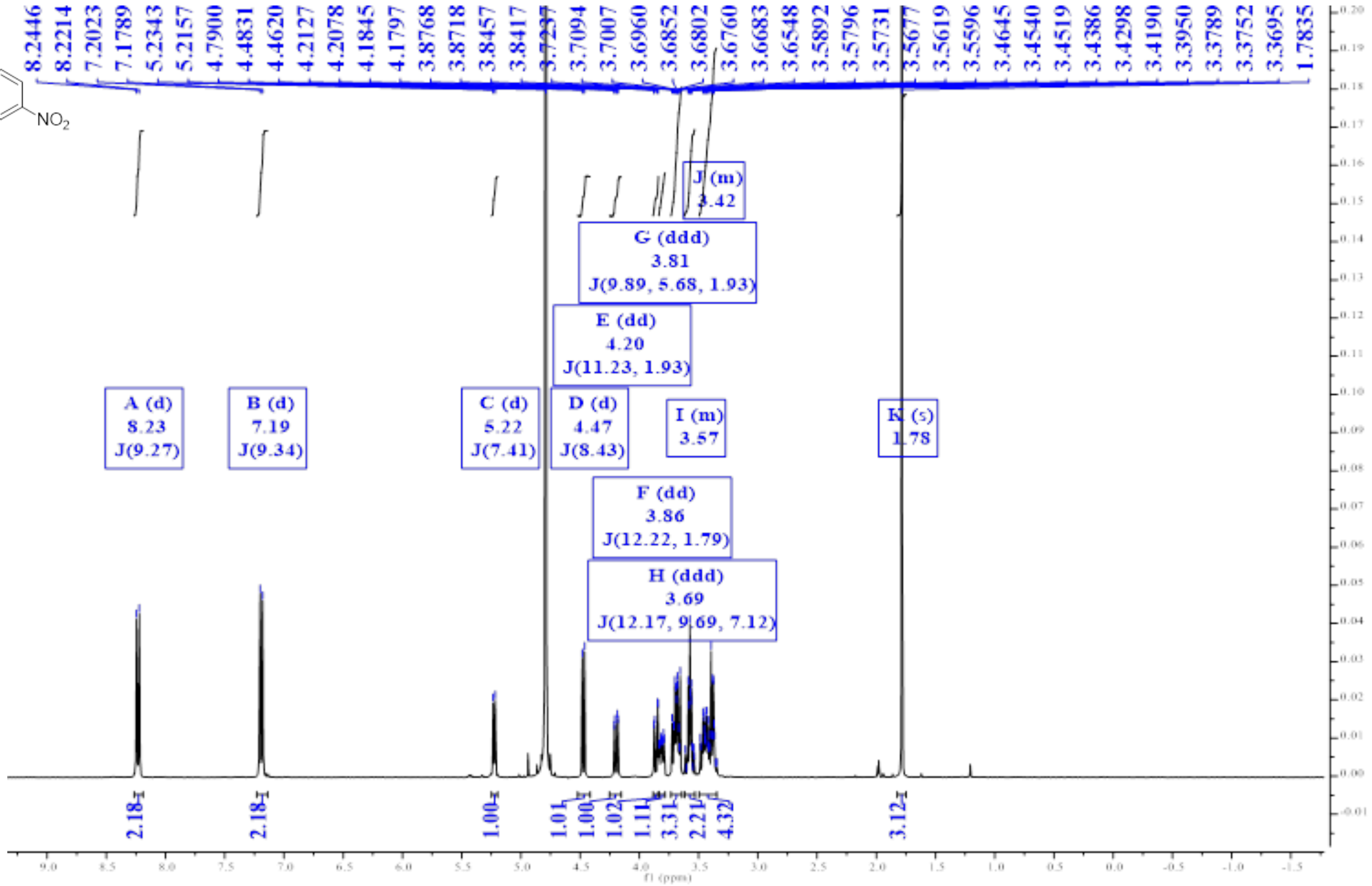

Compound **4a**, ^{13}C NMR (150 MHz, D_2O)

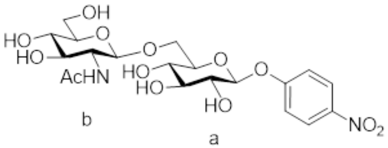

4a

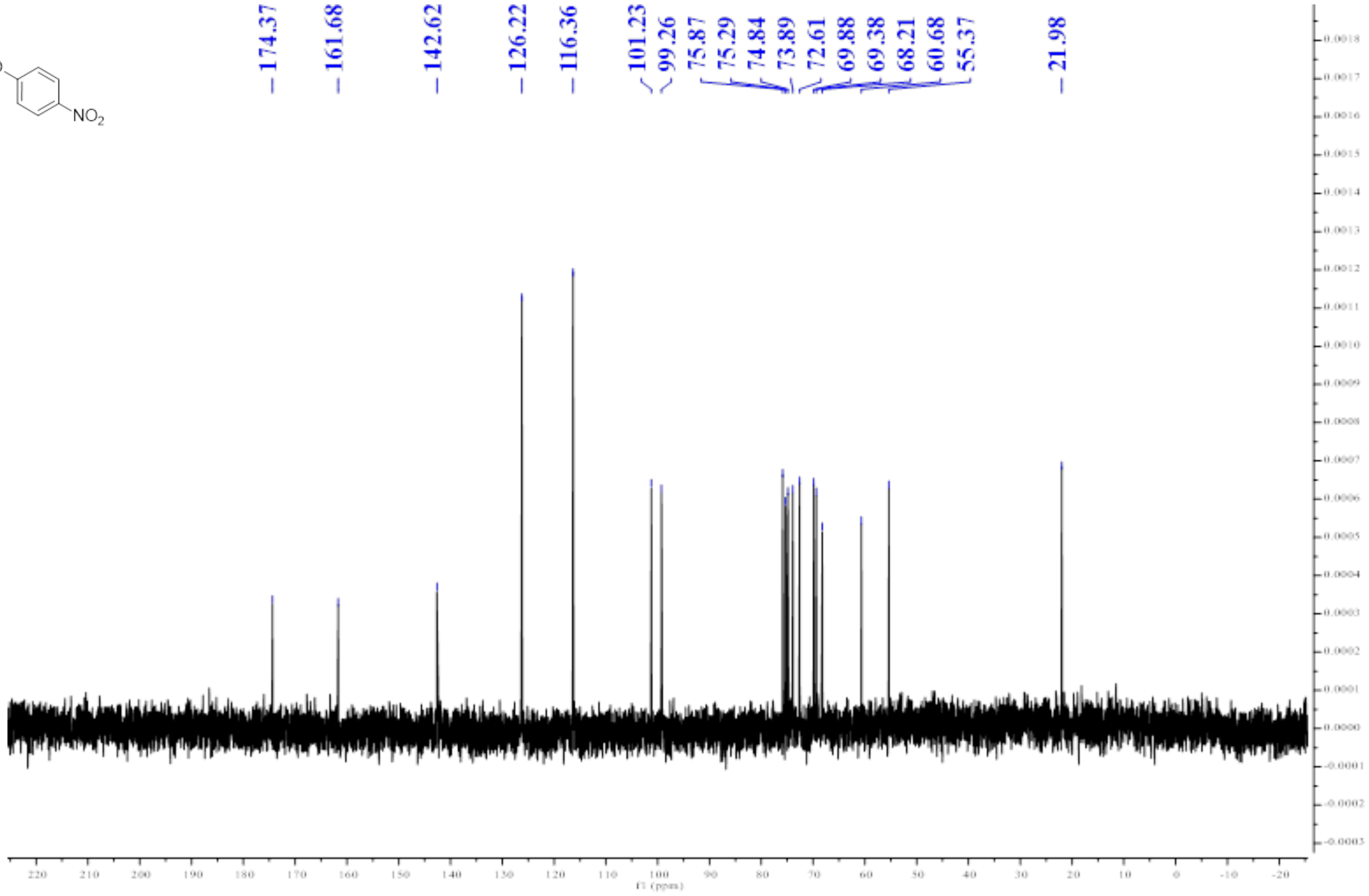

Compound **4a**, HSQC (600 MHz, D_2O)

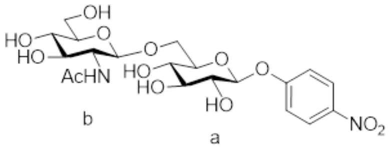

4a

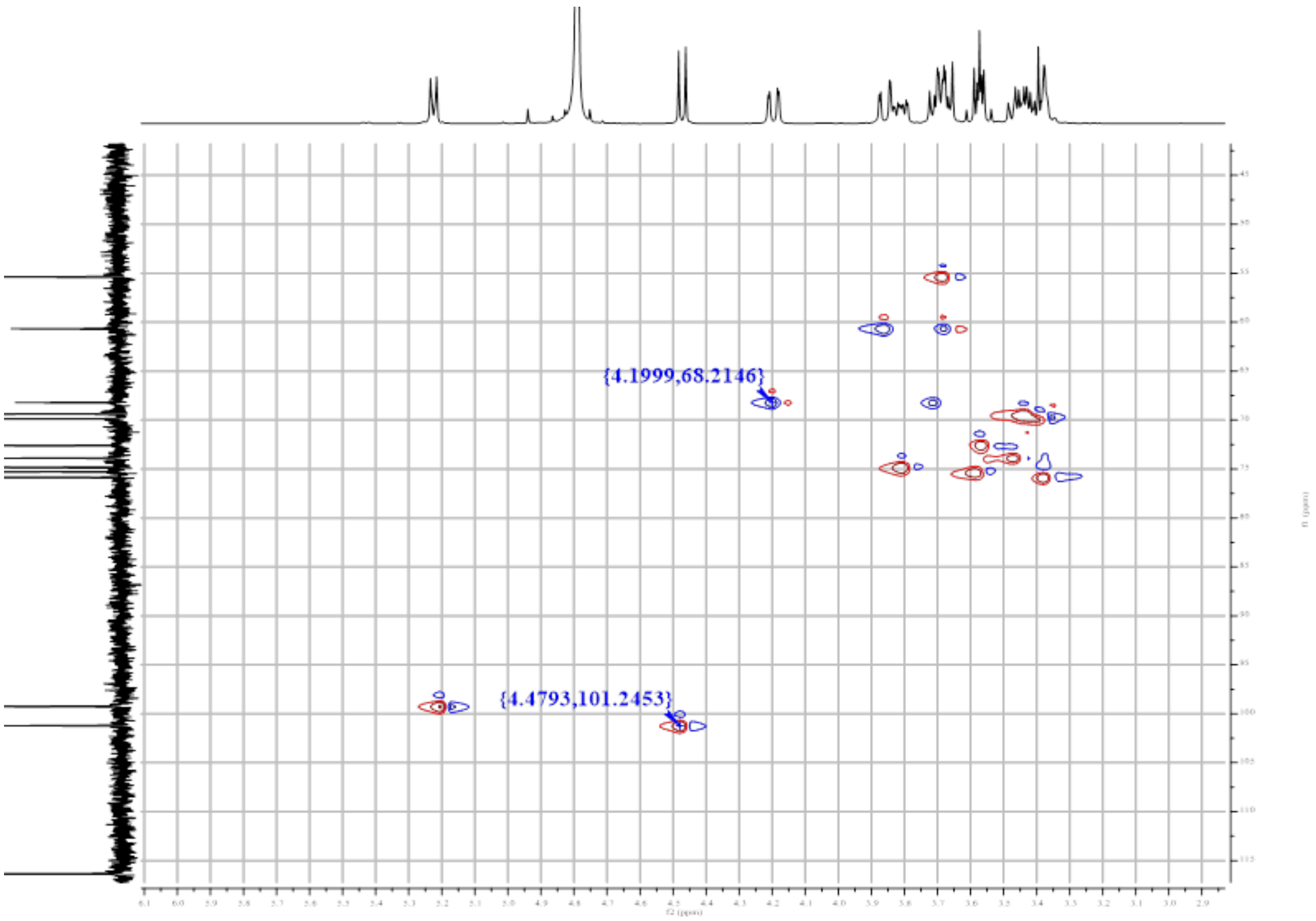

Compound **4a**, HMBC (600 MHz, D_2O)

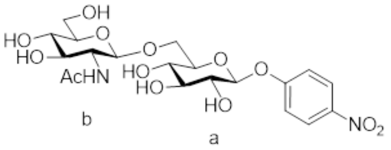

4a

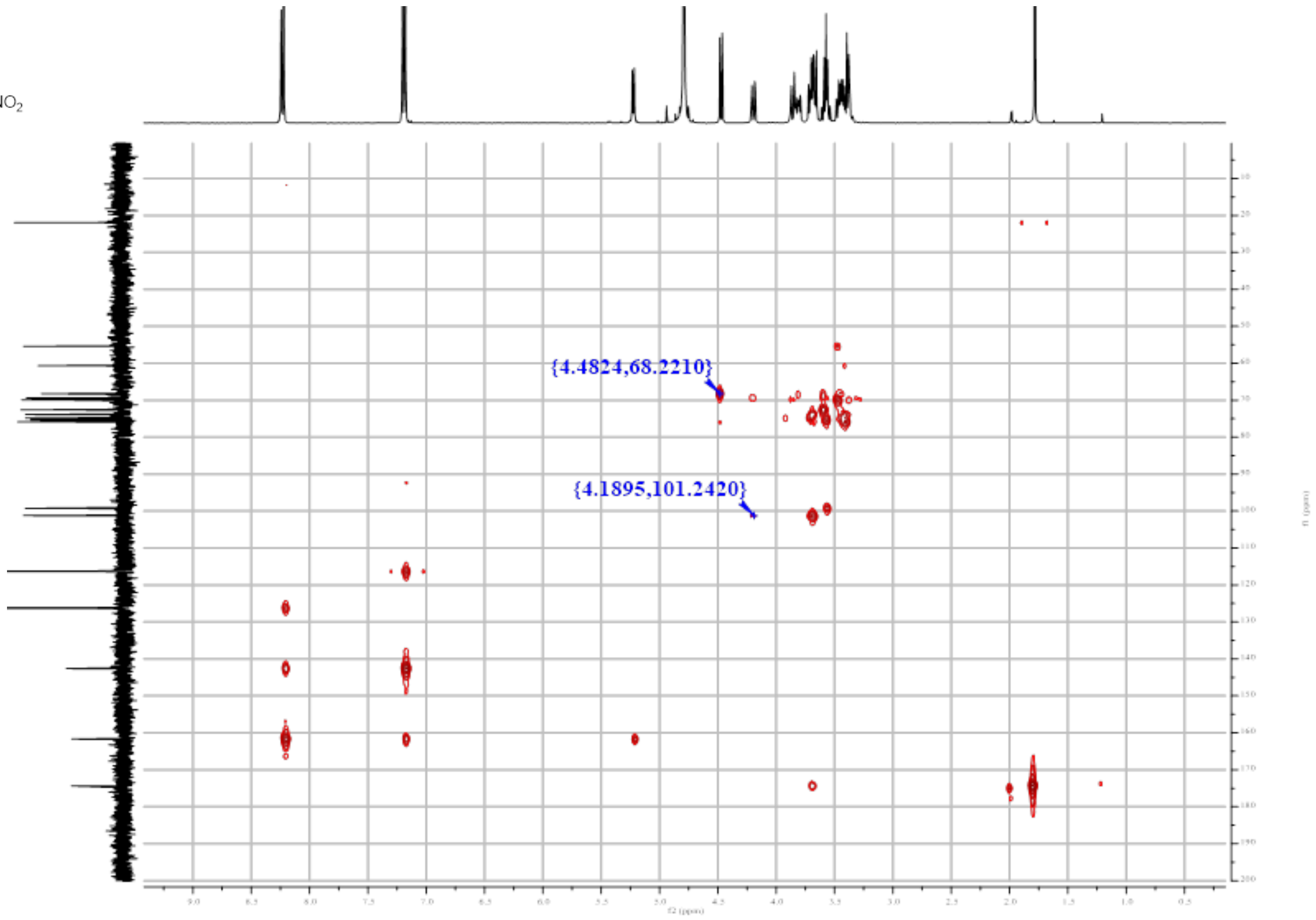

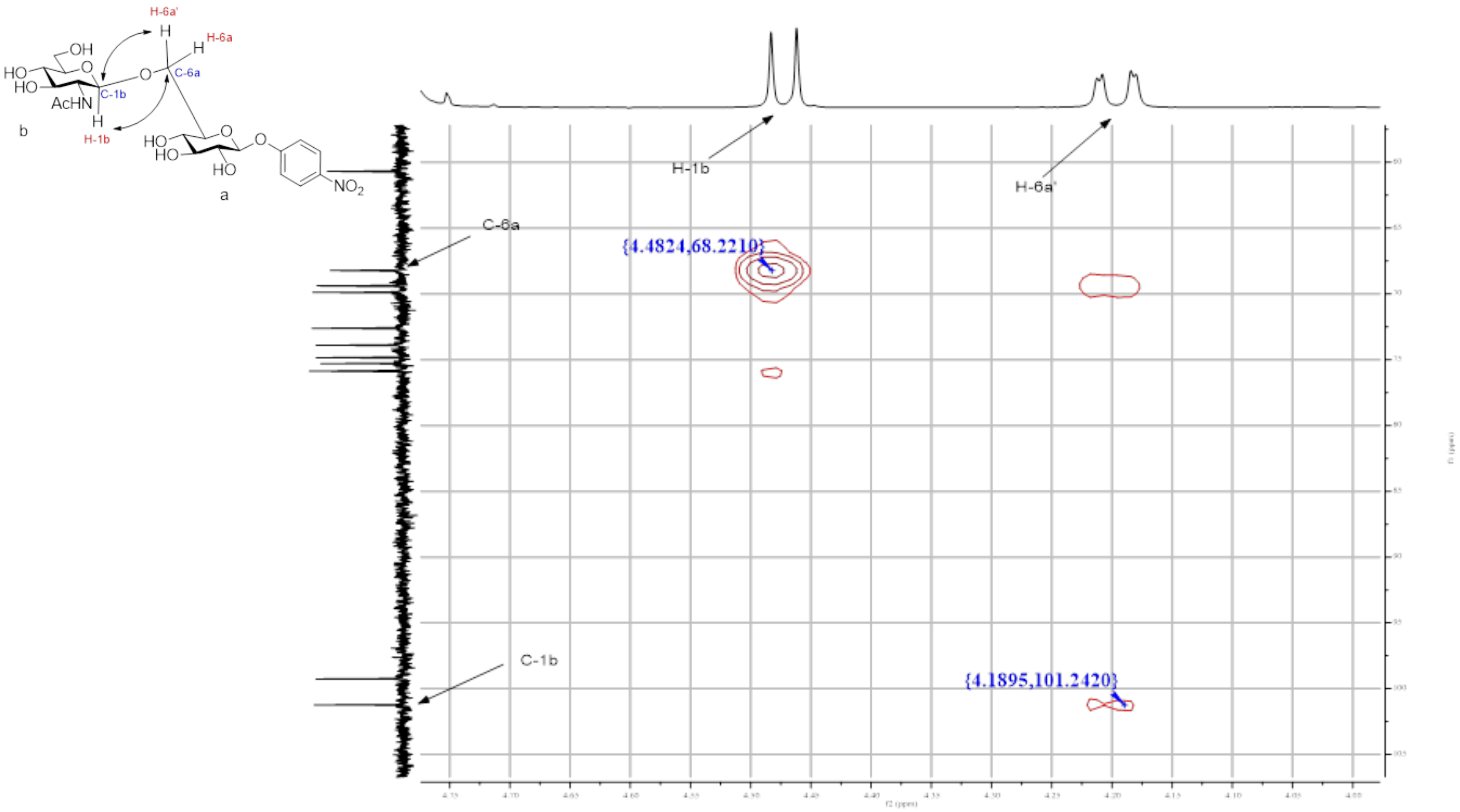

H-6a'
H-6a
C-6a
C-1b
H-1b
AcHN
NO2
b
a
H-1b
H-6a'
C-6a
{4.4824,68.2210}
C-1b
{4.1895,101.2420}

***p*-Nitrophenyl 2-acetamido-2-deoxy-β-D-glucopyranosyl-(1→6)-α-D-mannopyranoside 4b**, ^{1}H NMR (400 MHz, D_2O)

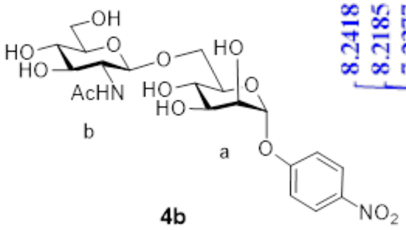

4b

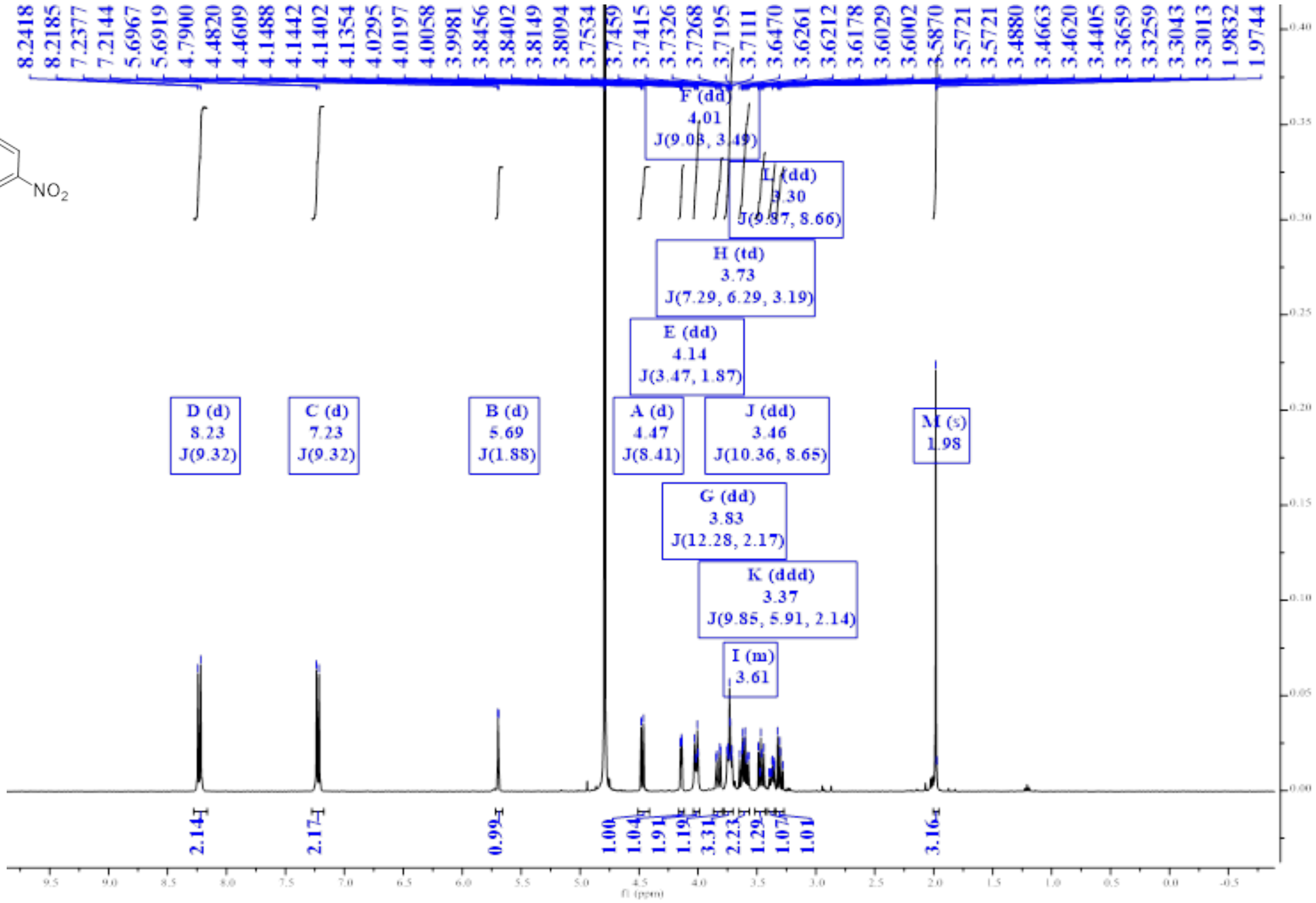

Compound **4b**, DEPT 135 NMR (100 MHz, D_2O)

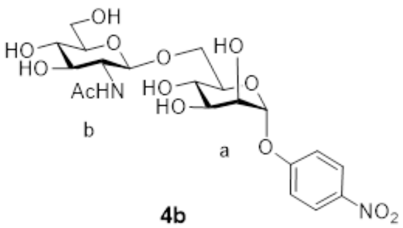

4b

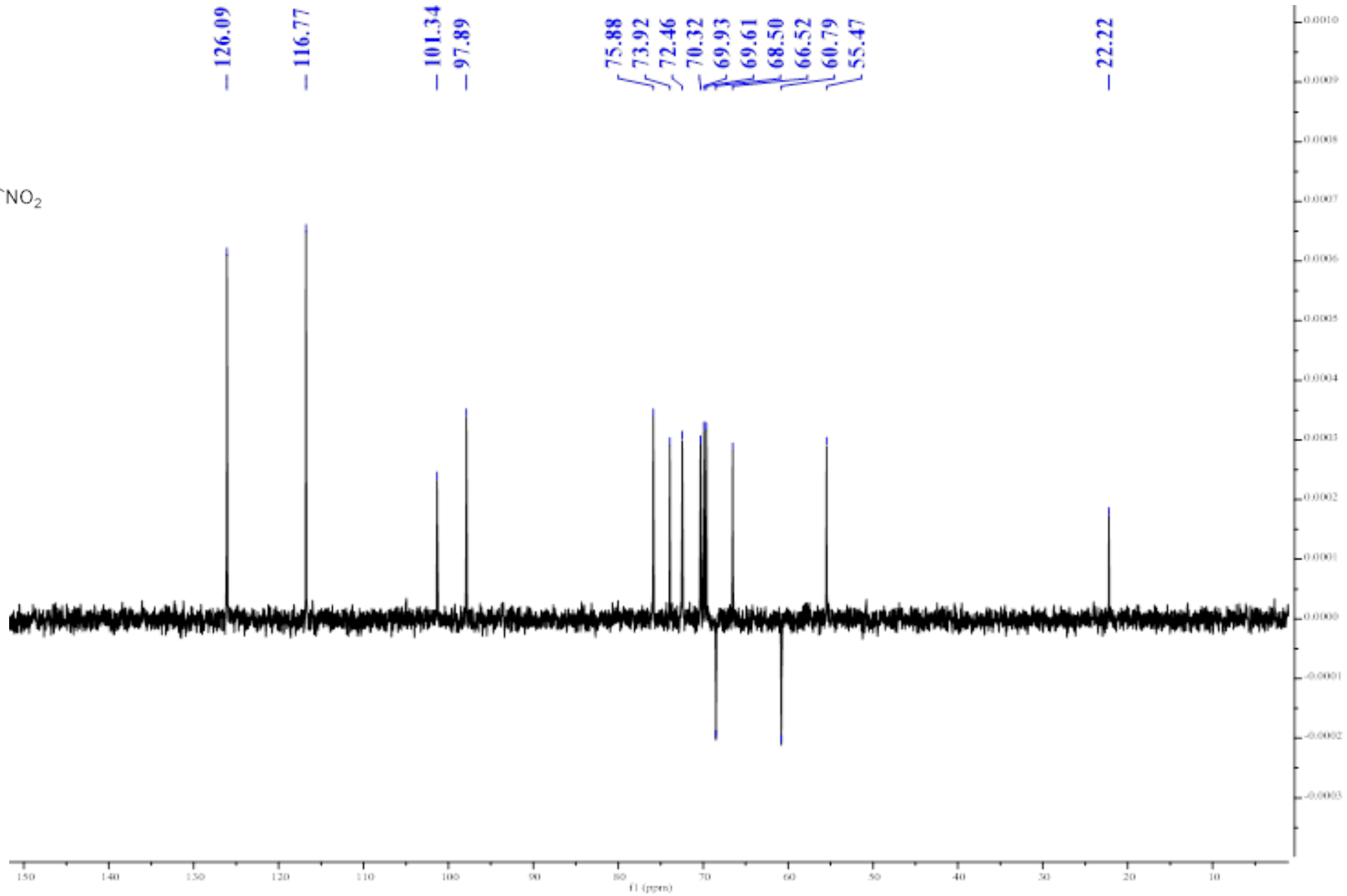

Compound **4b**, ^{13}C NMR (150 MHz, D_2O)

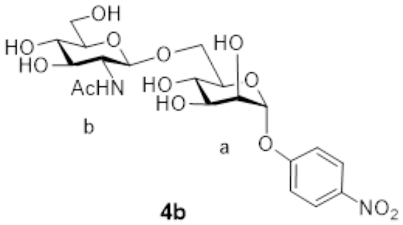

4b

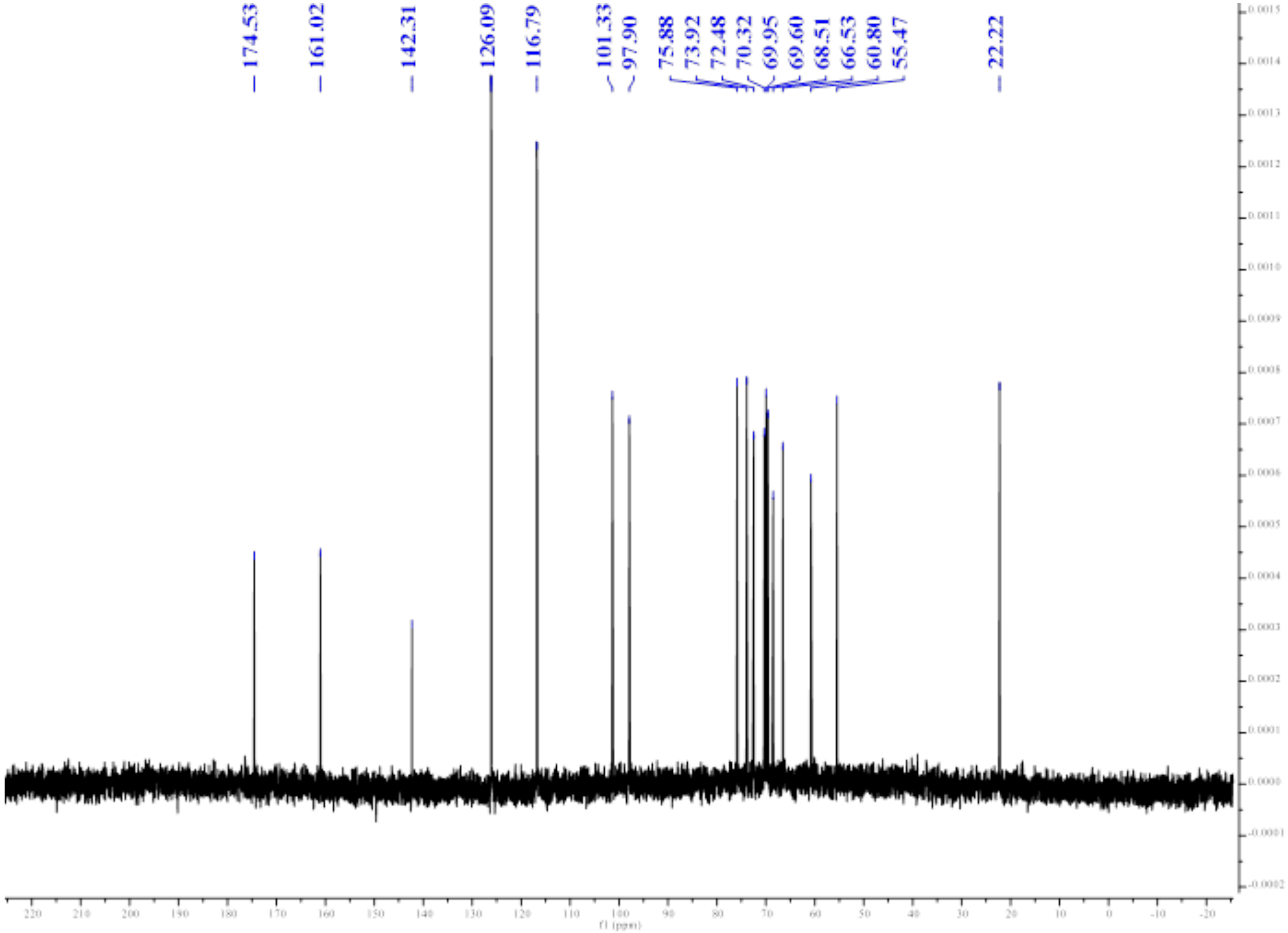

Compound **4b**, HMQC (600 MHz, D_2O)

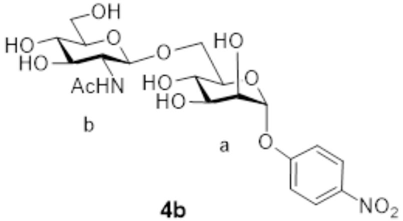

4b

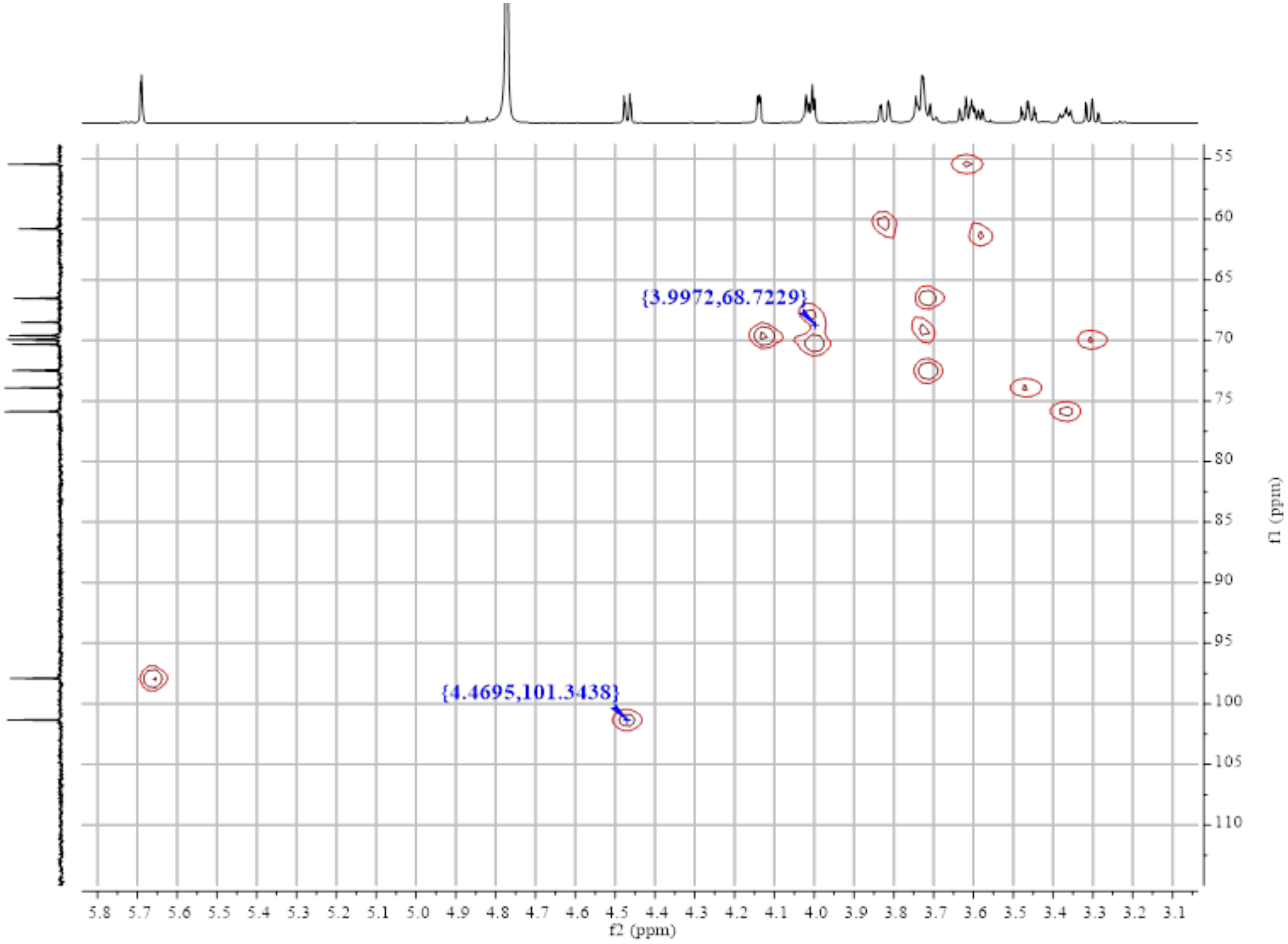

Compound **4b**, HMBC (600 MHz, D_2O)

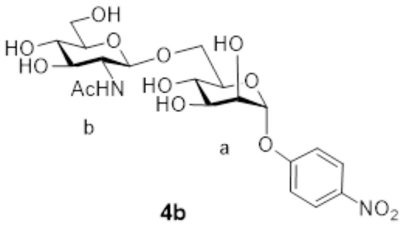

4b

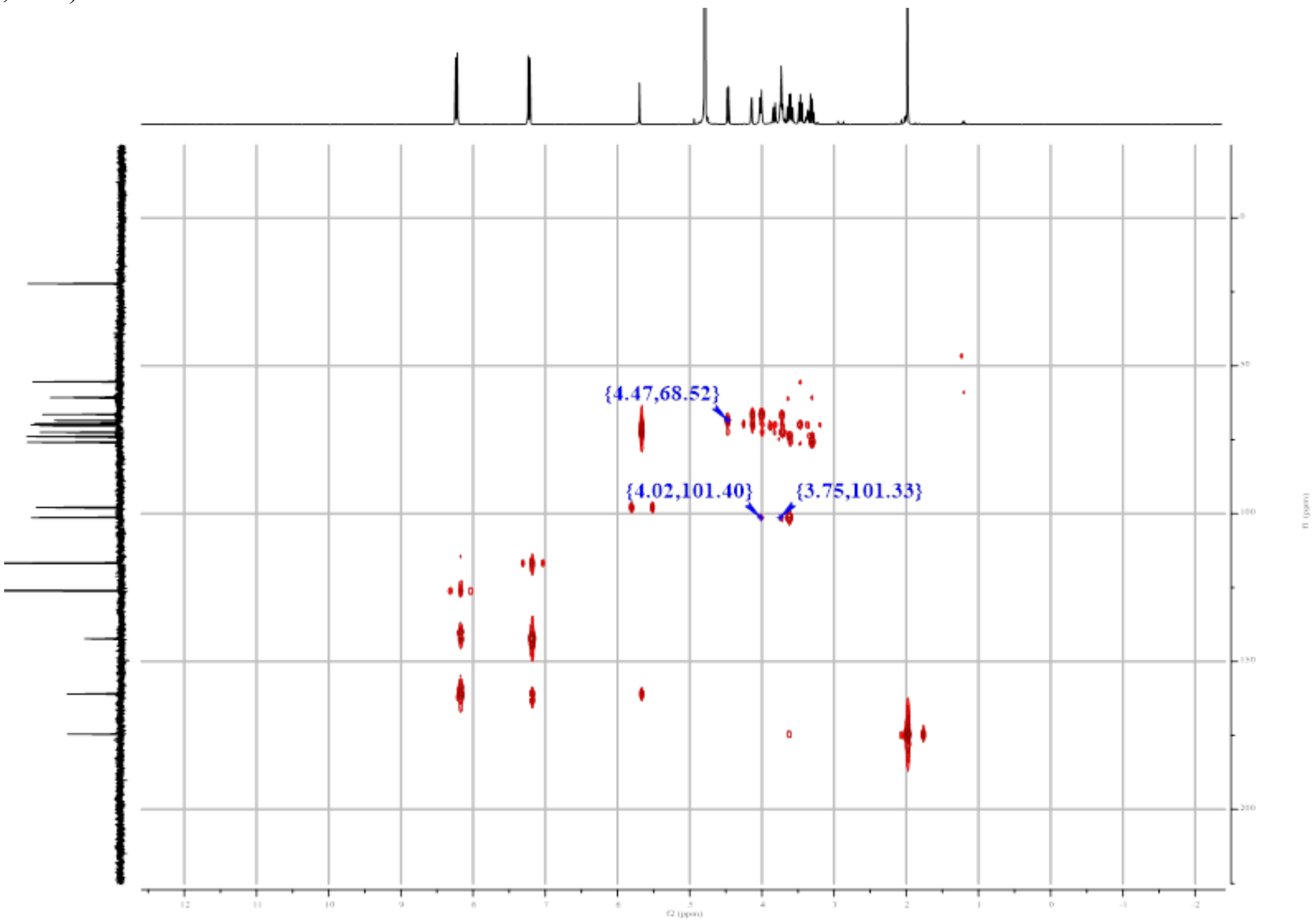

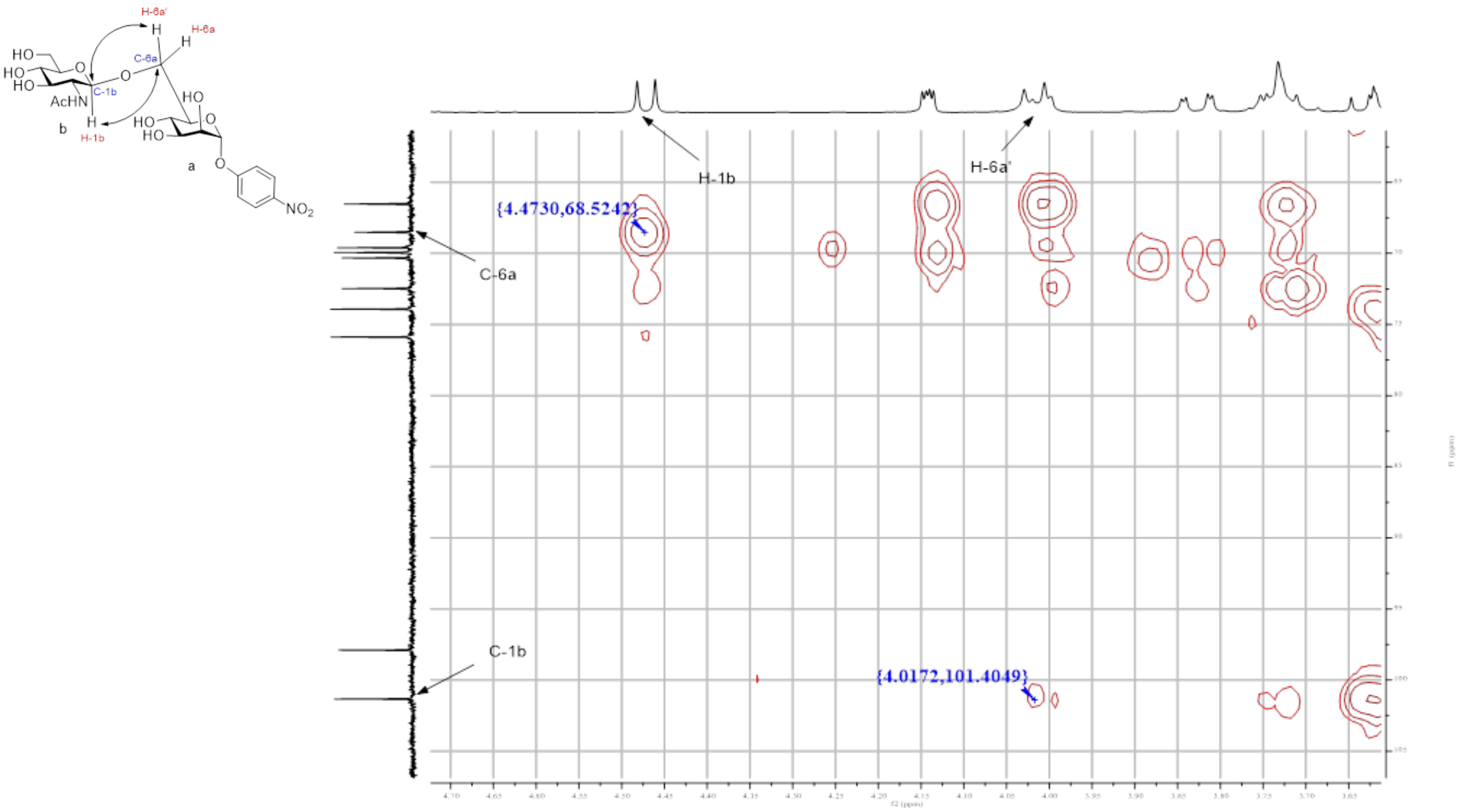

H-6a'
H-6a
C-6a
C-1b
AcHN
H-1b
b
a
NO2
{4.4730,68.5242}
{4.0172,101.4049}
H-1b
H-6a'
C-6a
C-1b

Methyl 2-acetamido-2-deoxy-β-D-glucopyranosyl-(1→6)-α-D-mannopyranoside 4c, ^{1}H NMR (400 MHz, D_2O)

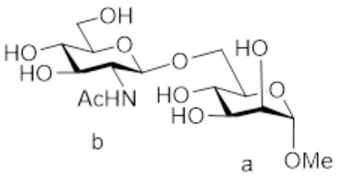

4c

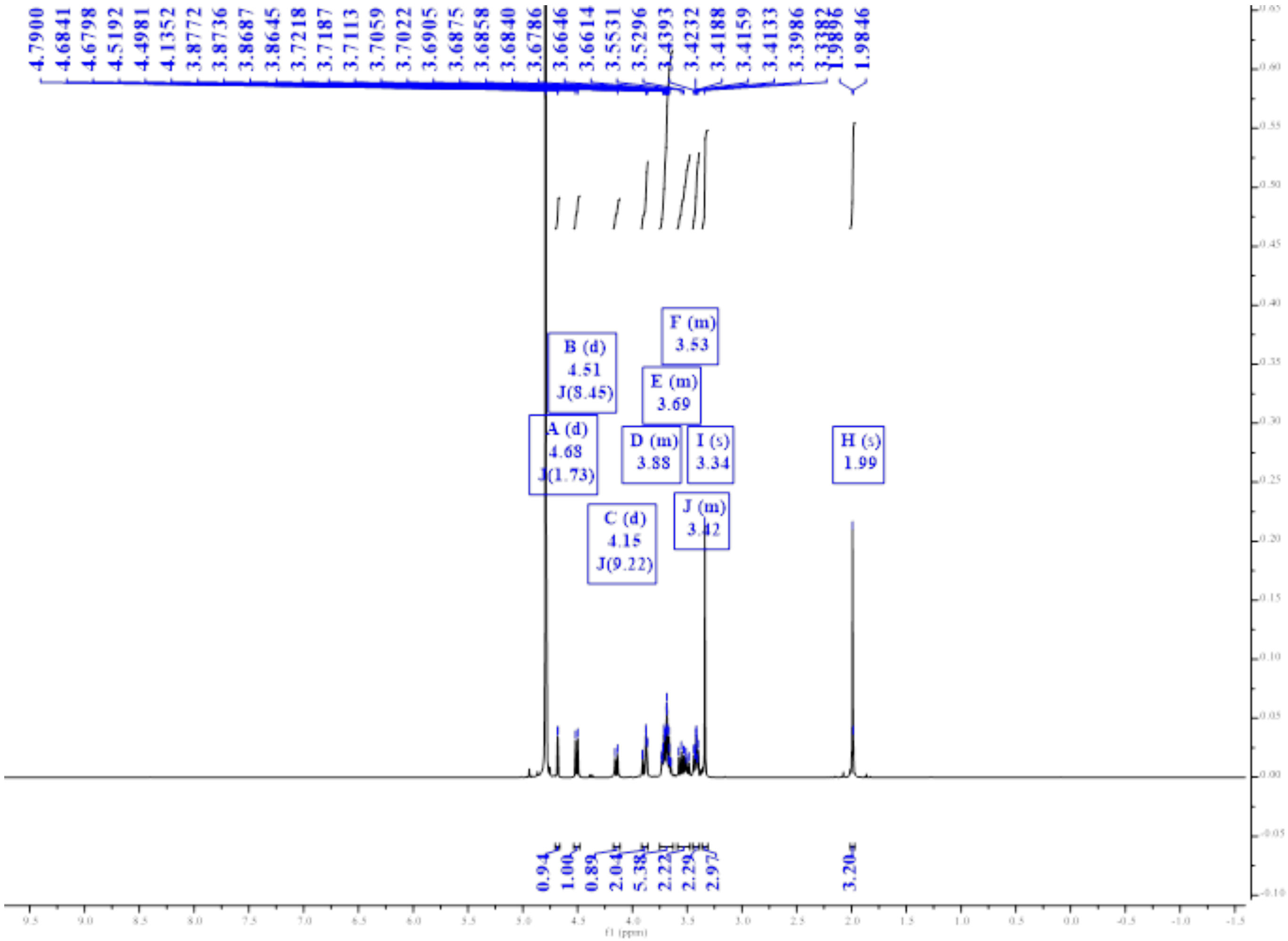

***p*-Methoxy phenyl 2-acetamido-2-deoxy-β-D-glucopyranosyl-(1→6)-α-D-mannopyranoside 4d**, ^{1}H NMR (600 MHz, D_2O)

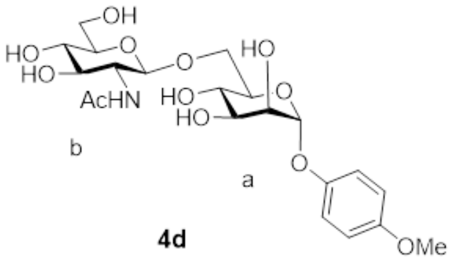

4d

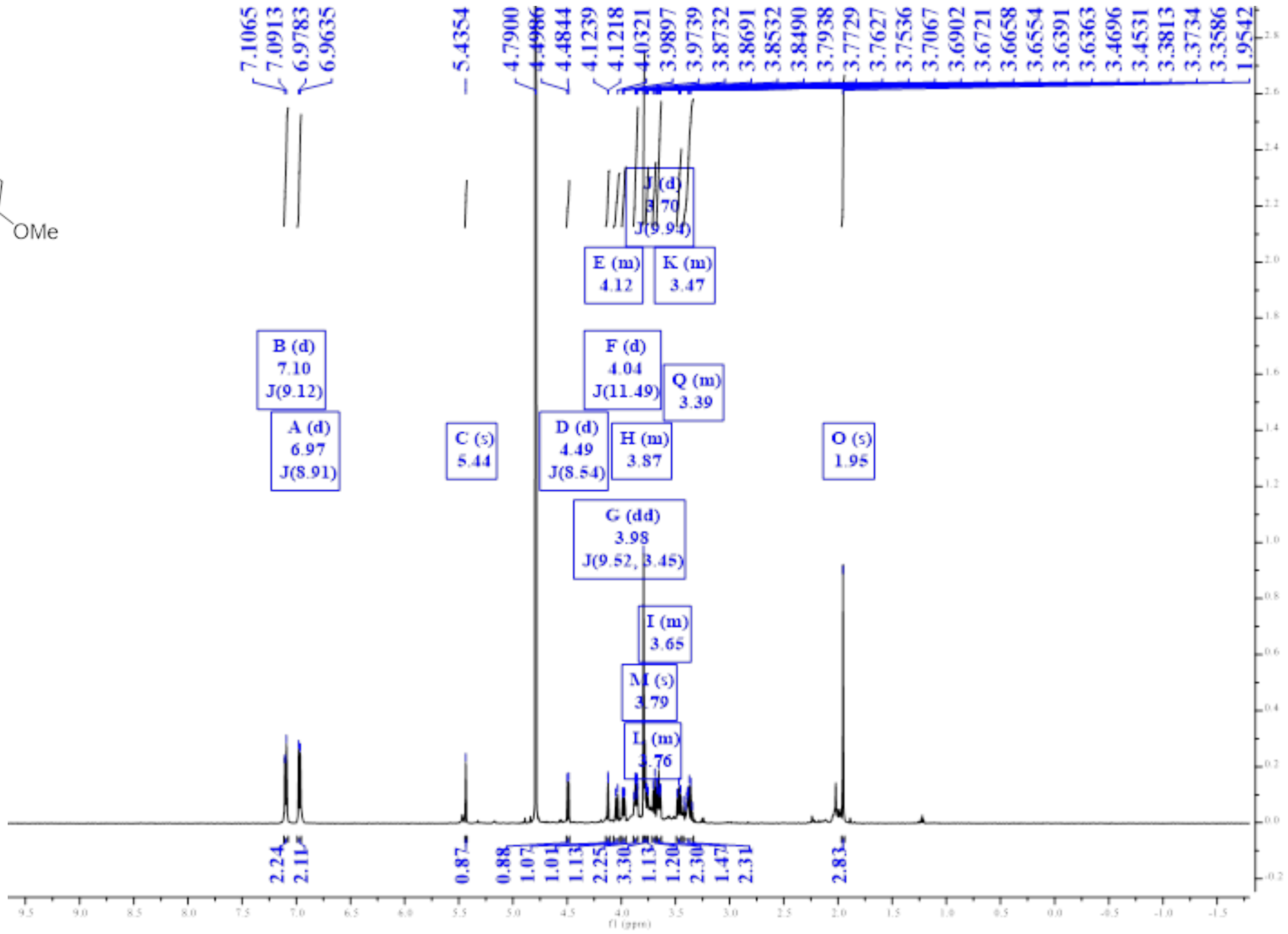

Compound **4d**, ^{13}C (150 MHz, D_2O)

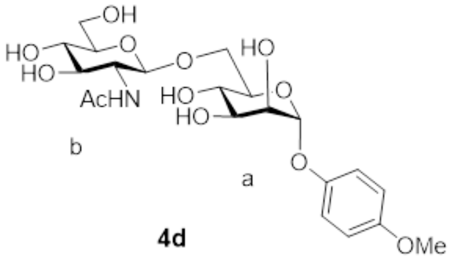

4d

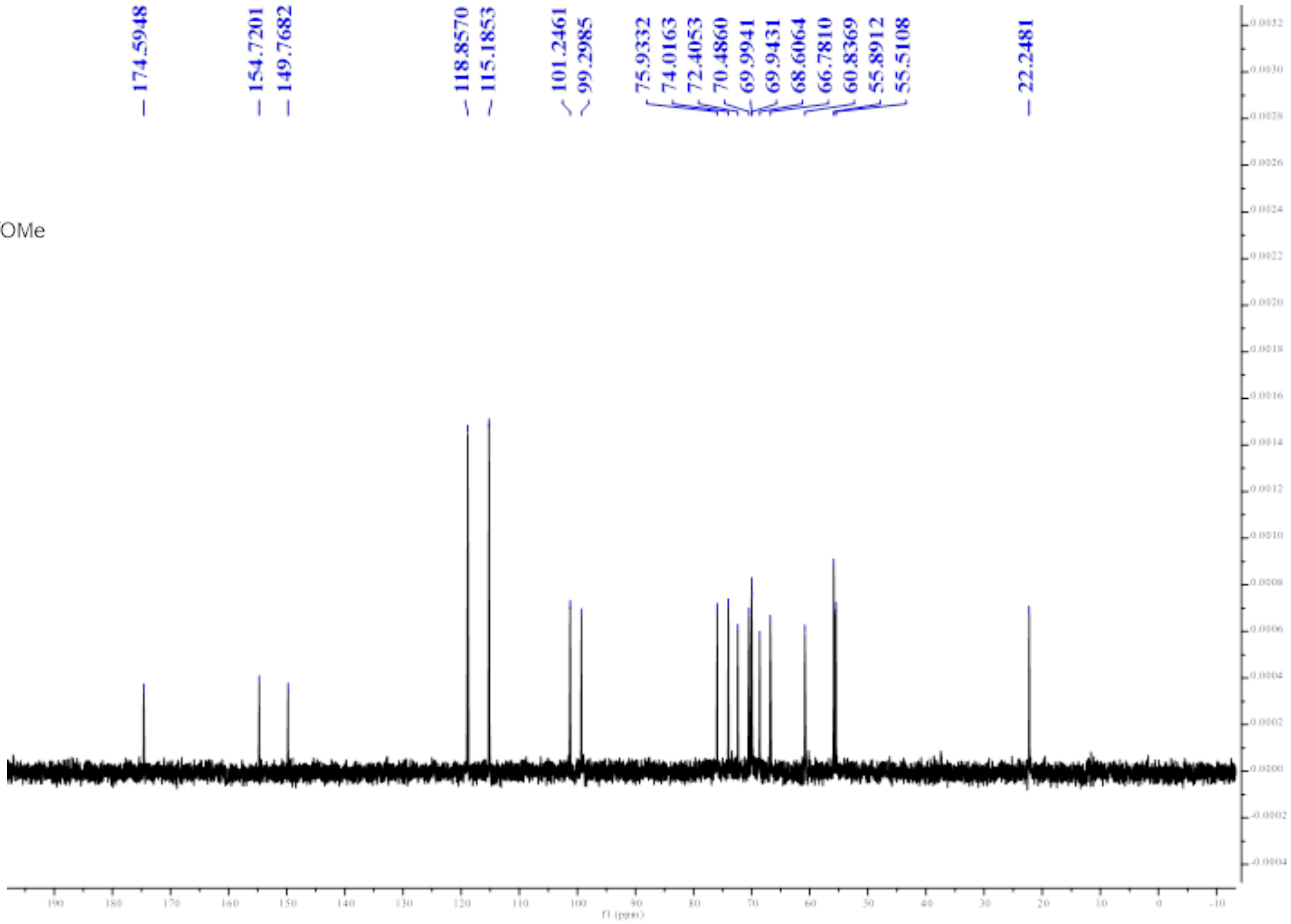

Compound **4d**, DEPT 135 (150 MHz, D_2O)

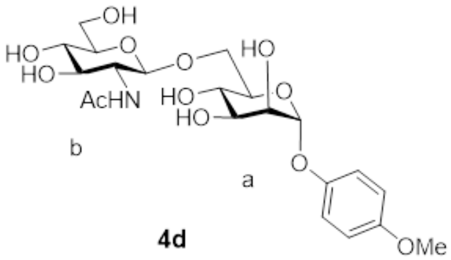

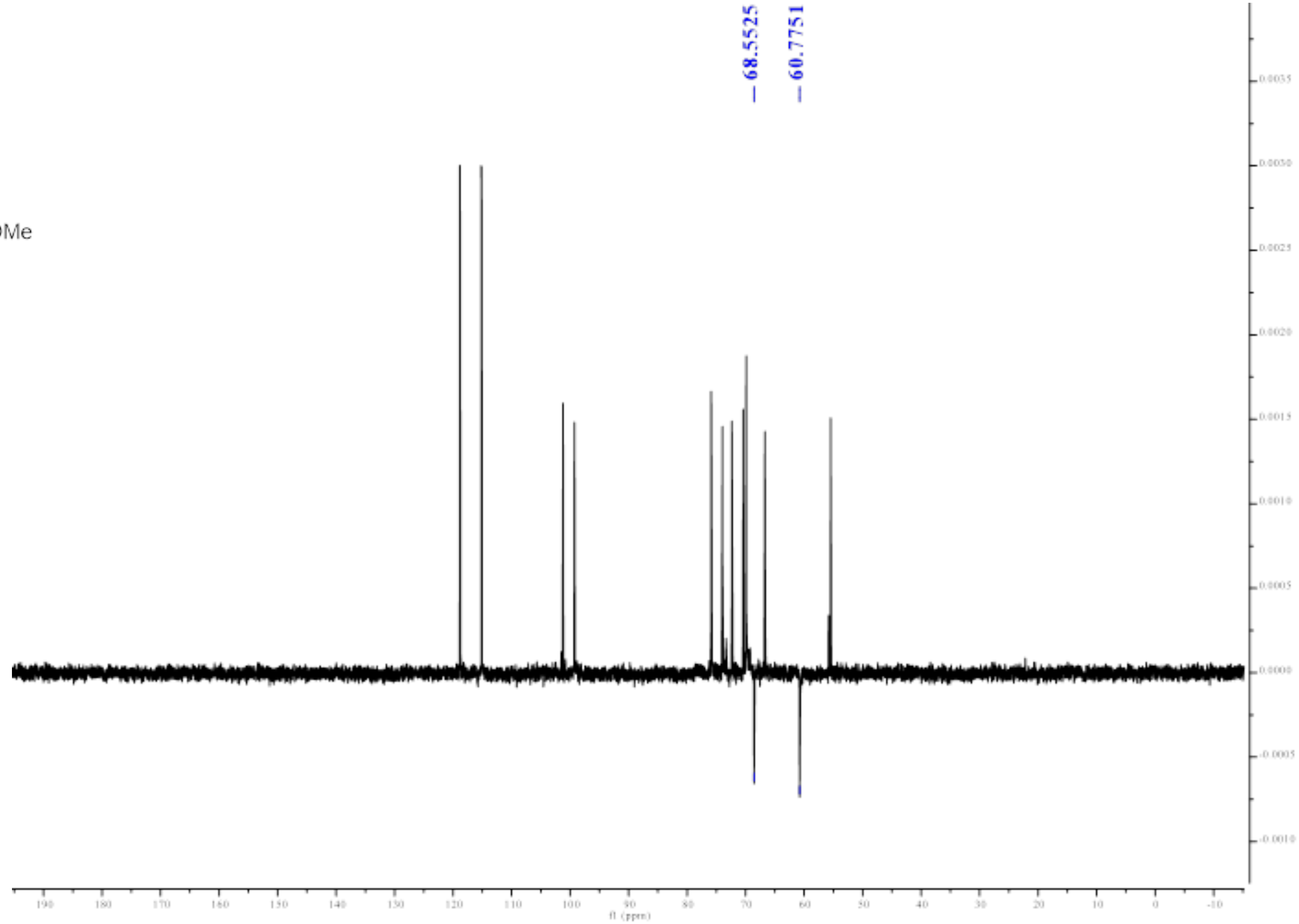

Compound **4d**, HMQC (600 MHz, D_2O)

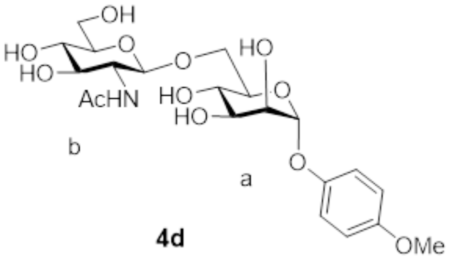

4d

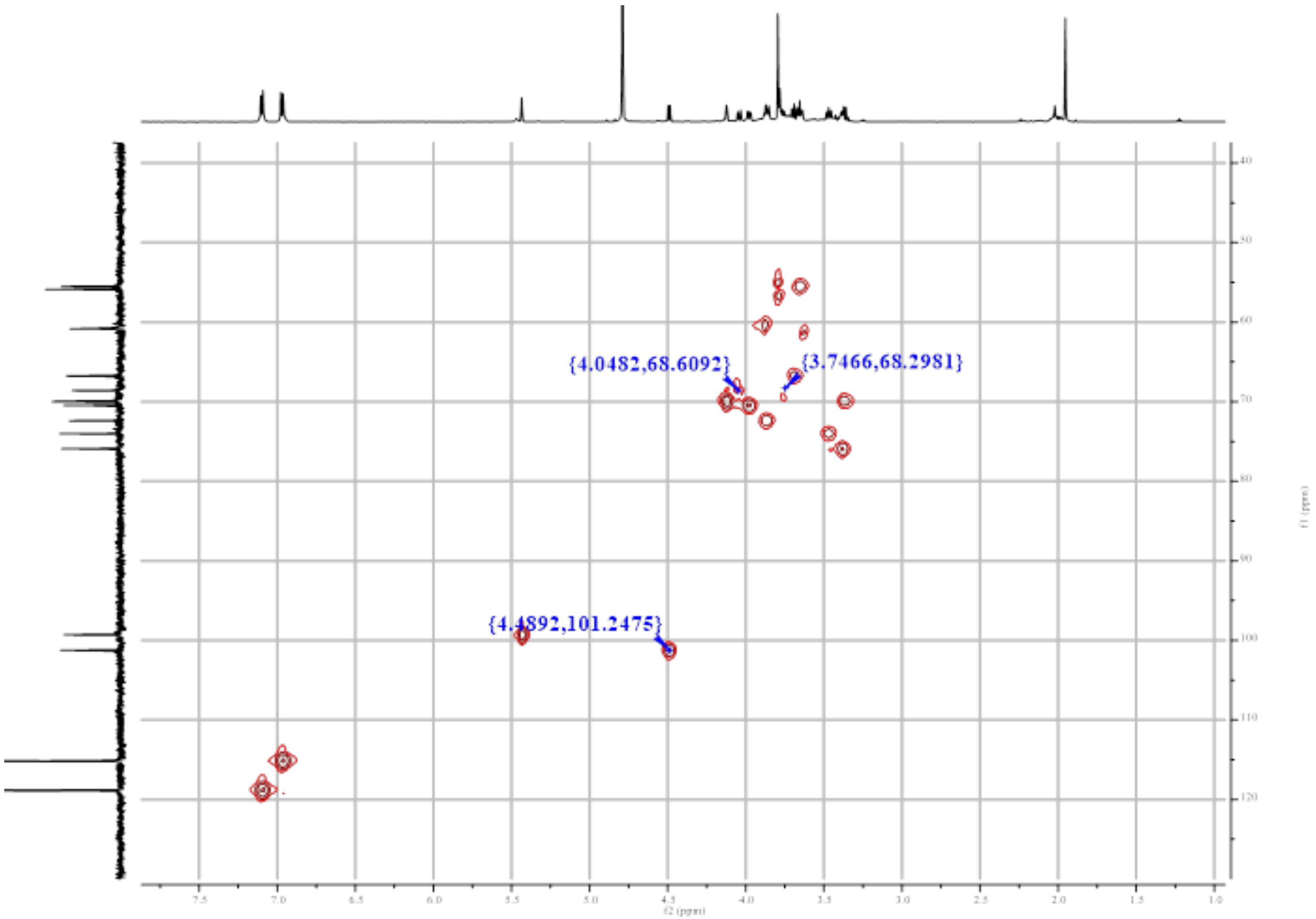

Compound **4d**, HMBC (600 MHz, D_2O)

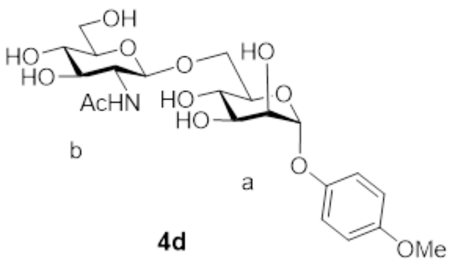

4d

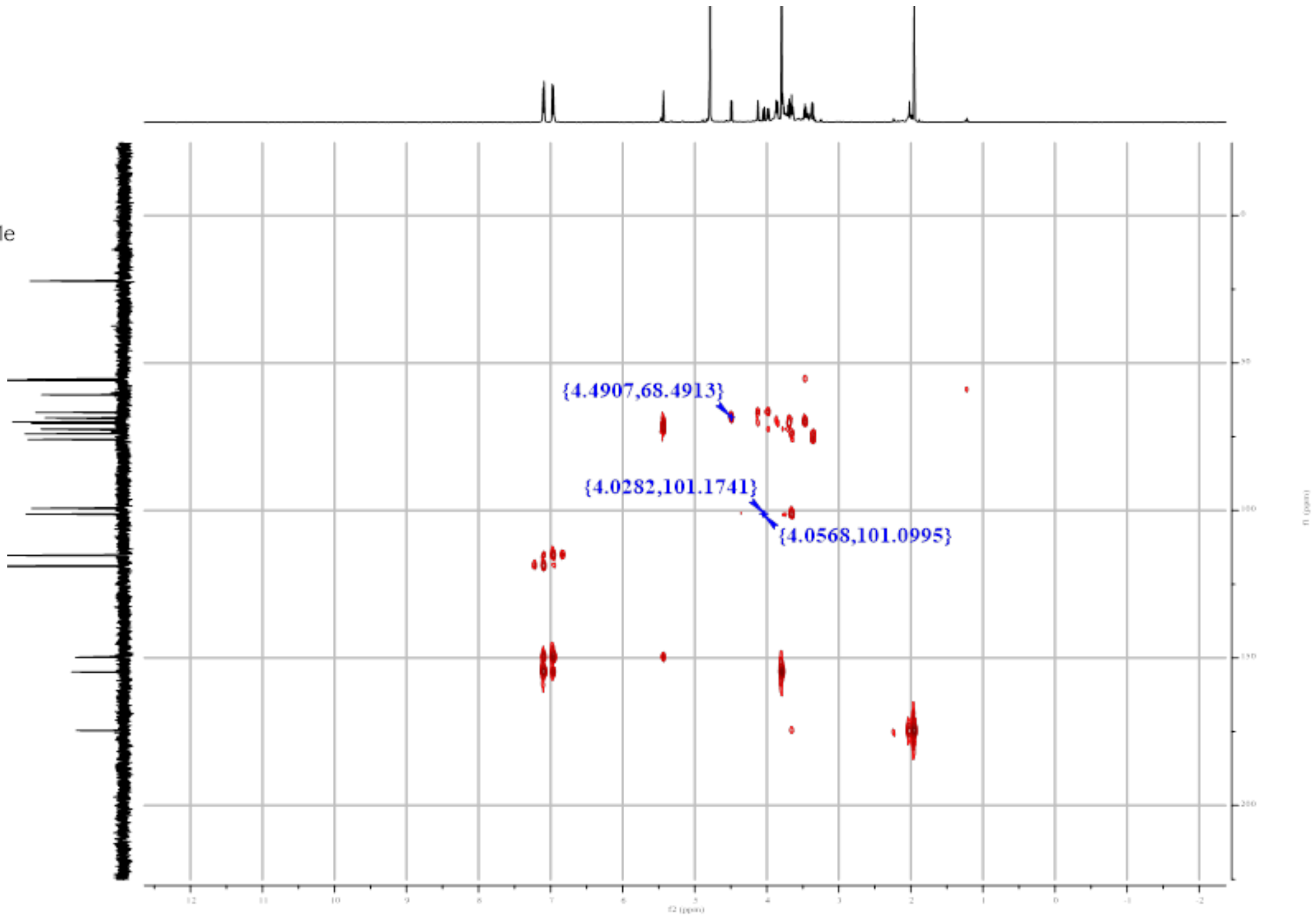

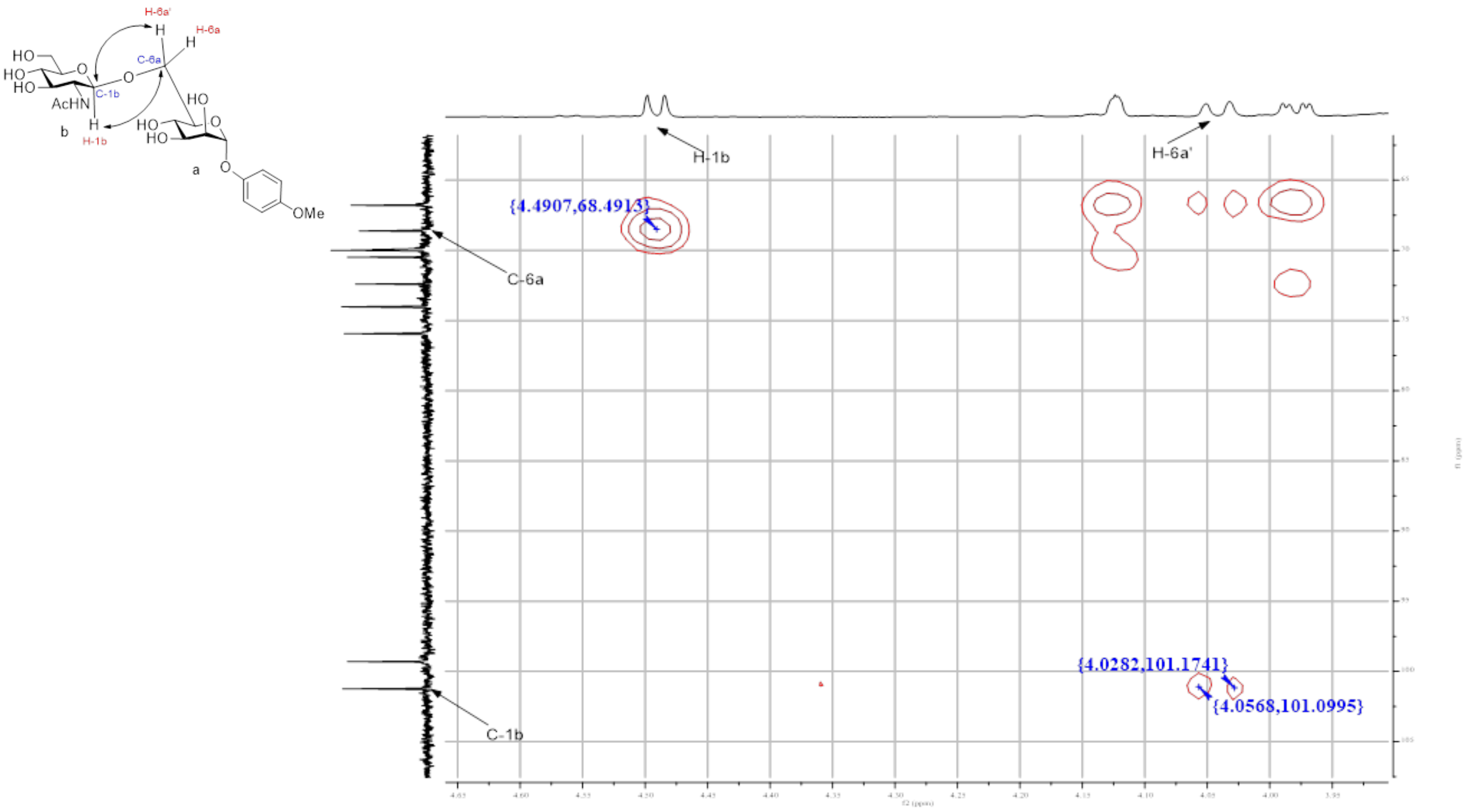
H-6a'
H-6a
C-6a
C-1b
AcHN
H-1b
OMe
H-1b
H-6a'
{4.4907,68.4913}
C-6a
{4.0282,101.1741}
{4.0568,101.0995}
C-1b

Acetyl 2-acetamido-2-deoxy-β-D-glucopyranosyl-(1→6)-α-D-mannopyranoside 4e, ^{1}H NMR (600 MHz, D_2O)

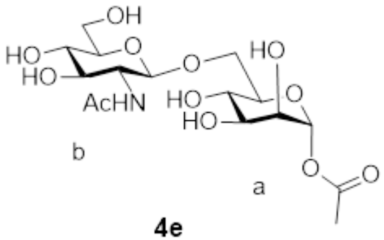

4e

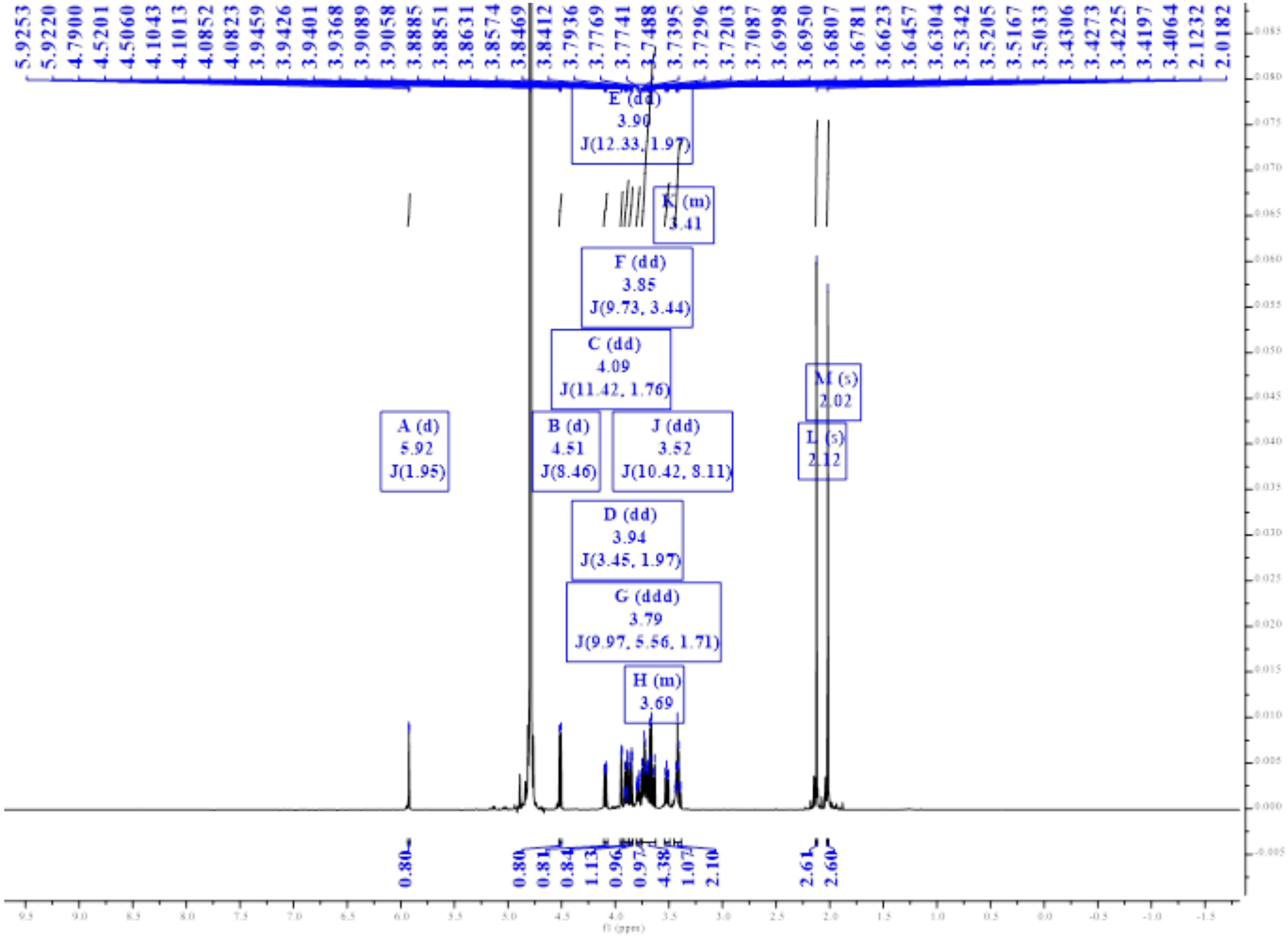

Compound **4e**, ^{13}C NMR (150 MHz, D_2O)

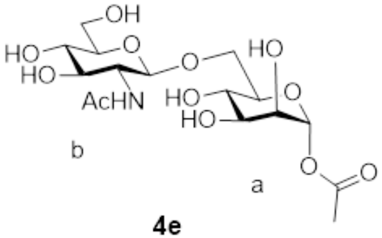

4e

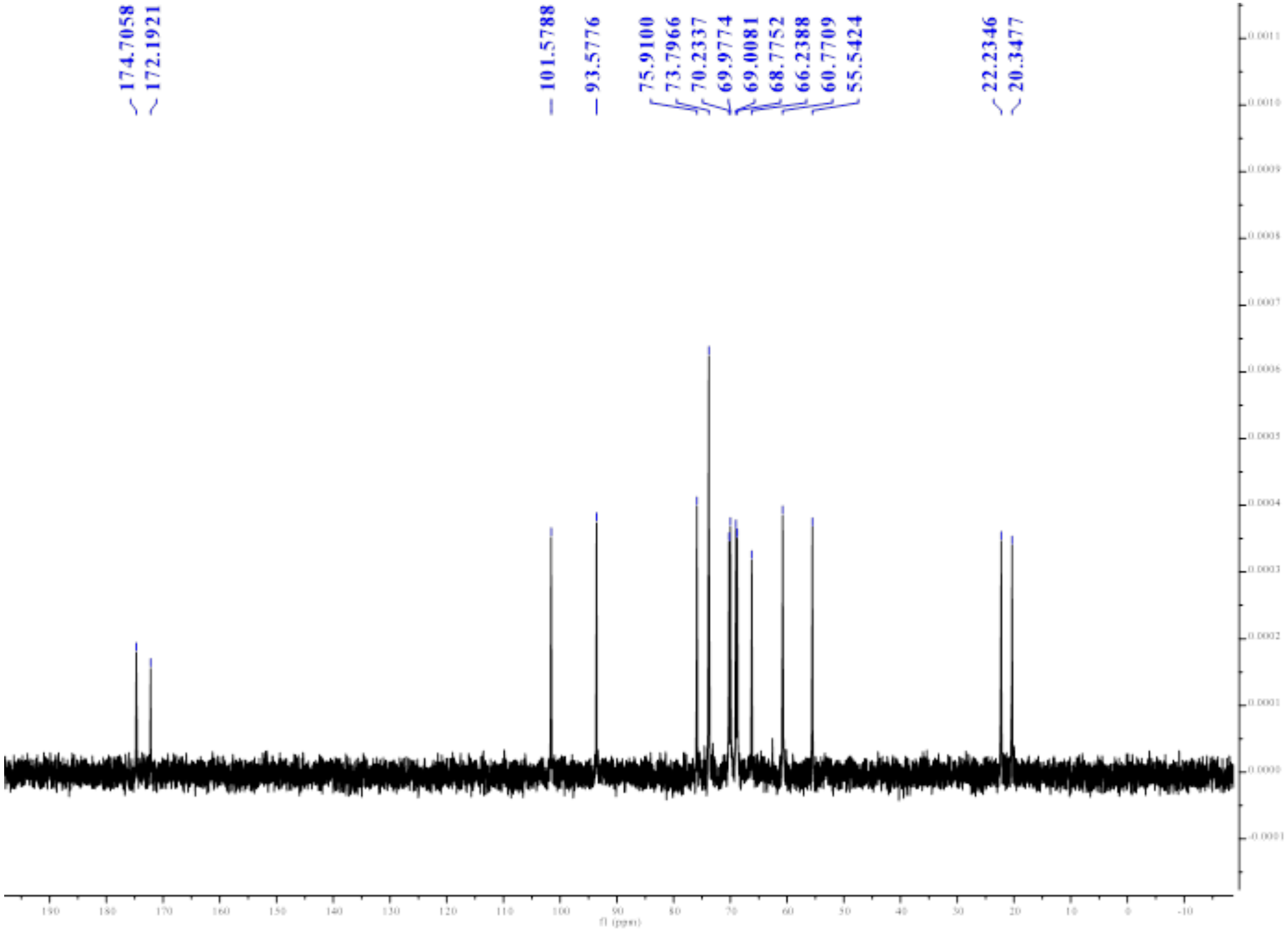

Compound **4e**, DEPT 135 (150 MHz, D_2O)

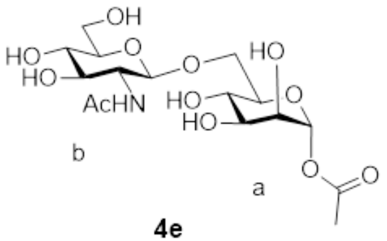

4e

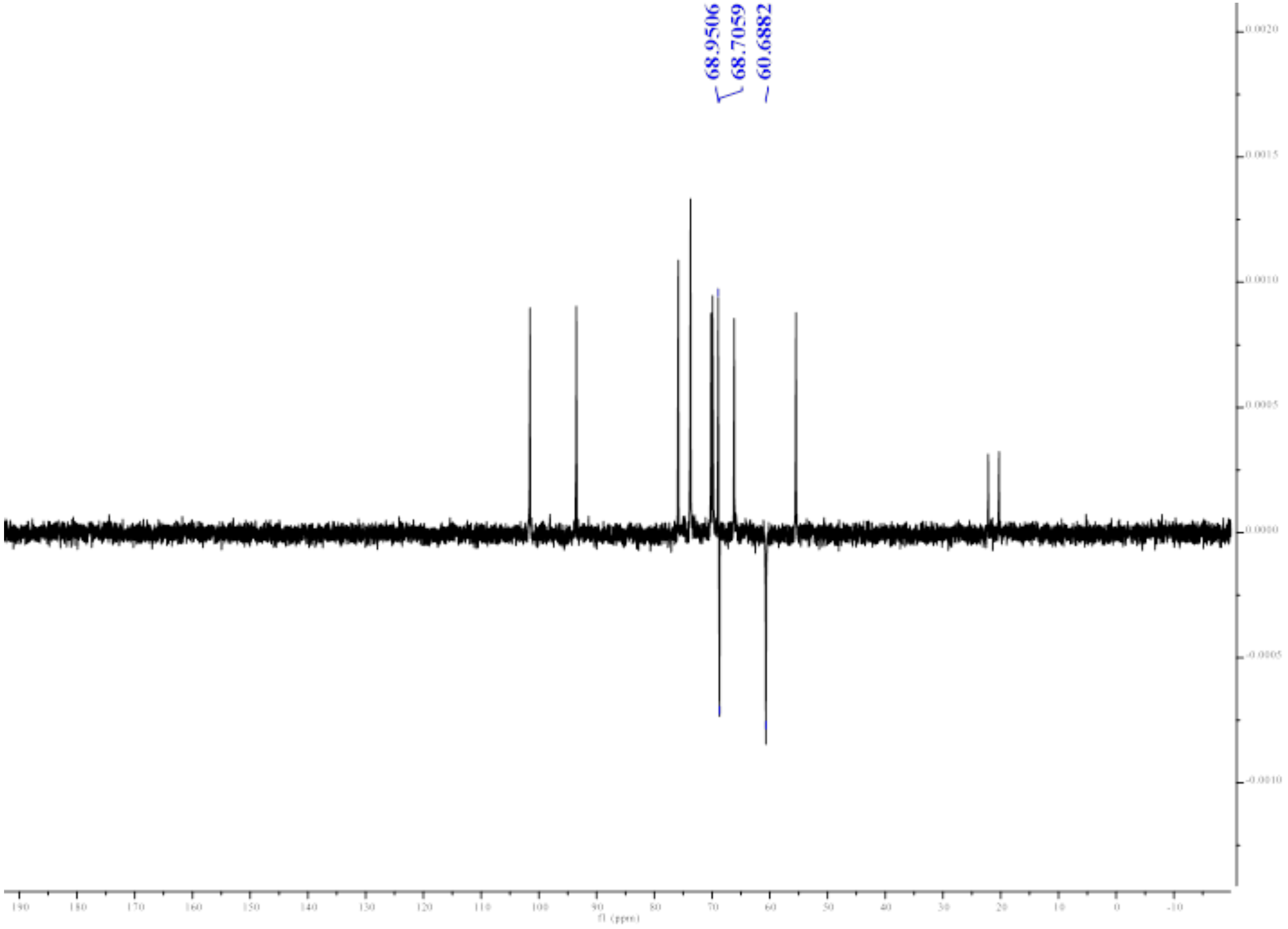

Compound **4e**, HMQC (600 MHz, D_2O)

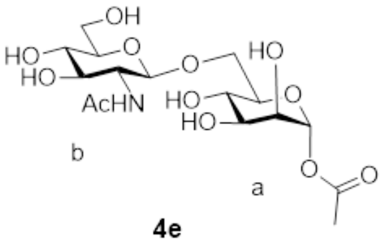

4e

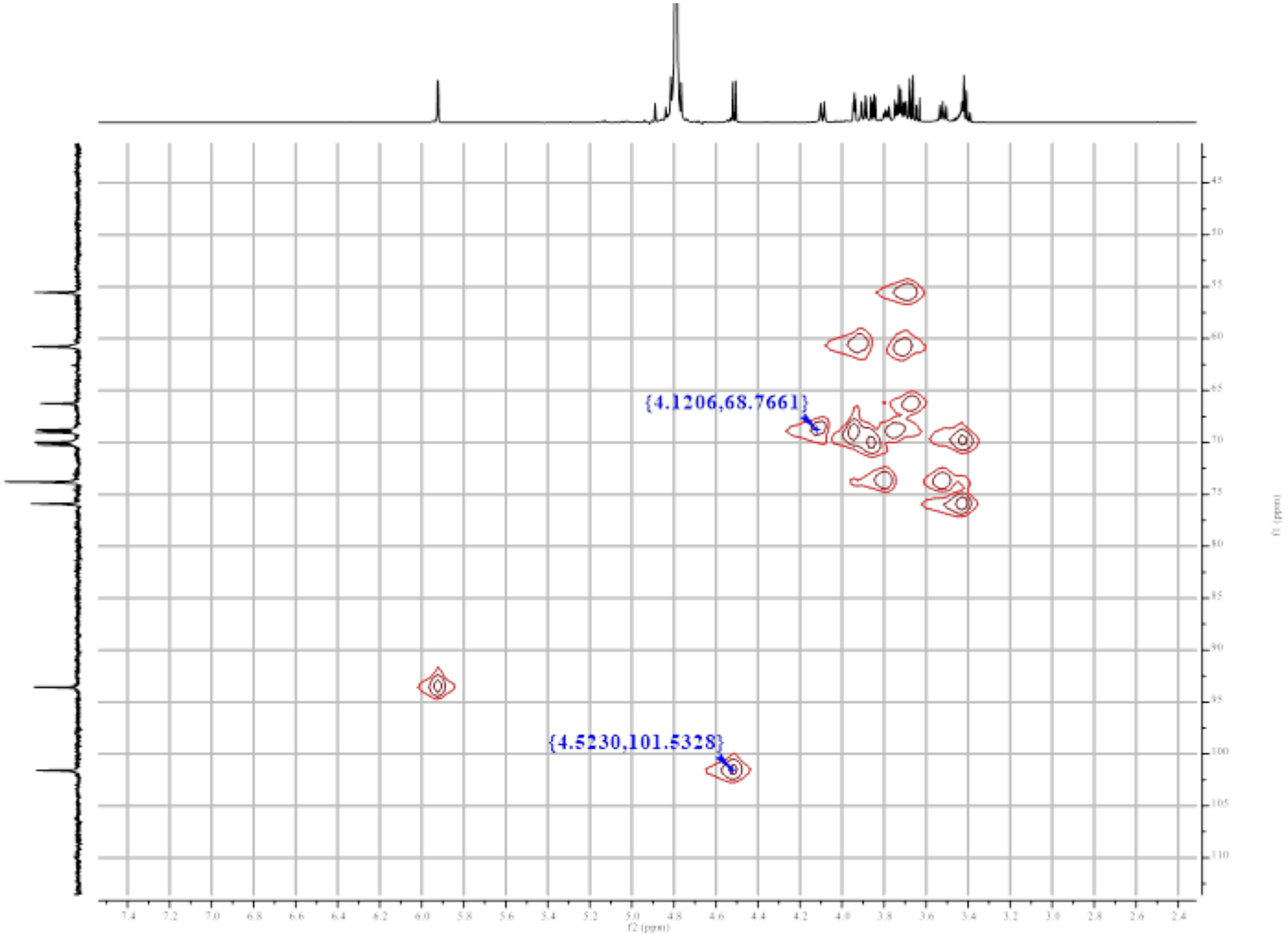

Compound **4e**, HMBC (600 MHz, D_2O)

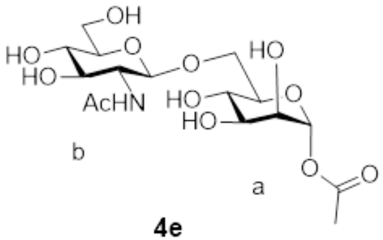

4e

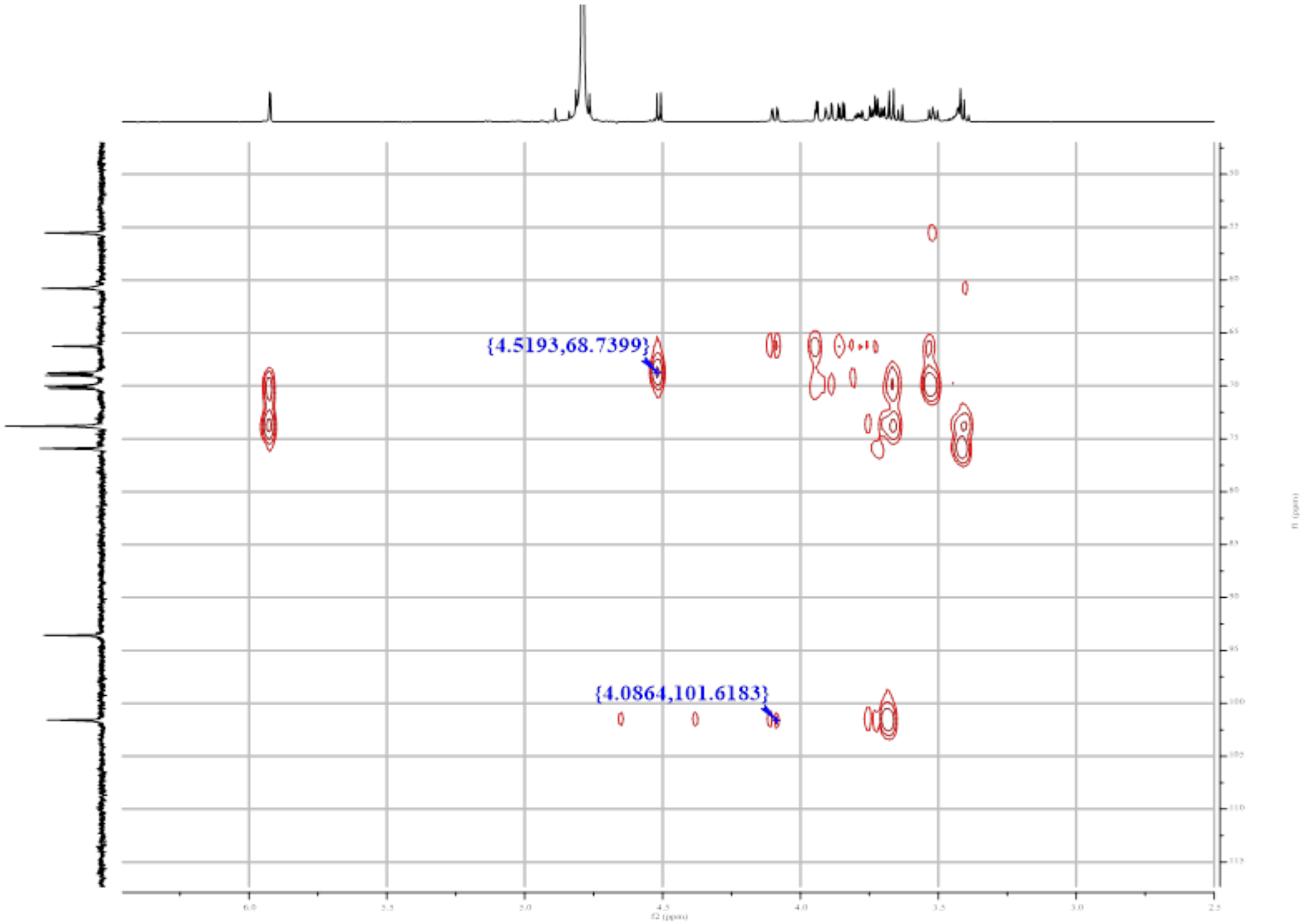

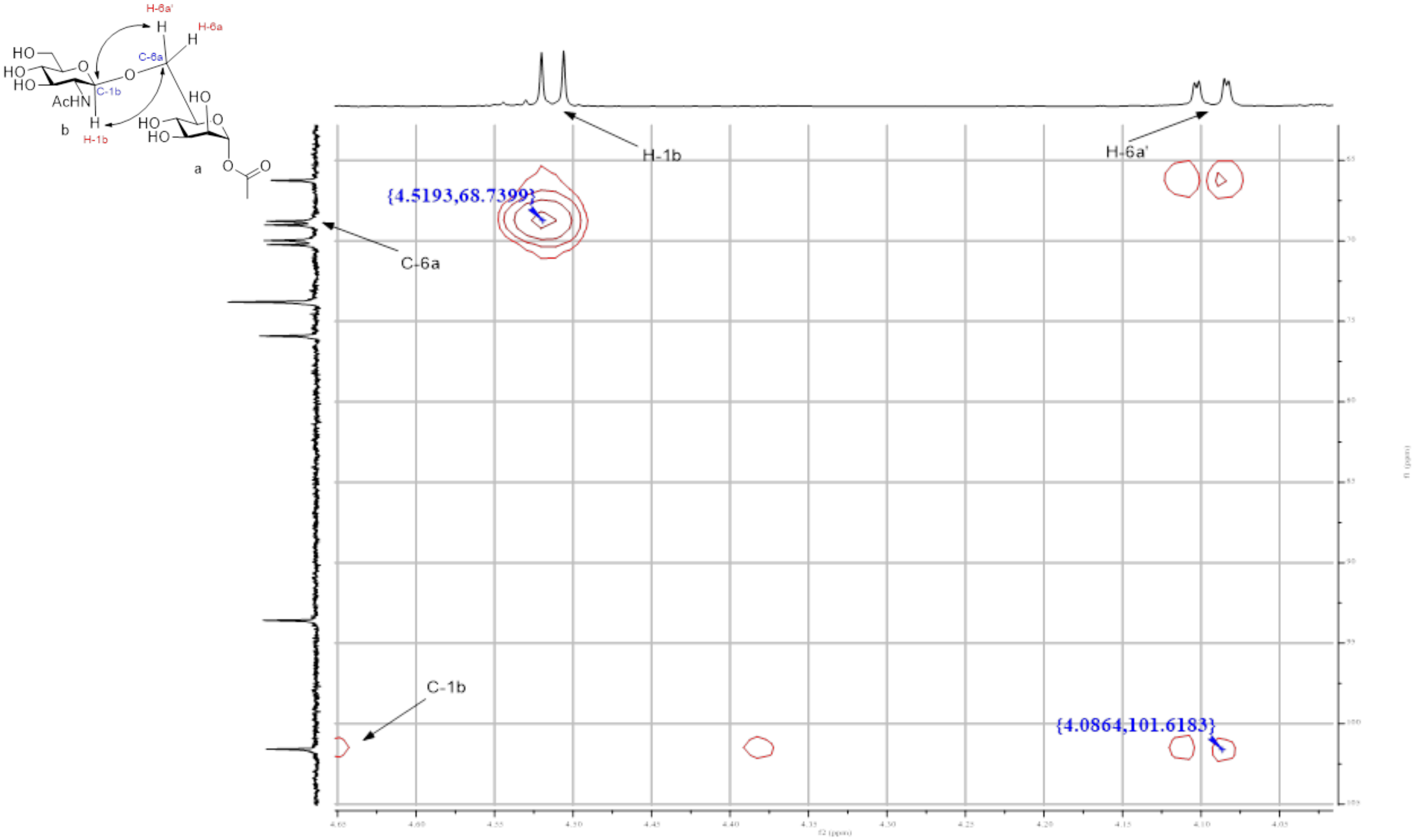

H-6a'
H-6a
C-6a
C-1b
AcHN
H-1b
b
a
{4.5193,68.7399}
H-1b
H-6a'
C-6a
C-1b
{4.0864,101.6183}

Fluoro 2-acetamido-2-deoxy-β-D-glucopyranosyl-(1→6)-α-D-mannopyranoside 4f, ^{1}H (400 MHz, D_2O)

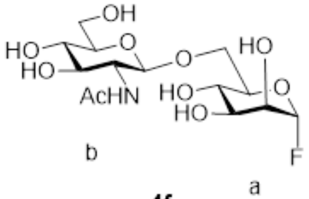

4f

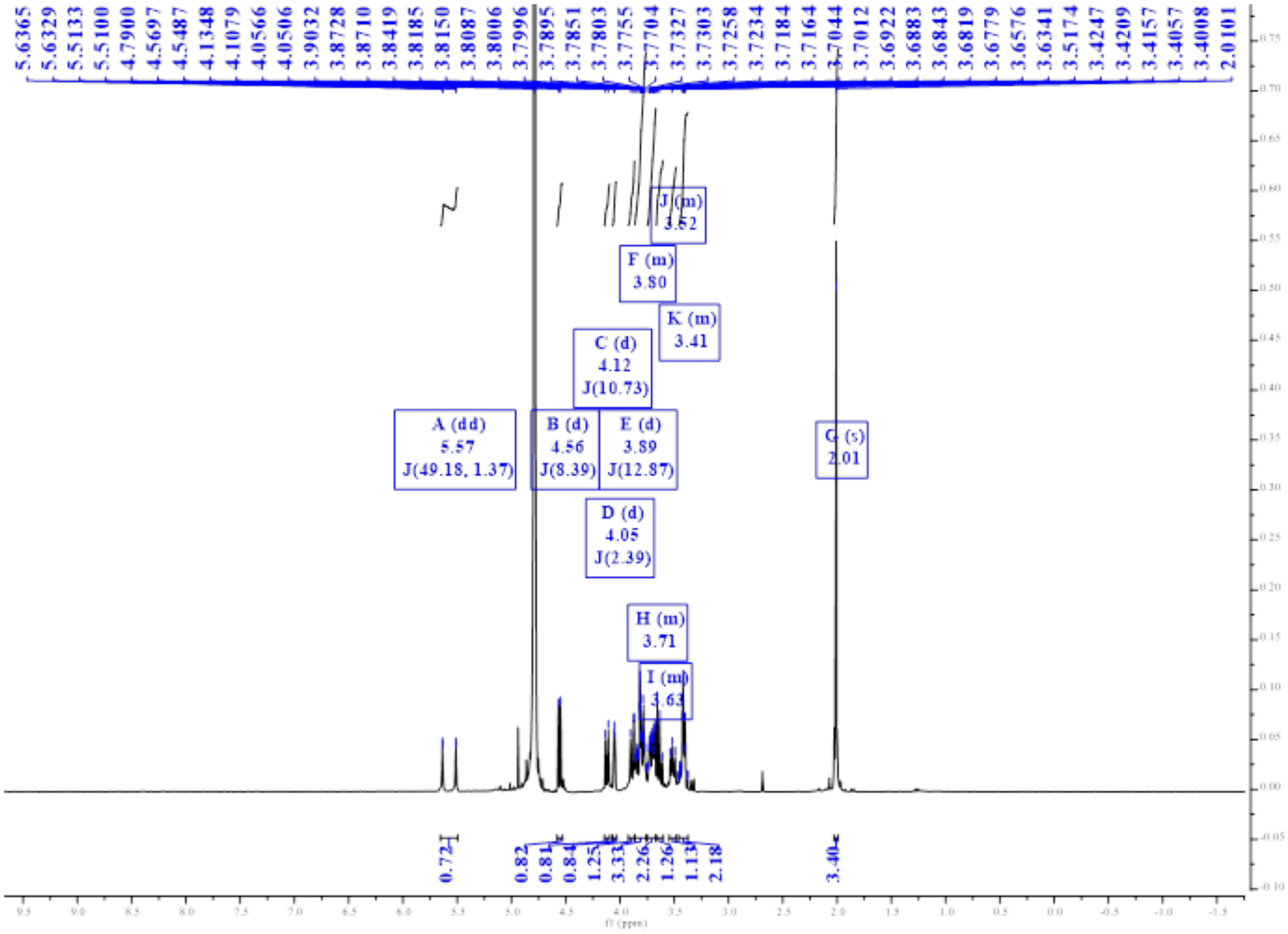

Compound **4f**, ^{13}C NMR (100 MHz, D_2O)

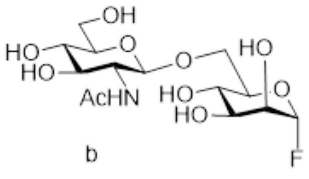

4f

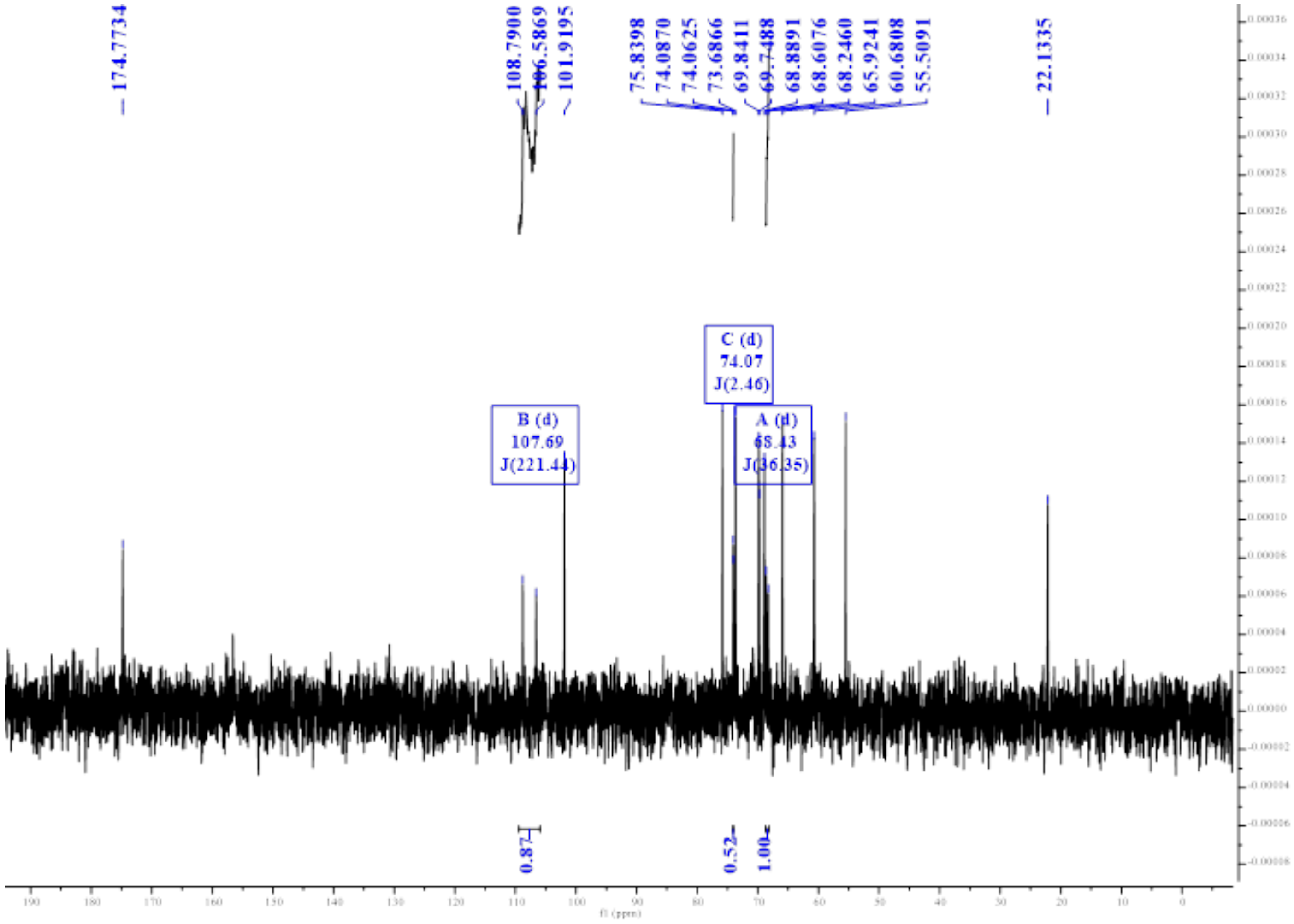

Compound **4f**, DEPT 135 (100 MHz, D_2O)

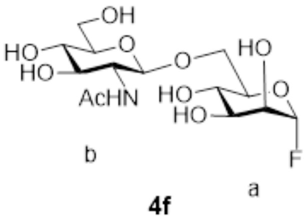

4f

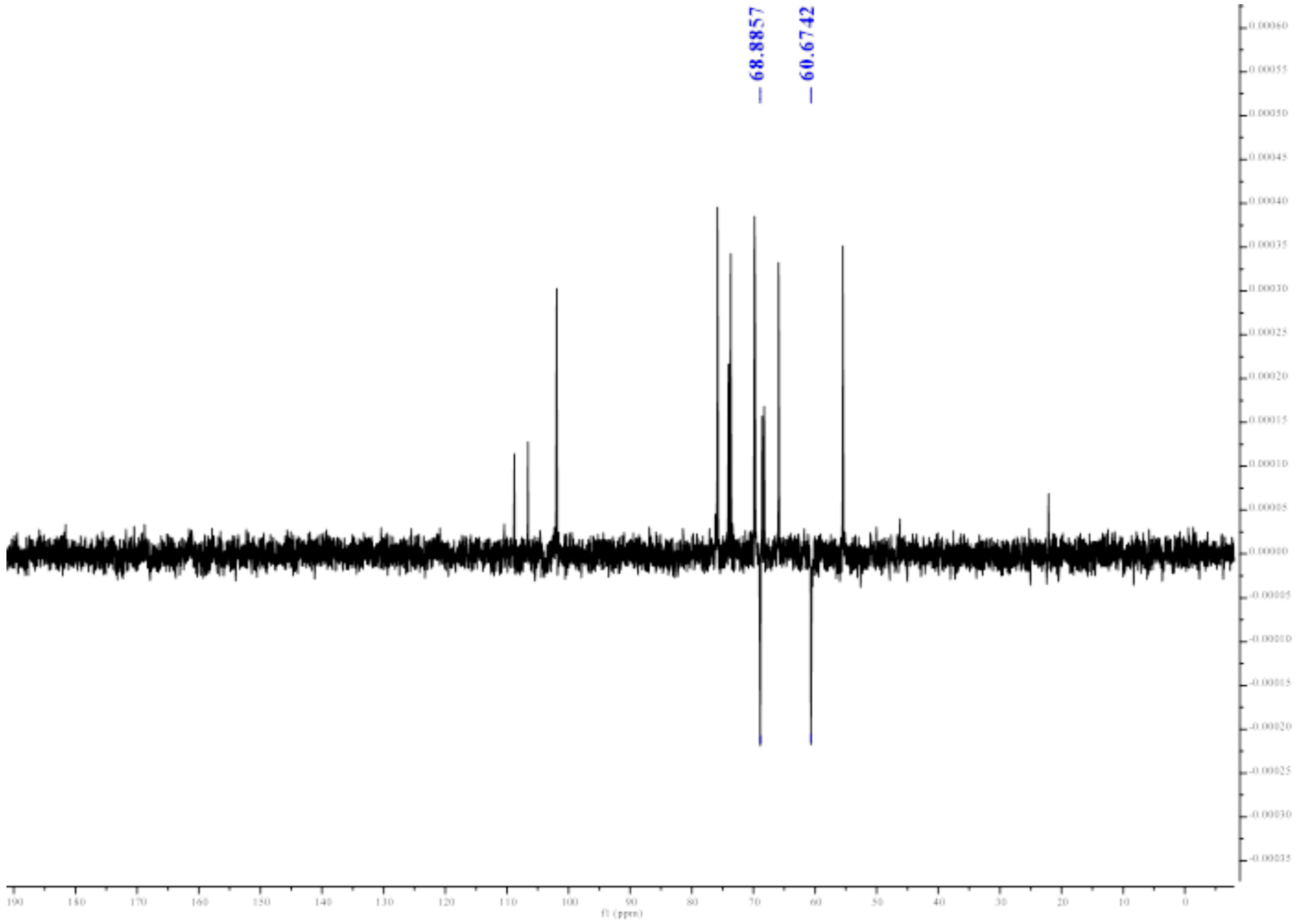

Compound **4f**, HMQC (600 MHz, D_2O)

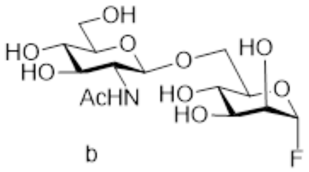

4f

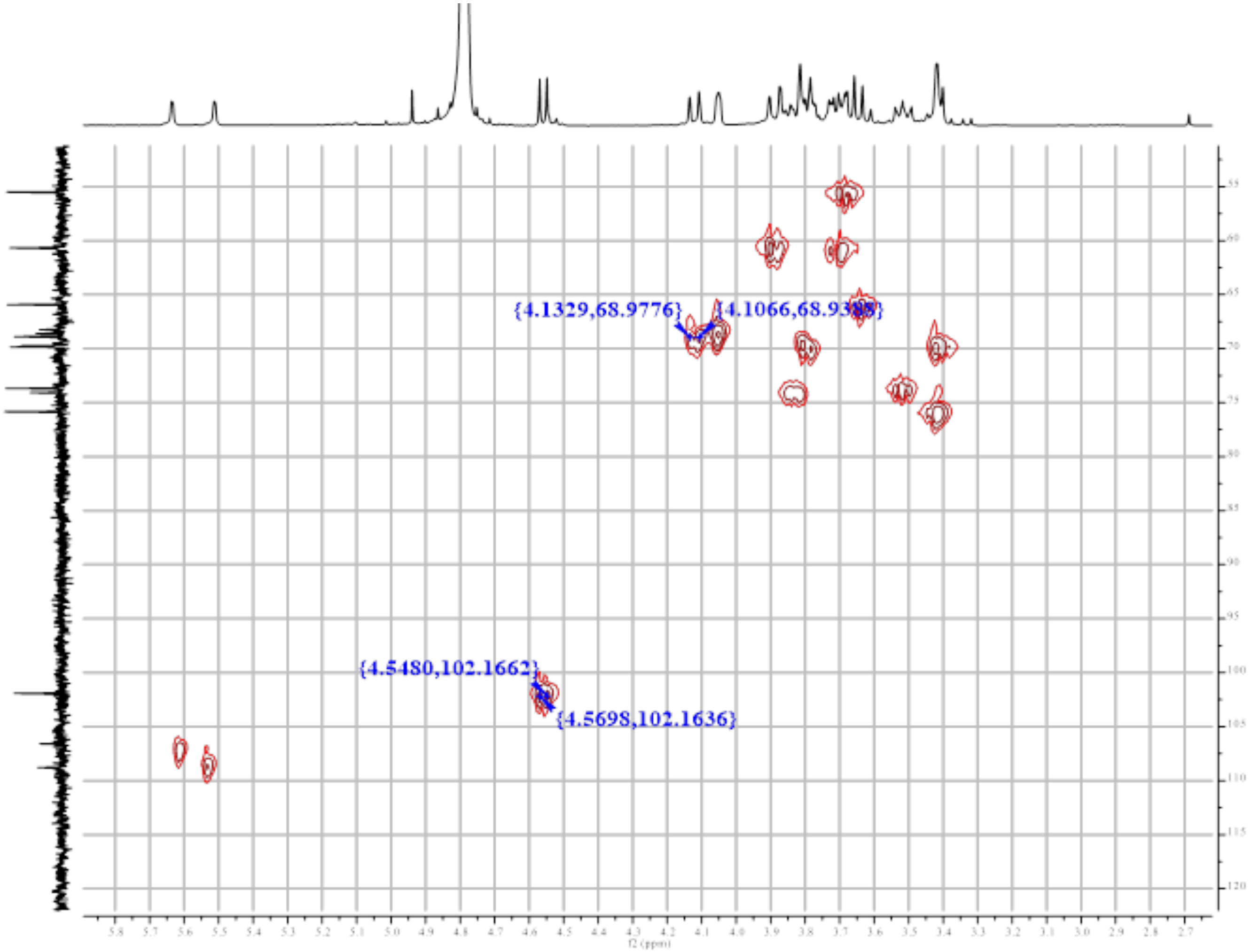

Compound **4f**, HMBC (600 MHz, D_2O)

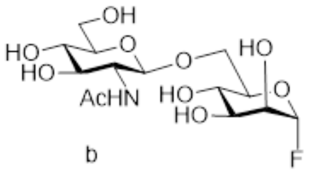

4f

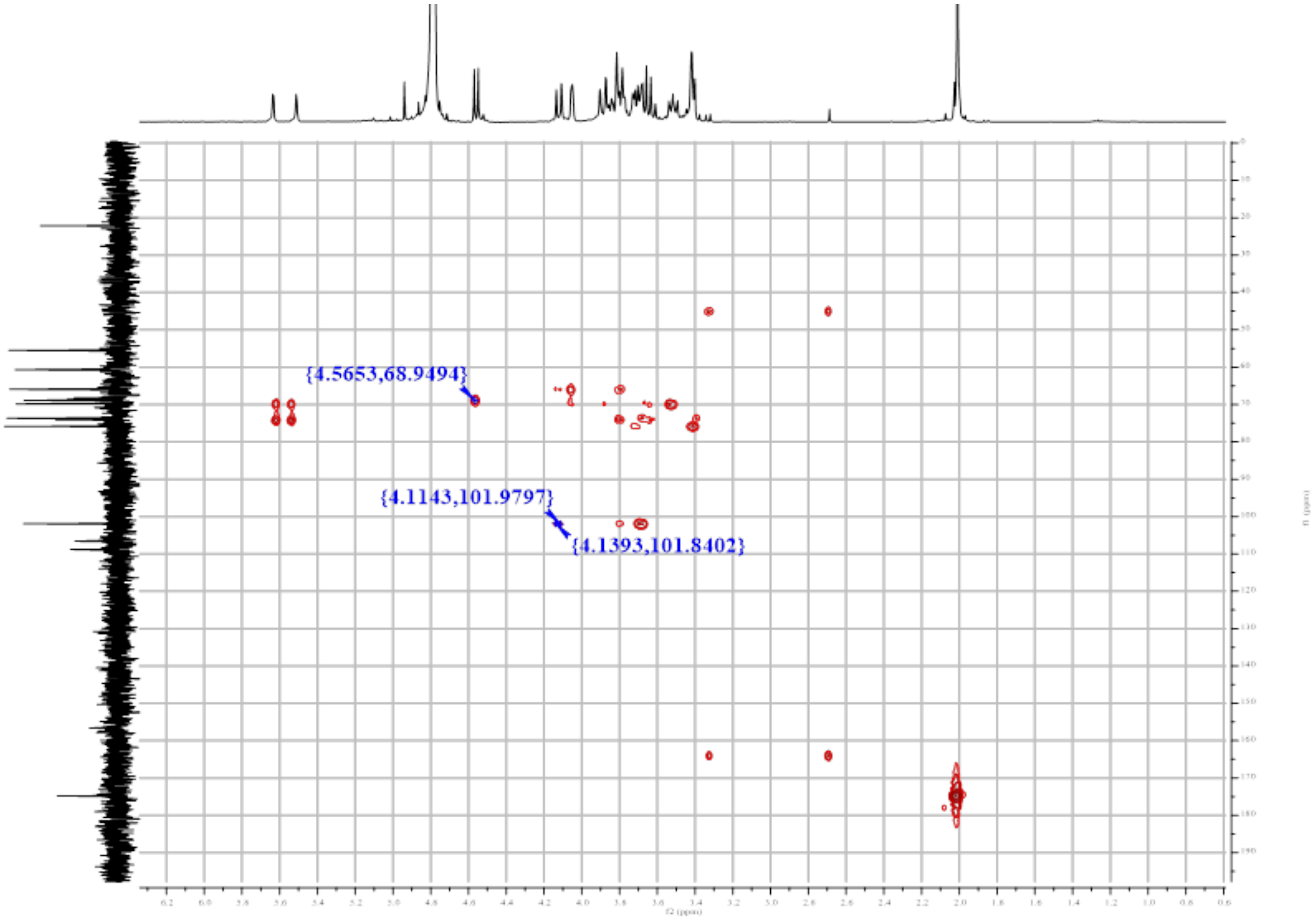

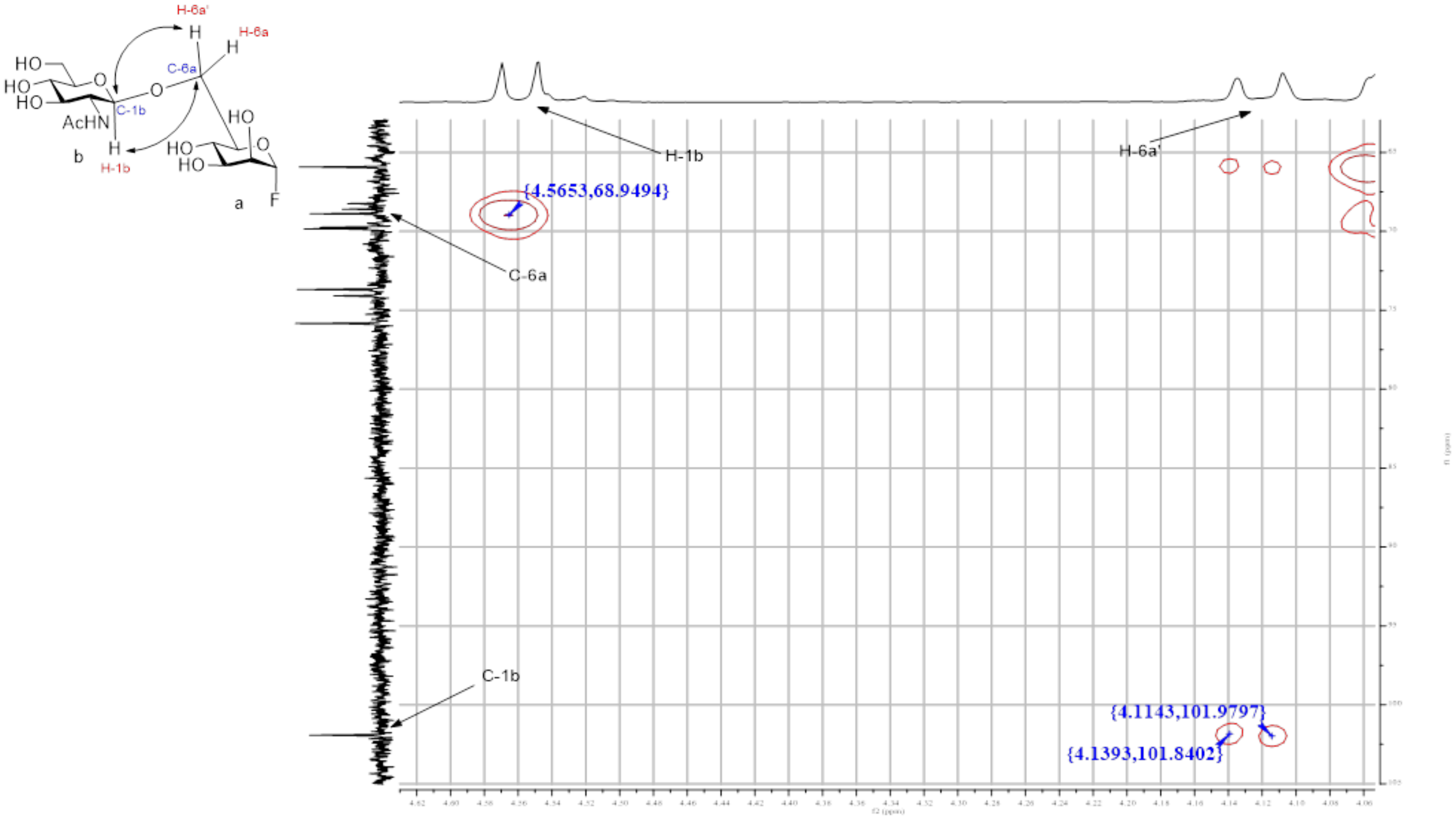
H-6a'
H-6a
C-6a
HO
HO
HO
O
O
C-1b
AcHN
H
b
H-1b
HO
HO
HO
O
a
F
H-1b
H-6a'
{4.5653,68.9494}
C-6a
C-1b
{4.1143,101.9797}
{4.1393,101.8402}

***p*-Nitrophenyl 2-acetamido-2-deoxy-β-D-glucopyranosyl-(1→6)-β-D-galctopyranoside 4g**, ^{1}H NMR (600 MHz, DMSO-d6)

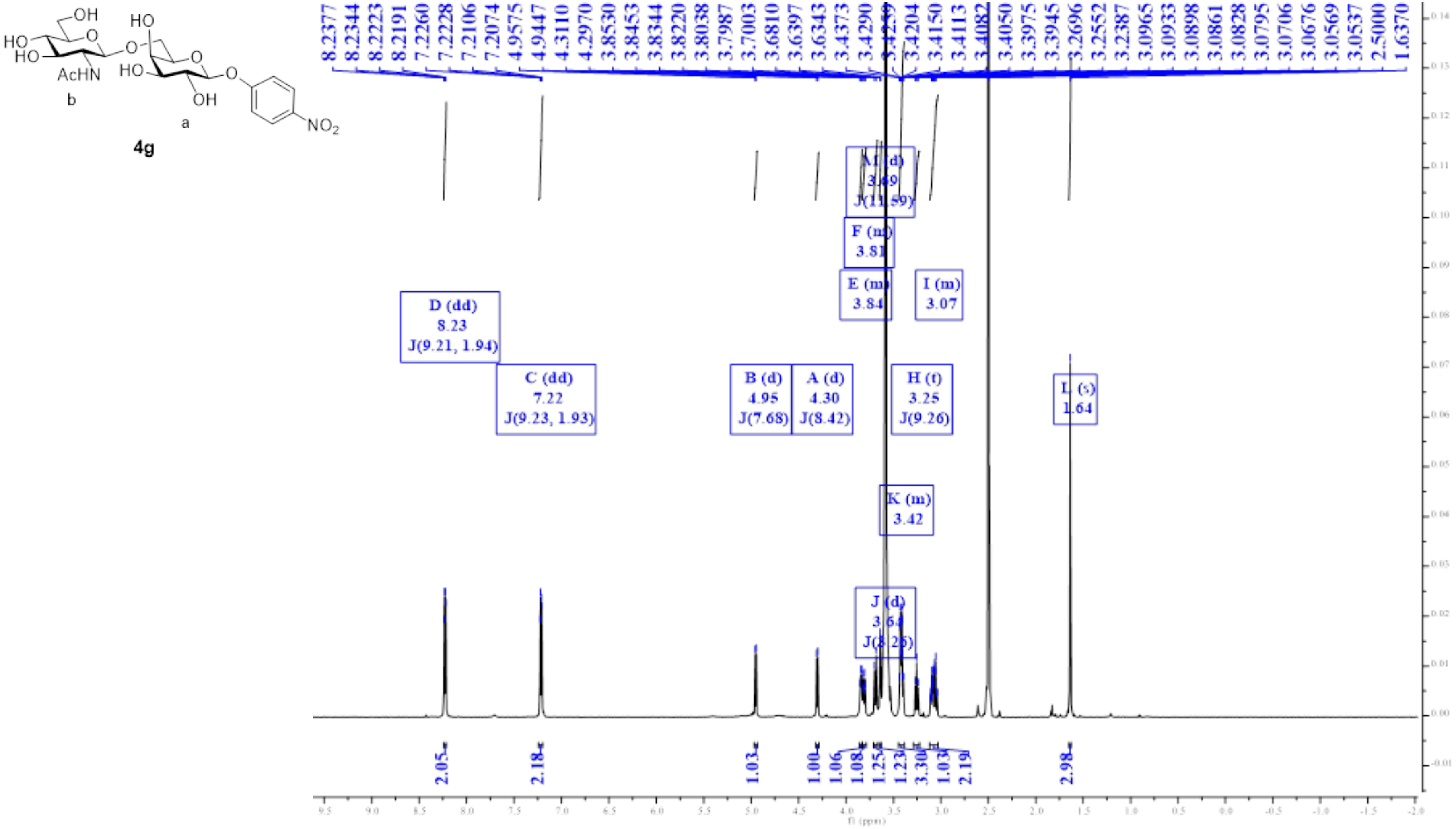

Compound **4g**, C[13] NMR (150 MHz, DMSO-d6)

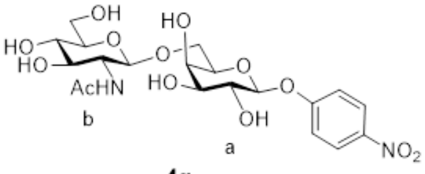

4g

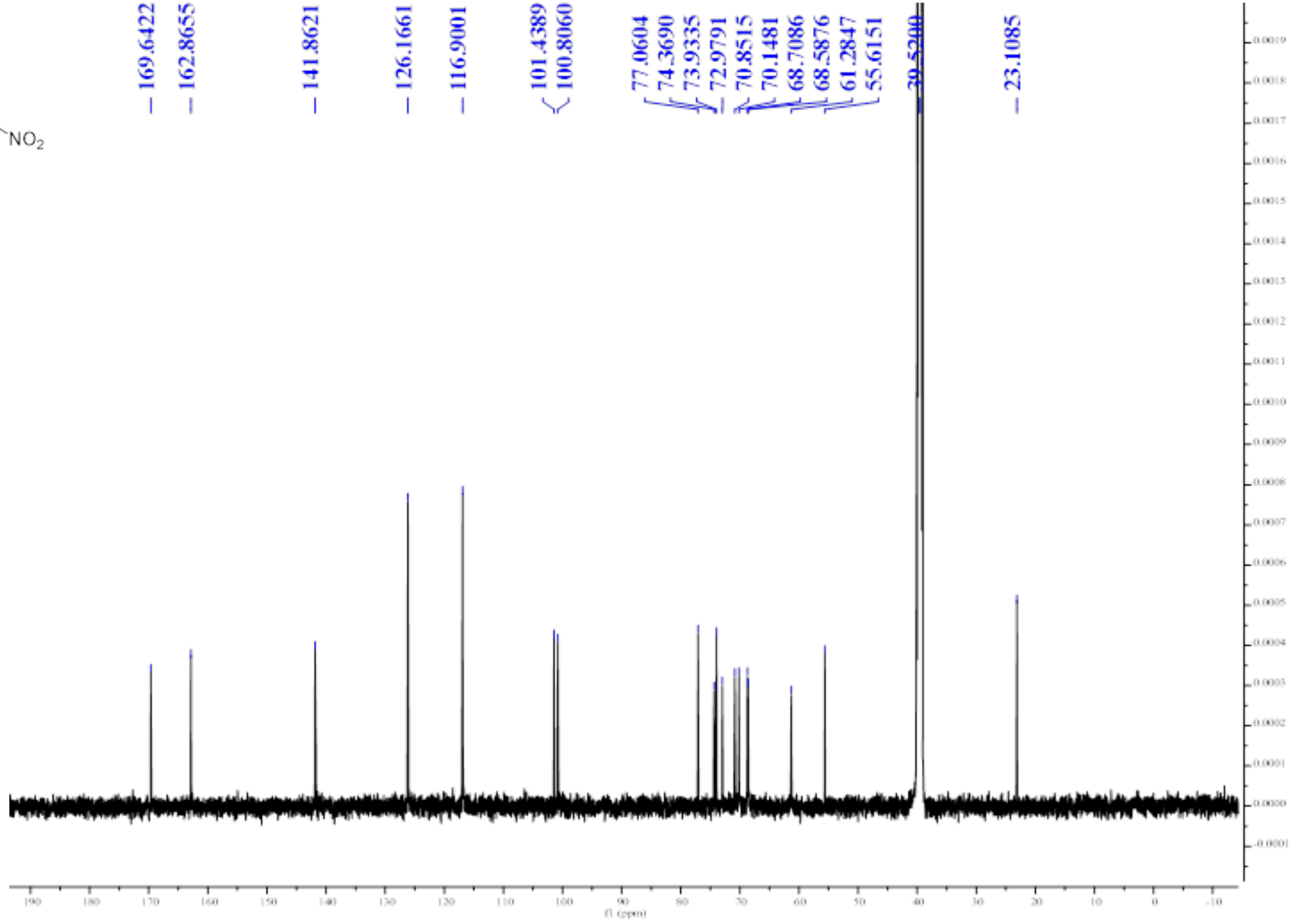

Compound **4g**, DEPT135 (100 MHz, DMSO-d6)

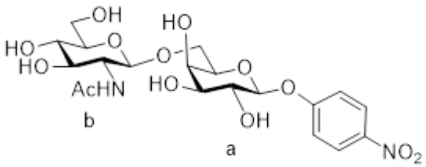

4g

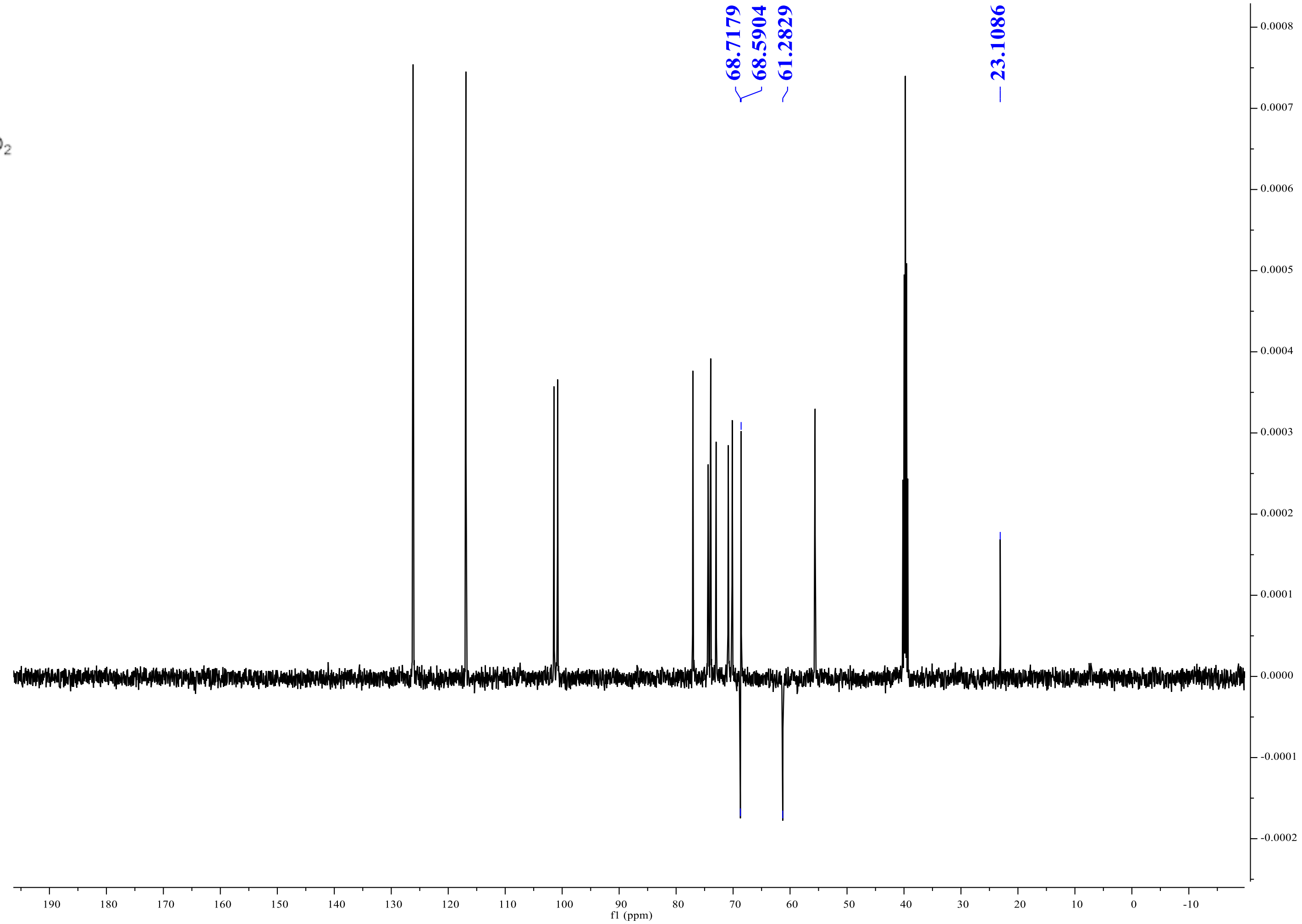

Compound **4g**, HMQC (600 MHz, DMSO-d6)

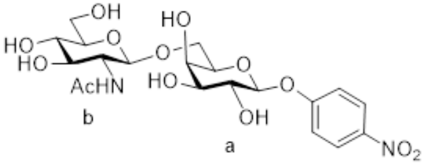

4g

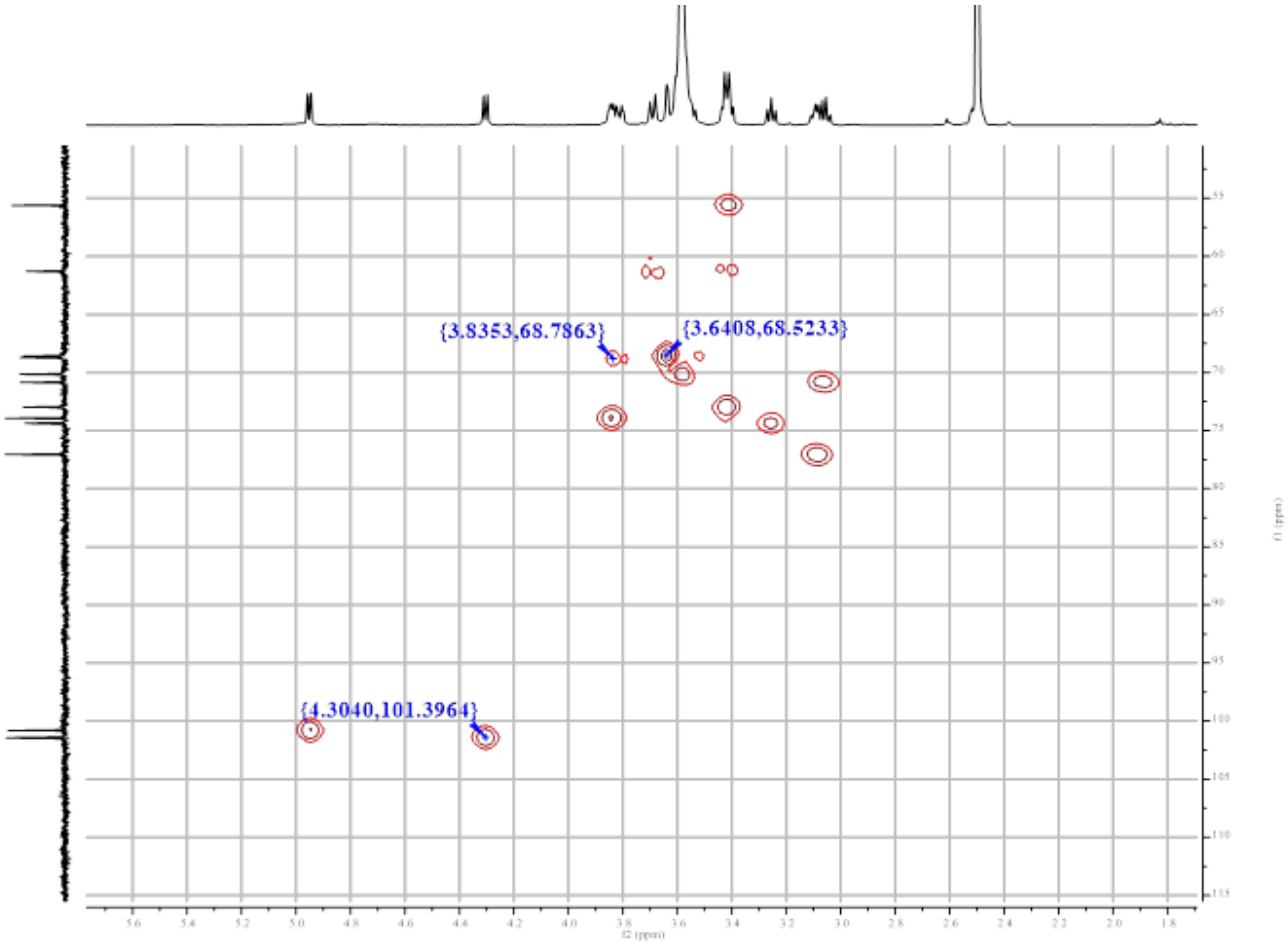

Compound **4g**, HMBC (600 MHz, DMSO-d6)

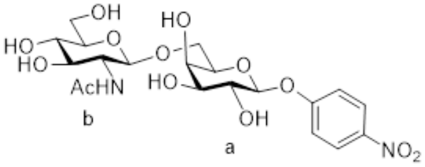

4g

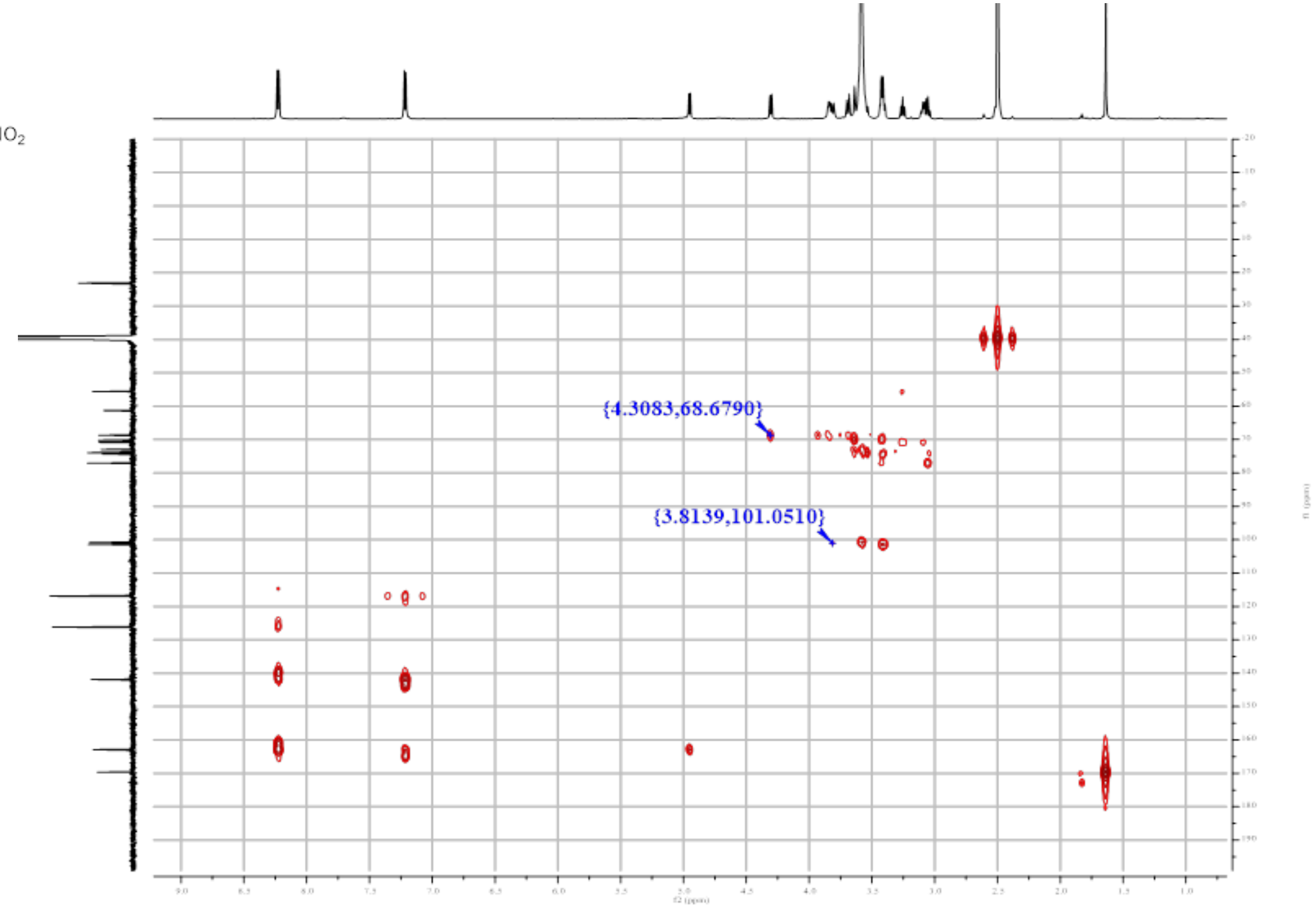

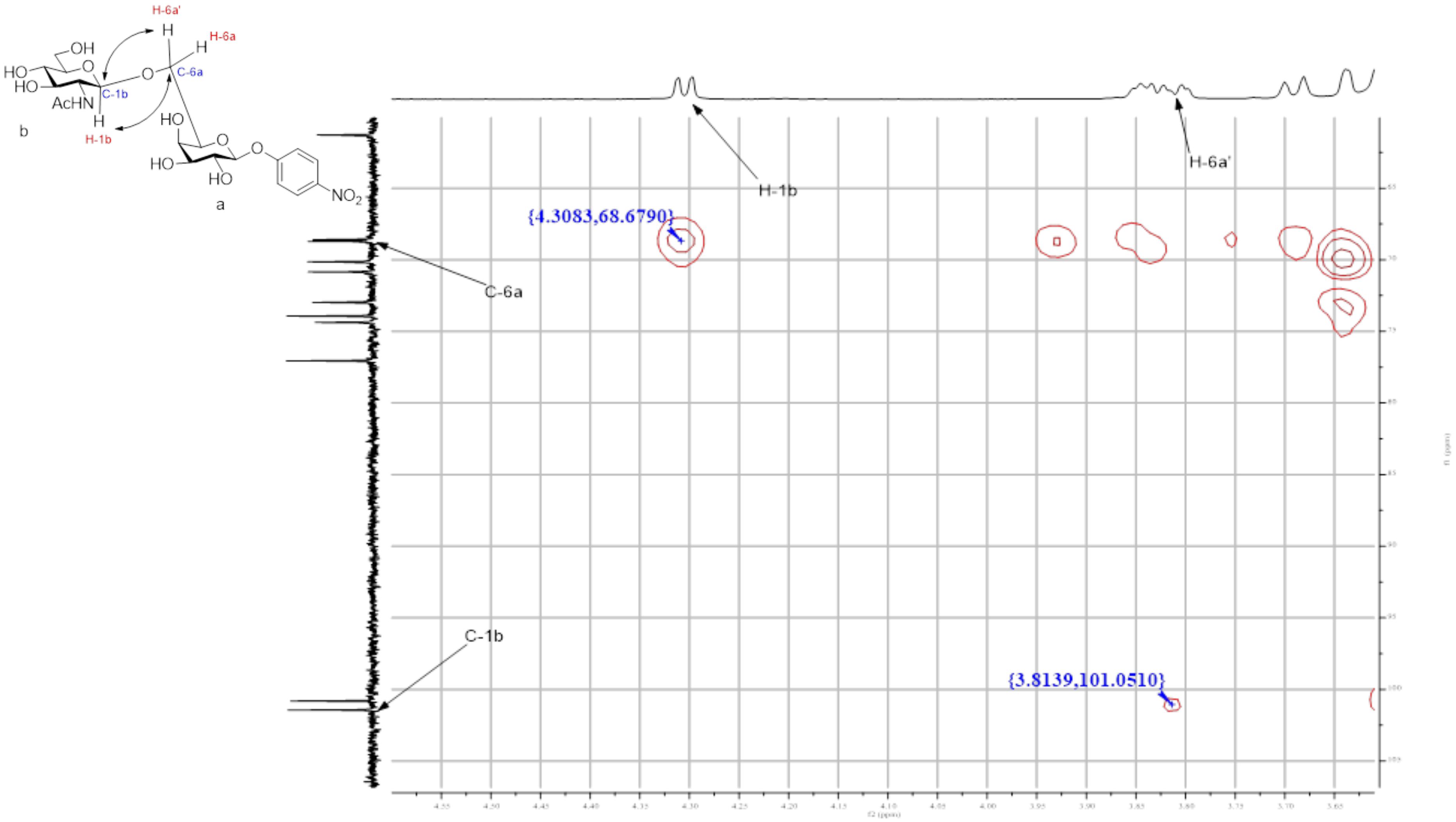
H-6a'
H-6a
C-6a
C-1b
H-1b
AcHN
NO2
a
b
H-1b
H-6a'
{4.3083,68.6790}
C-6a
C-1b
{3.8139,101.0510}
f2 (ppm)
f1 (ppm)

***p*-Nitrophenyl 2-acetamido-2-deoxy-β-D-glucopyranosyl-(1→6)-2-acetamido-2-deoxy-β-D-glucopyranoside 4h**, ^{1}H NMR (600 MHz, D_2O)

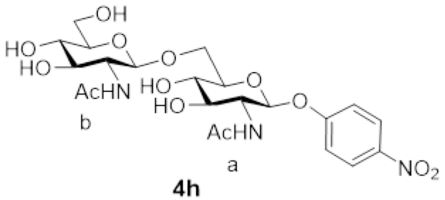

4h

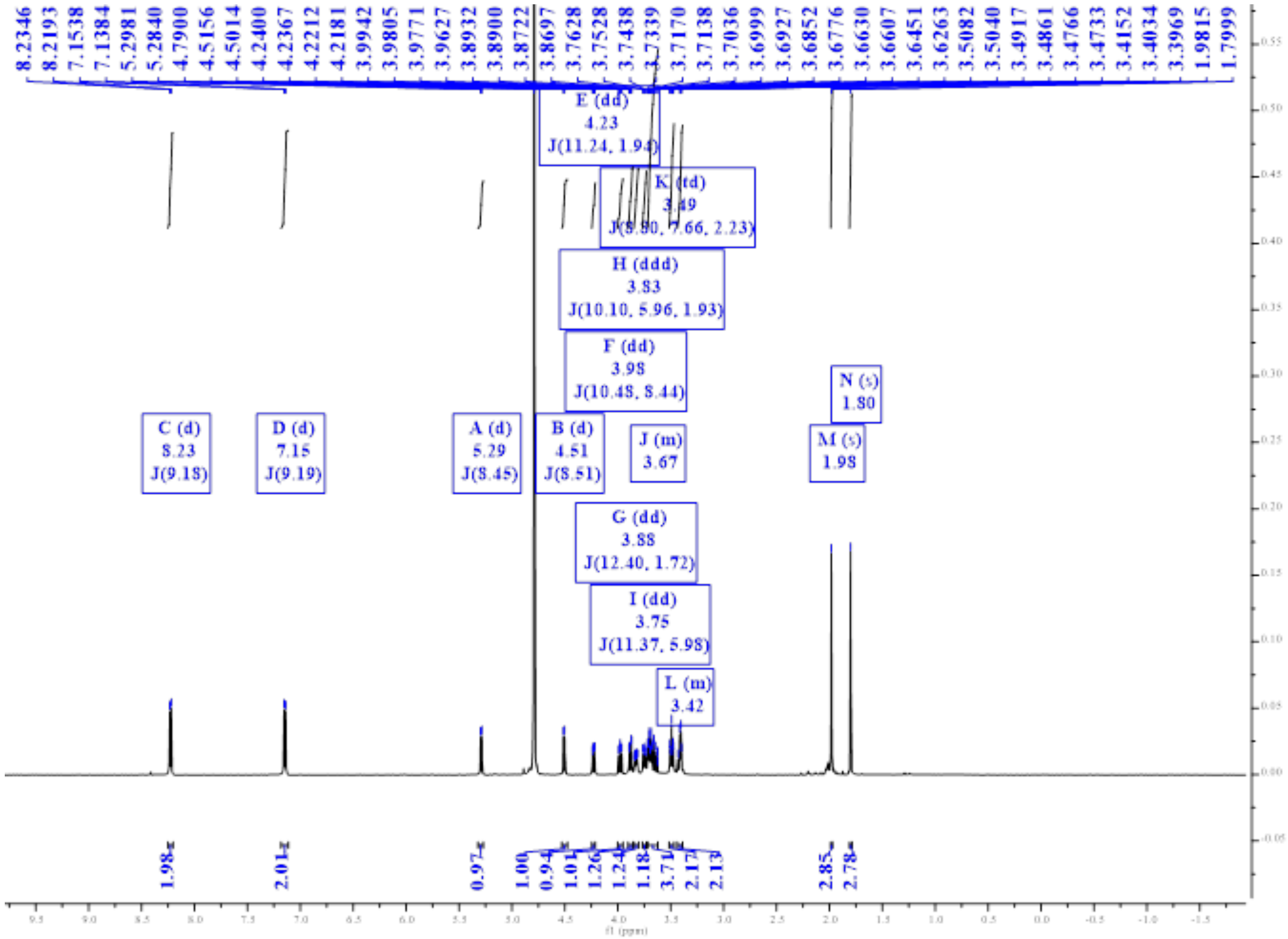

Compound **4h**, ^{13}C NMR (150 MHz, D_2O)

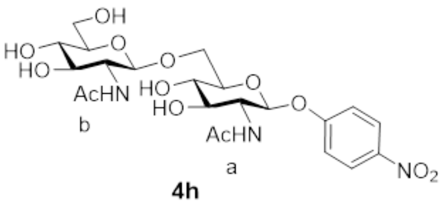

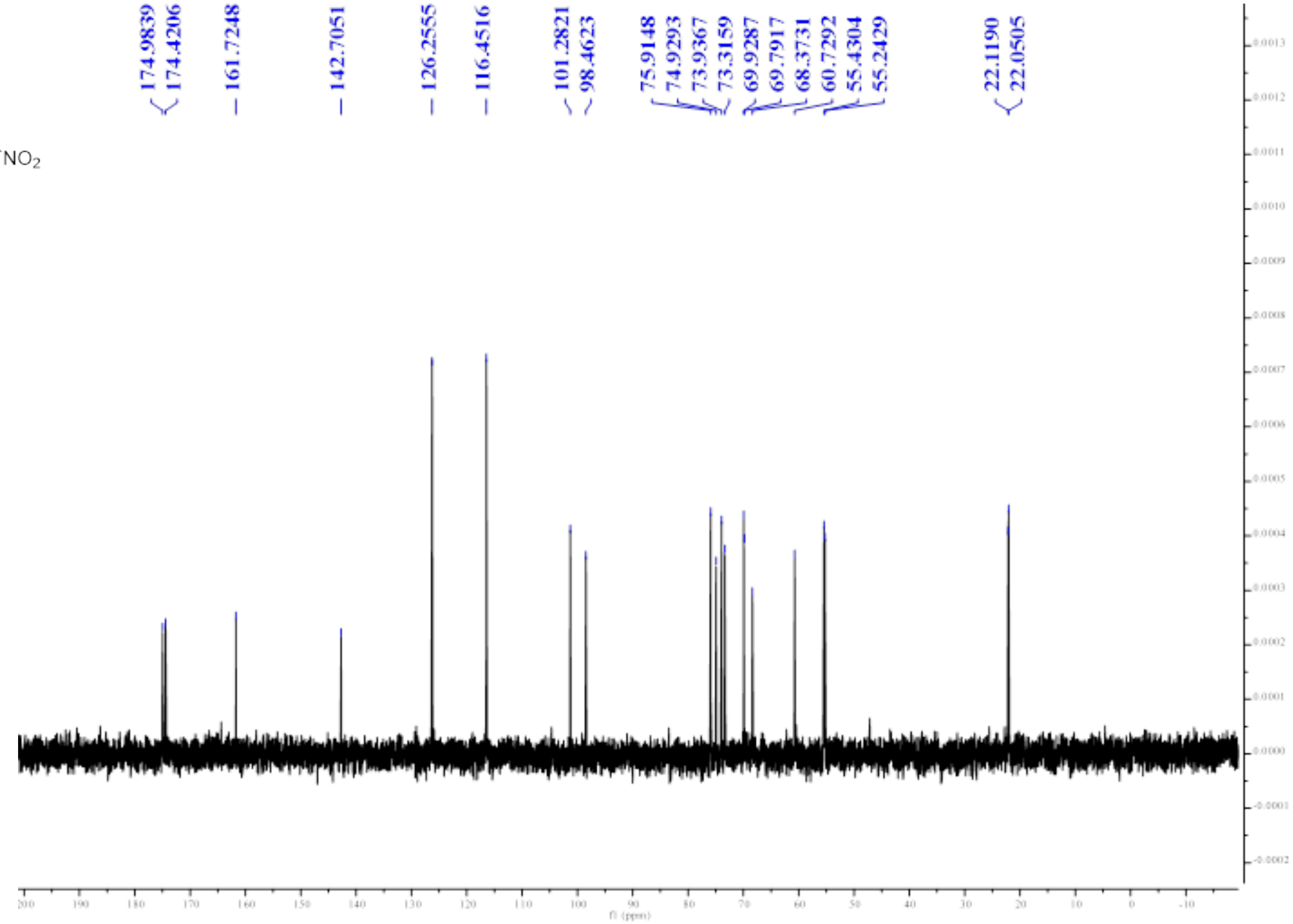

Compound **4h**, DEPT 135 (150 MHz, D_2O)

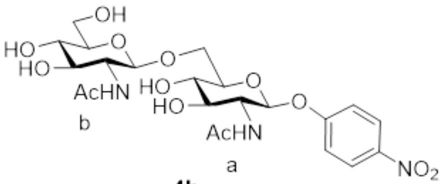

4h

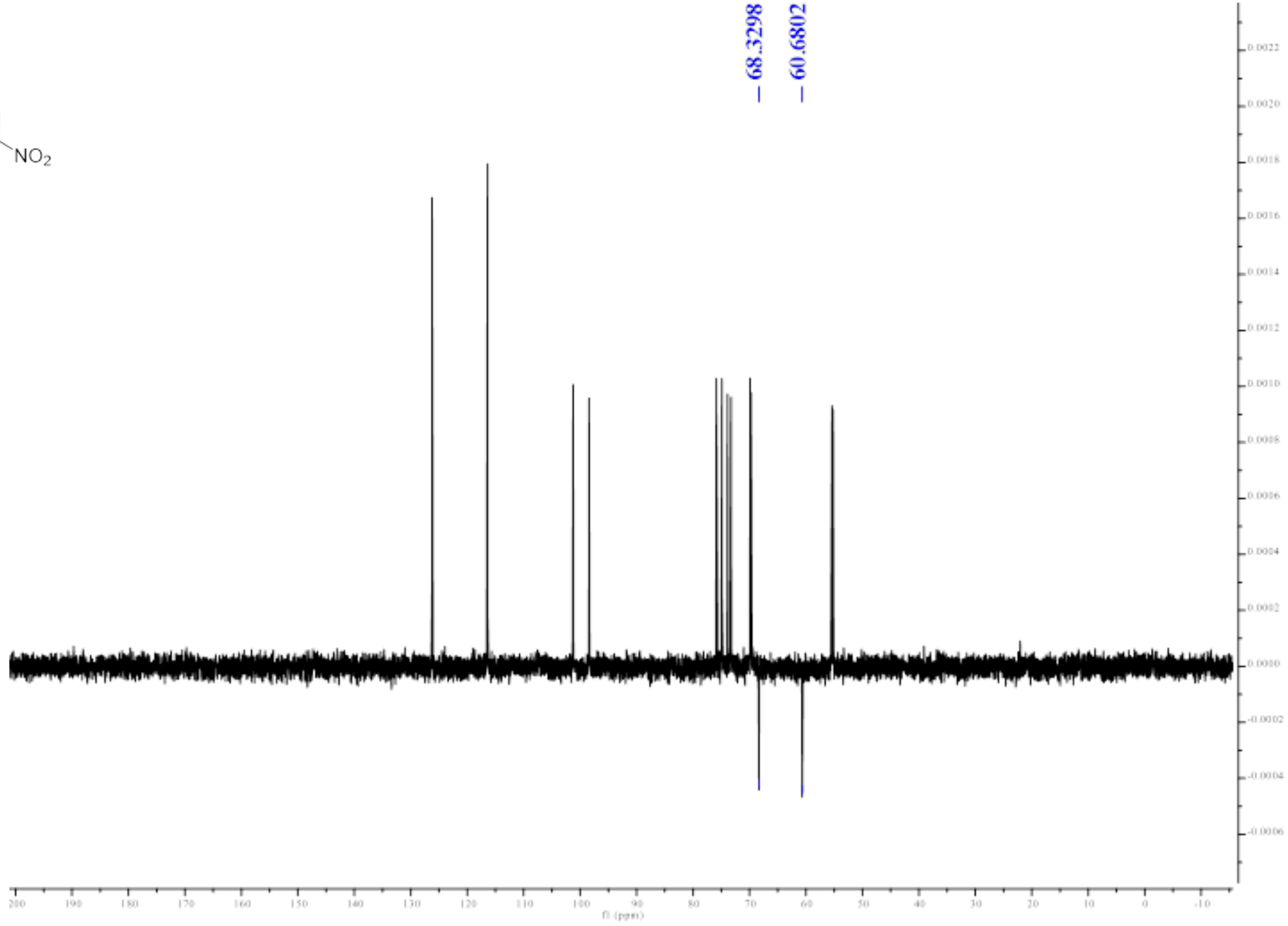

Compound **4h**, HMQC (600 MHz, D_2O)

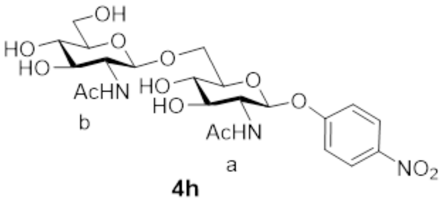

4h

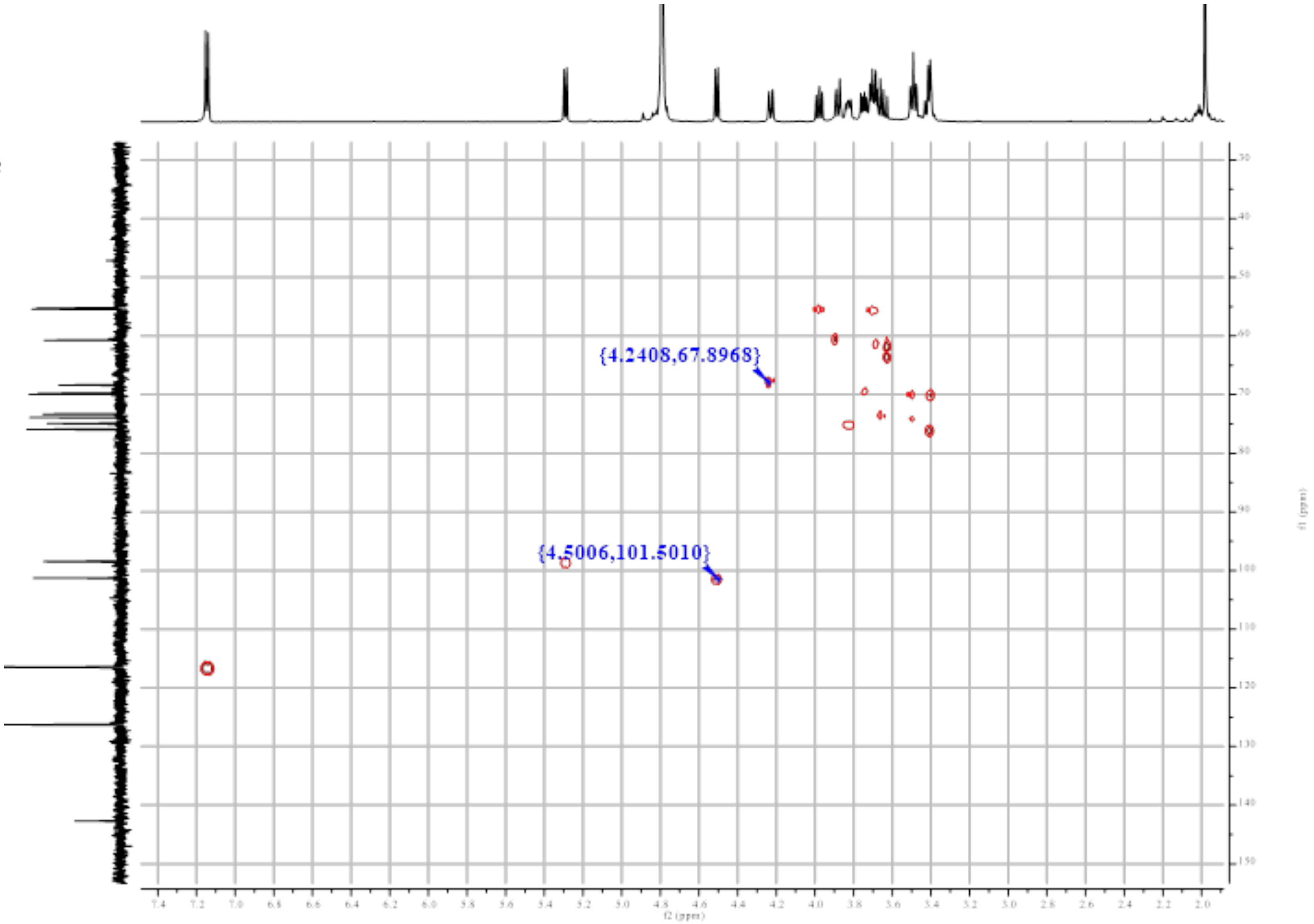

Compound **4h**, HMBC (600 MHz, D_2O)

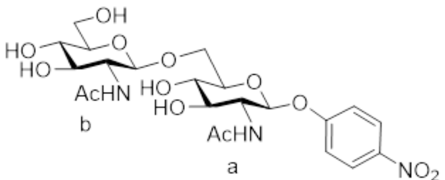

4h

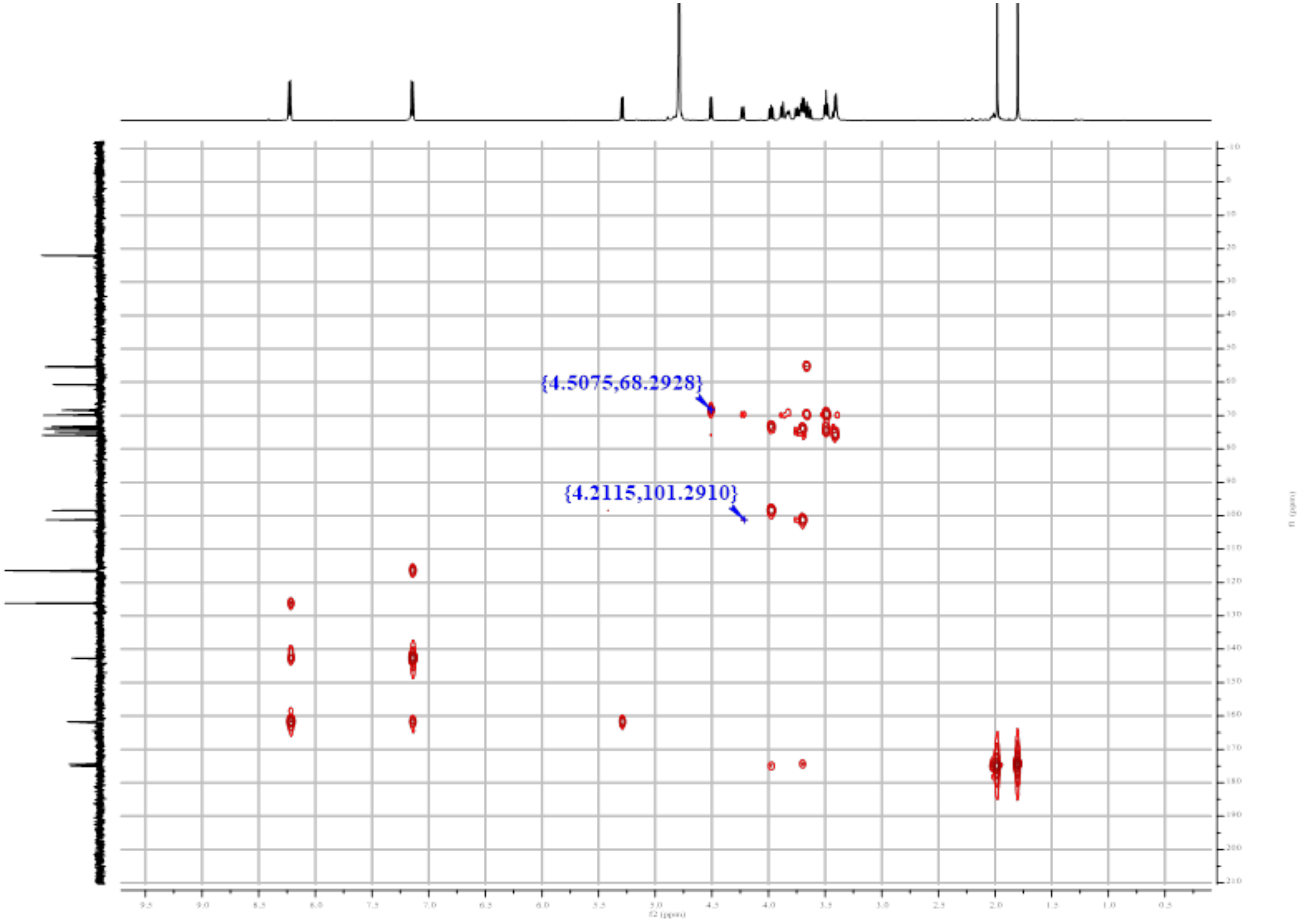

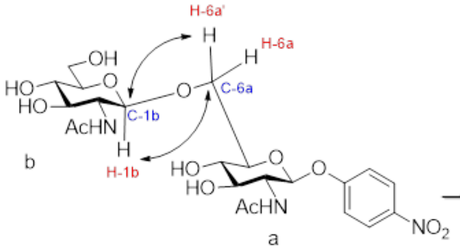
H-6a'
H-6a
OH
HO
HO
O
C-1b
O
C-6a
AcHN
H
b
H-1b
HO
HO
O
O
AcHN
a
NO2

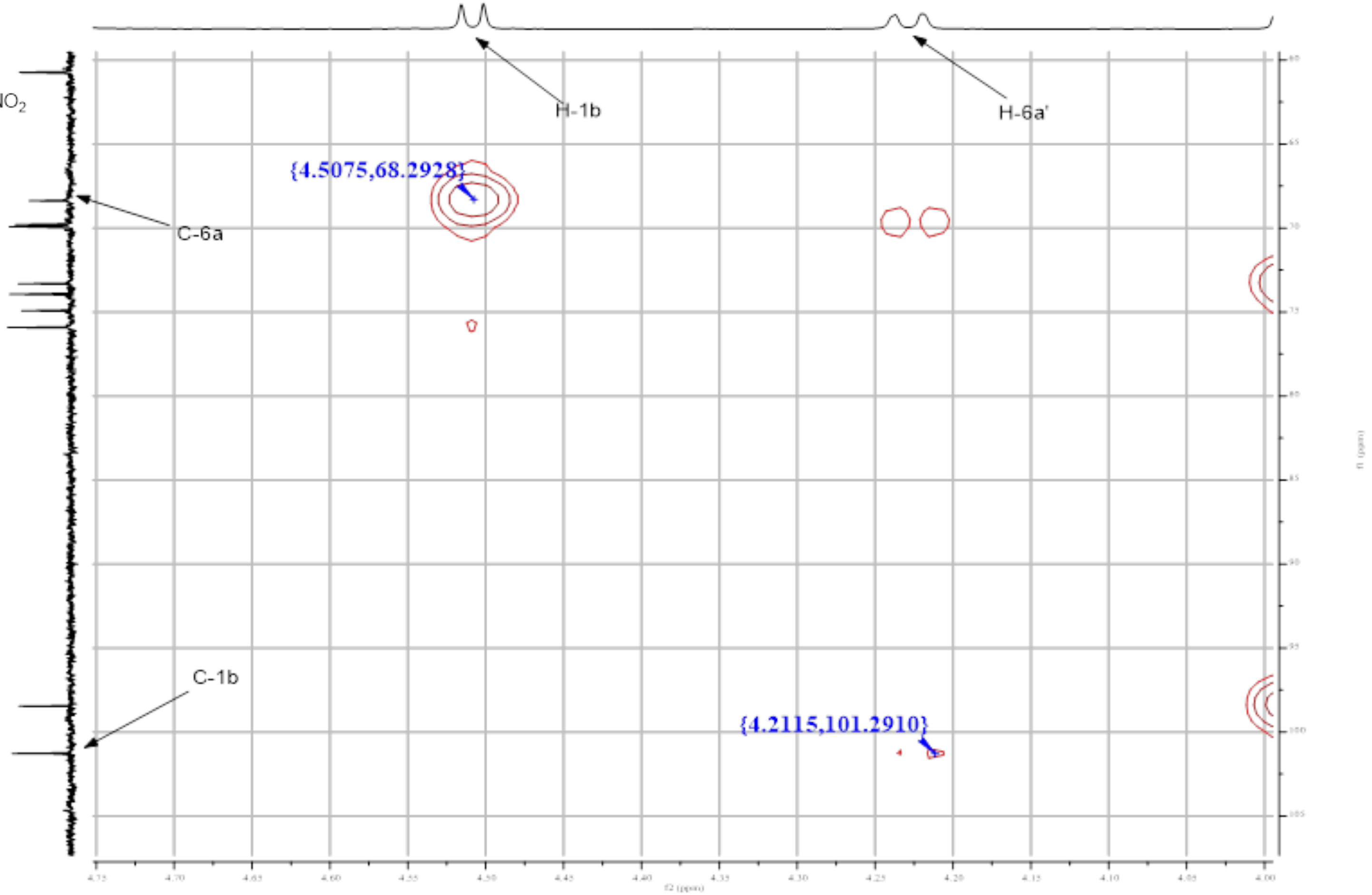
H-1b
H-6a'
{4.5075,68.2928}
C-6a
C-1b
{4.2115,101.2910}

2-acetamido-2-deoxy-β-D-glucopyranosyl-(1→6)-2-acetamido-2-deoxy-β-D-glucopyranosyl azide 4i, ^{1}H NMR (400 MHz, D_2O)

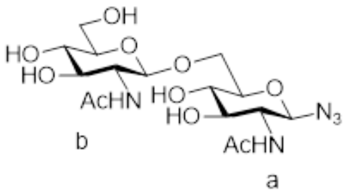

4i

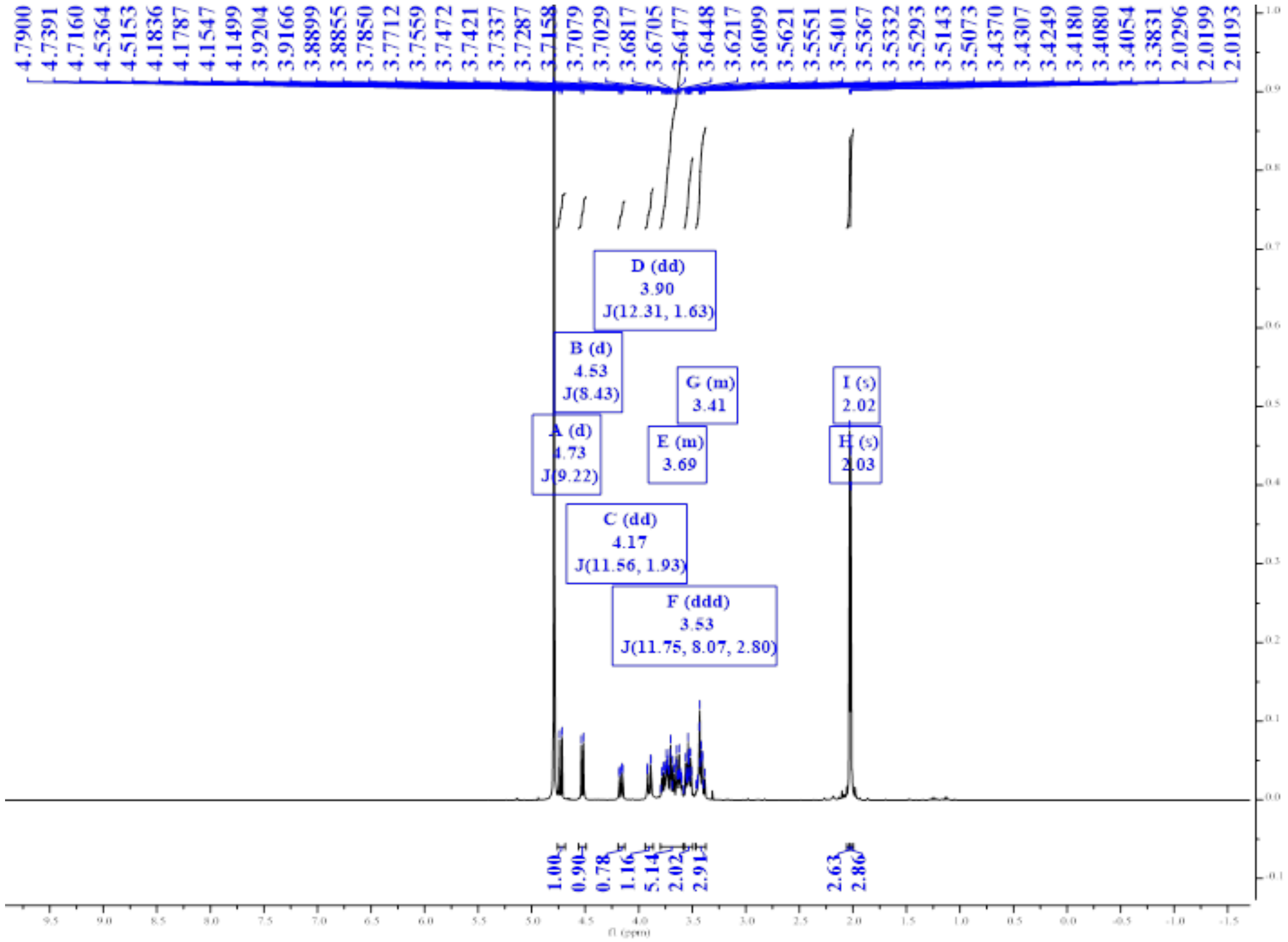

Compound **4i**, ^{13}C NMR (150 MHz, D_2O)

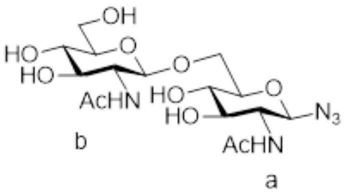

4i

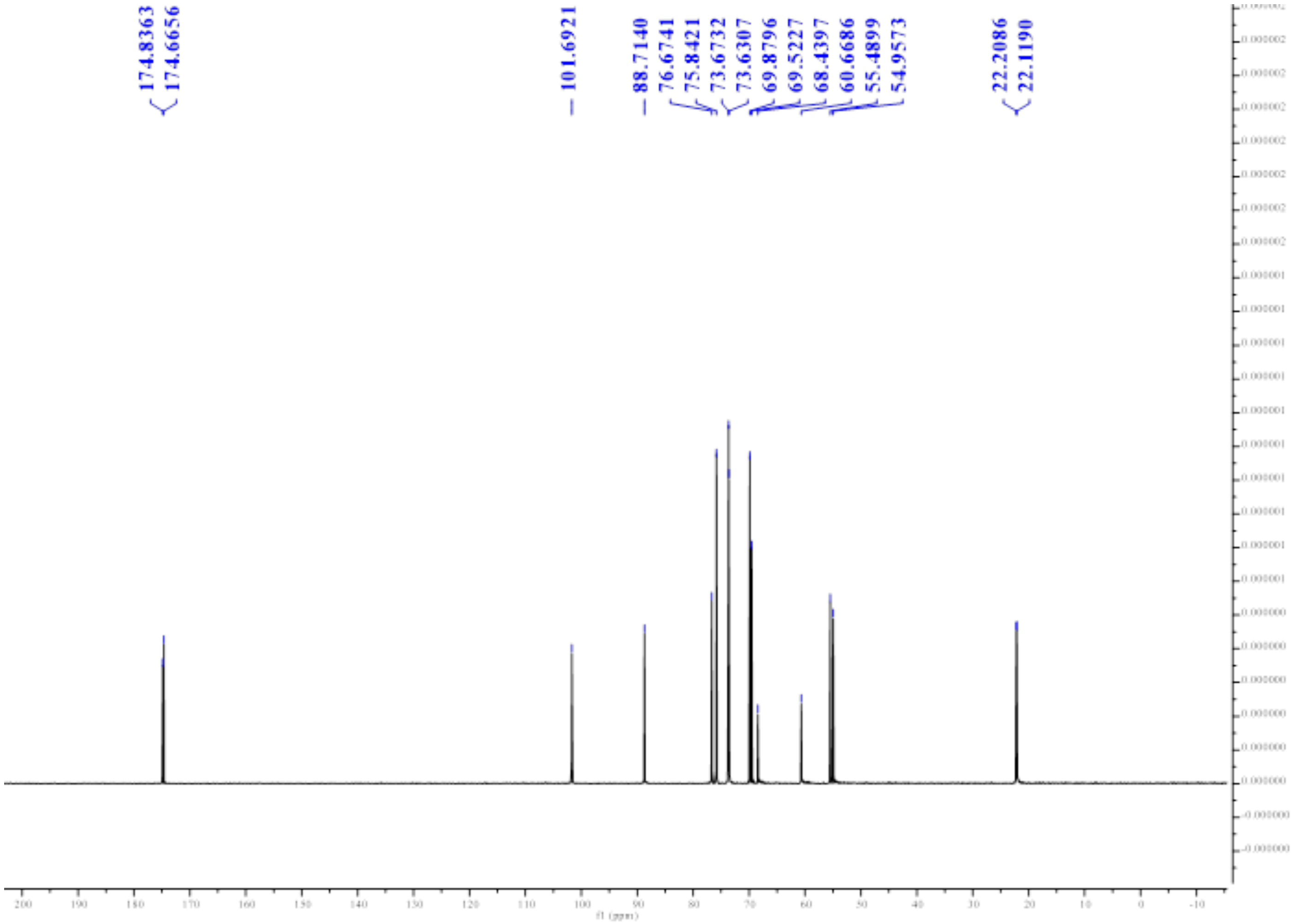

Compound **4i**, DEPT 135 NMR (150 MHz, D_2O)

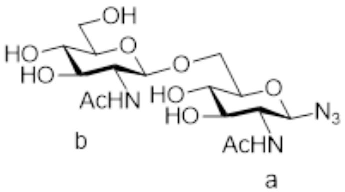

4i

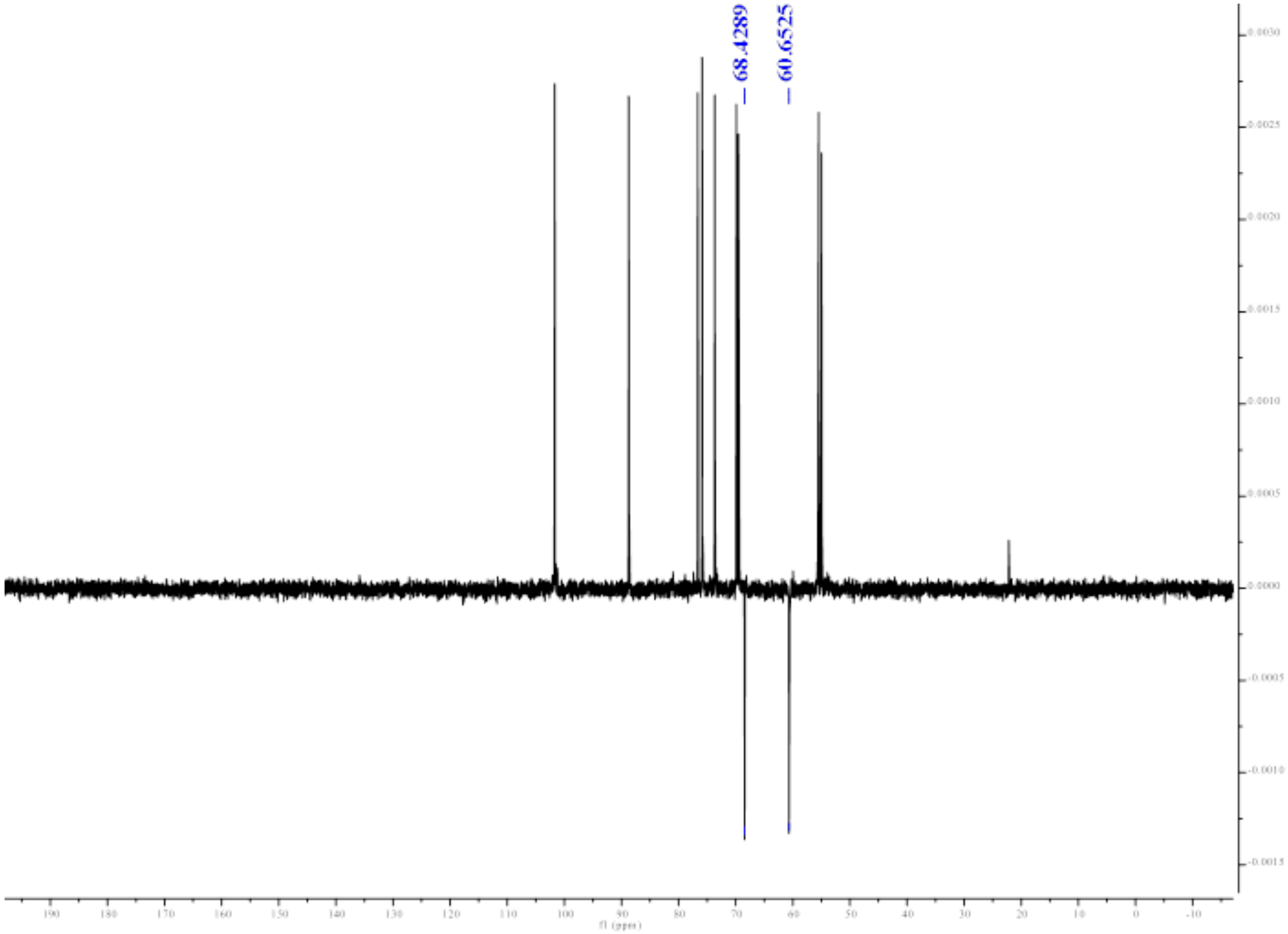

Compound **4i**, HMQC (600 MHz, D_2O)

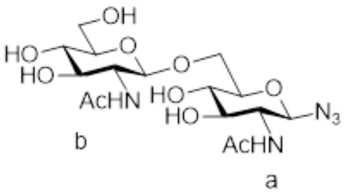

4i

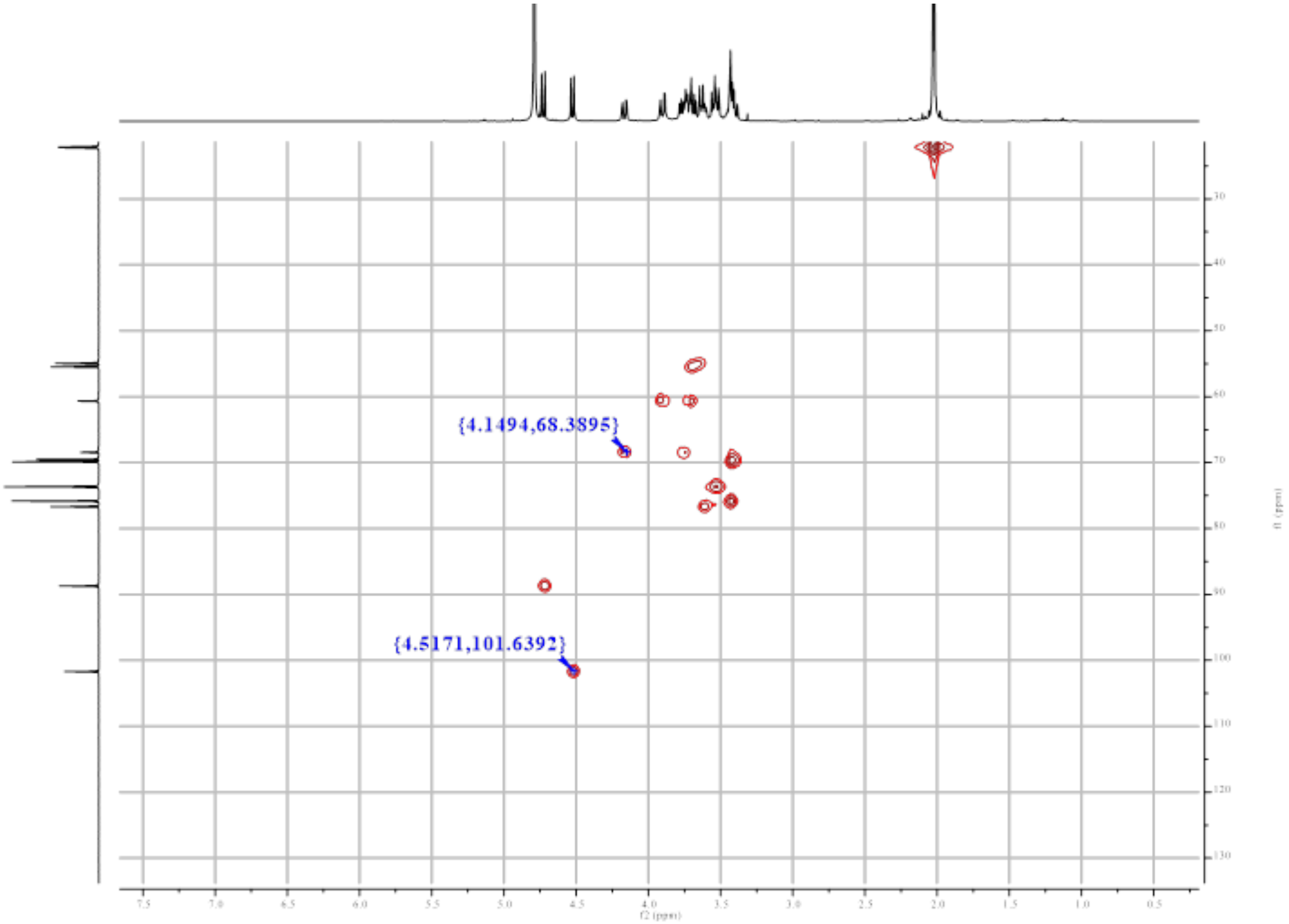

Compound **4i**, HMBC (600 MHz, D_2O)

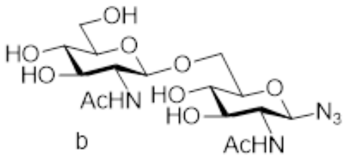

4i

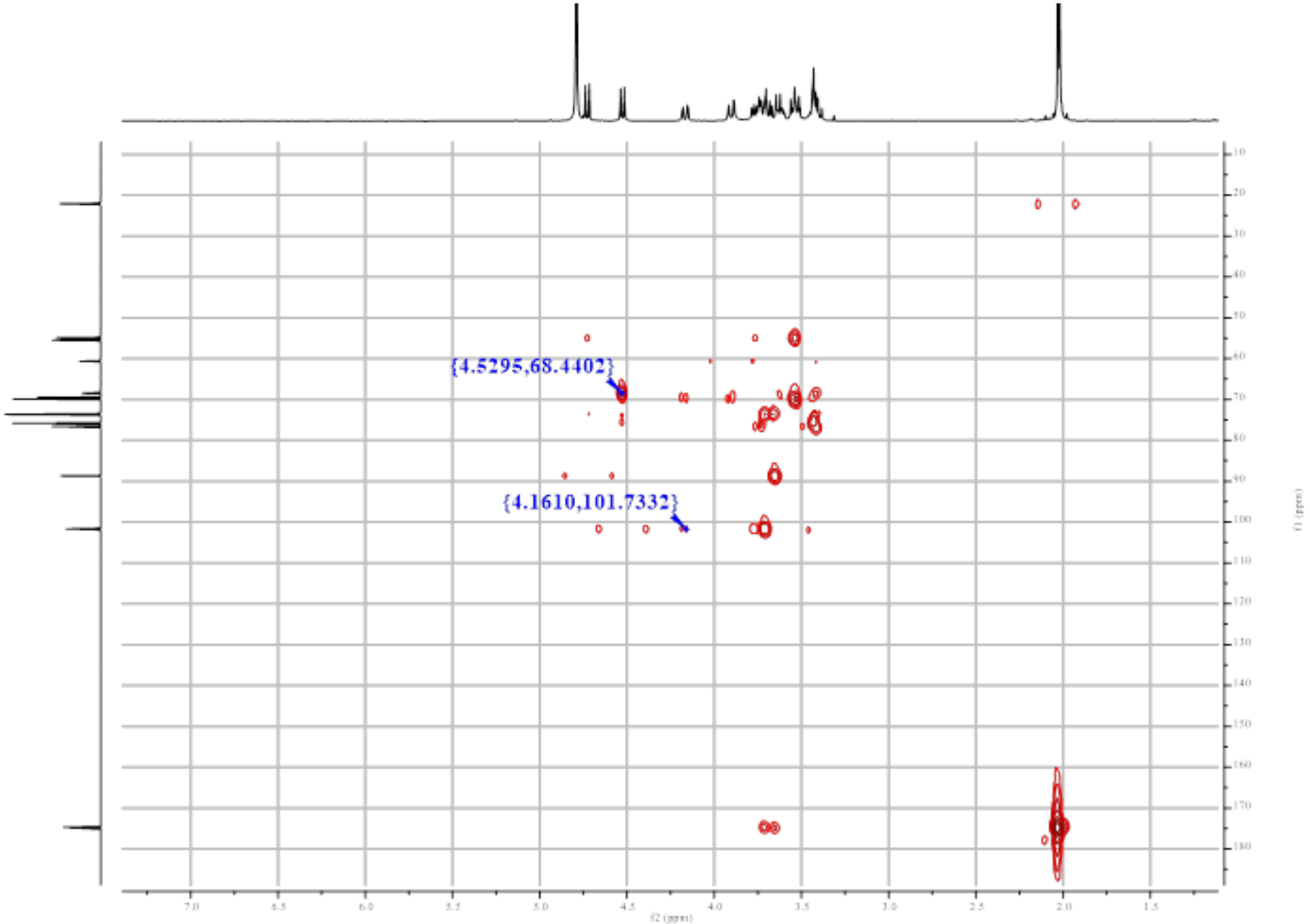

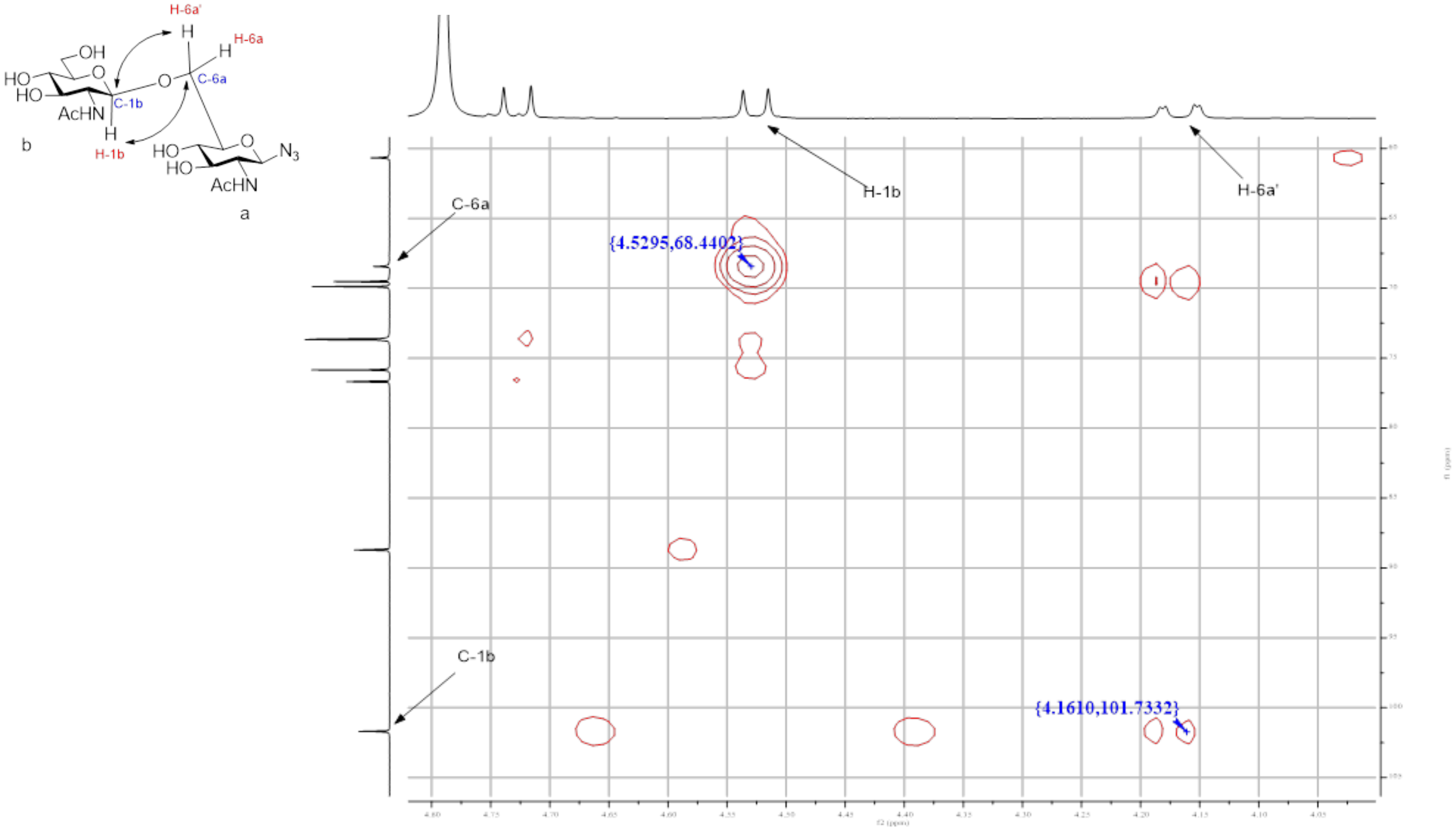
H-6a'
H-6a
C-6a
C-1b
H-1b
b
a
H-1b
H-6a'
C-6a
{4.5295,68.4402}
C-1b
{4.1610,101.7332}

***t*-Butyl 2-acetamido-2-deoxy-β-D-glucopyranosyl-(1→6)-2-acetamido-2-deoxy-β-D-glucopyranoside 4j**, ^{1}H NMR (600 MHz, D_2O)

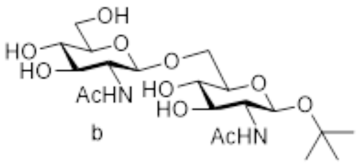

4j

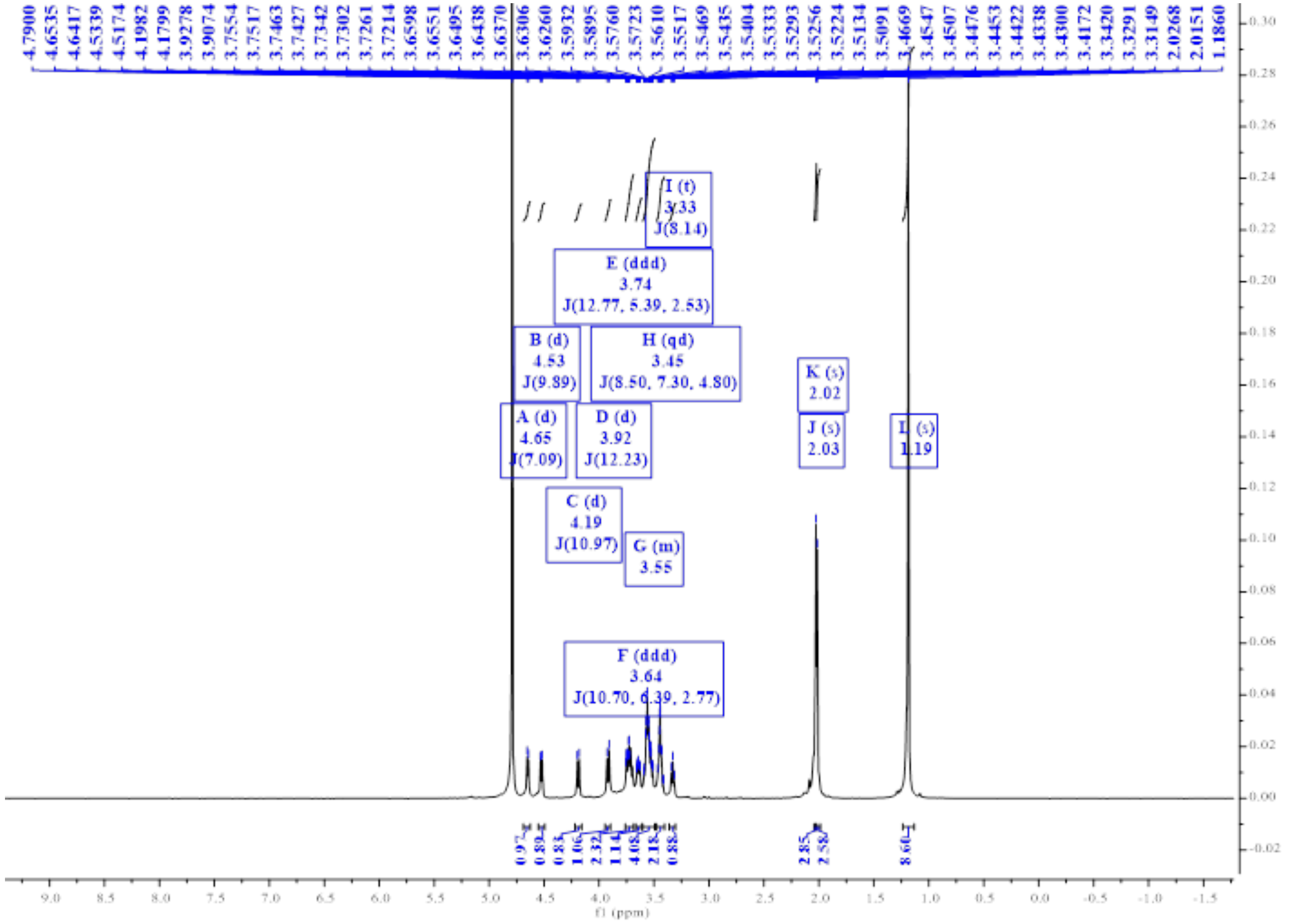

Compound **4j**, ^{13}C (150 MHz, D_2O)

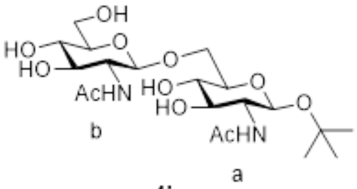

4j

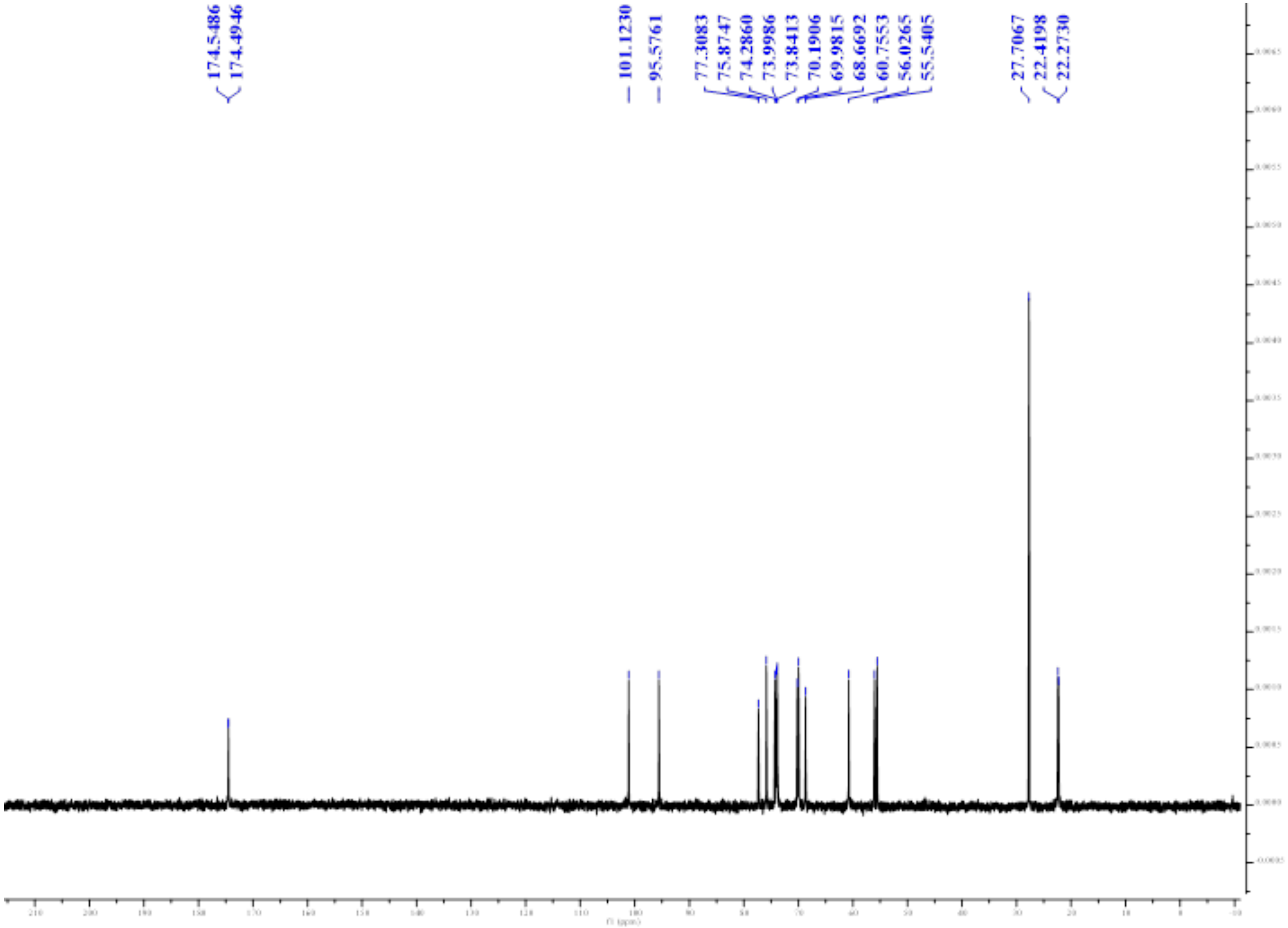

Compound **4j**, DEPT 135 (150 MHz, D_2O)

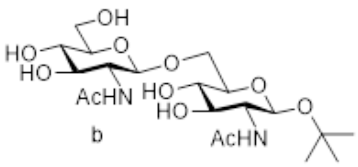

4j

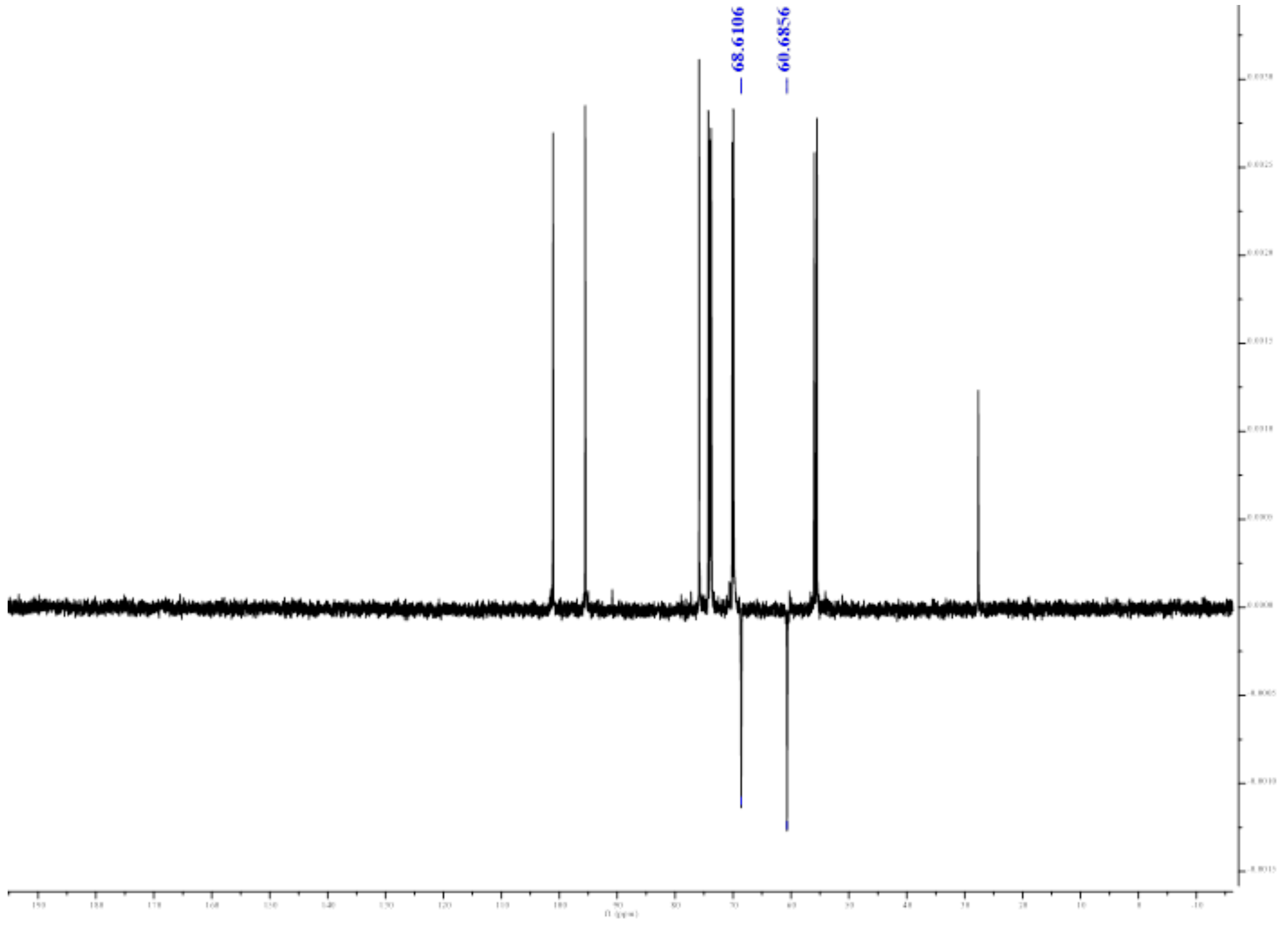

Compound **4j**, HMQC (150 MHz, D_2O)

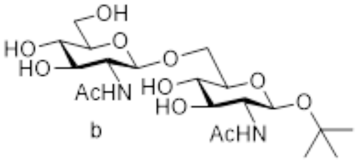

4j

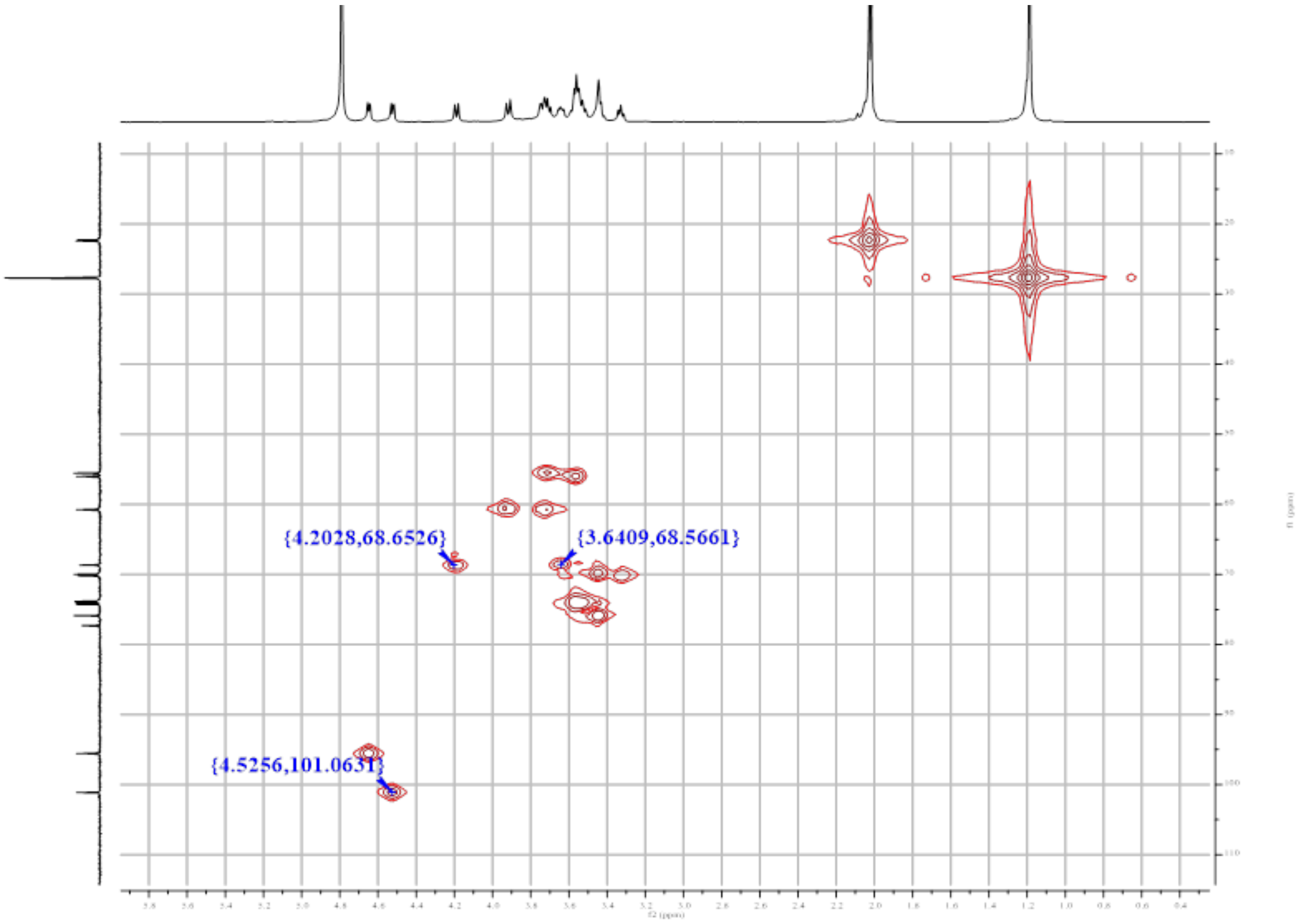

Compound **4j**, HMBC (150 MHz, D_2O)

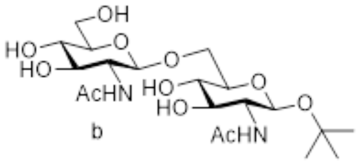

4j

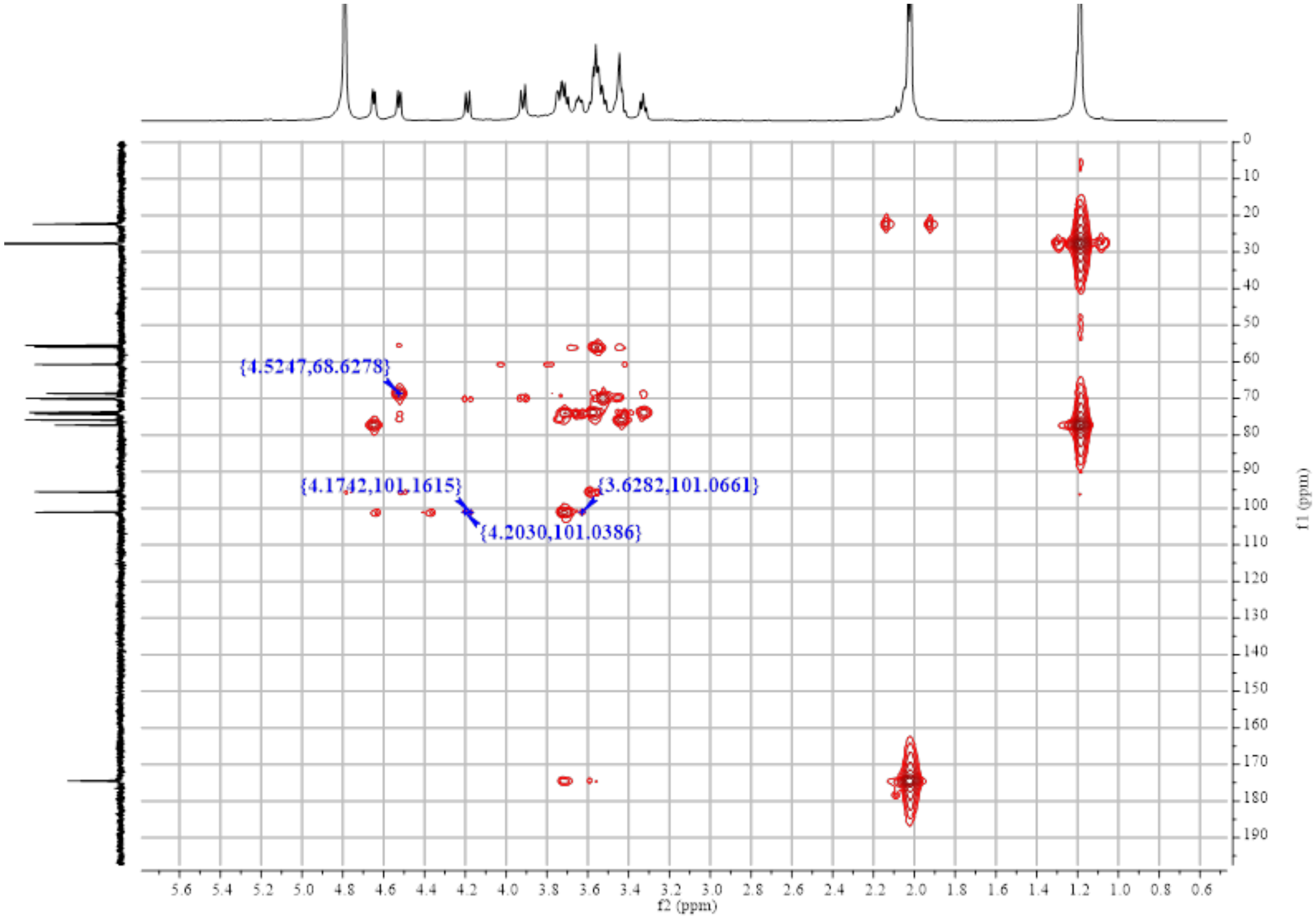

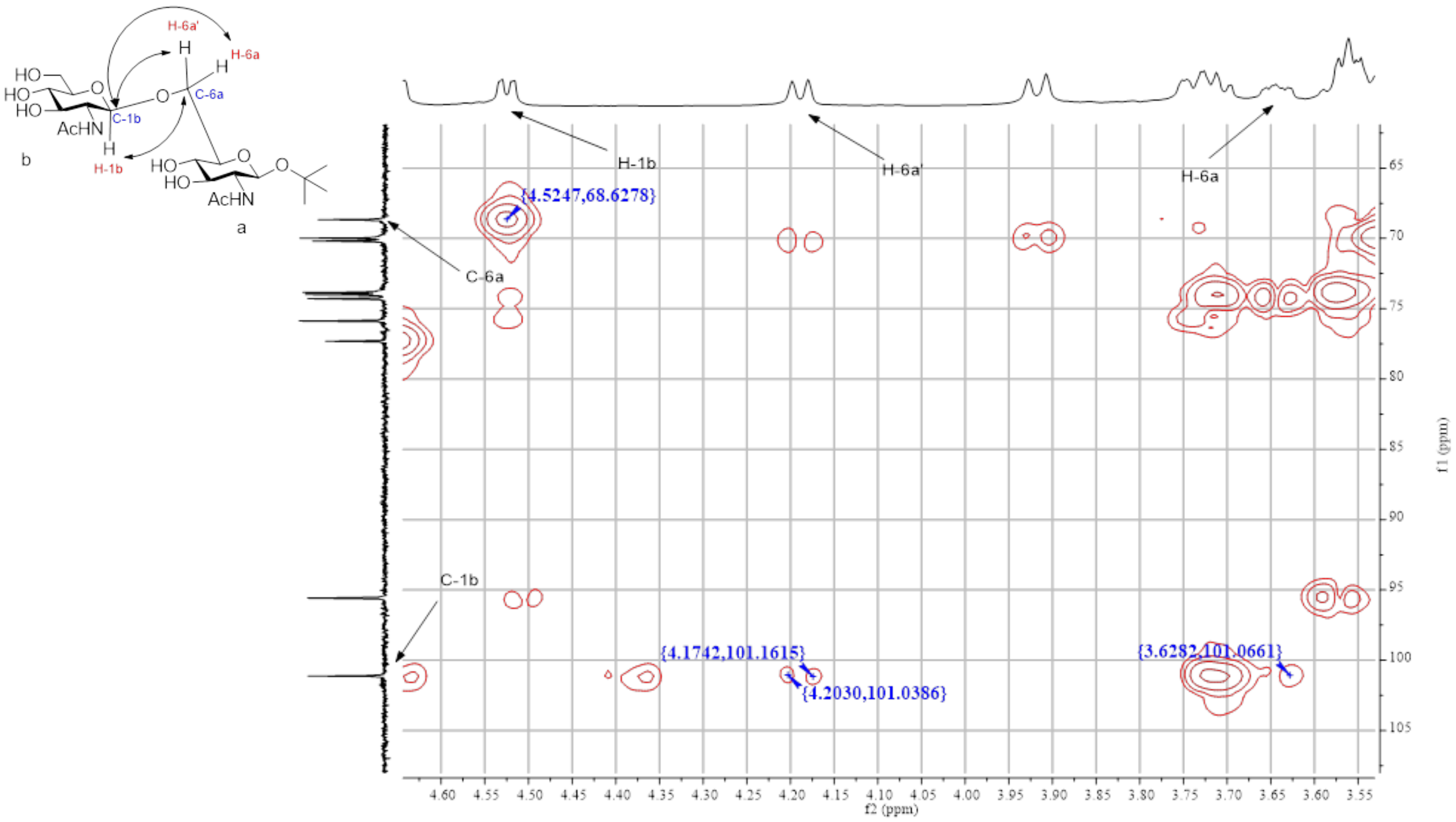
H-6a'
H-6a
H
H
HO
HO
HO
O
O
C-6a
C-1b
AcHN
H
H-1b
b
HO
HO
O
O
AcHN
a
H-1b
H-6a'
H-6a
{4.5247,68.6278}
C-6a
C-1b
{4.1742,101.1615}
{4.2030,101.0386}
{3.6282,101.0661}
f2 (ppm)
f1 (ppm)

Phenyl 2-acetamido-2-deoxy-β-D-glucopyranosyl-(1→6)-β-D-glucopyranoside 4k, ^{1}H NMR (400 MHz, D_2O)

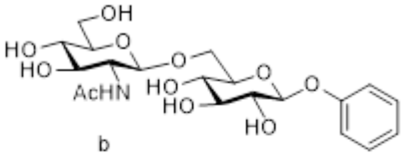

4k

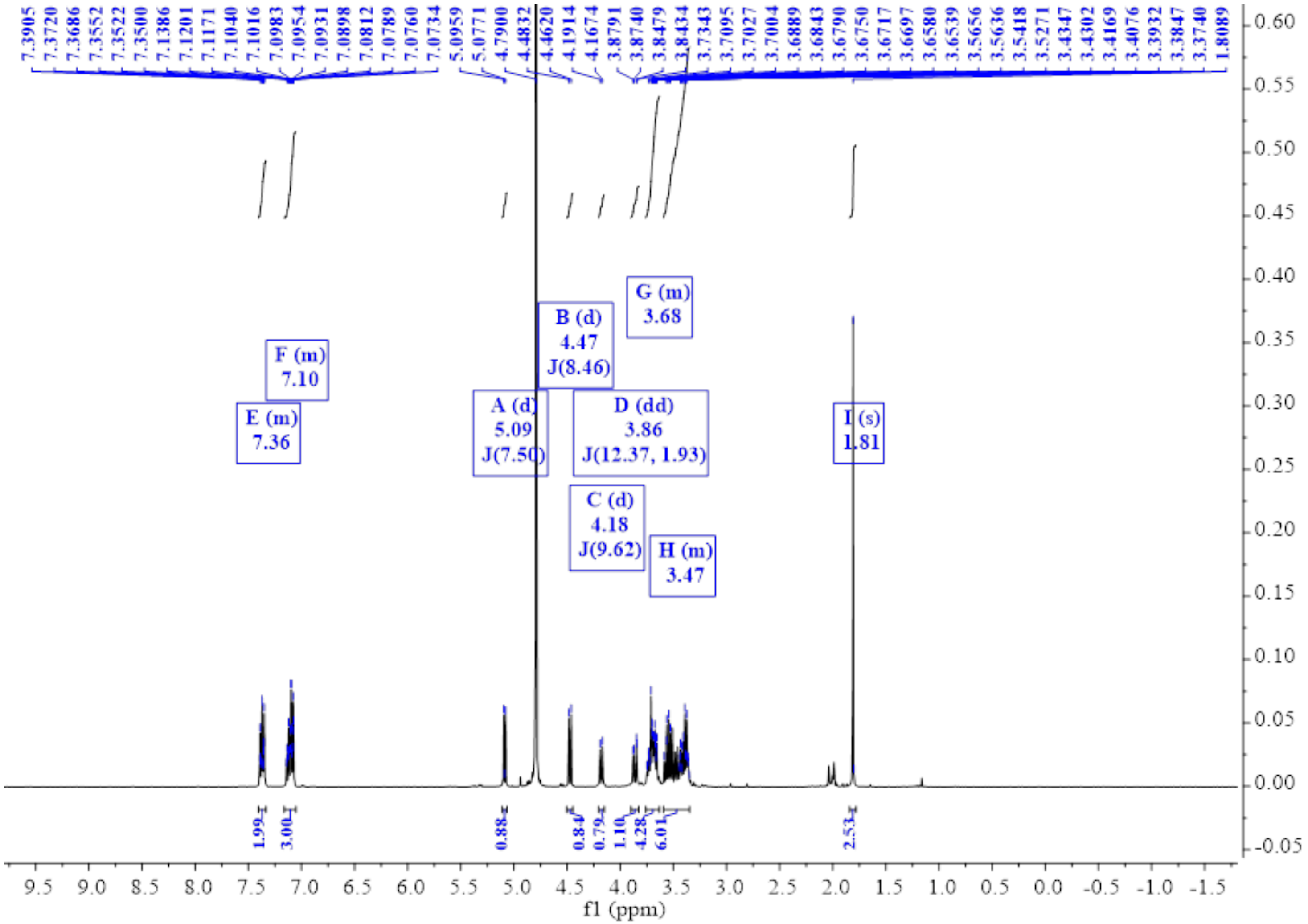

Compound **4k**, ^{13}C NMR (150 MHz, D_2O)

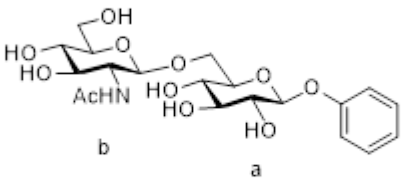

4k

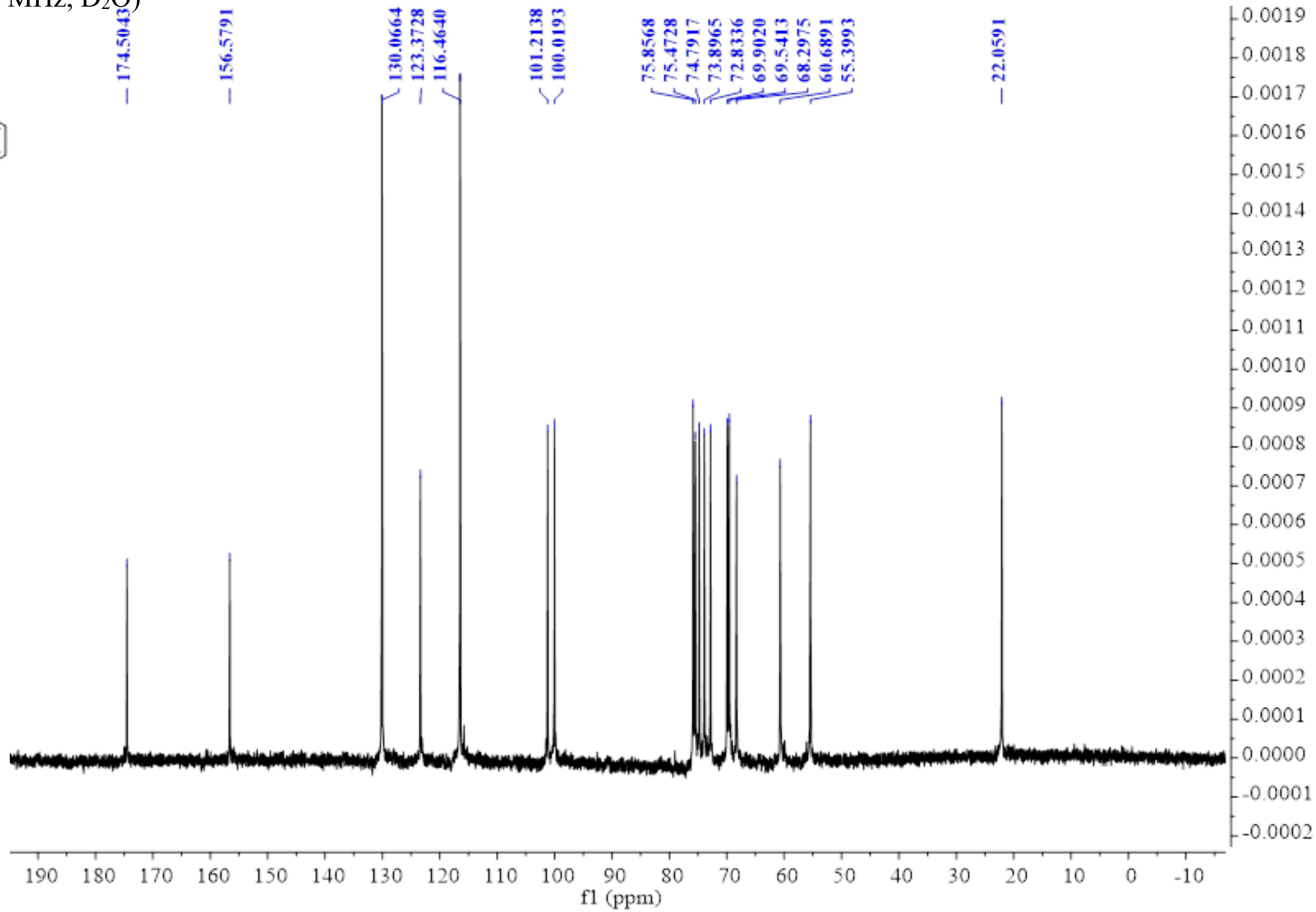

Compound **4k**, DEPT 135 (100 MHz, D_2O)

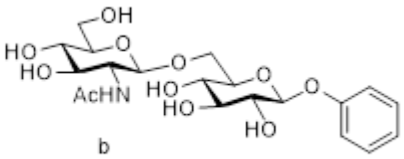

4k

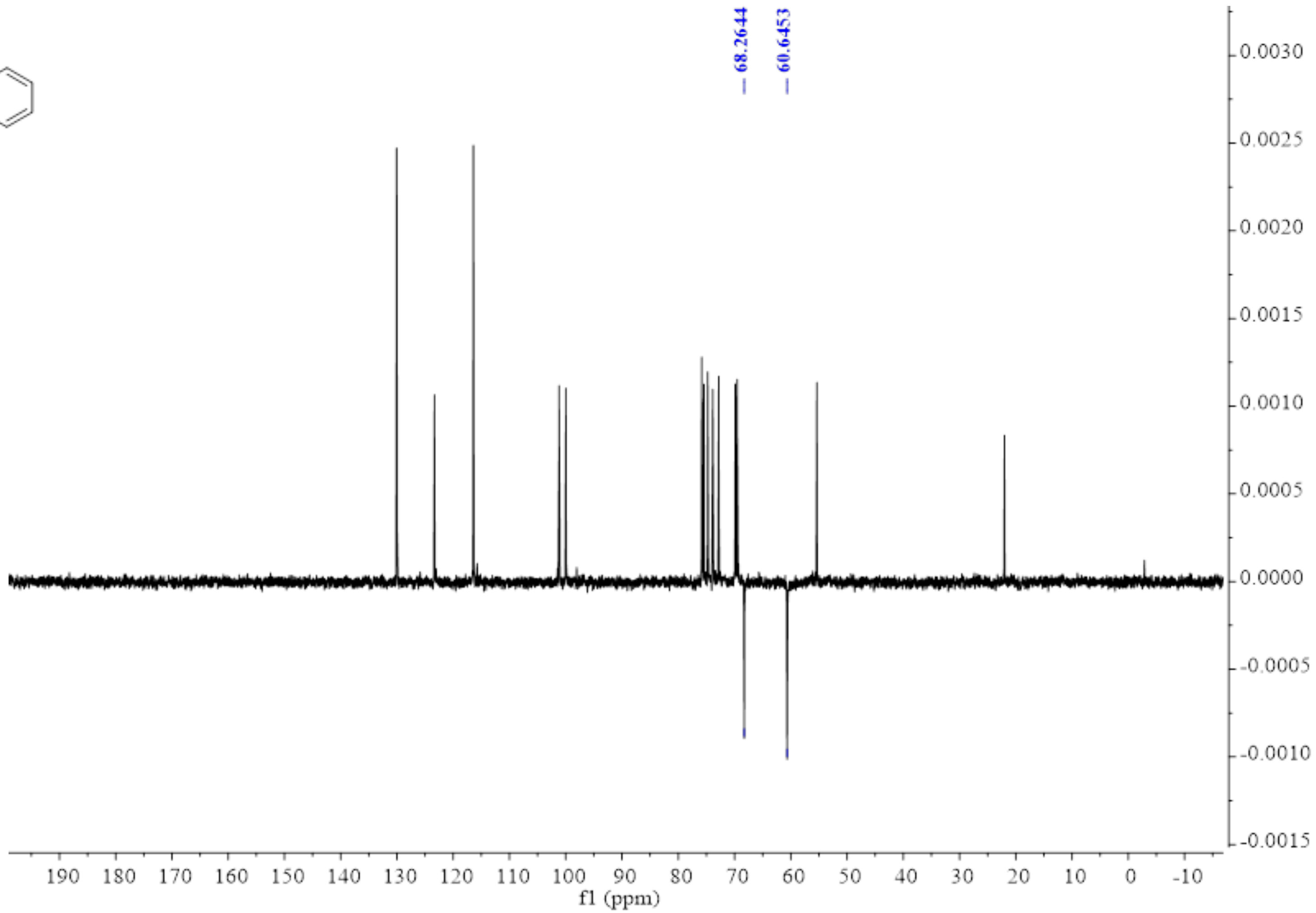

Compound **4k**, HMQC (400 MHz, D_2O)

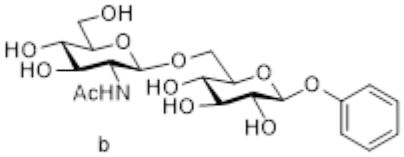

4k

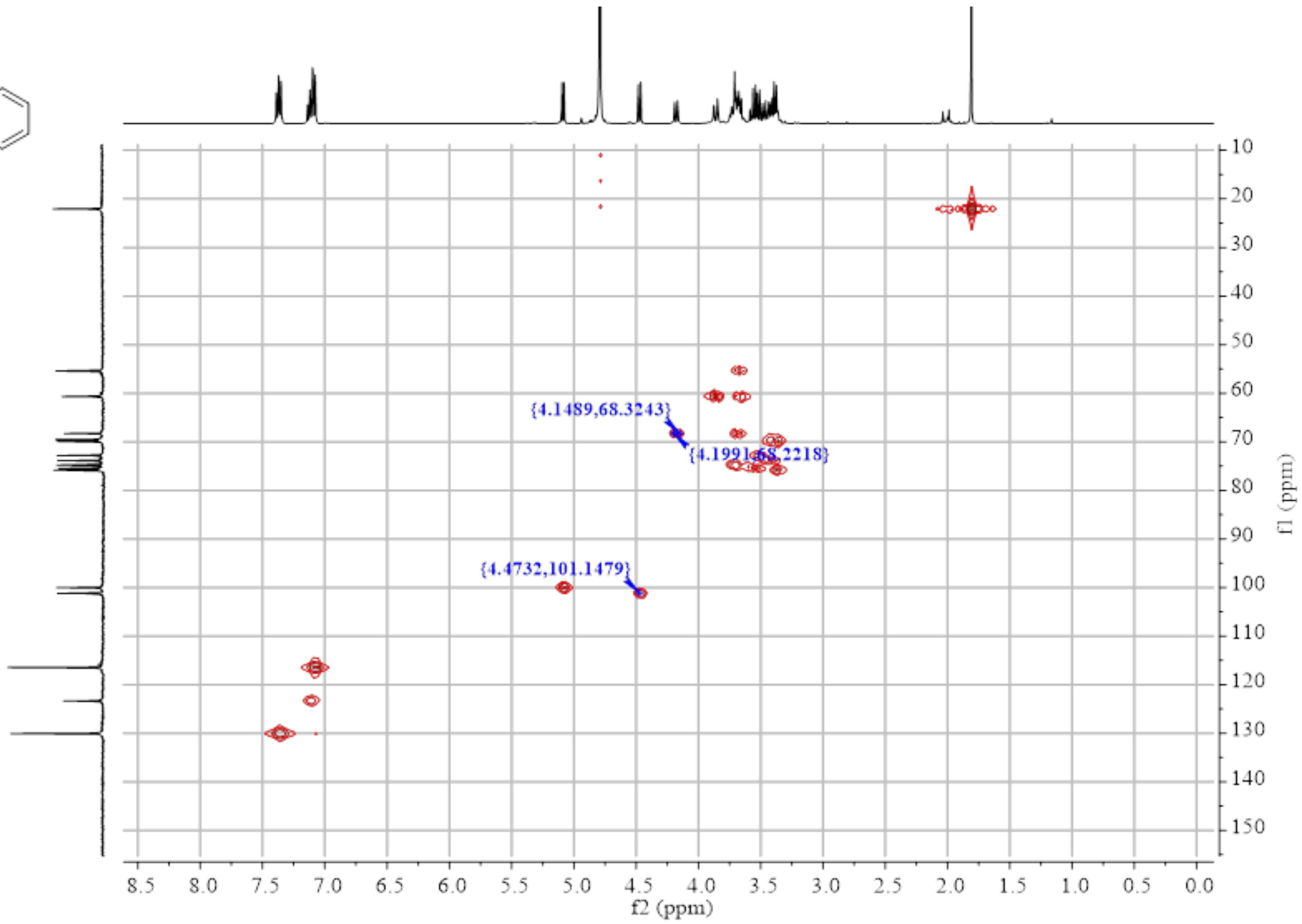

Compound **4k**, HMBC (400 MHz, D_2O)

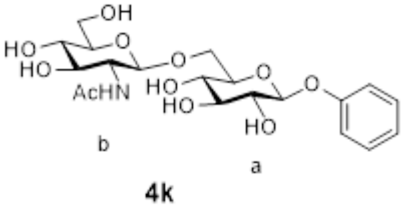

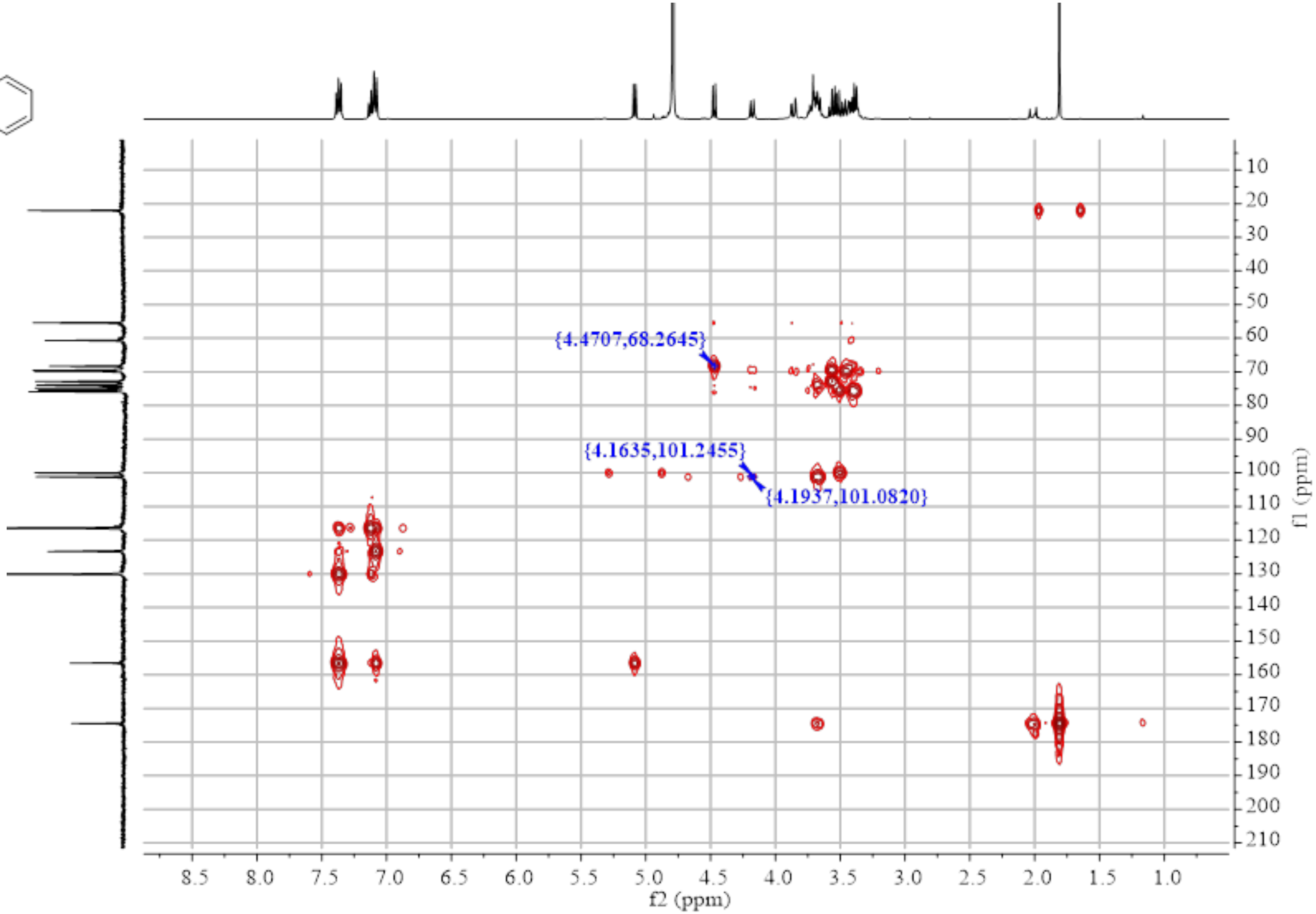

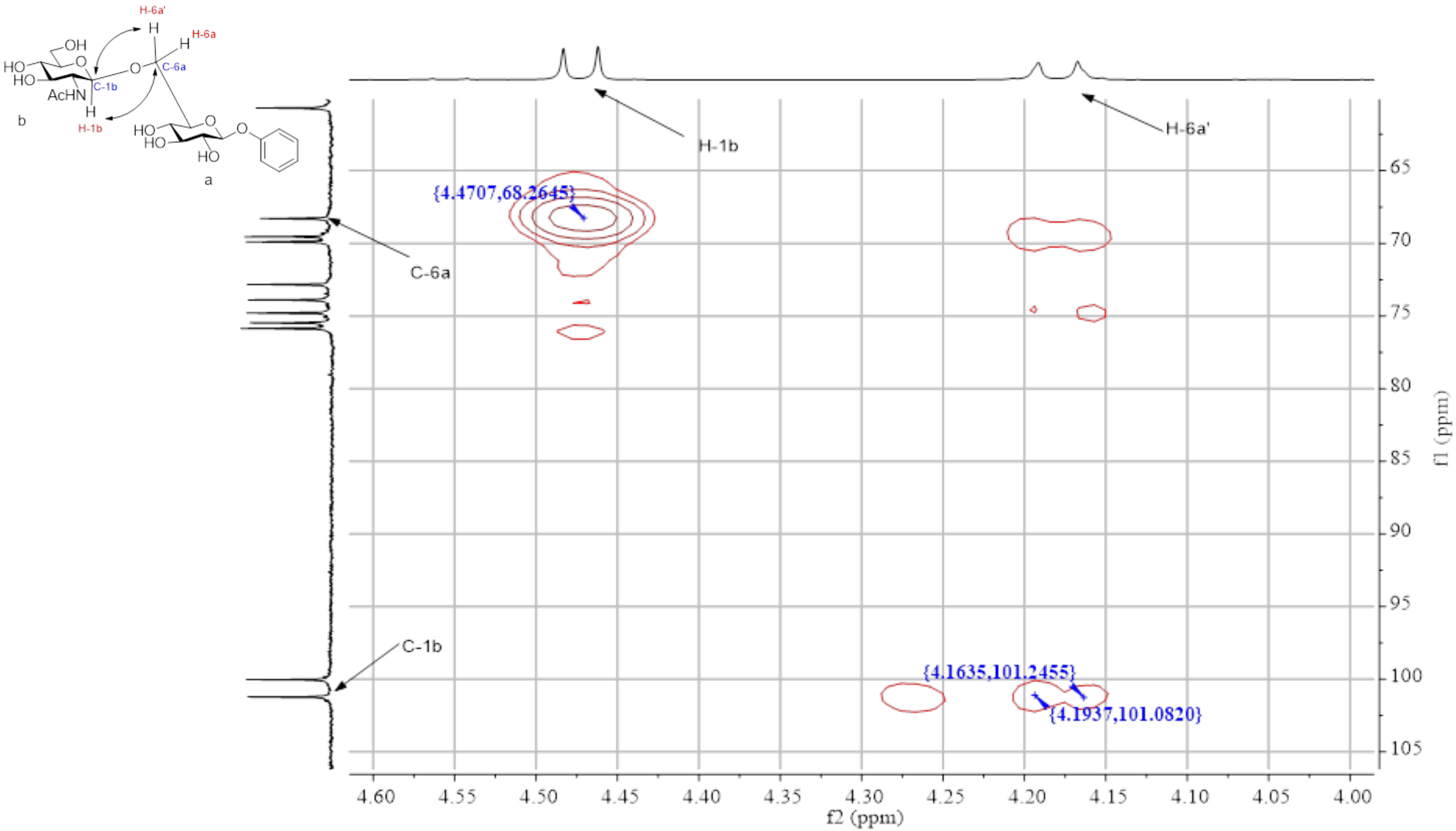
H-6a'
H-6a
C-6a
C-1b
H-1b
HO
OH
AcHN
HO
O
b
a
H-1b
H-6a'
C-6a
C-1b
{4.4707,68.2645}
{4.1635,101.2455}
{4.1937,101.0820}
f2 (ppm)
f1 (ppm)

Phenyl 2-acetamido-2-deoxy-β-D-glucopyranosyl-(1→6)-β-D-thioglucopyranoside 4l, ^{1}H NMR (600 MHz, D_2O)

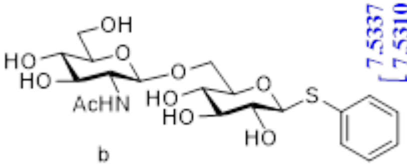

4l

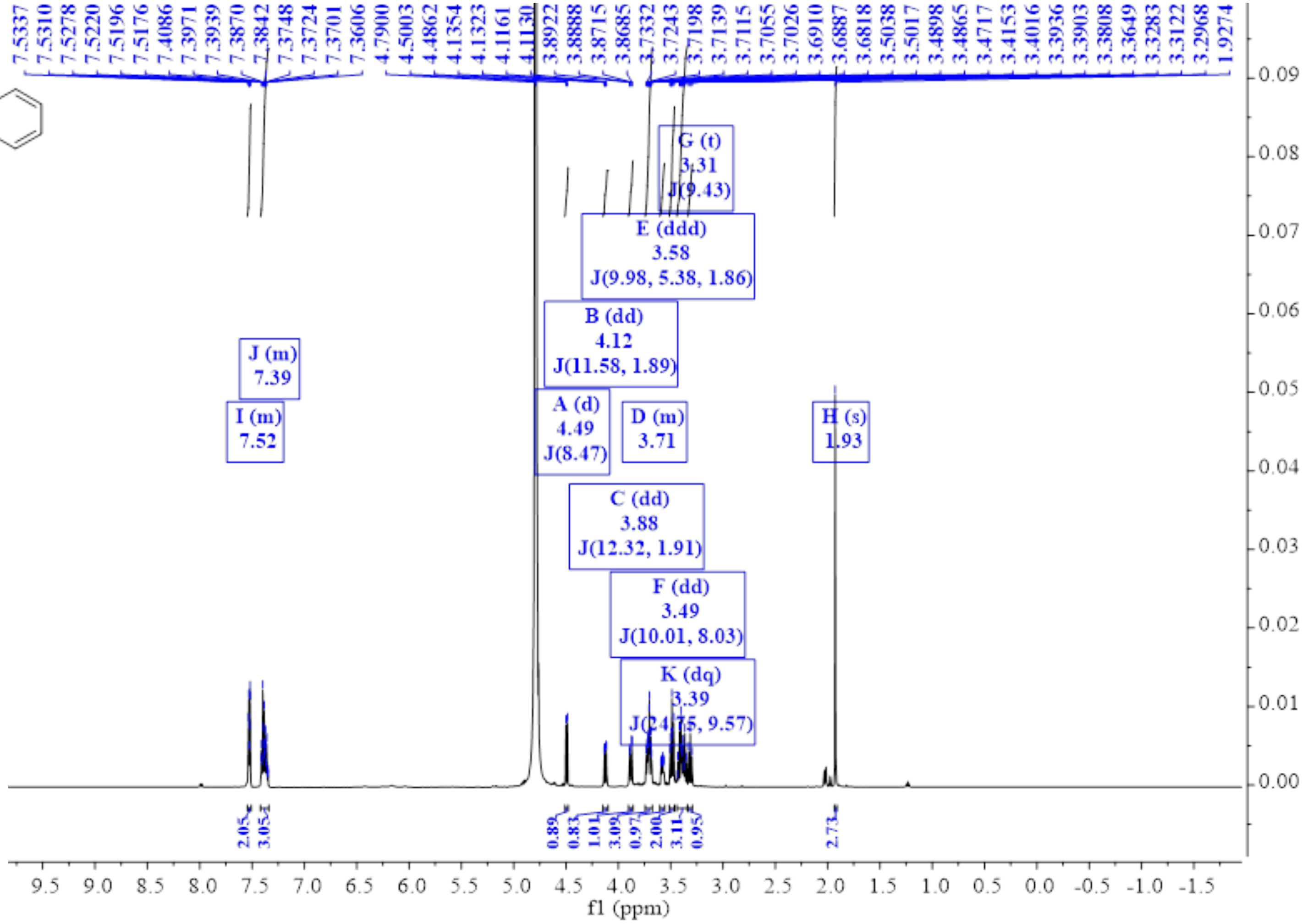

Compound **4l**, ^{13}C NMR (150 MHz, D_2O)

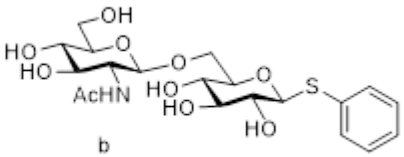

4l

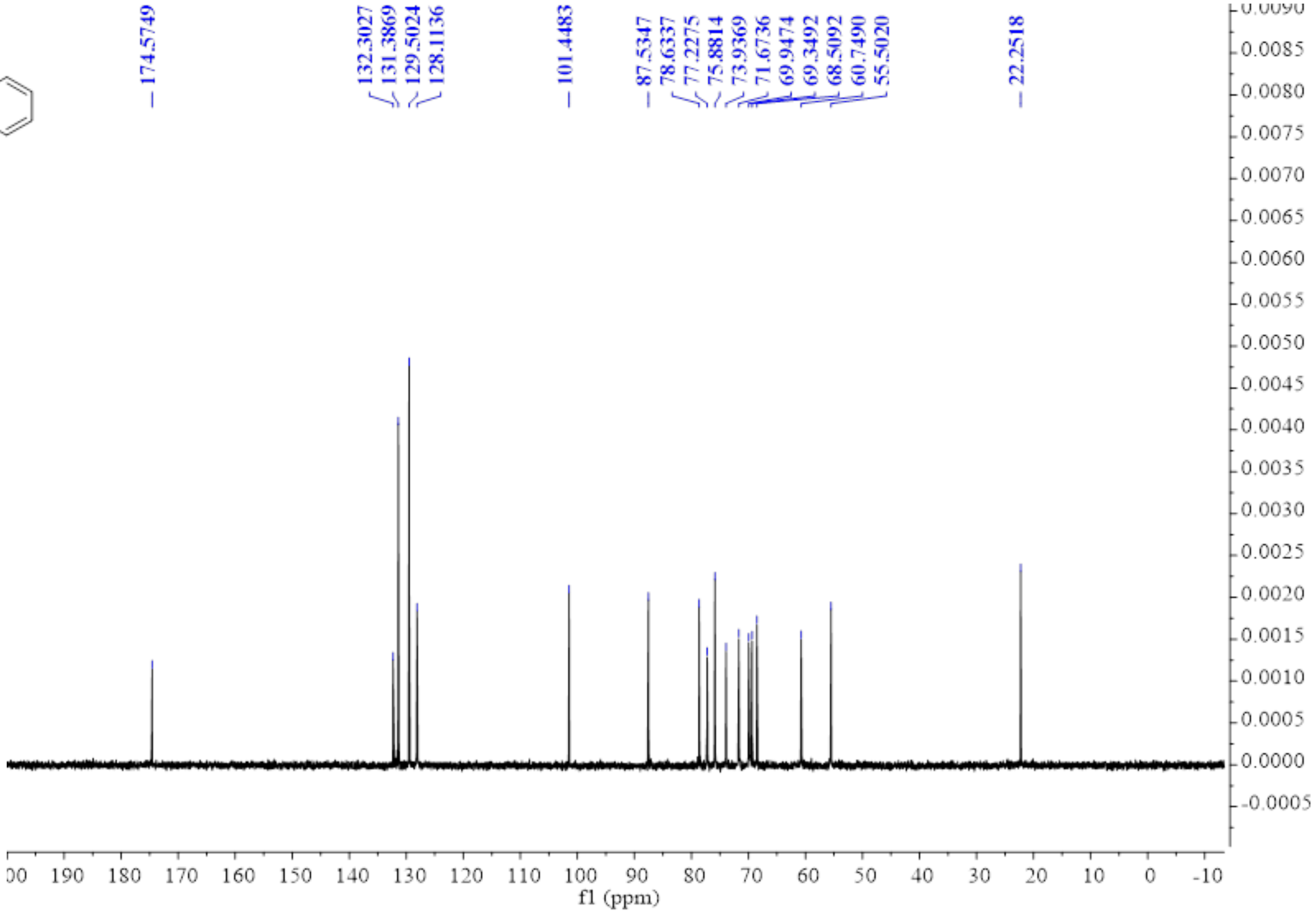

Compound **4l**, HMQC (600 MHz, D_2O)

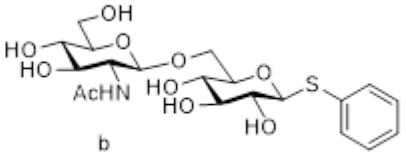

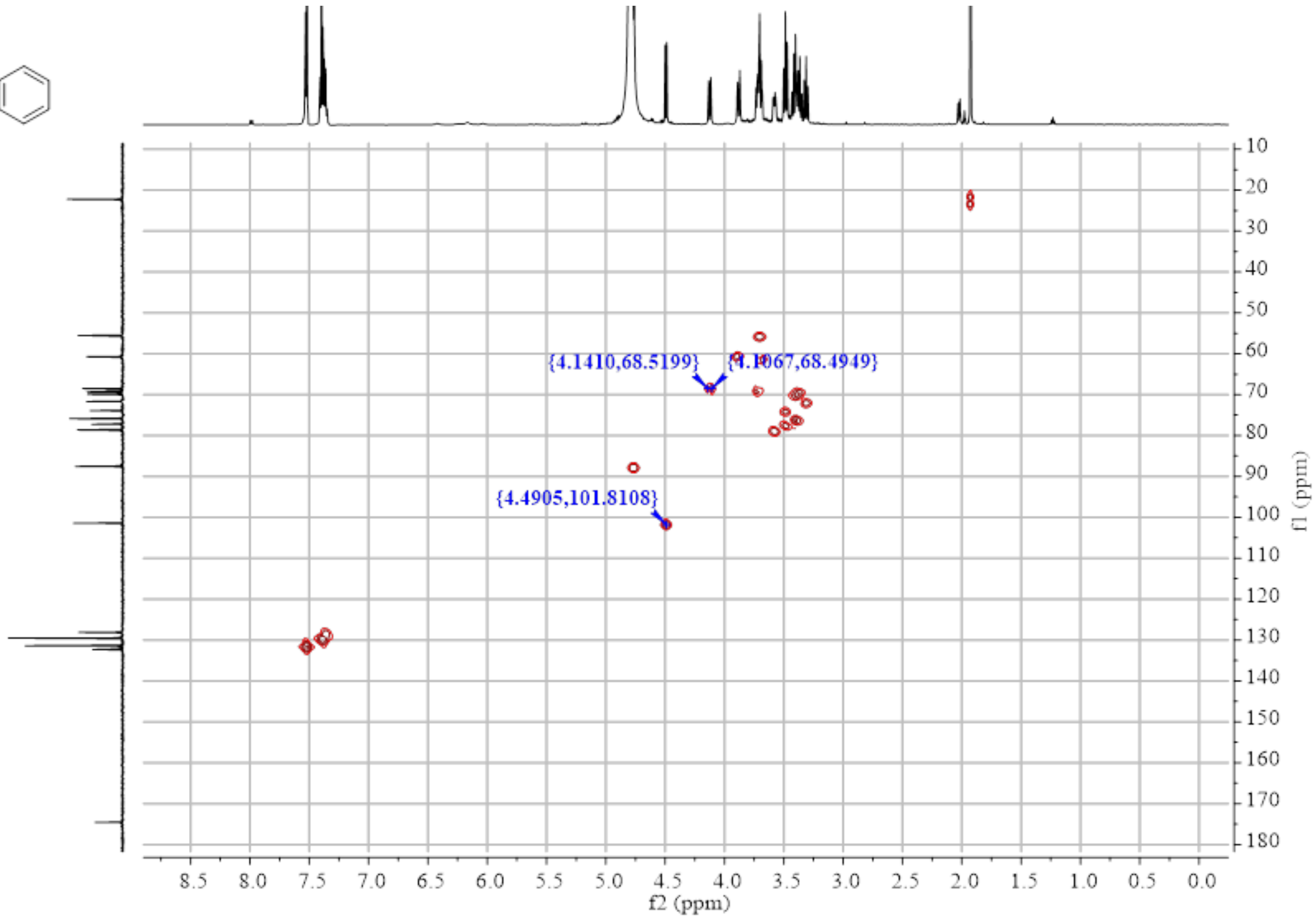

Compound **4l**, HMBC (600 MHz, D_2O)

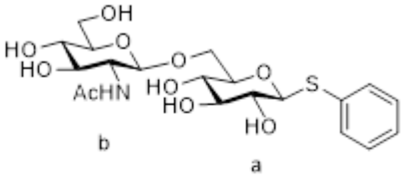

4l

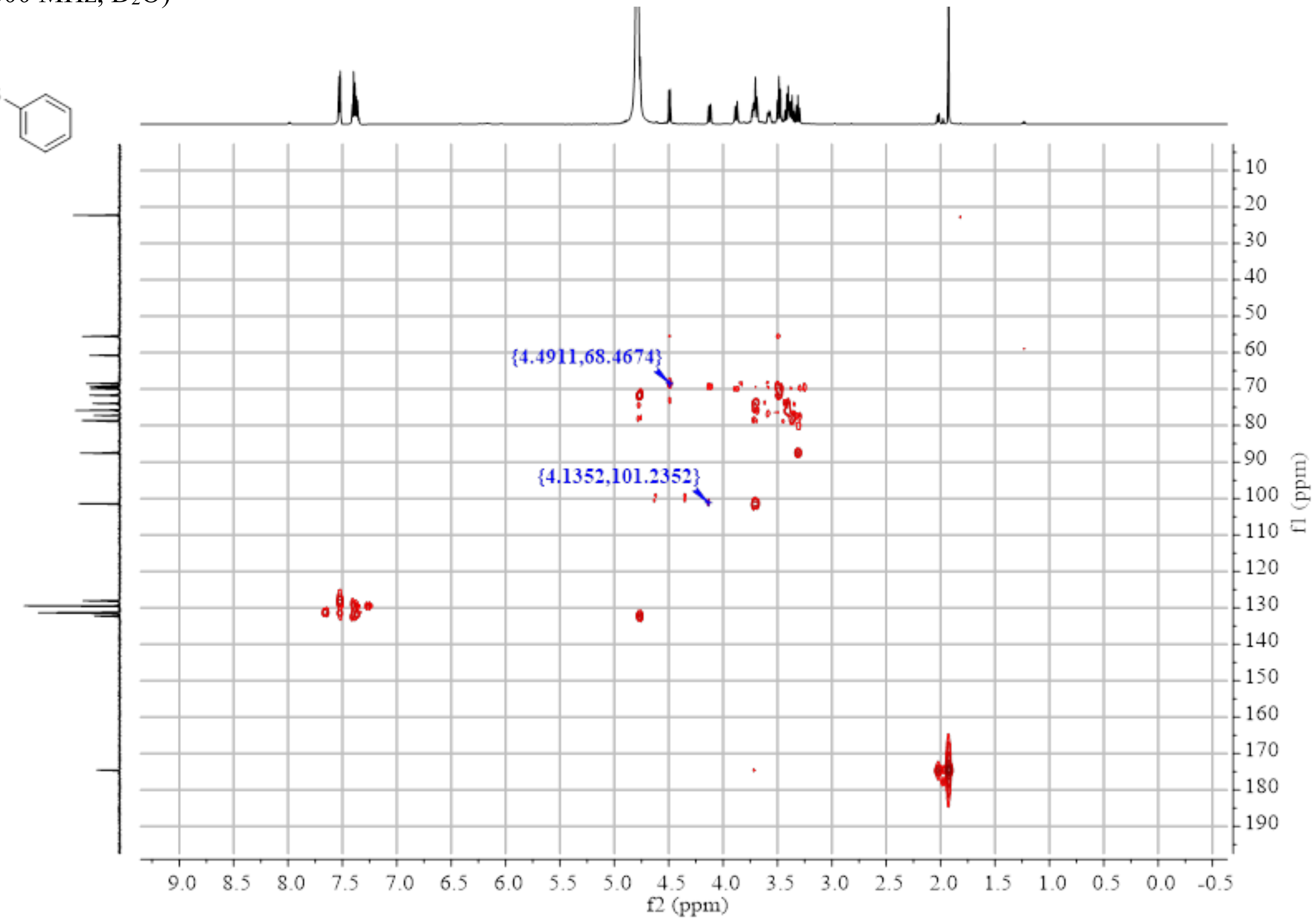

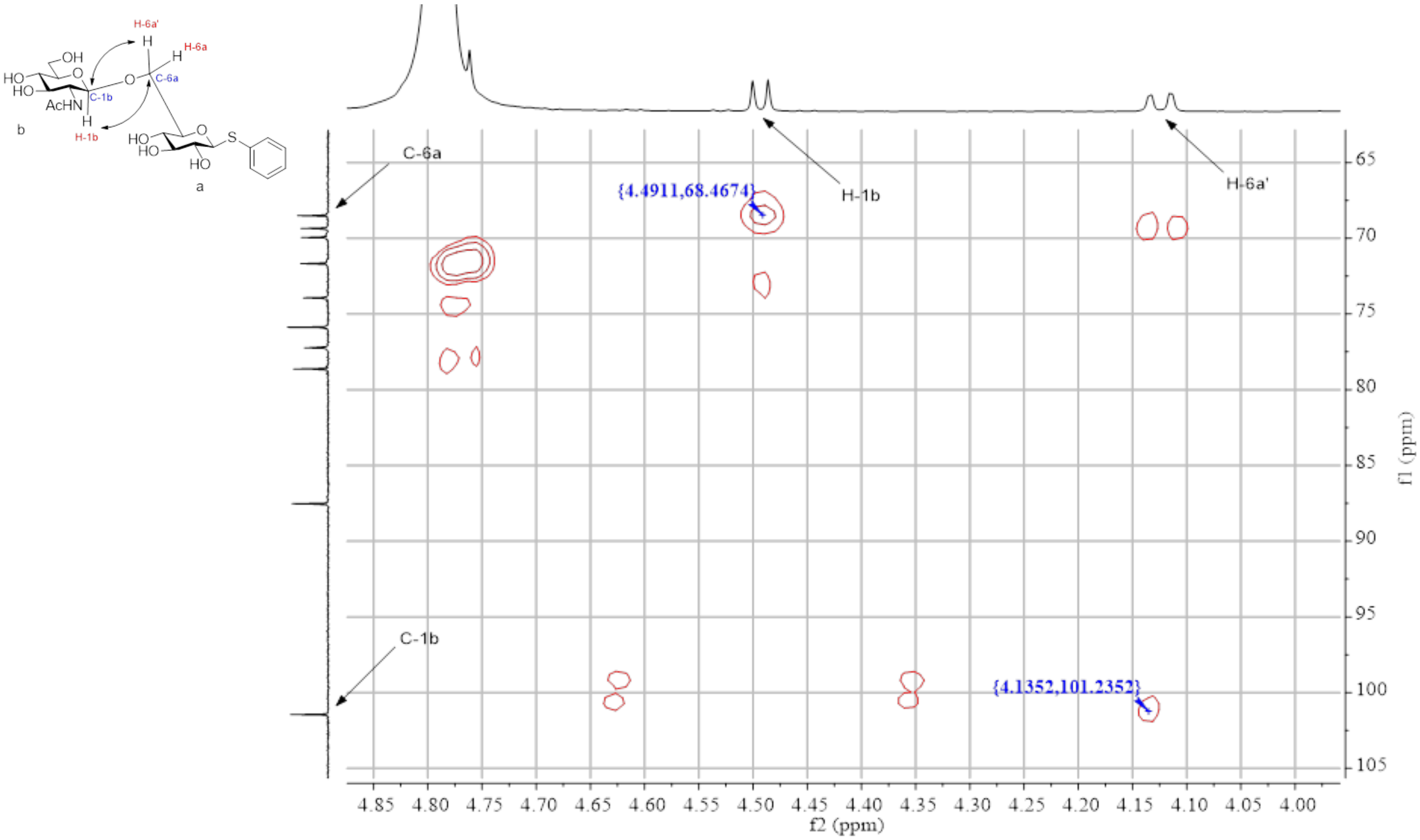

H-6a'
H-6a
C-6a
C-1b
H-1b
b
a
C-6a
C-1b
{4.4911,68.4674}
{4.1352,101.2352}
H-1b
H-6a'
f2 (ppm)
f1 (ppm)

Methyl 2-acetamido-2-deoxy-β-D-glucopyranosyl-(1→6)-β-D-glucopyranoside 4m ^{1}H NMR (400 MHz, D_2O)

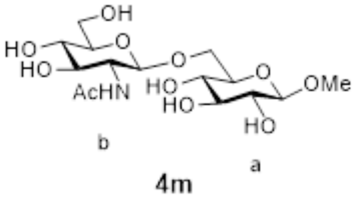

4m

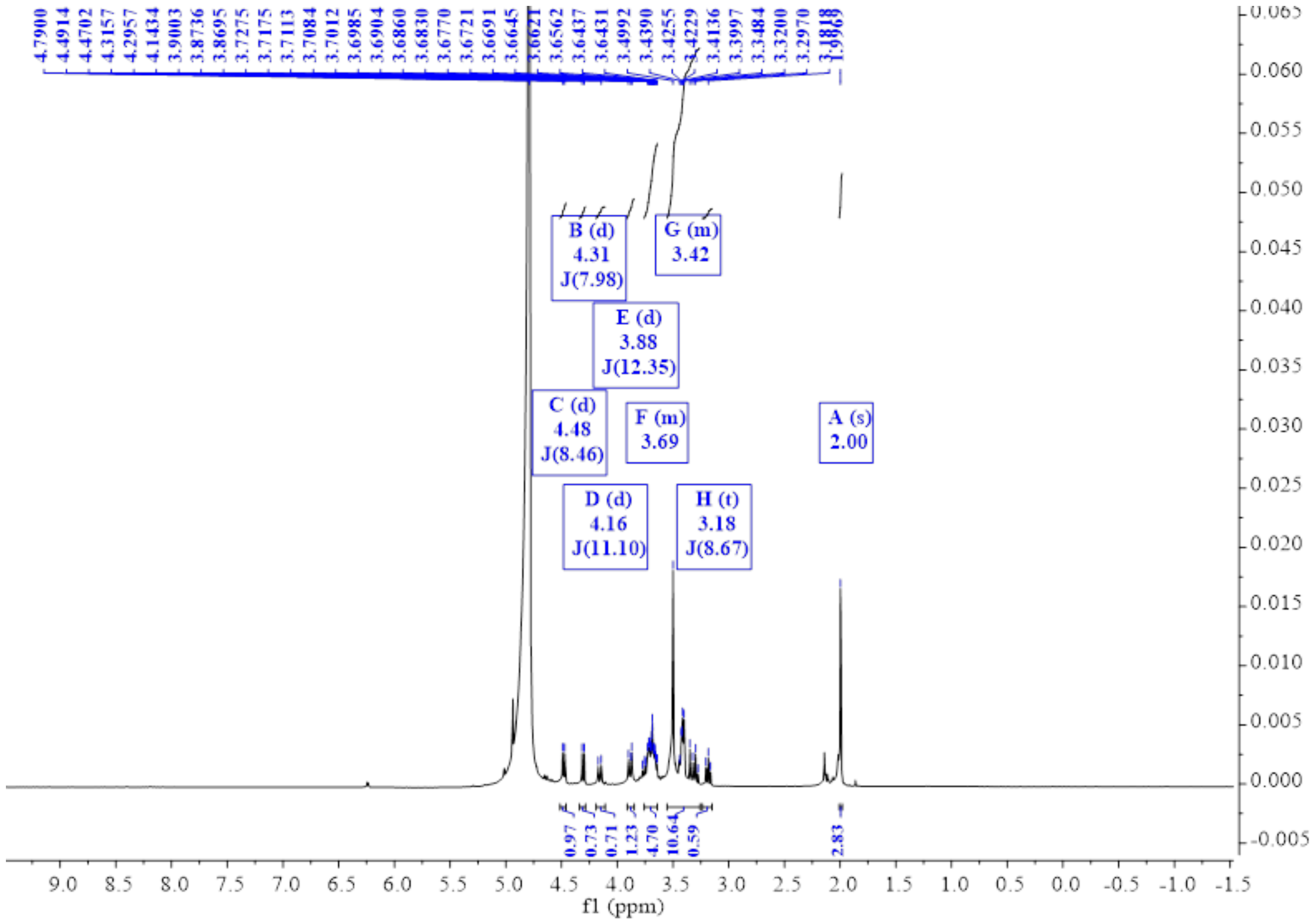

Compound **4m**, ^{13}C NMR (100 MHz, D_2O)

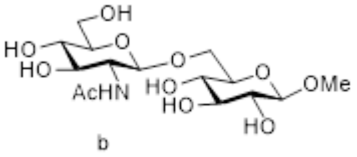

4m

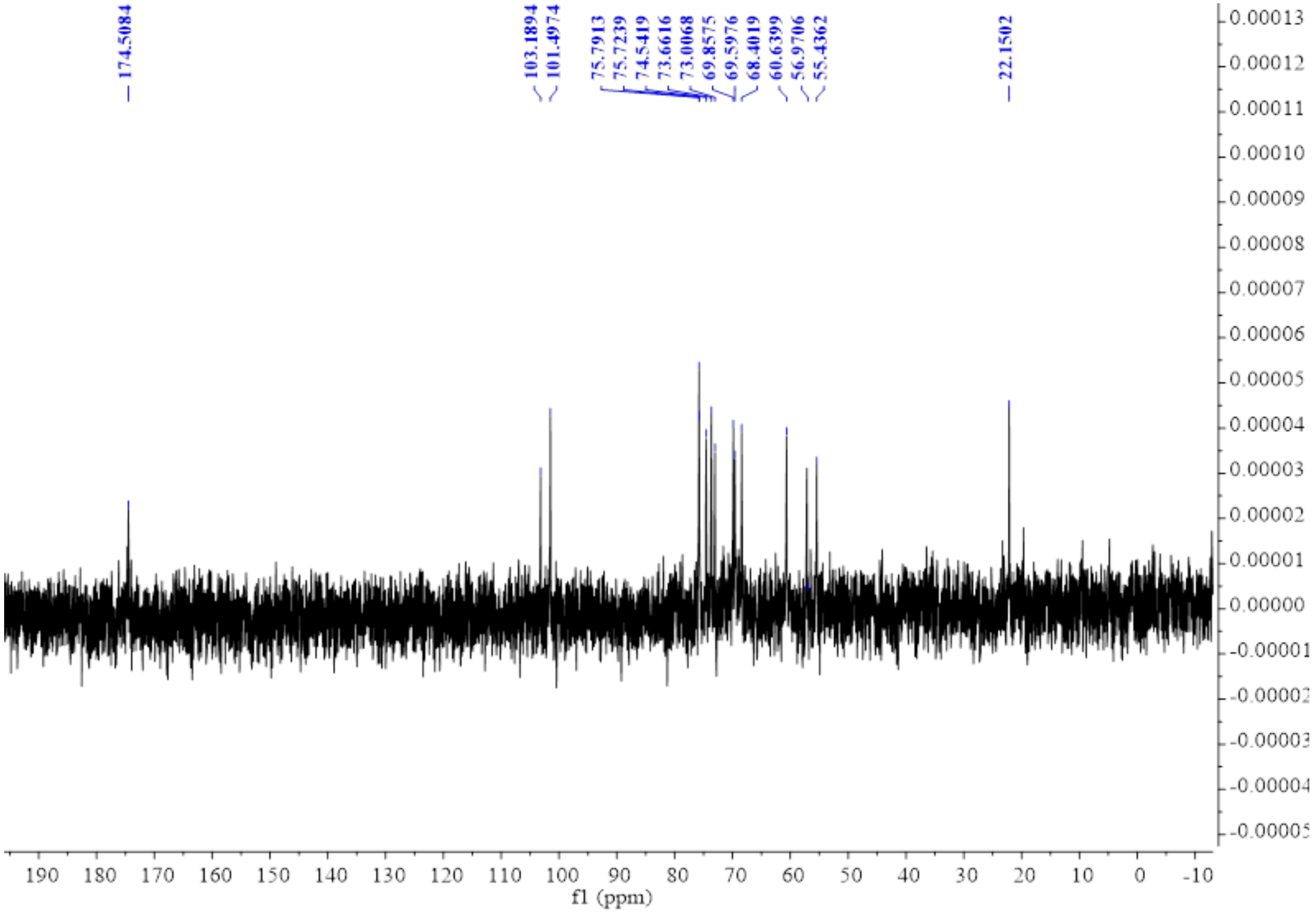

Compound **4m**, DEPT 135 (100 MHz, D_2O)

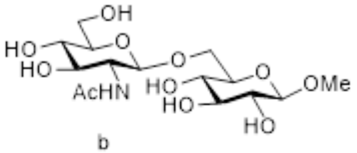

4m

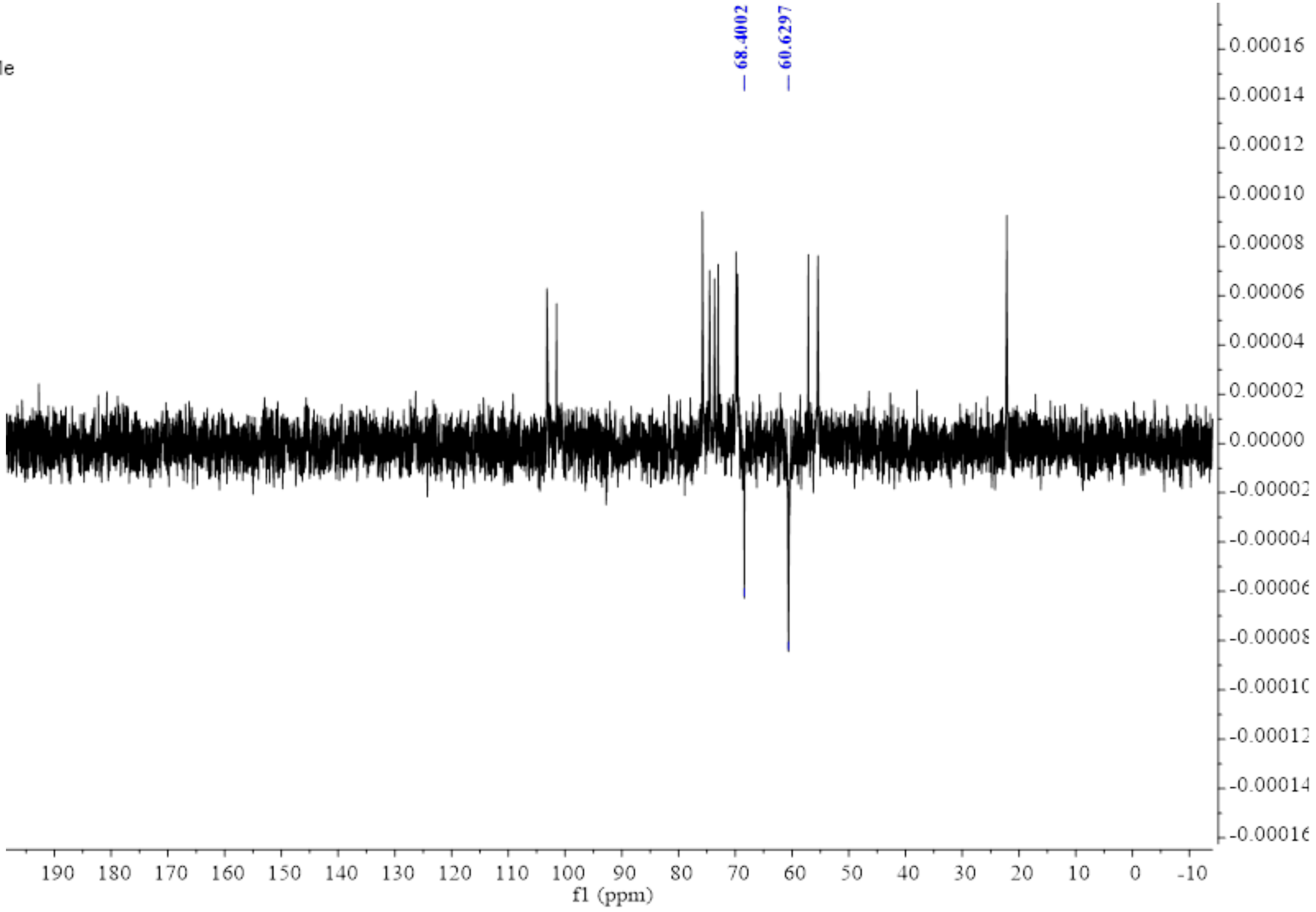

Compound **4m**, HMQC (400 MHz, D_2O)

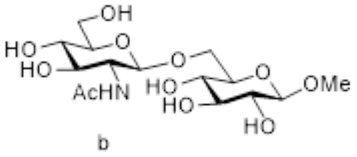

4m

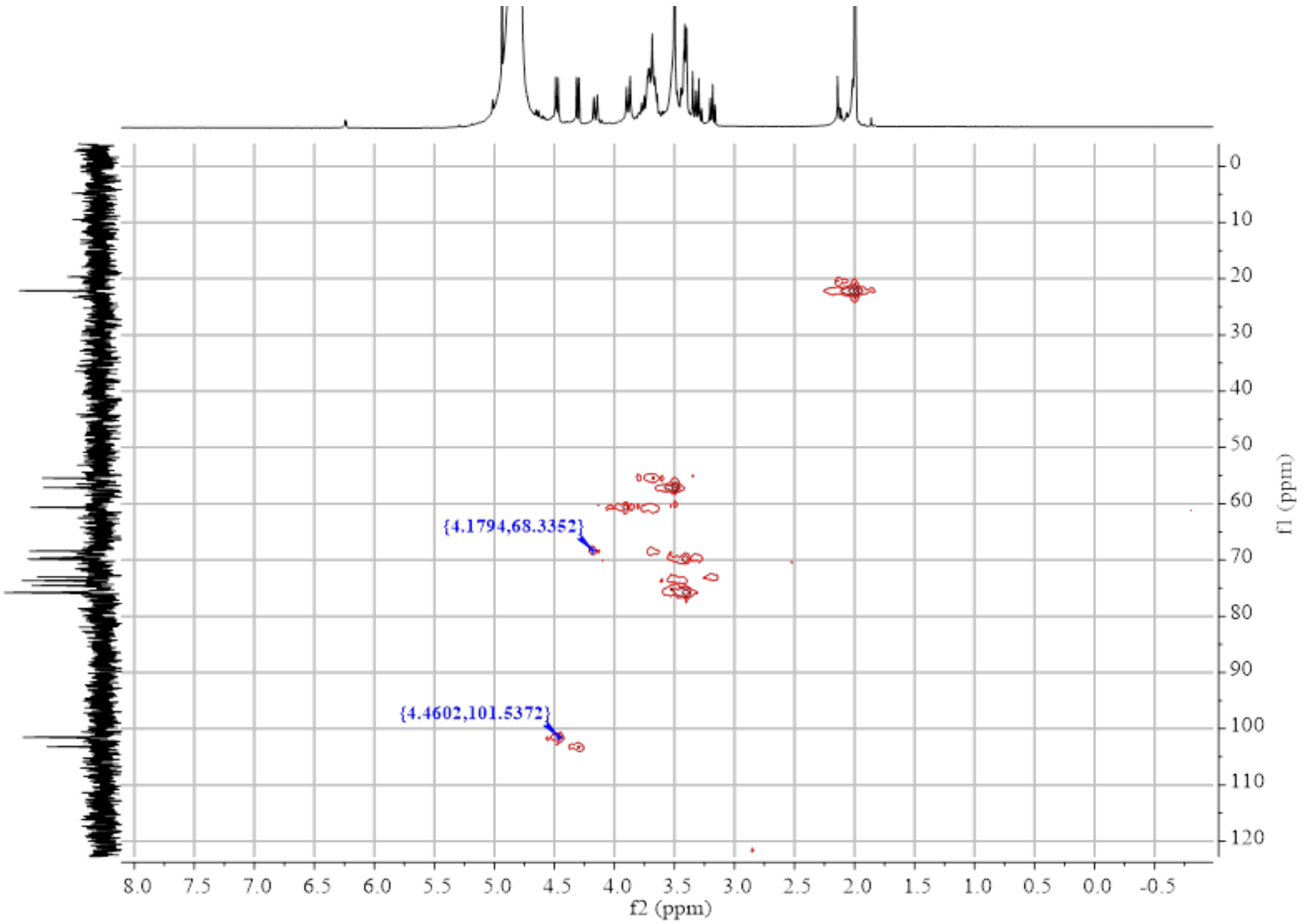

Compound **4m**, HMBC (400 MHz, D_2O)

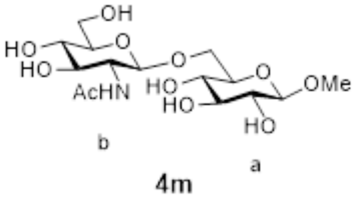

4m

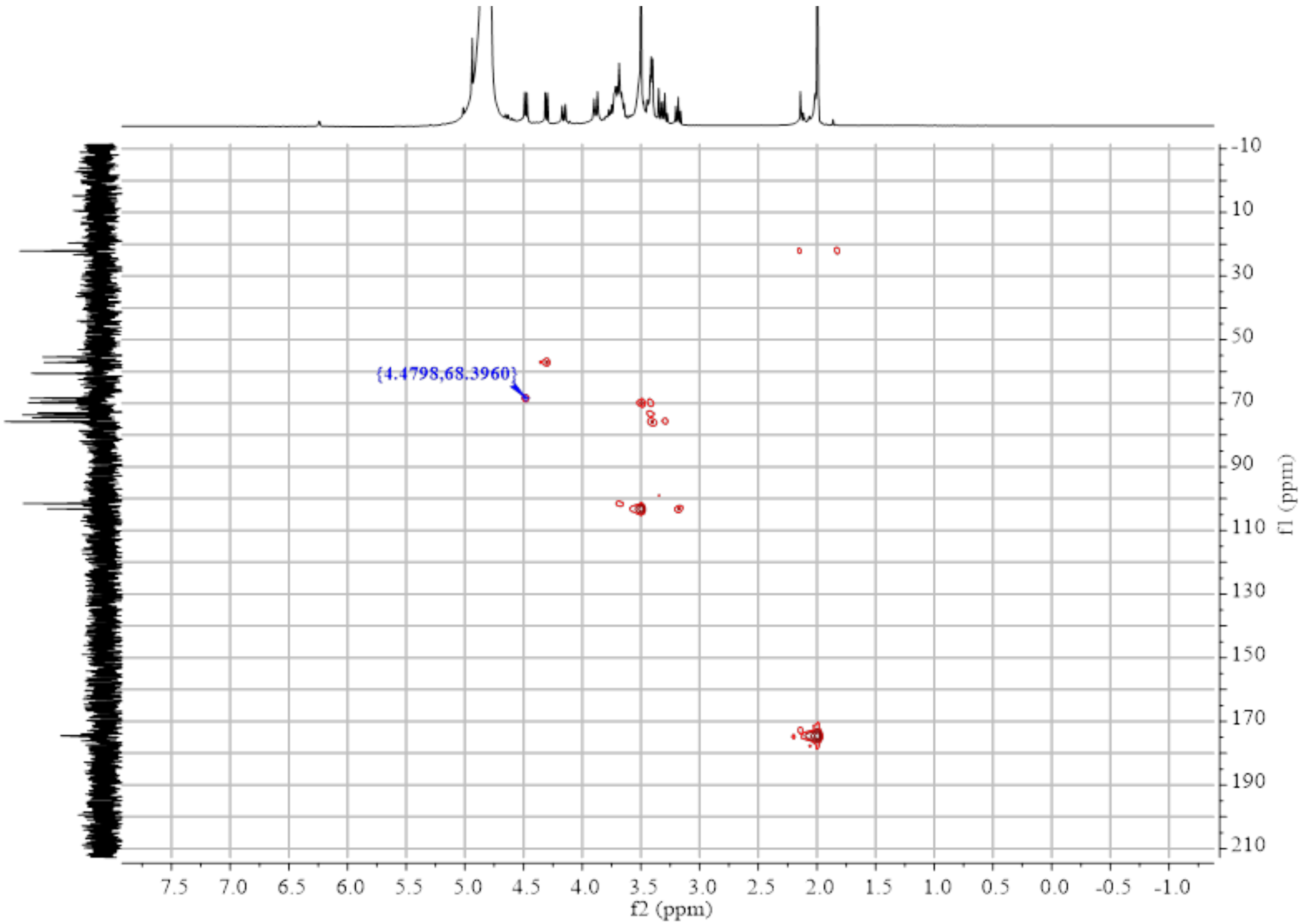

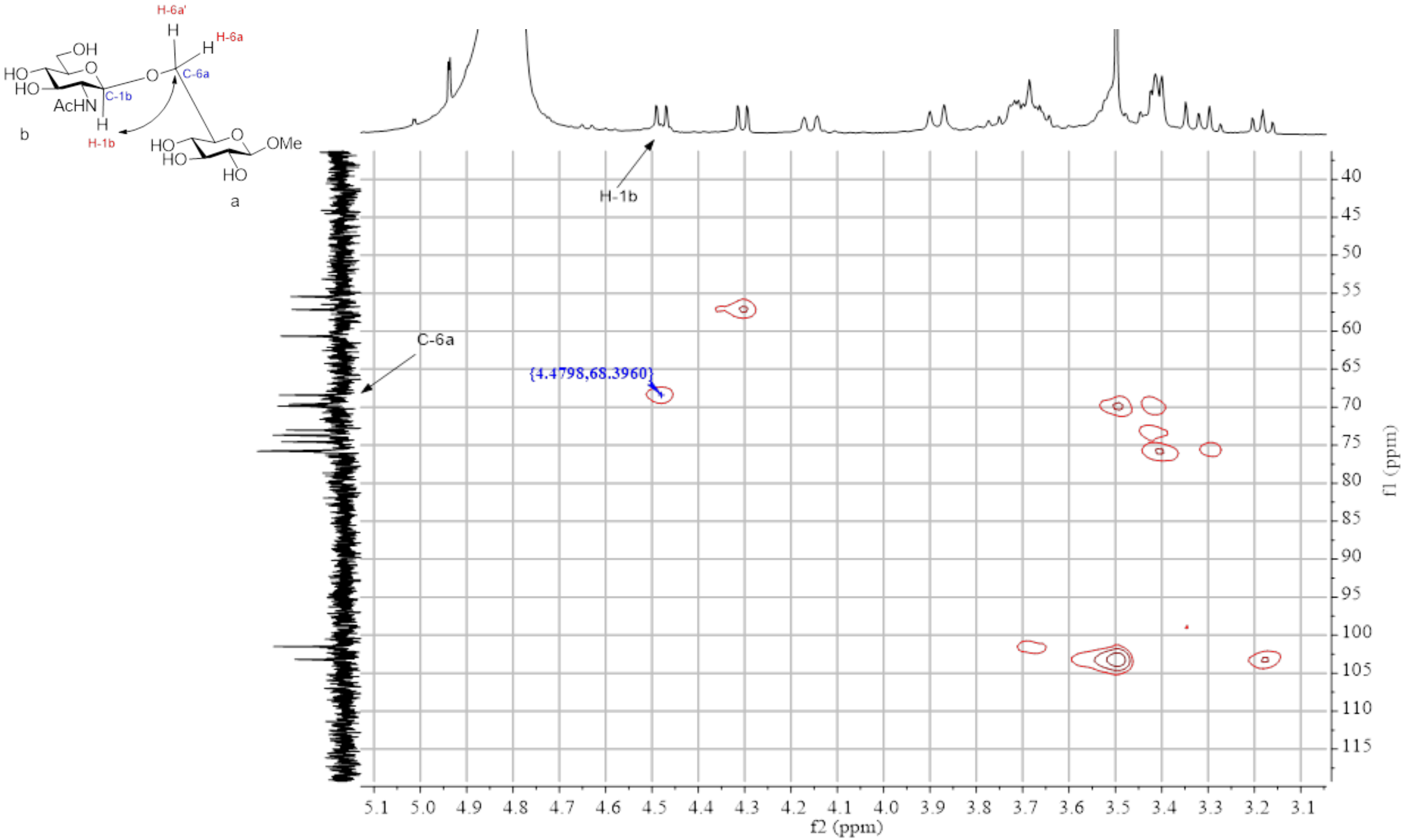
H-6a'
H-6a
OH
HO
HO
AcHN
O
O
C-6a
C-1b
H
H-1b
b
HO
HO
HO
OMe
a
H-1b
C-6a
{4.4798,68.3960}
f2 (ppm)
f1 (ppm)

***p*-Nitrophenyl 2-acetamido-2-deoxy-β-D-galactopyranosyl-(1→6)-β-D-glucopyranoside 4n**, ^{1}H NMR (600 MHz, DMSO-d6)

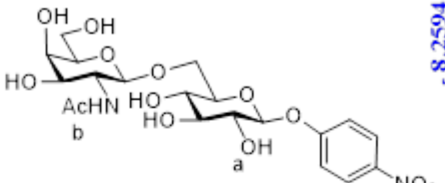

4n

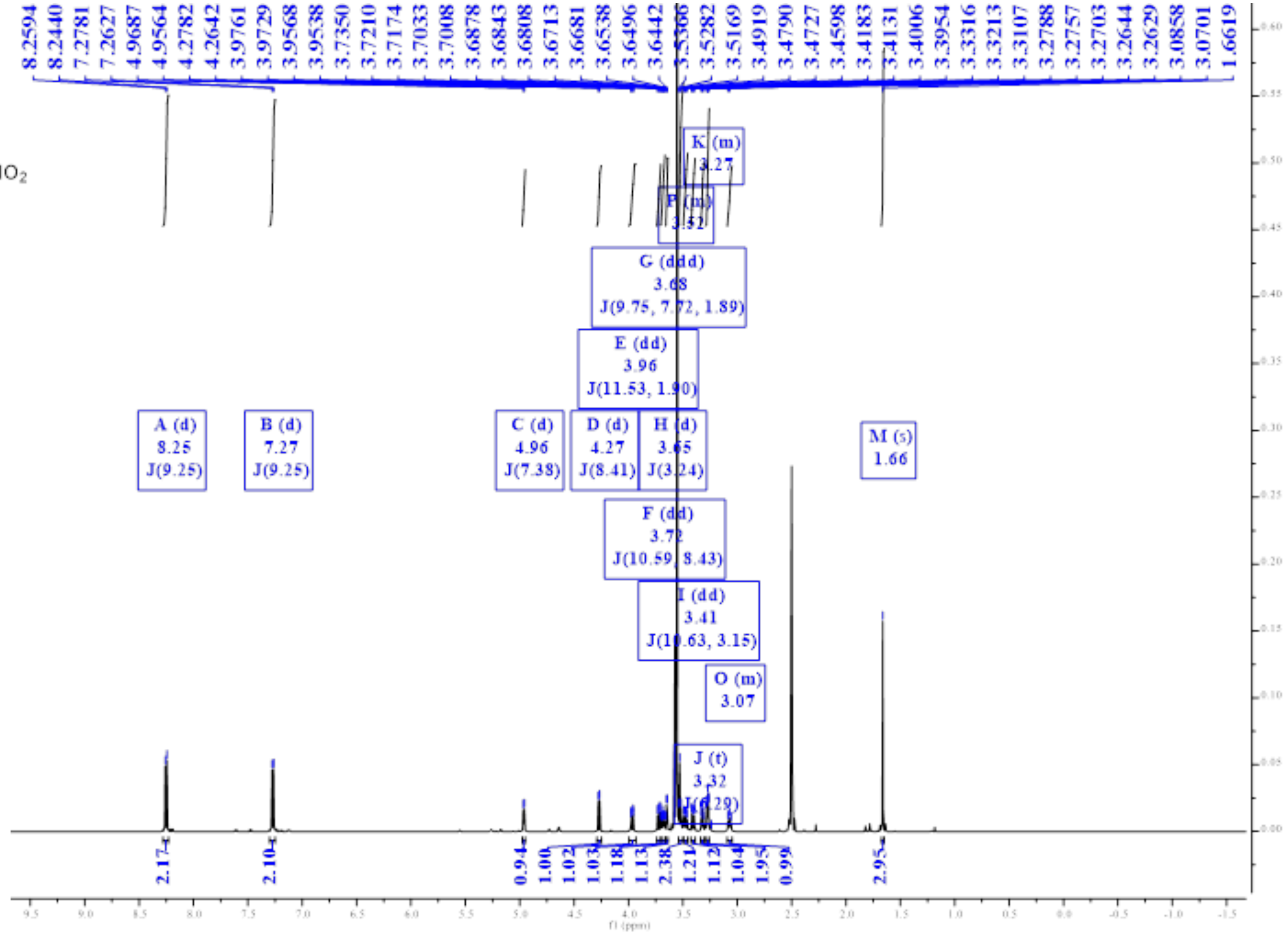

Compound **4n**, ^{13}C NMR (150 MHz, DMSO-d6)

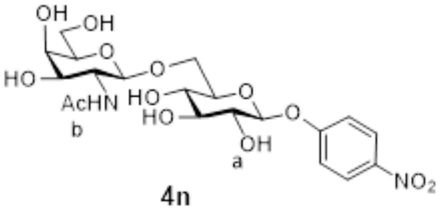

4n

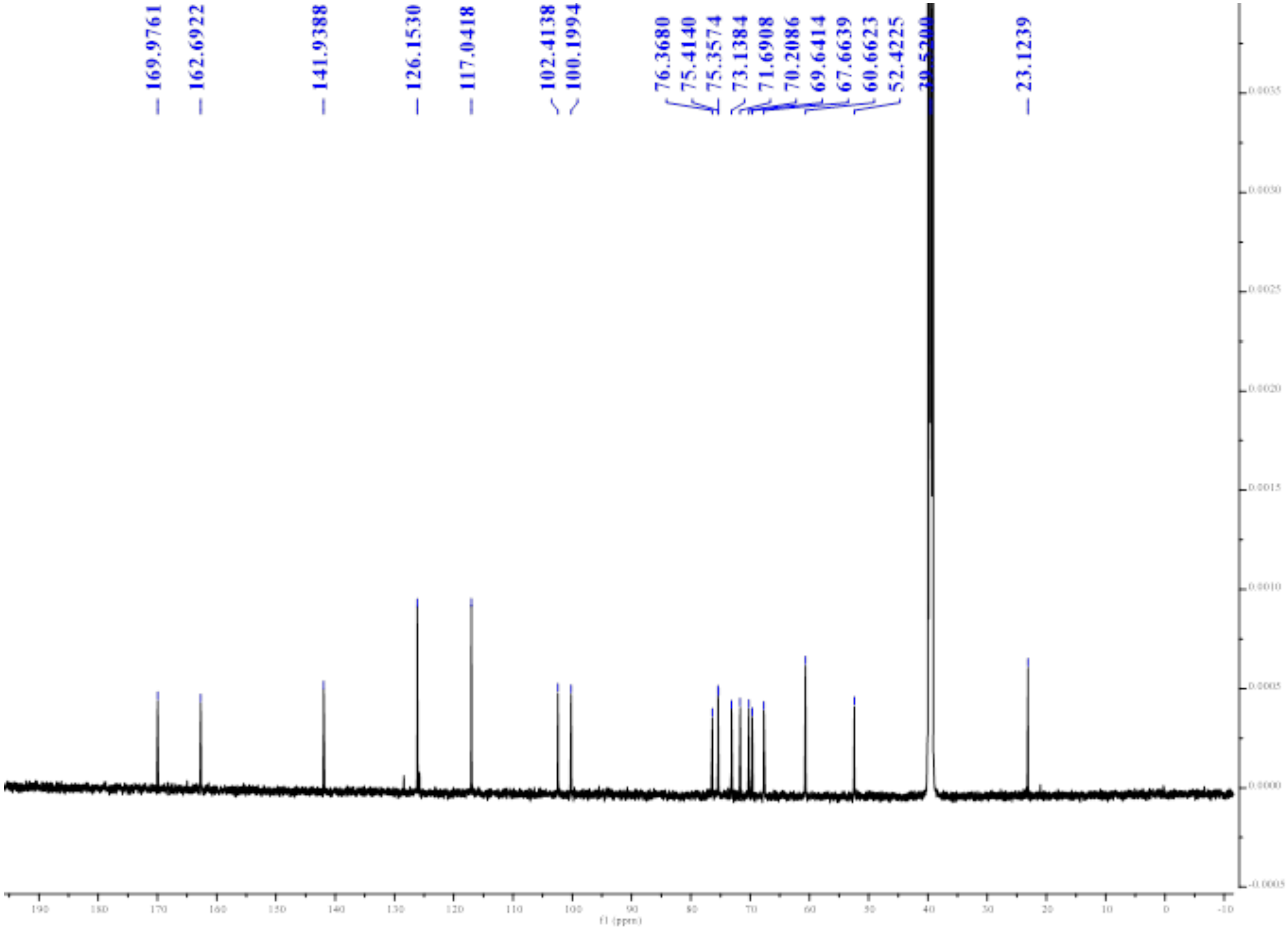

Compound **4n**, DEPT 135 (100 MHz, DMSO-d6)

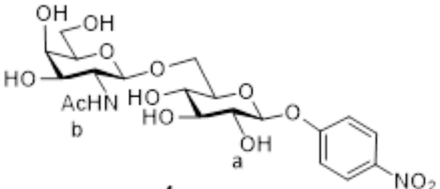

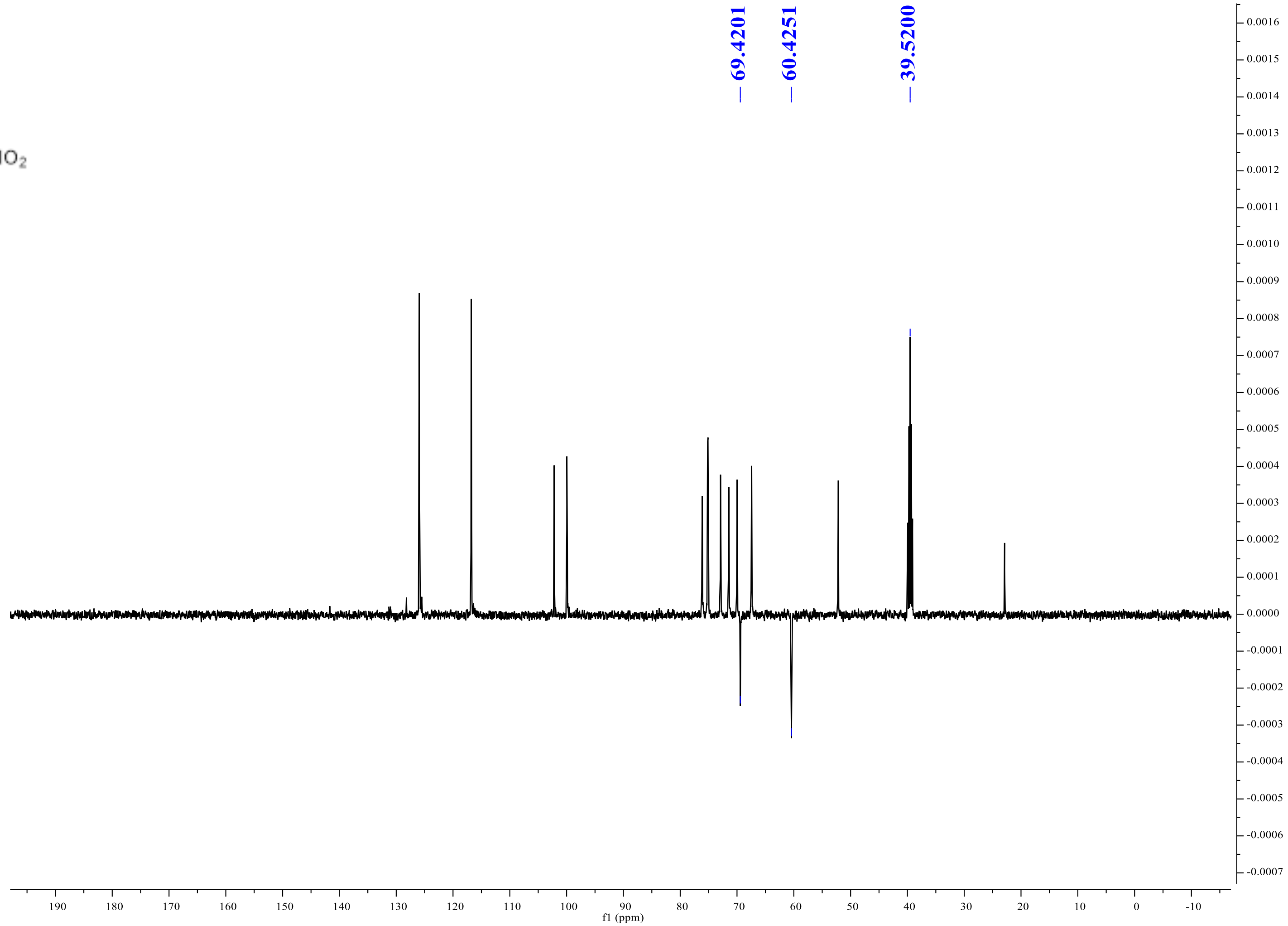

Compound **4n**, HMQC (600 MHz, DMSO-d6)

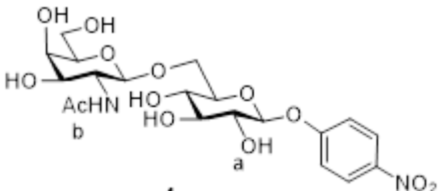

4n

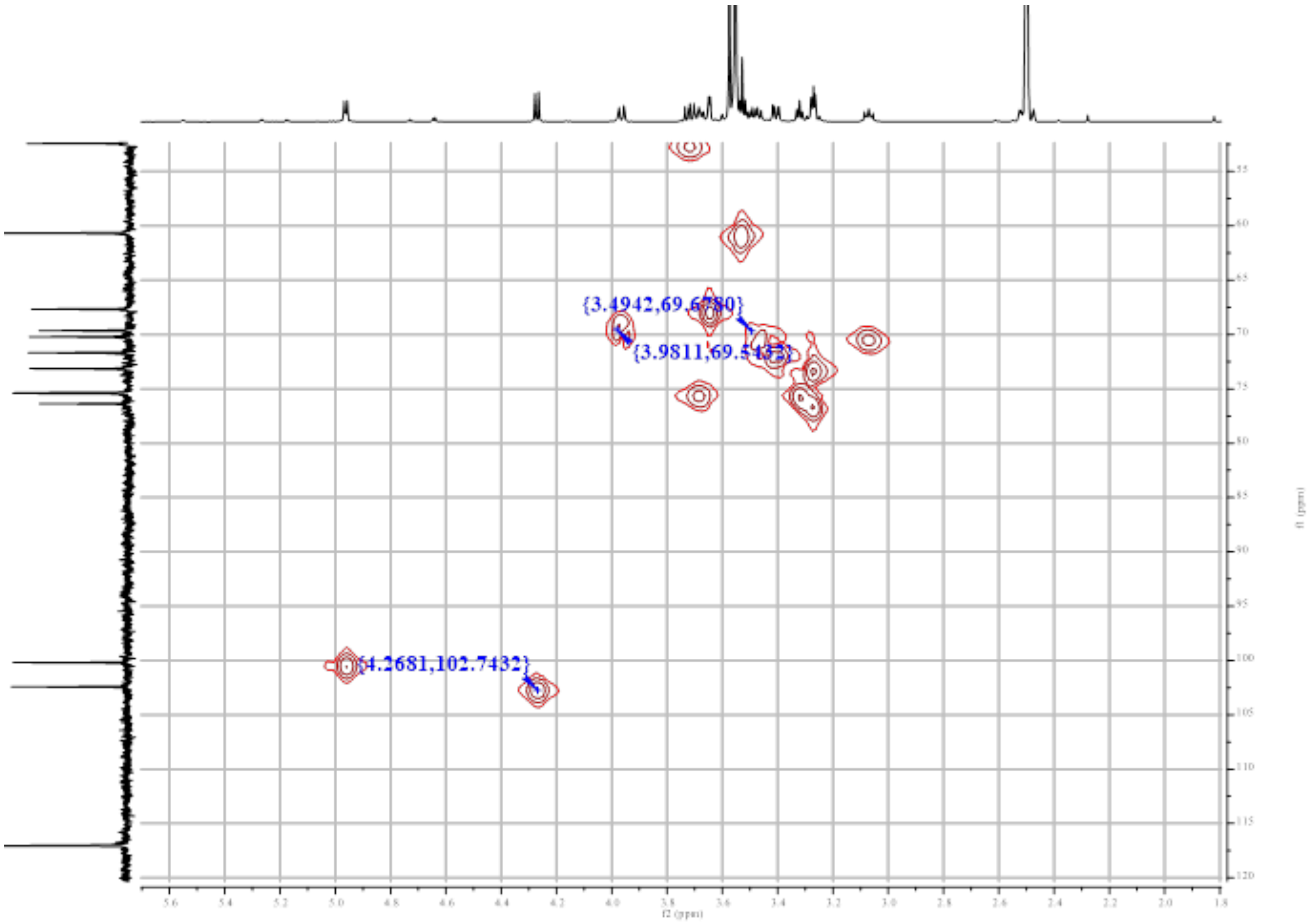

Compound **4n**, HMBC (600 MHz, DMSO-d6)

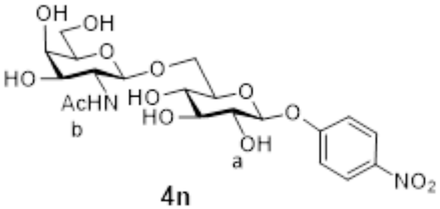

4n

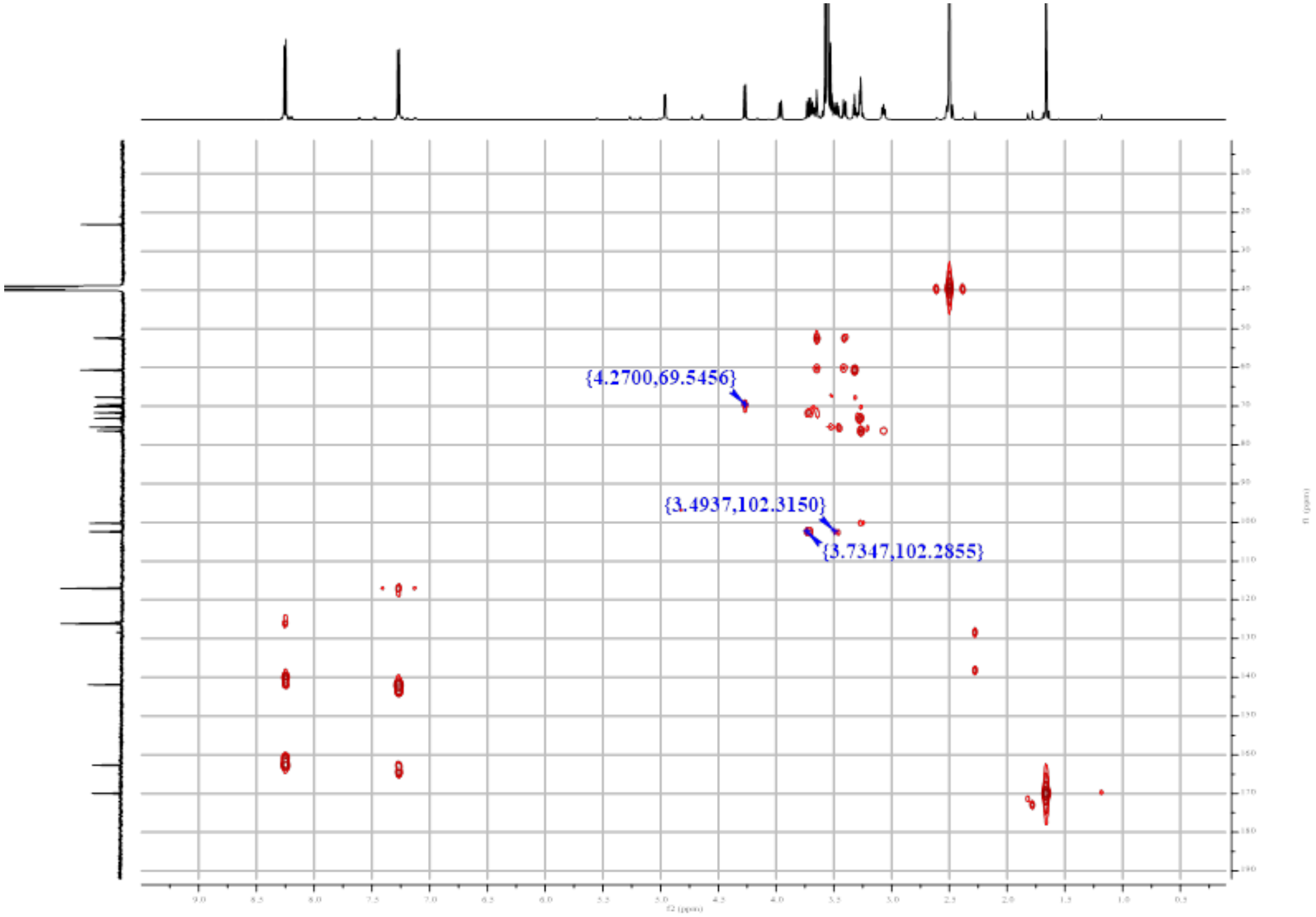

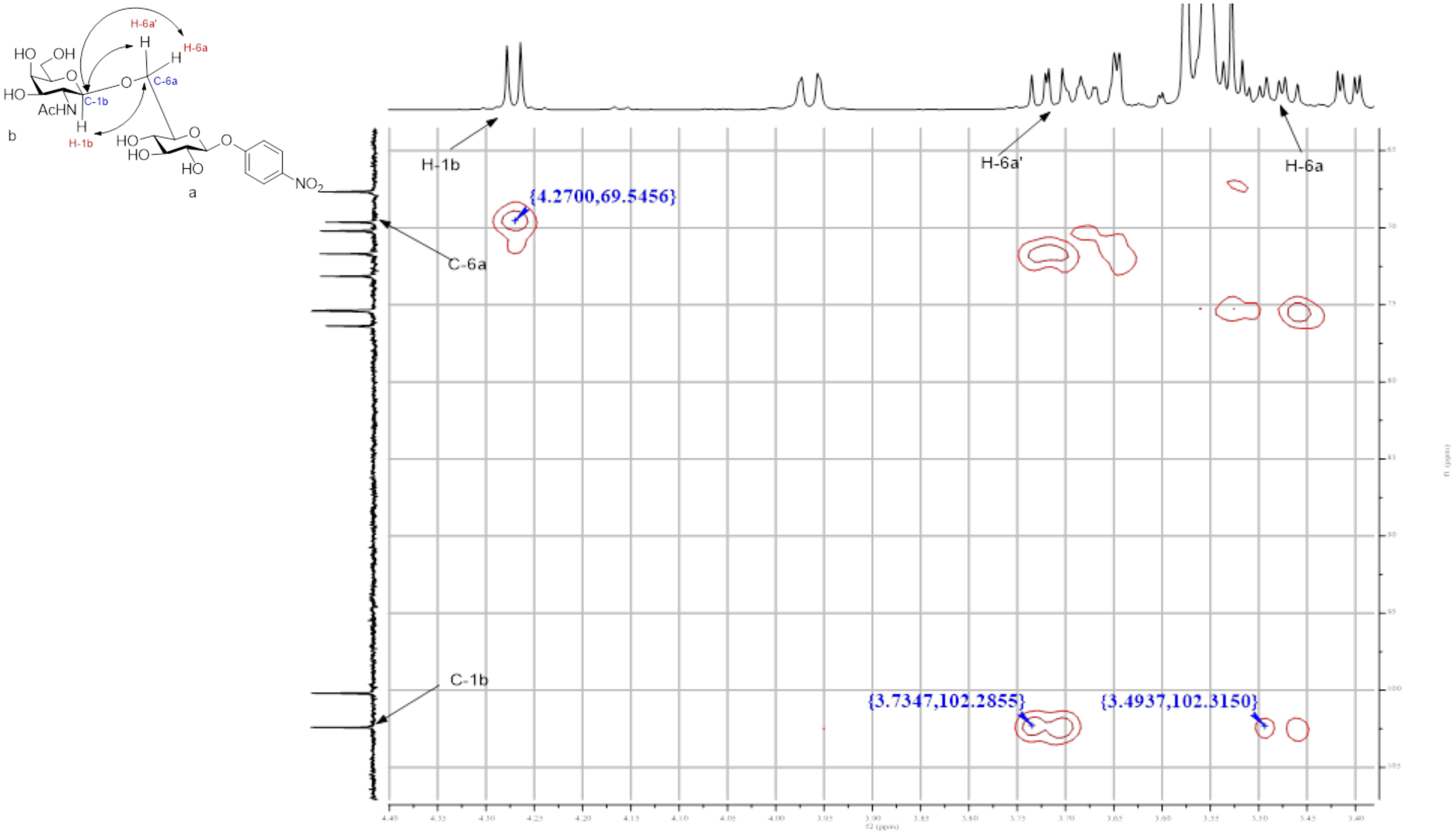
H-6a'
H-6a
H
H
HO
OH
HO
O
O
C-6a
C-1b
AcHN
H
b
H-1b
HO
O
O
HO
HO
NO2
a
H-1b
H-6a'
H-6a
{4.2700,69.5456}
C-6a
C-1b
{3.7347,102.2855}
{3.4937,102.3150}

***p*-Nitrophenyl 2-acetamido-2-deoxy-β-D-galactopyranosyl-(1→6)-β-D-galactopyranoside 4o**, ^{1}H NMR (400 MHz, D_2O)

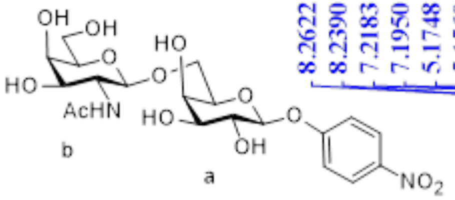

4o

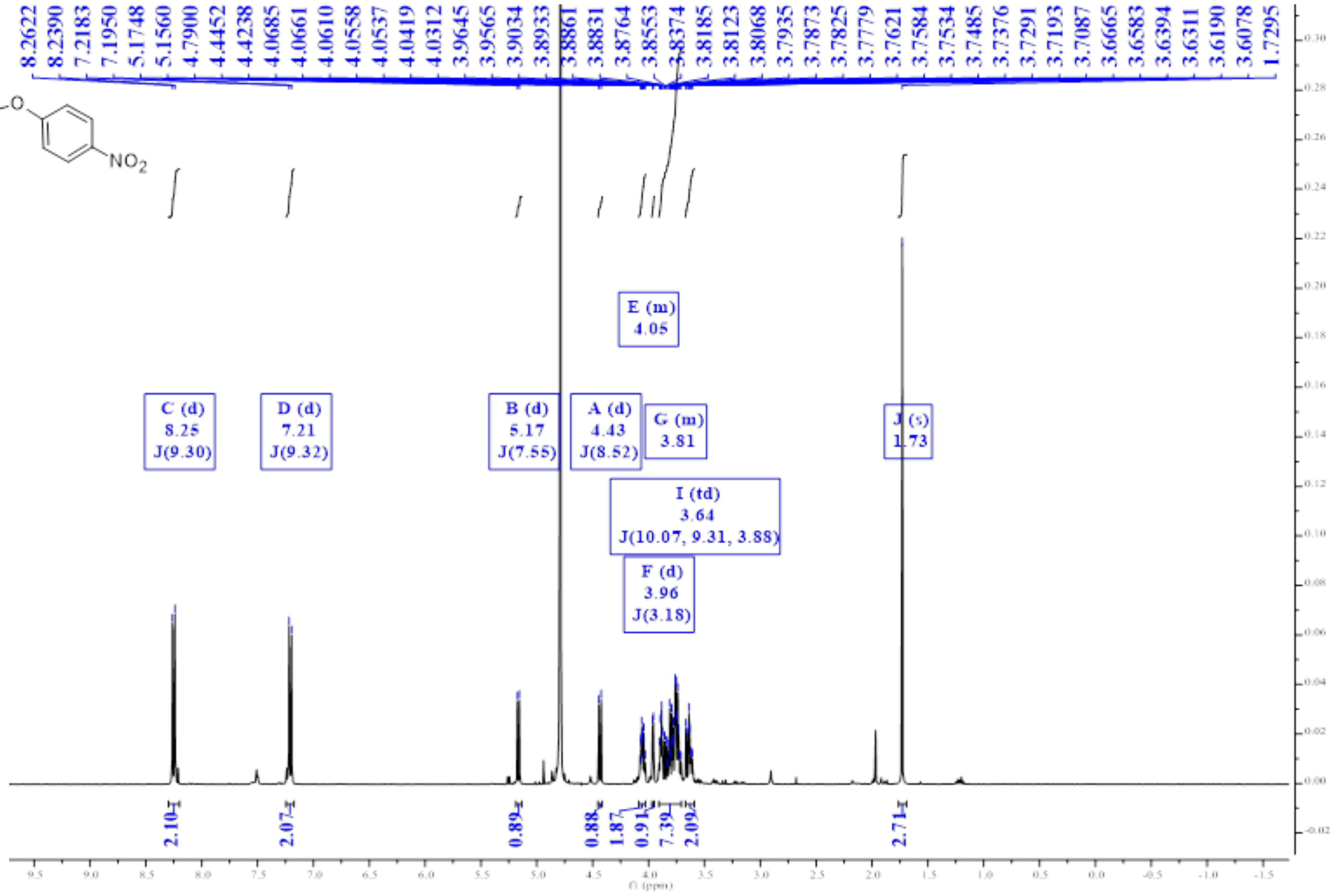

Compound **4o**, DEPT 135 NMR (100 MHz, D_2O)

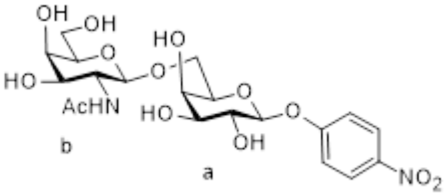

4o

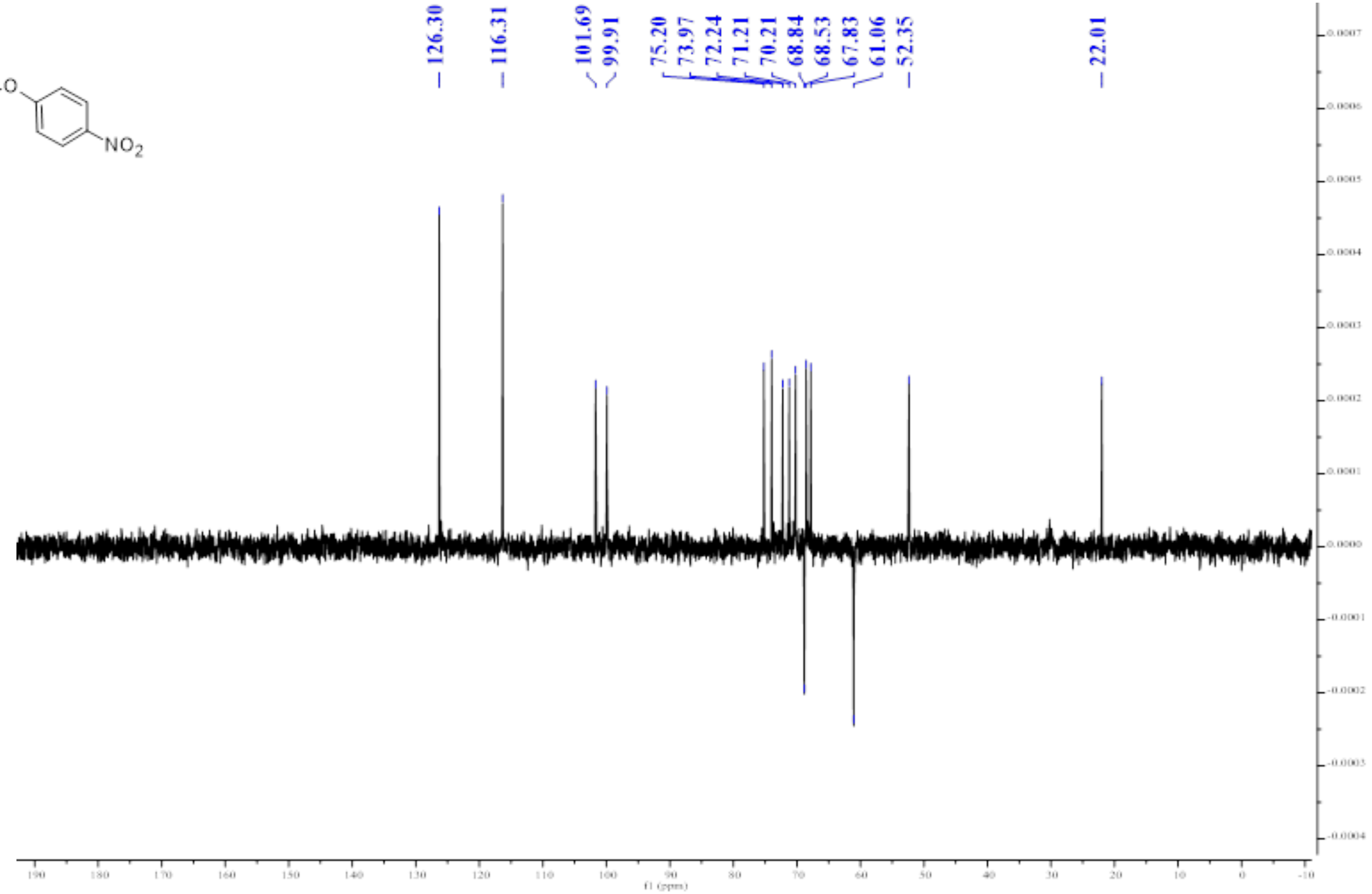

Compound **4o**, ^{13}C NMR (150 MHz, D_2O)

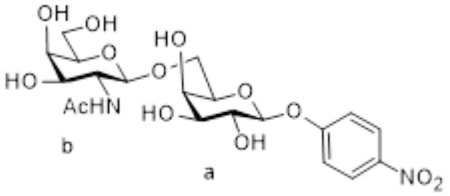

4o

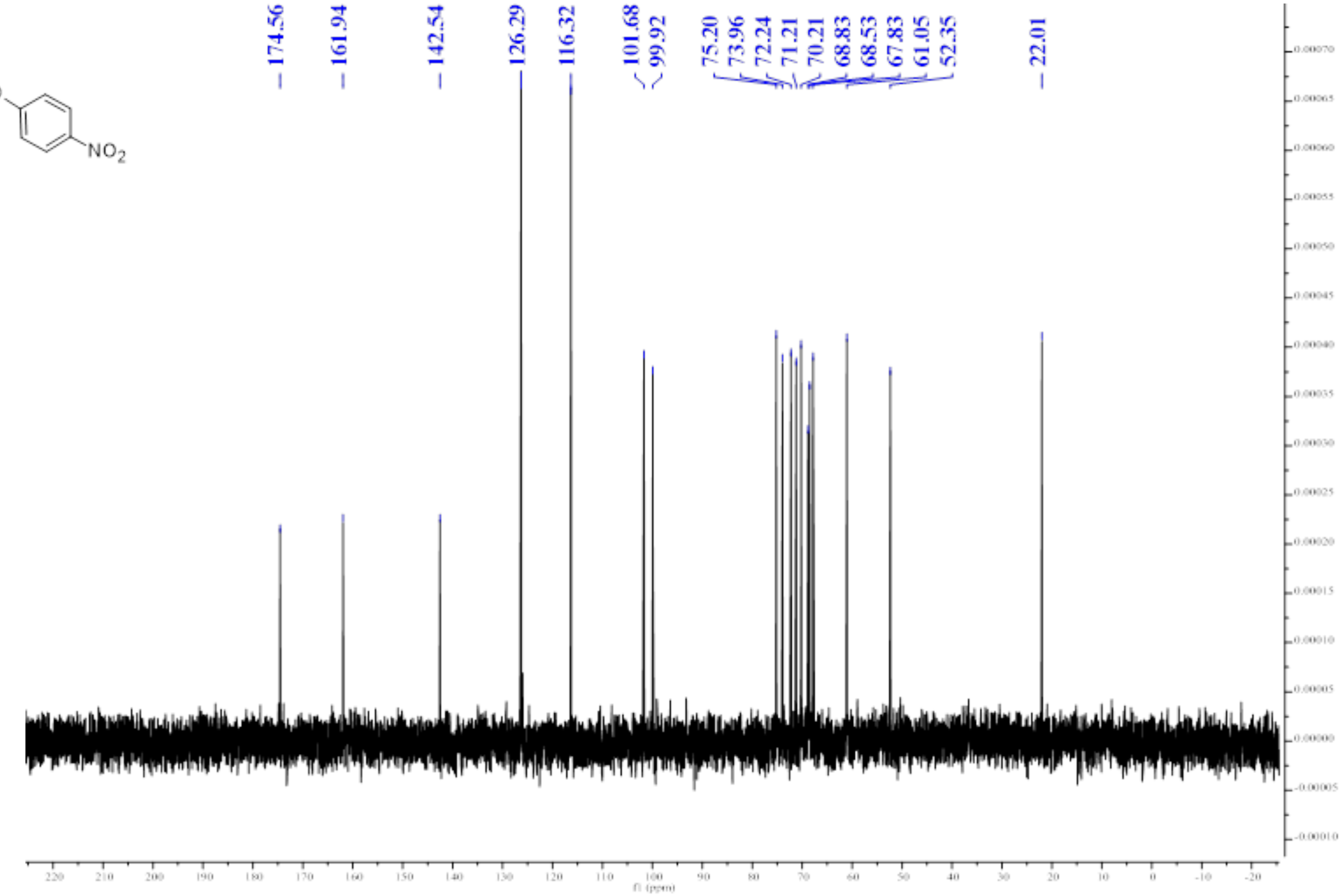

Compound **4o**, HMQC (600 MHz, D_2O)

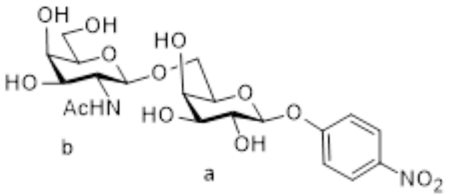

4o

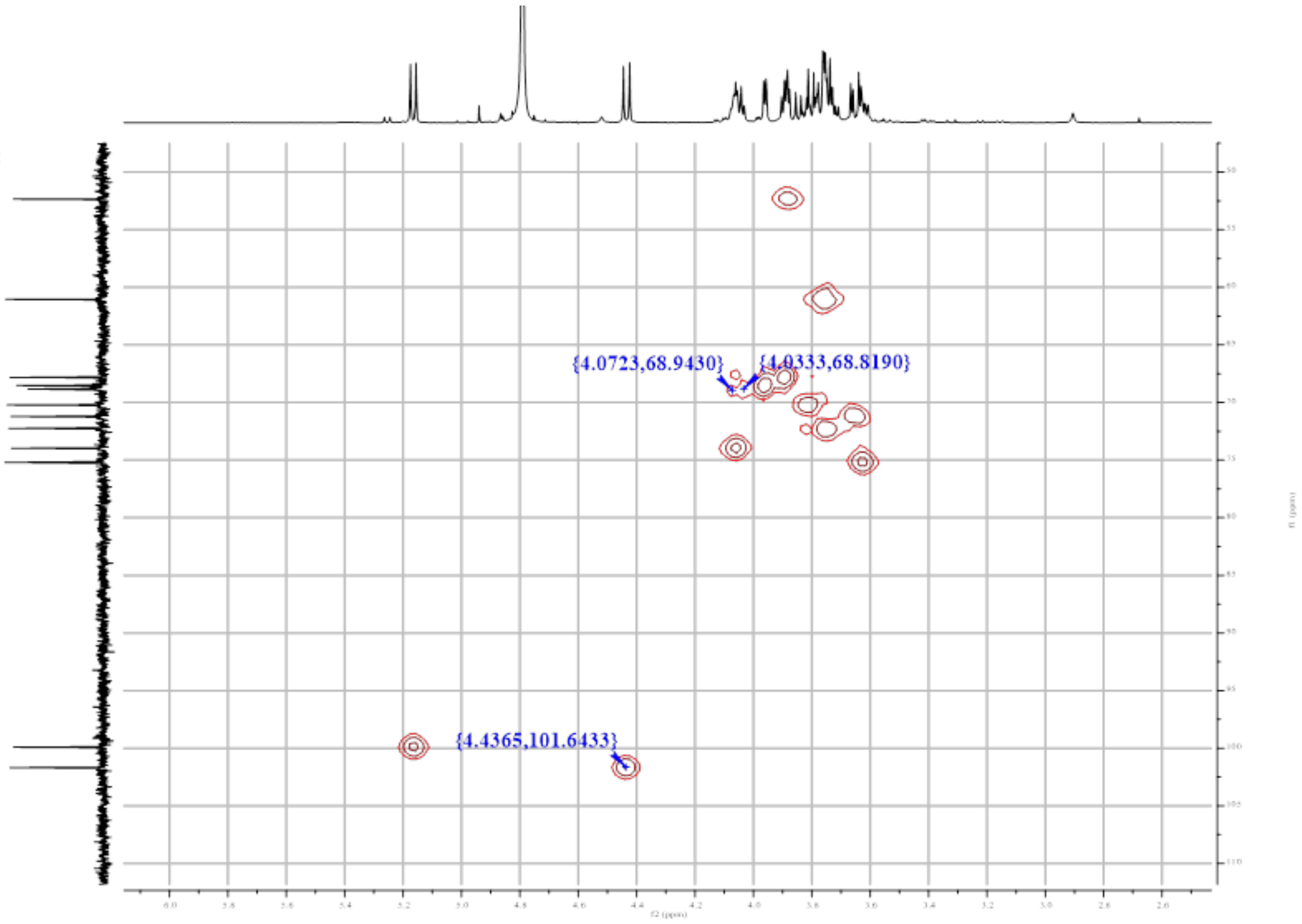

Compound **4o**, HMBC (600 MHz, D_2O)

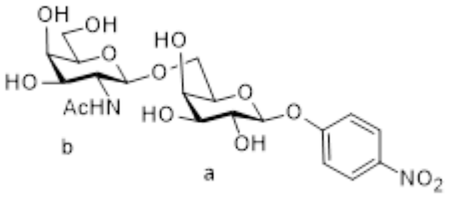

4o

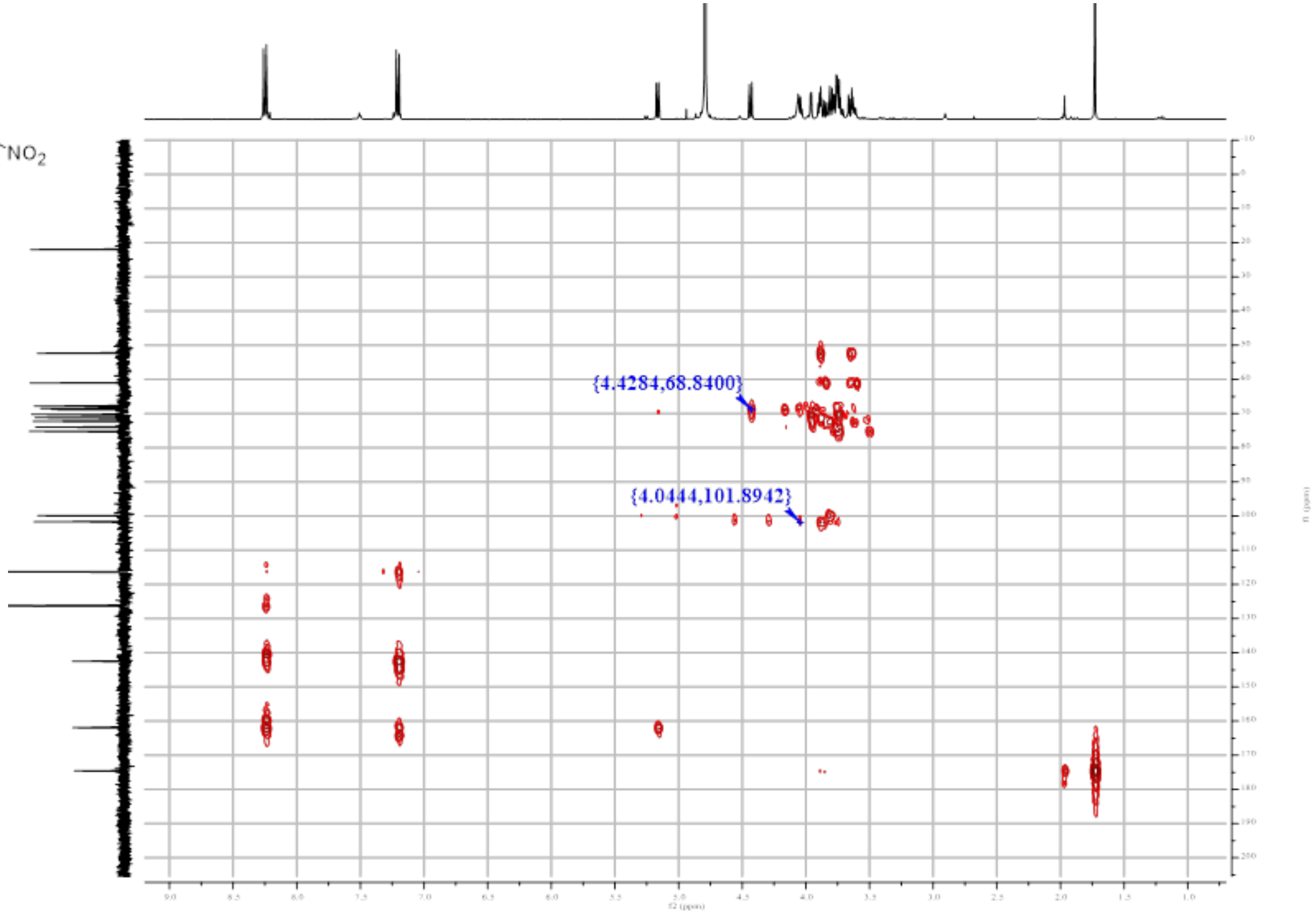

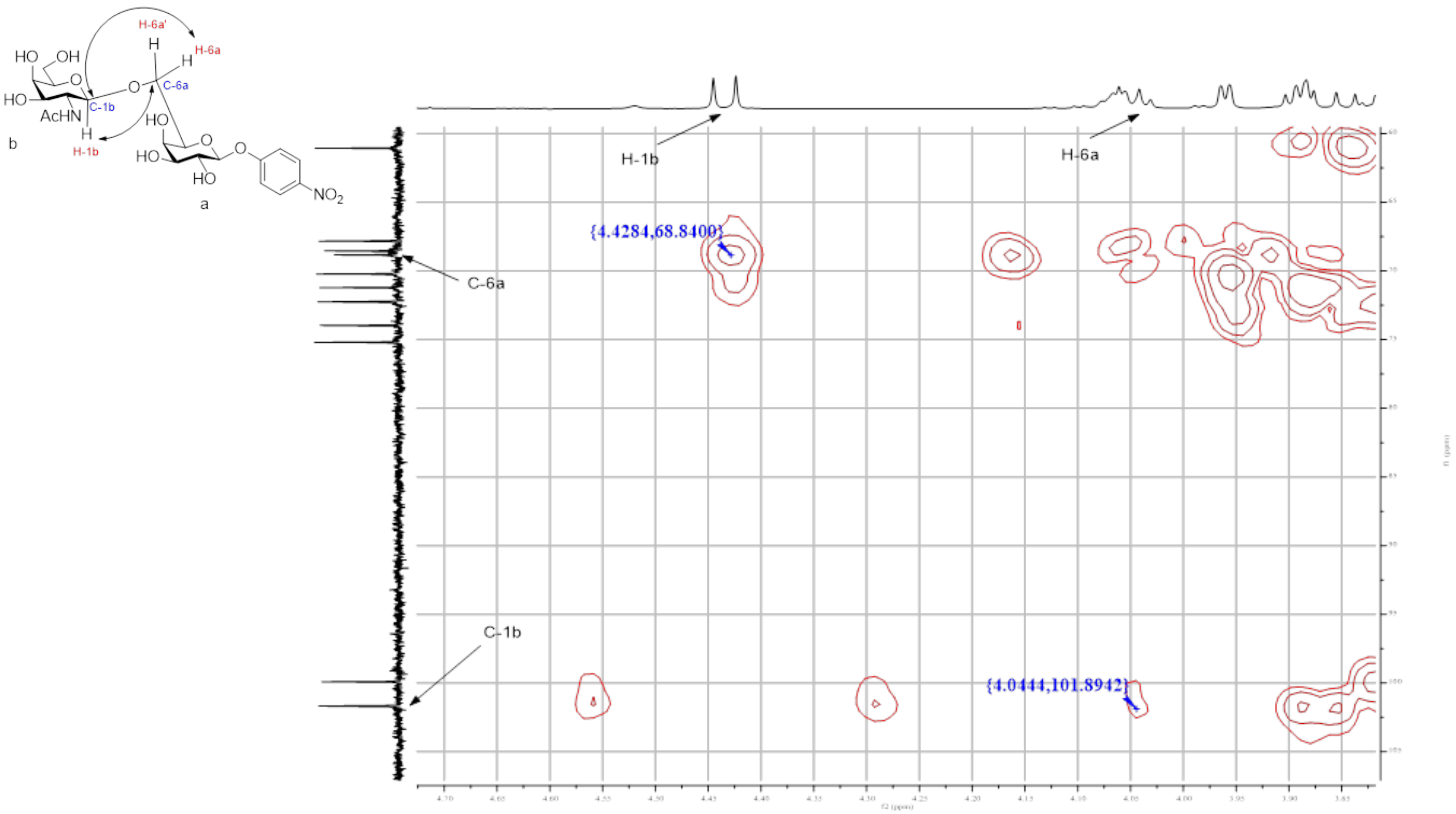

H-6a'
H-6a
H
HO
OH
O
O
C-6a
HO
C-1b
AcHN
H
b
H-1b
HO
HO
O
O
HO
NO2
a
H-1b
H-6a
{4.4284,68.8400}
C-6a
C-1b
{4.0444,101.8942}

***p*-Nitrophenyl 2-acetamido-2-deoxy-α-D-mannopyranosyl-(1→6)-β-D-glucopyranoside 4p**, ^{1}H NMR (400 MHz, CD_3OD)

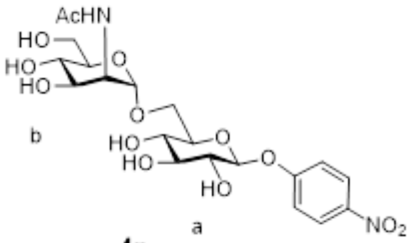

4p

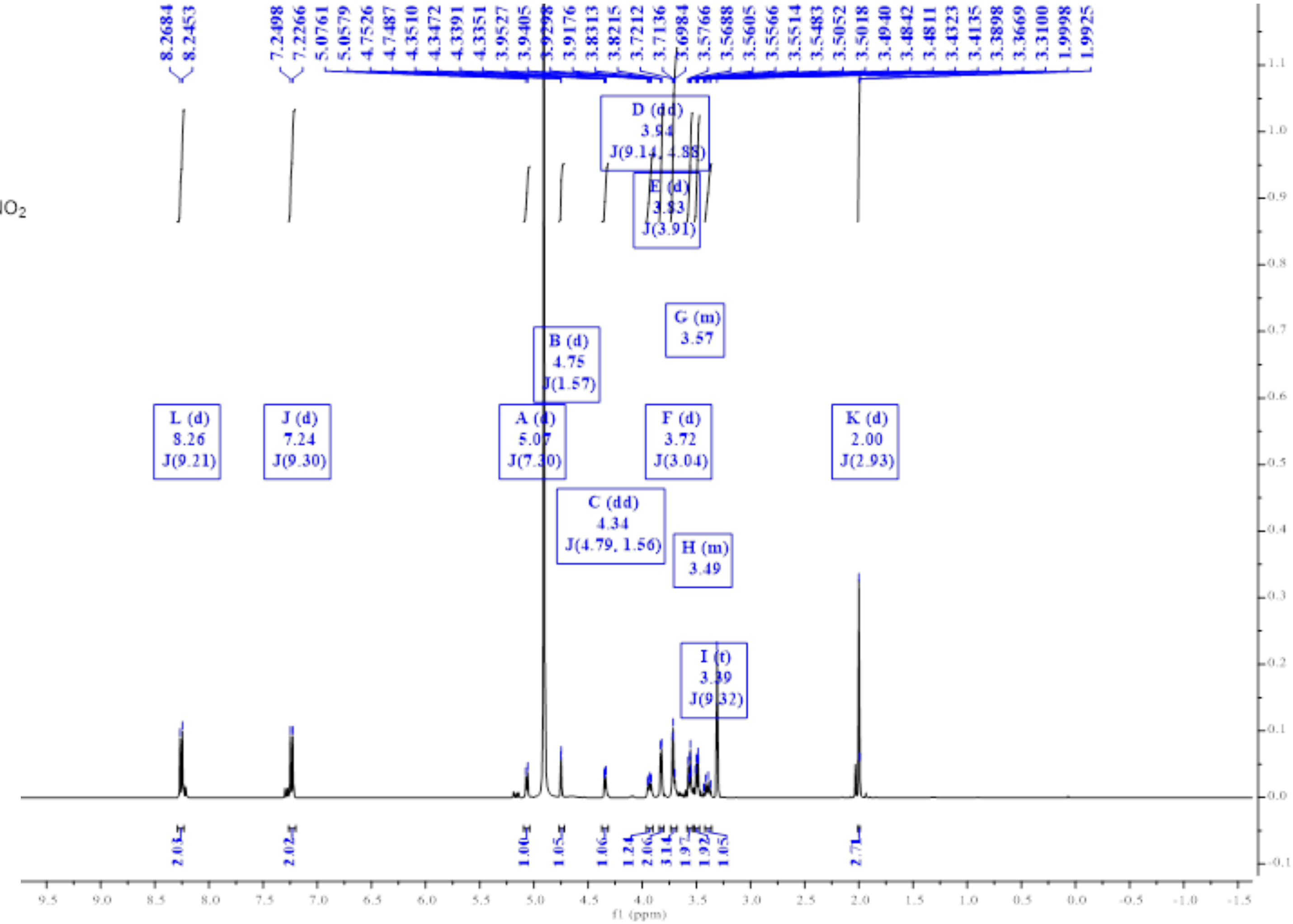

Compound **4p**, ^{13}C NMR (150 MHz, CD_3OD)

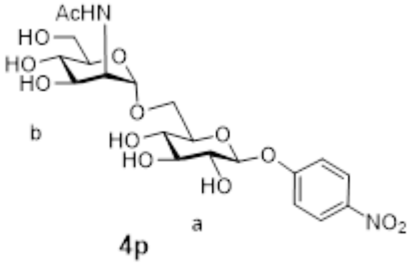

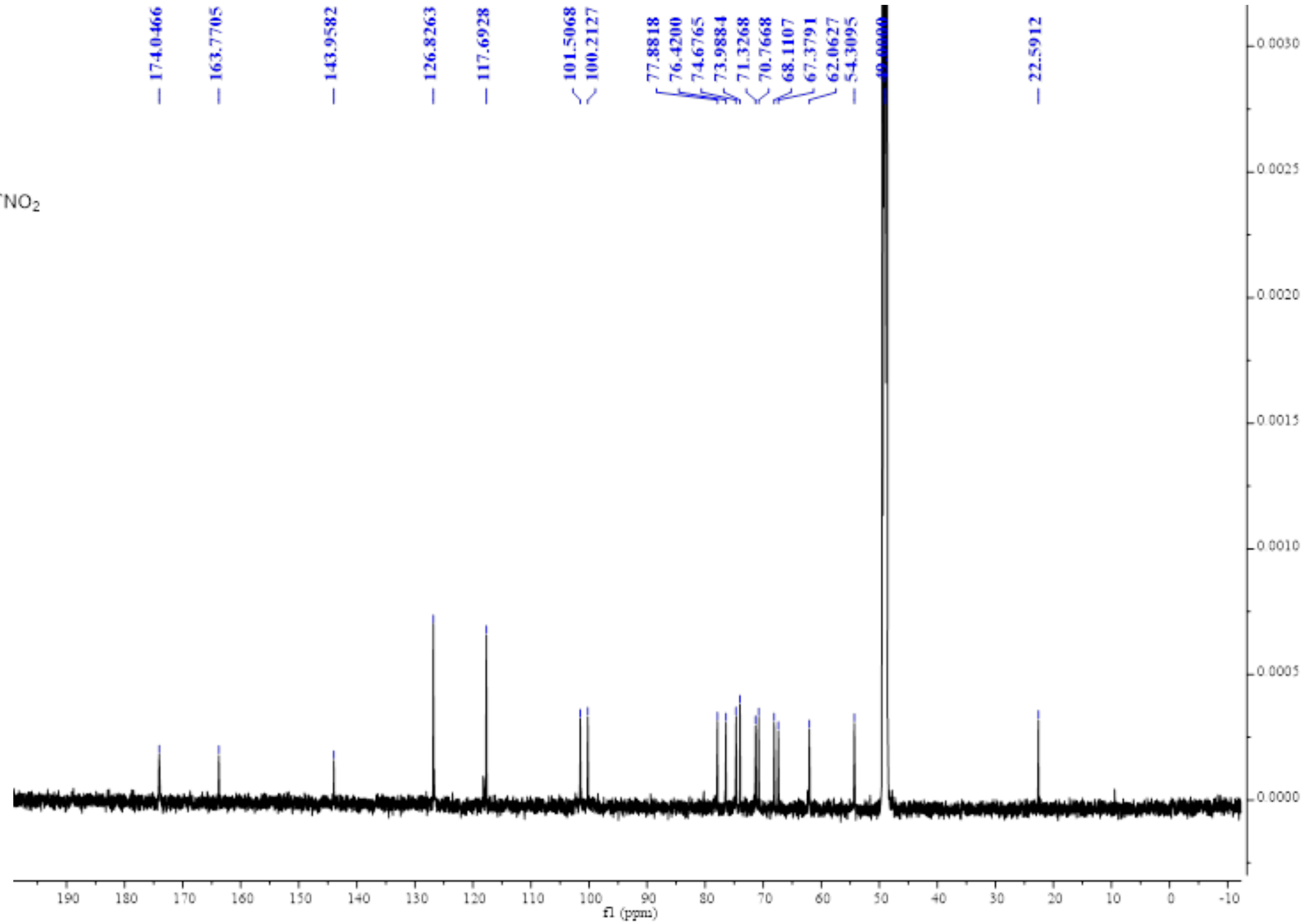

Compound **4p**, DEPT 135 (150 MHz, CD_3OD)

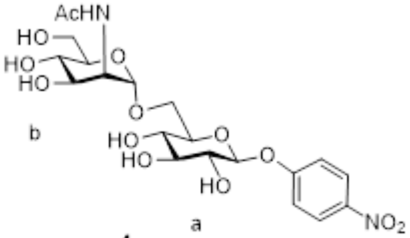

4p

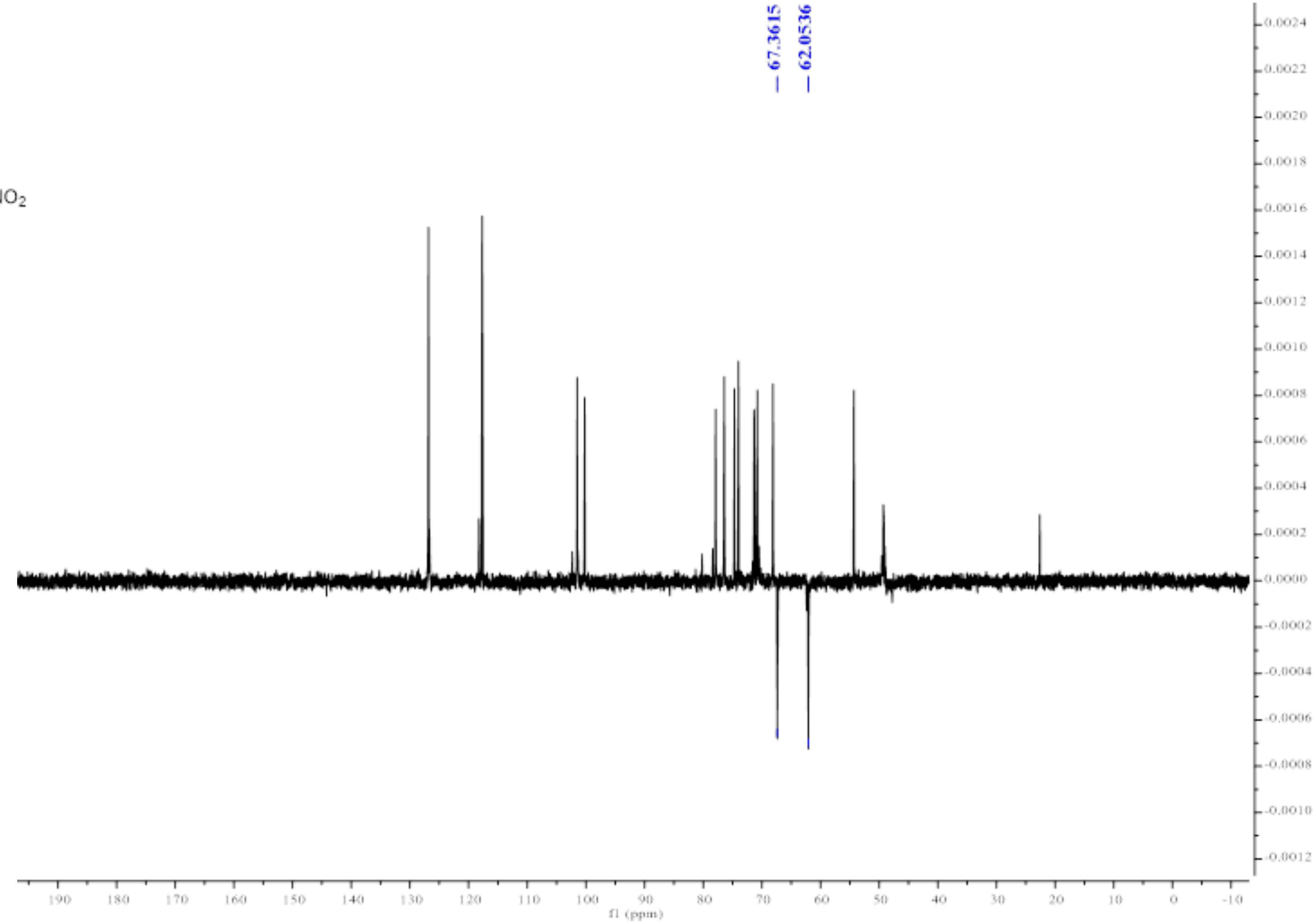

Compound **4p**, HMQC (150 MHz, CD_3OD)

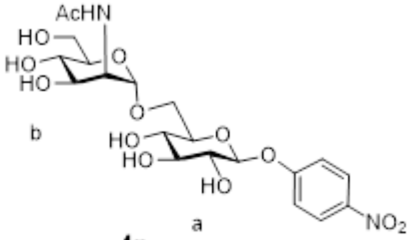

4p

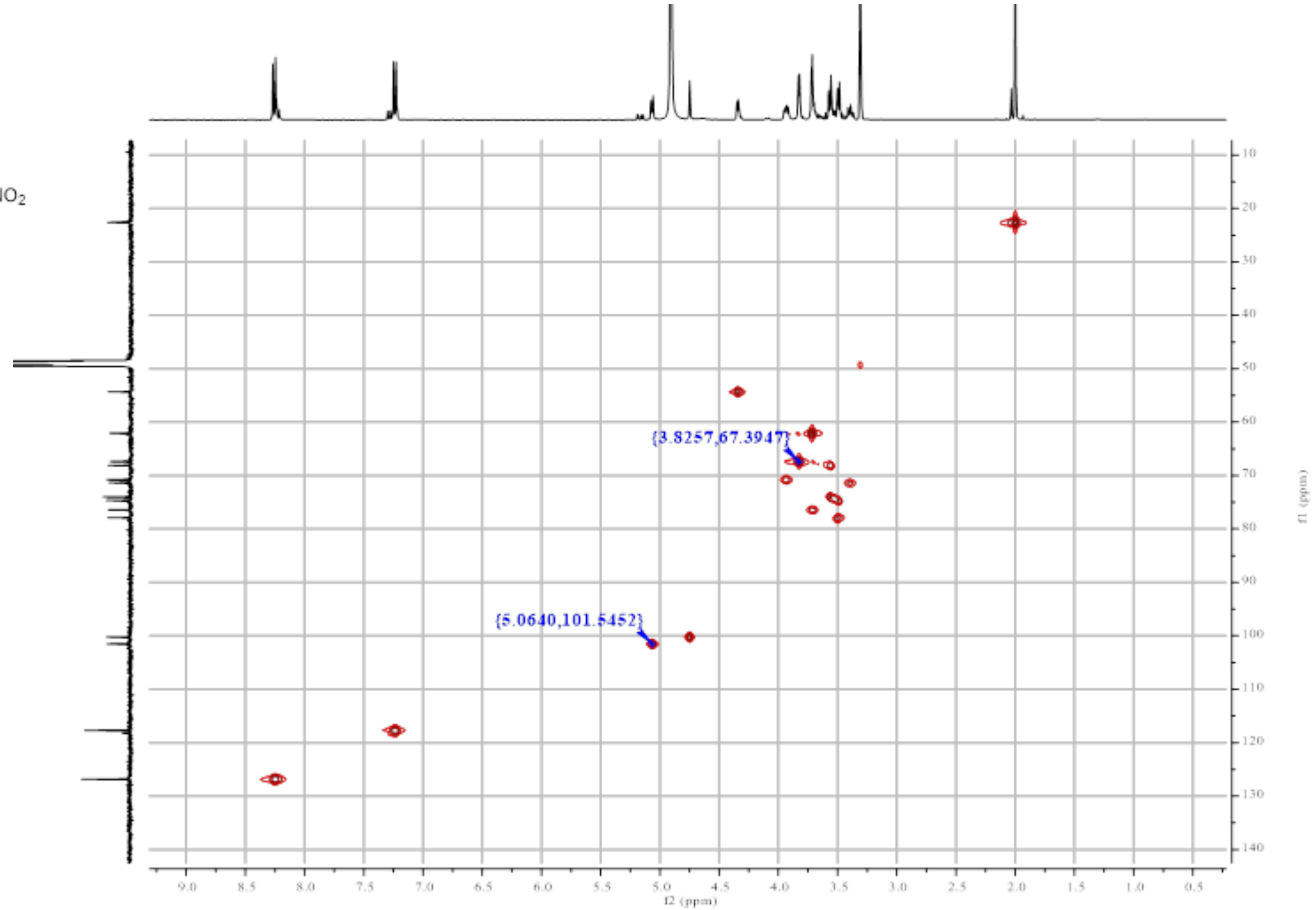

Compound **4p**, HMBC (150 MHz, CD_3OD)

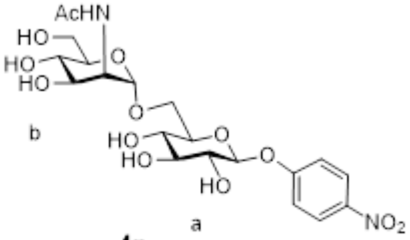

4p

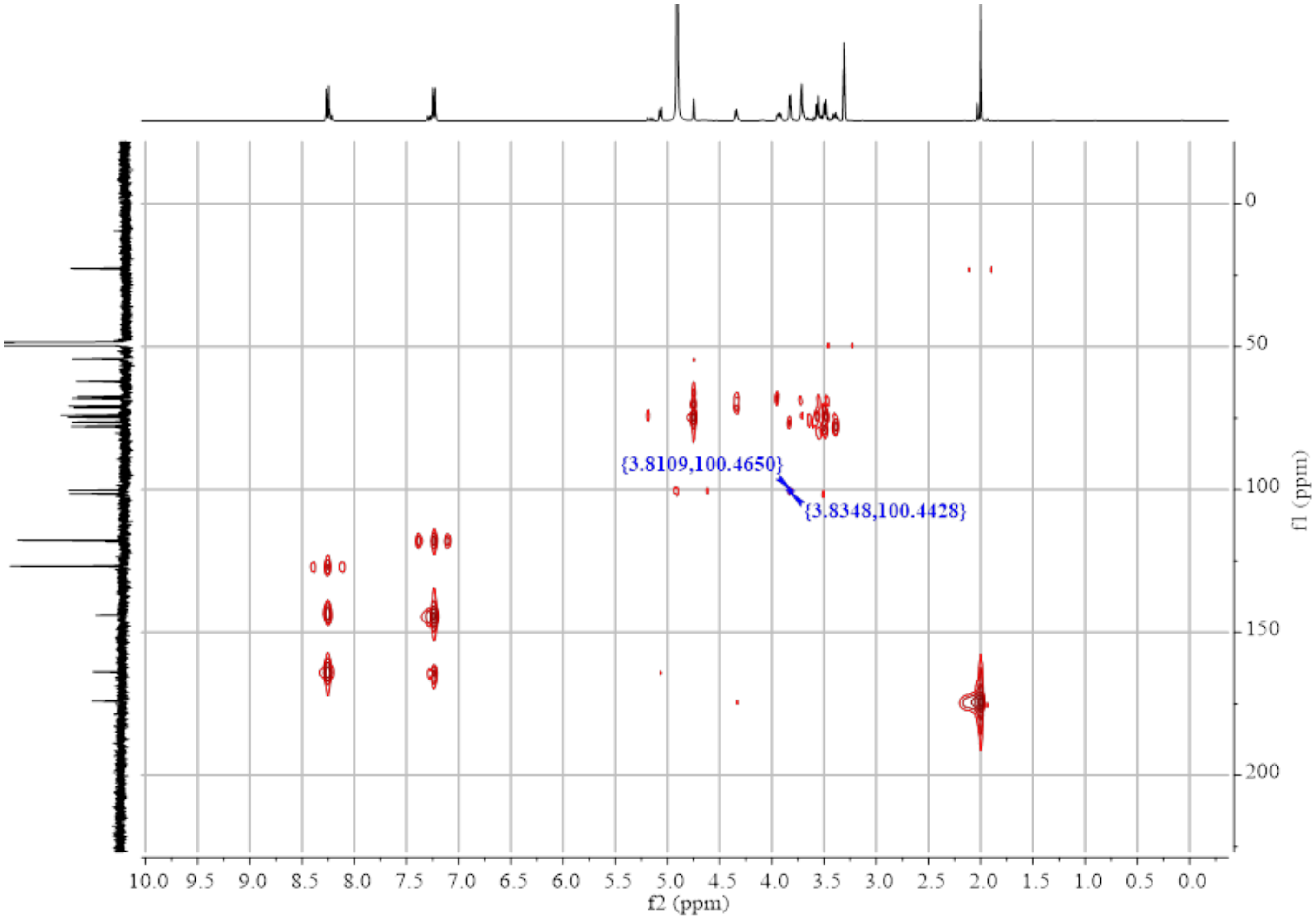

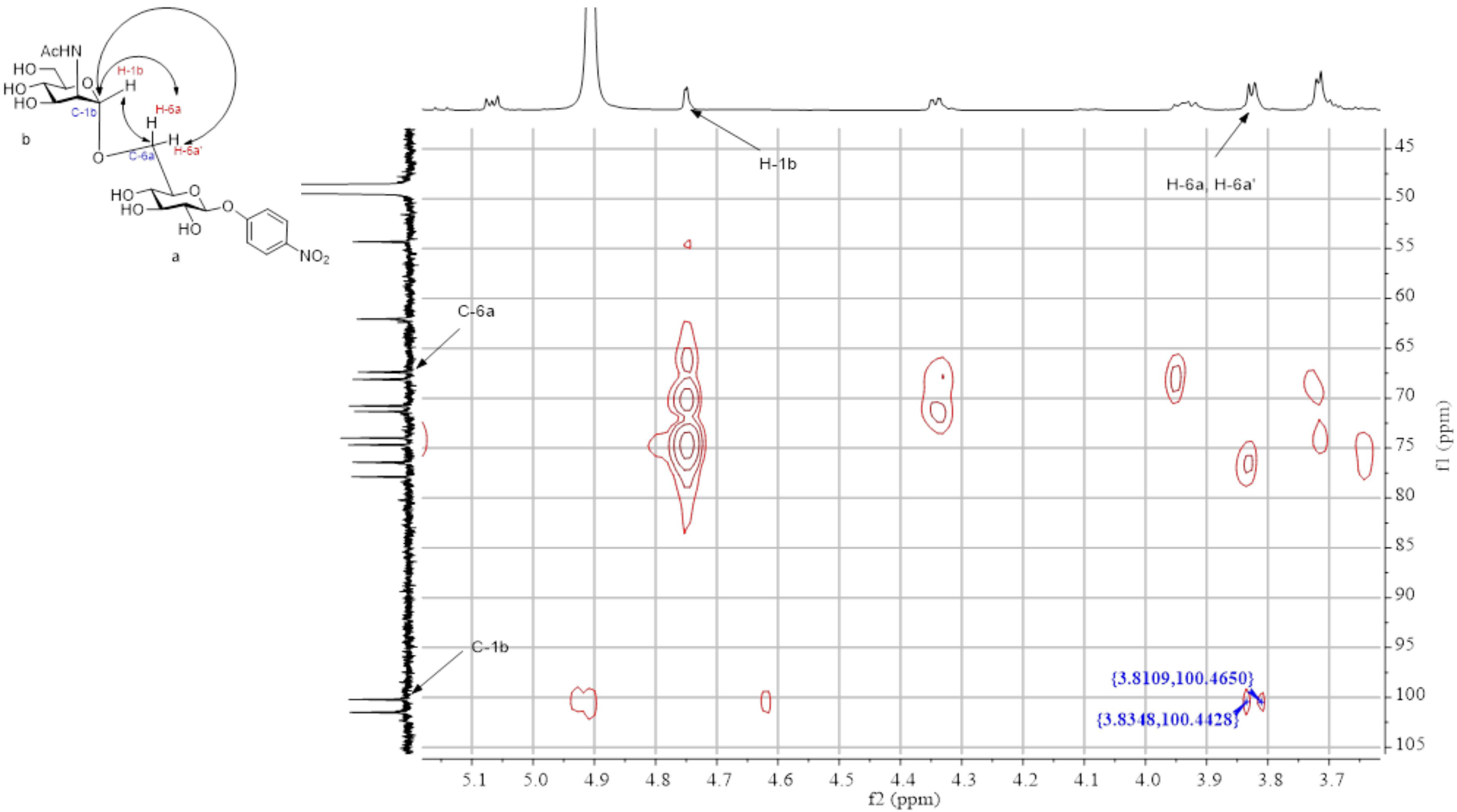
AcHN
HO
HO
HO
O
H-1b
H
C-1b
H-6a
H
H
H-6a'
C-6a
O
b
HO
HO
O
O
HO
a
NO2
H-1b
H-6a, H-6a'
C-6a
C-1b
{3.8109,100.4650}
{3.8348,100.4428}
f2 (ppm)
f1 (ppm)

***p*-Nitrophenyl 2-acetamido-2-deoxy-β-D-glucopyranosyl-(1→3)-β-D-galctopyranoside**, ^{1}H NMR (600 MHz, D_2O)

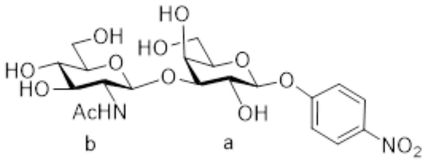

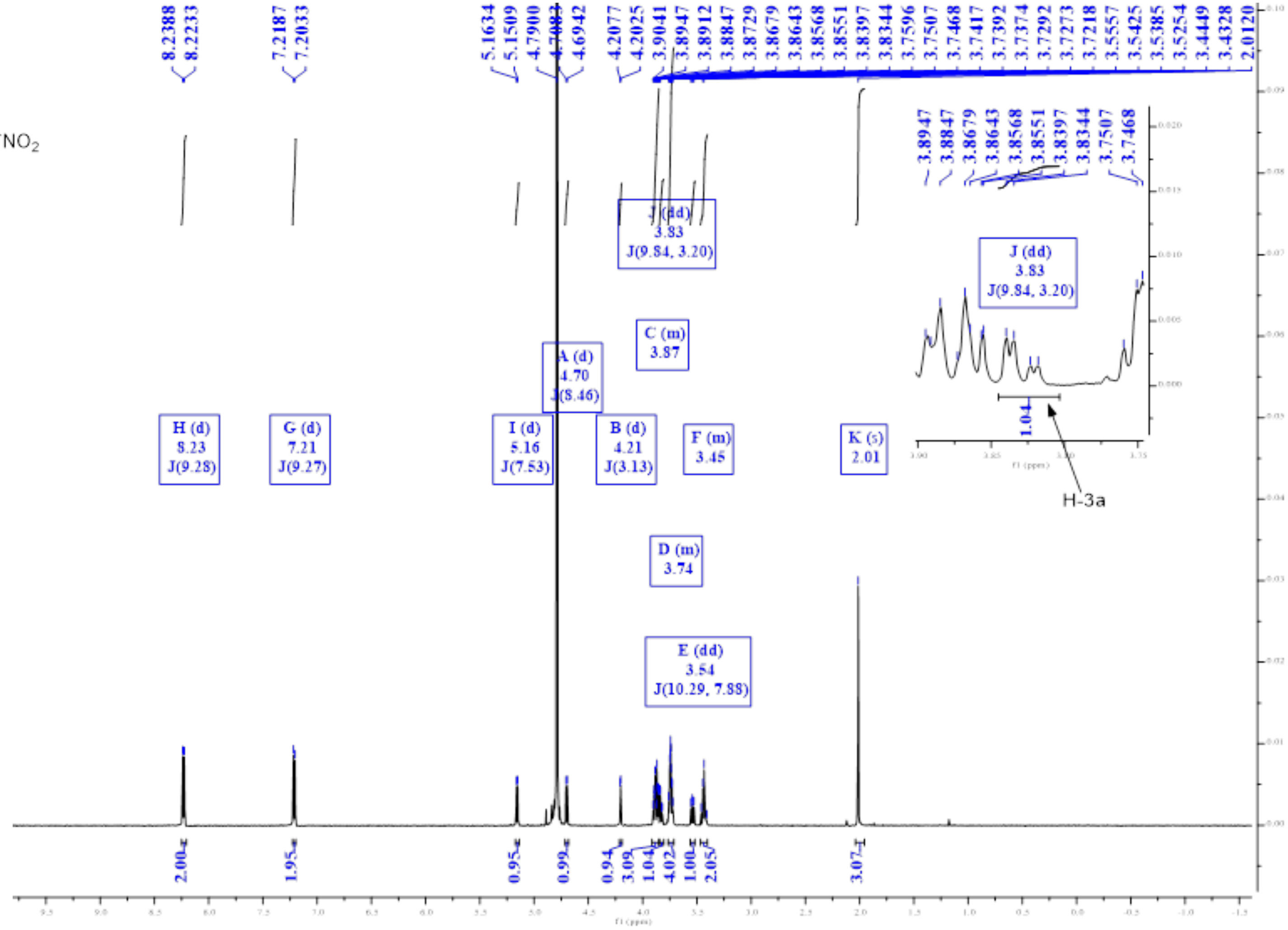

***p*-Nitrophenyl 2-acetamido-2-deoxy-β-D-glucopyranosyl-(1→3)-β-D-galctopyranoside**, ^{13}C NMR (150 MHz, D_2O)

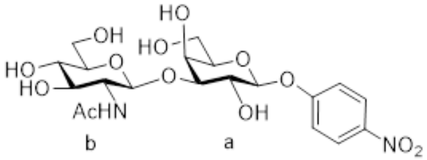

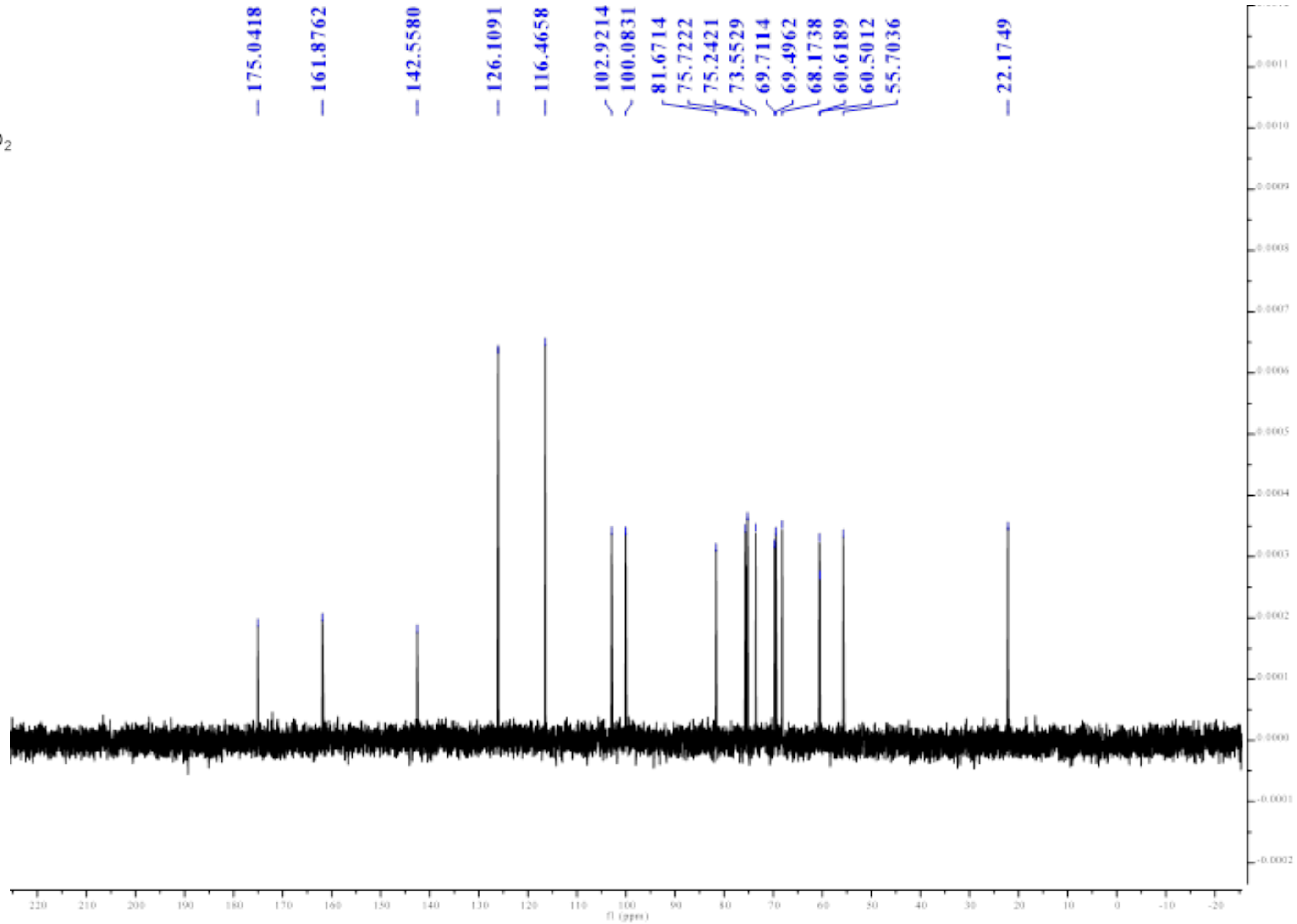

***p*-Nitrophenyl 2-acetamido-2-deoxy-β-D-glucopyranosyl-(1→3)-β-D-galctopyranoside**, DEPT 135 (150 MHz, D_2O)

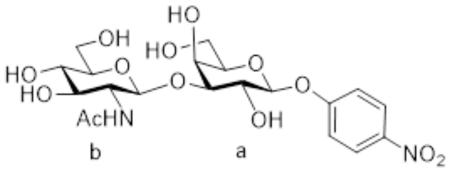

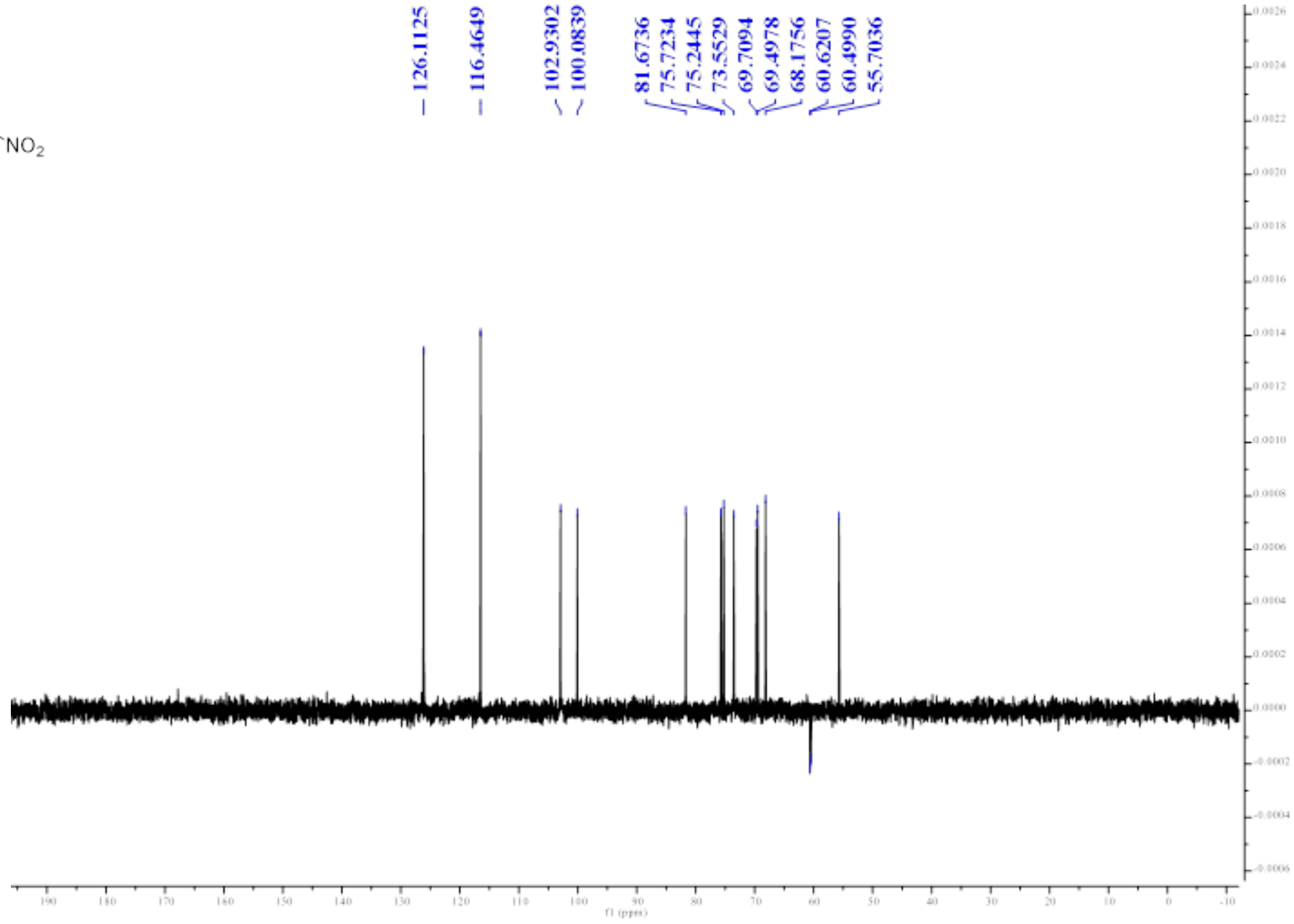

***p*-Nitrophenyl 2-acetamido-2-deoxy-β-D-glucopyranosyl-(1→3)-β-D-galctopyranoside**, HSQC (600 MHz, D_2O)

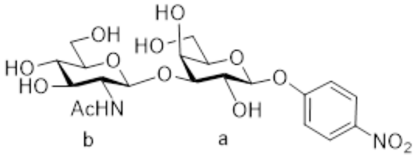

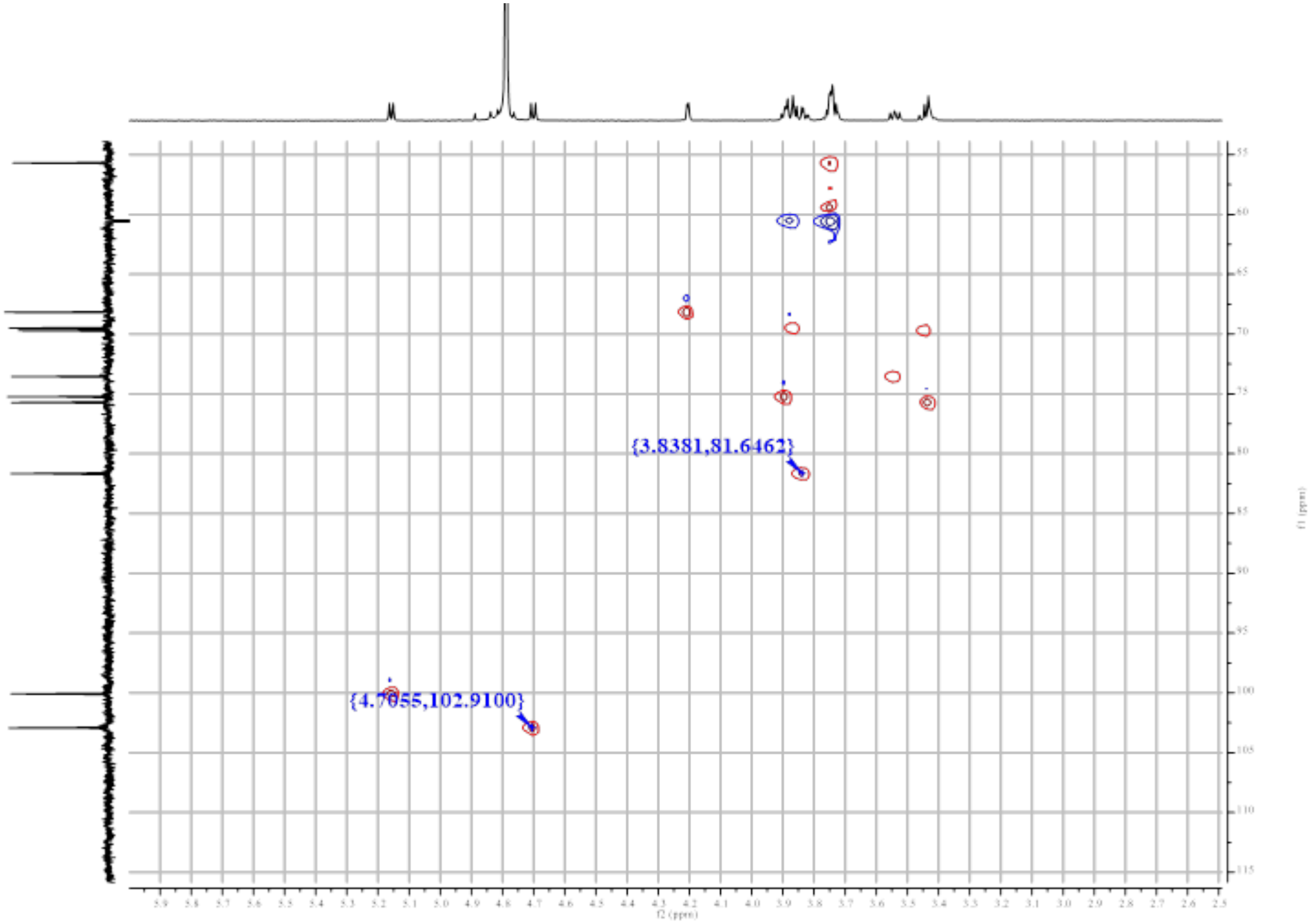

***p*-Nitrophenyl 2-acetamido-2-deoxy-β-D-glucopyranosyl-(1→3)-β-D-galctopyranoside**, HMBC (600 MHz, D_2O)

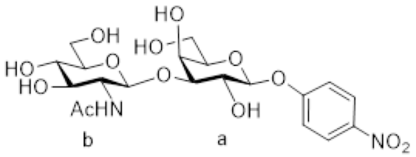

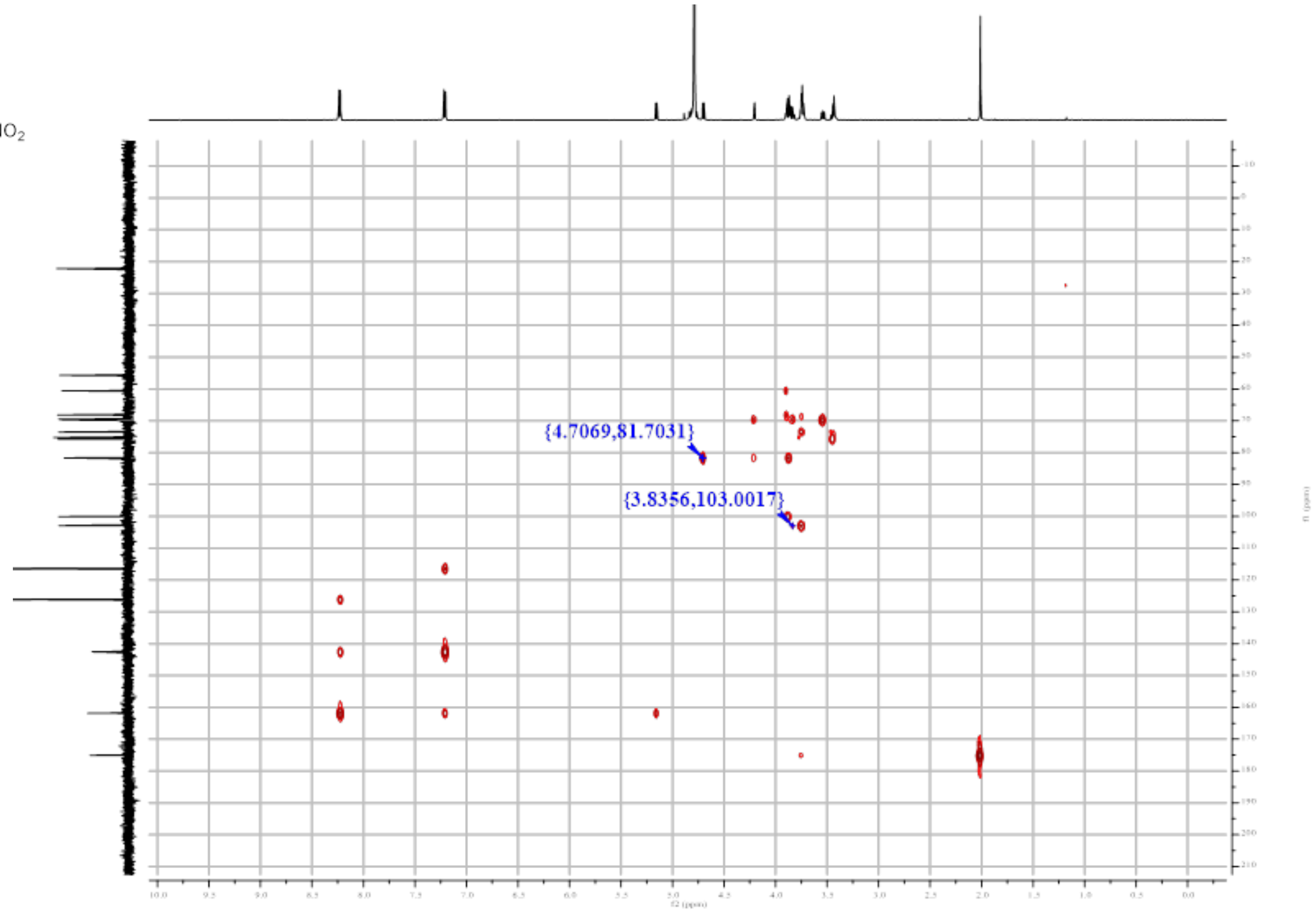

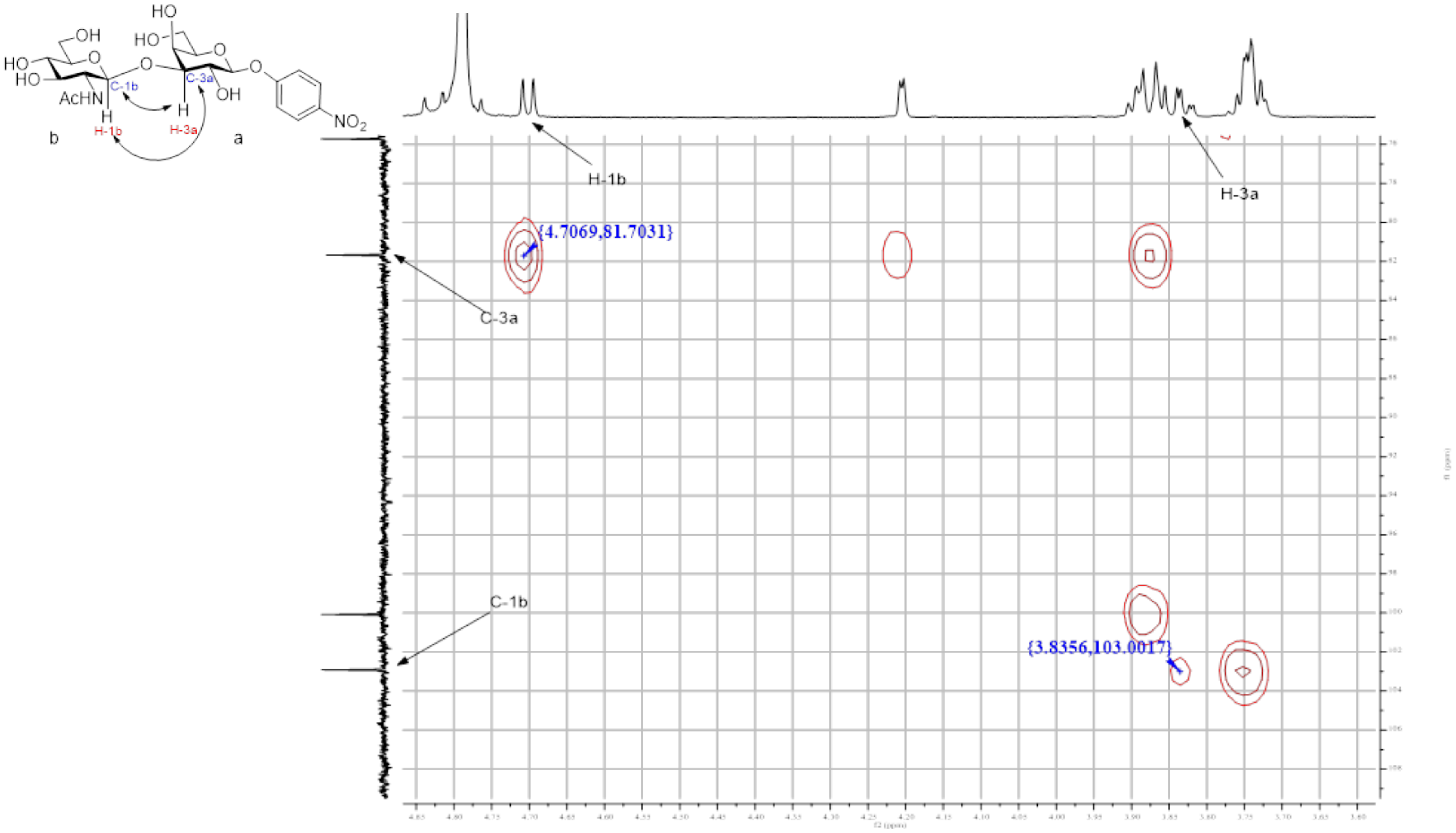
HO
OH
HO
HO
AcHN
O
C-1b
H
H-1b
b
HO
O
C-3a
H
H-3a
OH
a
O
NO2
H-1b
H-3a
C-3a
C-1b
{4.7069,81.7031}
{3.8356,103.0017}

***p*-Nitrophenyl 2-acetamido-2-deoxy-β-D-galactopyranosyl-(1→3)-β-D-galactopyranoside**, ^{1}H NMR (600 MHz, D_2O)

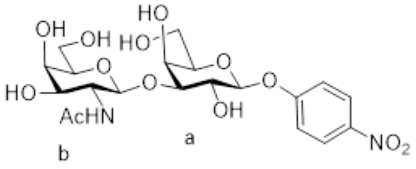

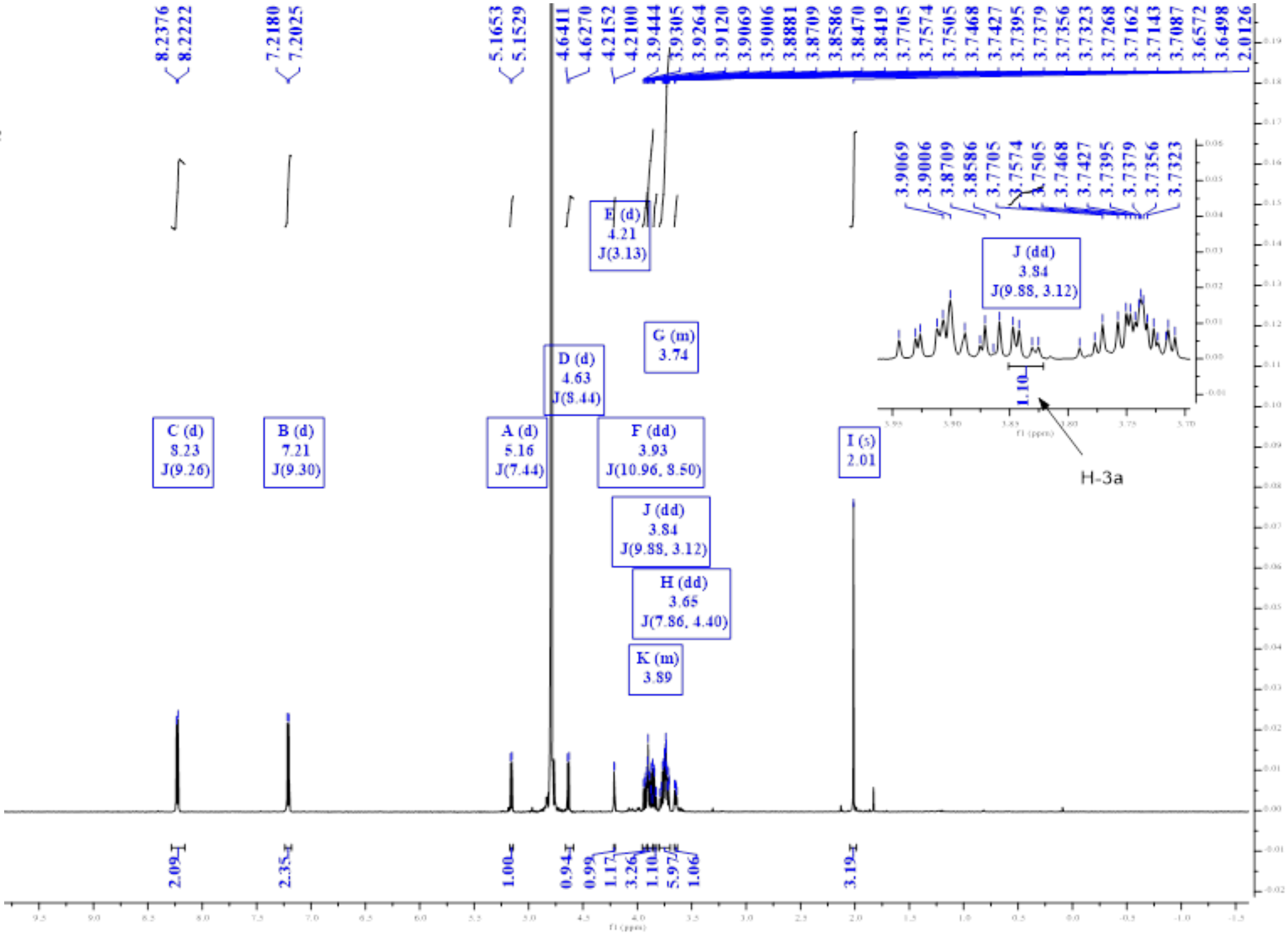

***p*-Nitrophenyl 2-acetamido-2-deoxy-β-D-galactopyranosyl-(1→3)-β-D-galactopyranoside**, ^{13}C NMR (150 MHz, D_2O)

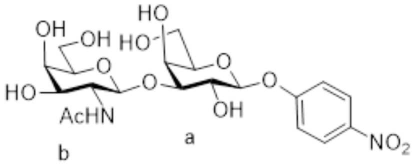

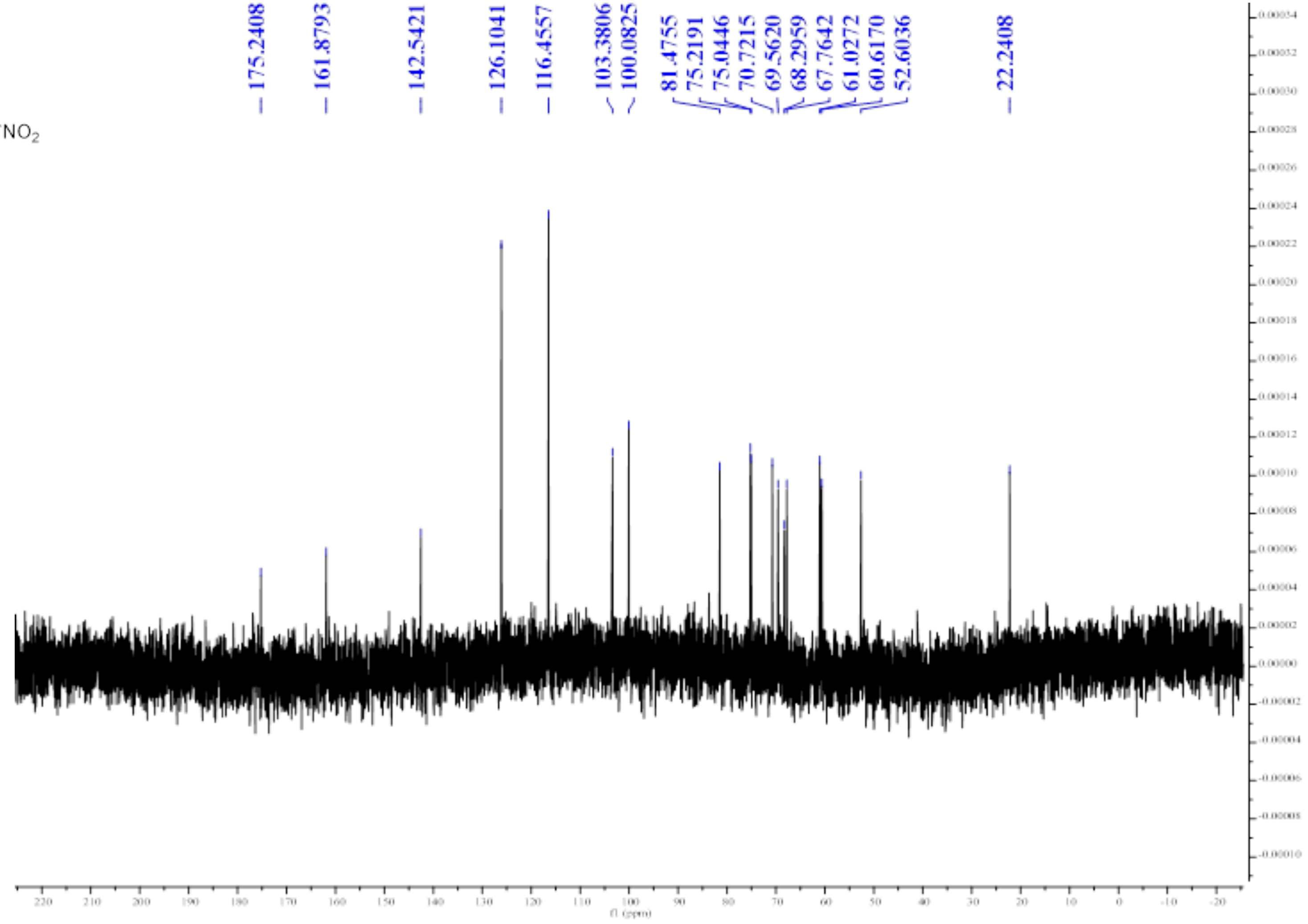

p-Nitrophenyl 2-acetamido-2-deoxy-β-D-galactopyranosyl-(1→3)-β-D-galactopyranoside, HSQC (600 MHz, D_2O)

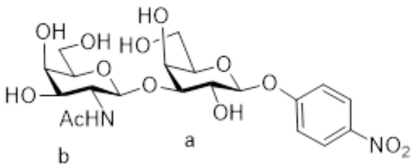

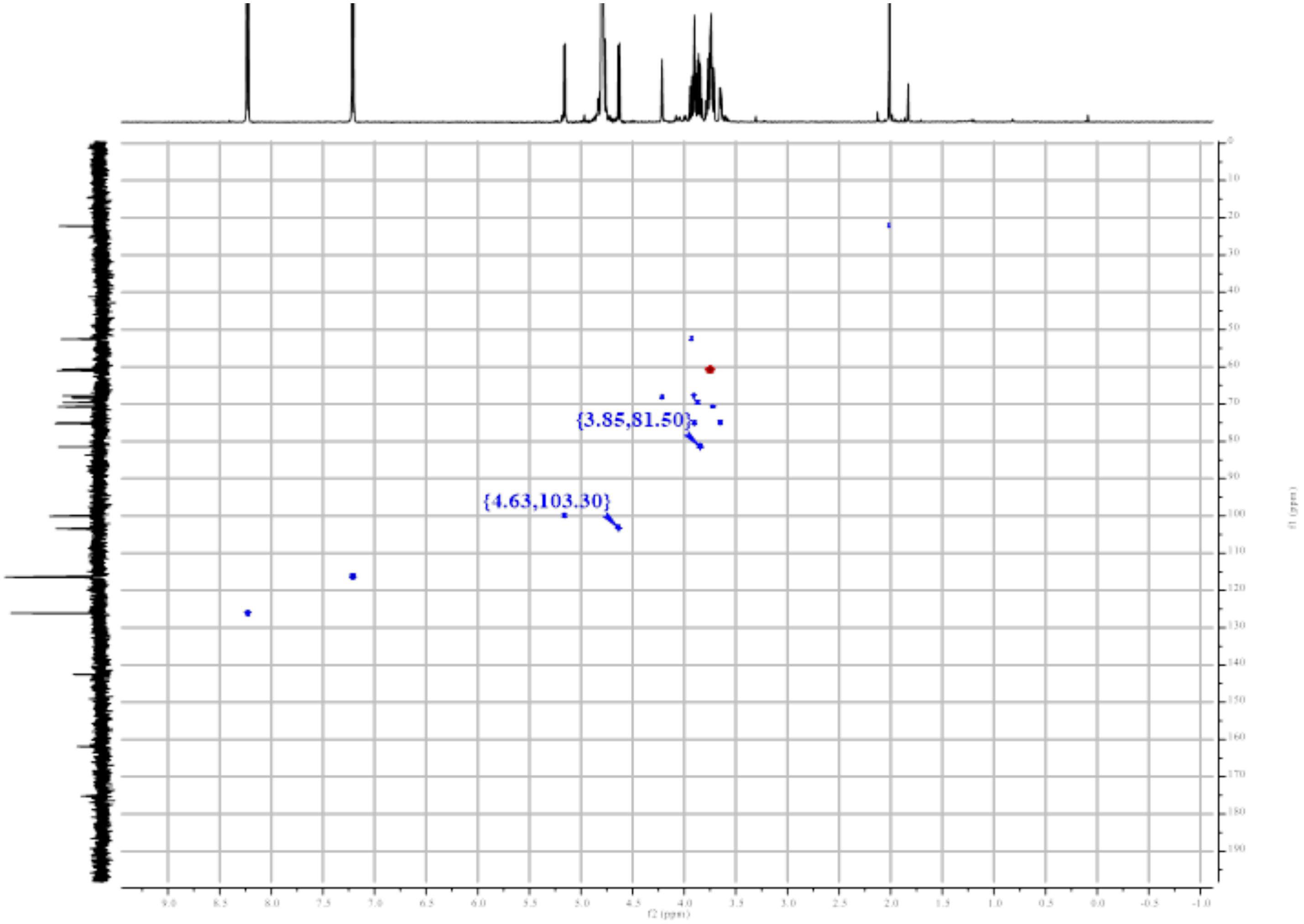

***p*-Nitrophenyl 2-acetamido-2-deoxy-β-D-galactopyranosyl-(1→3)-β-D-galactopyranoside**, HMBC (600 MHz, D_2O)

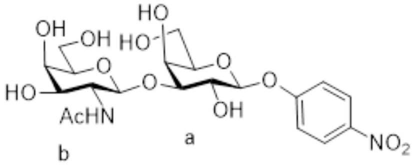

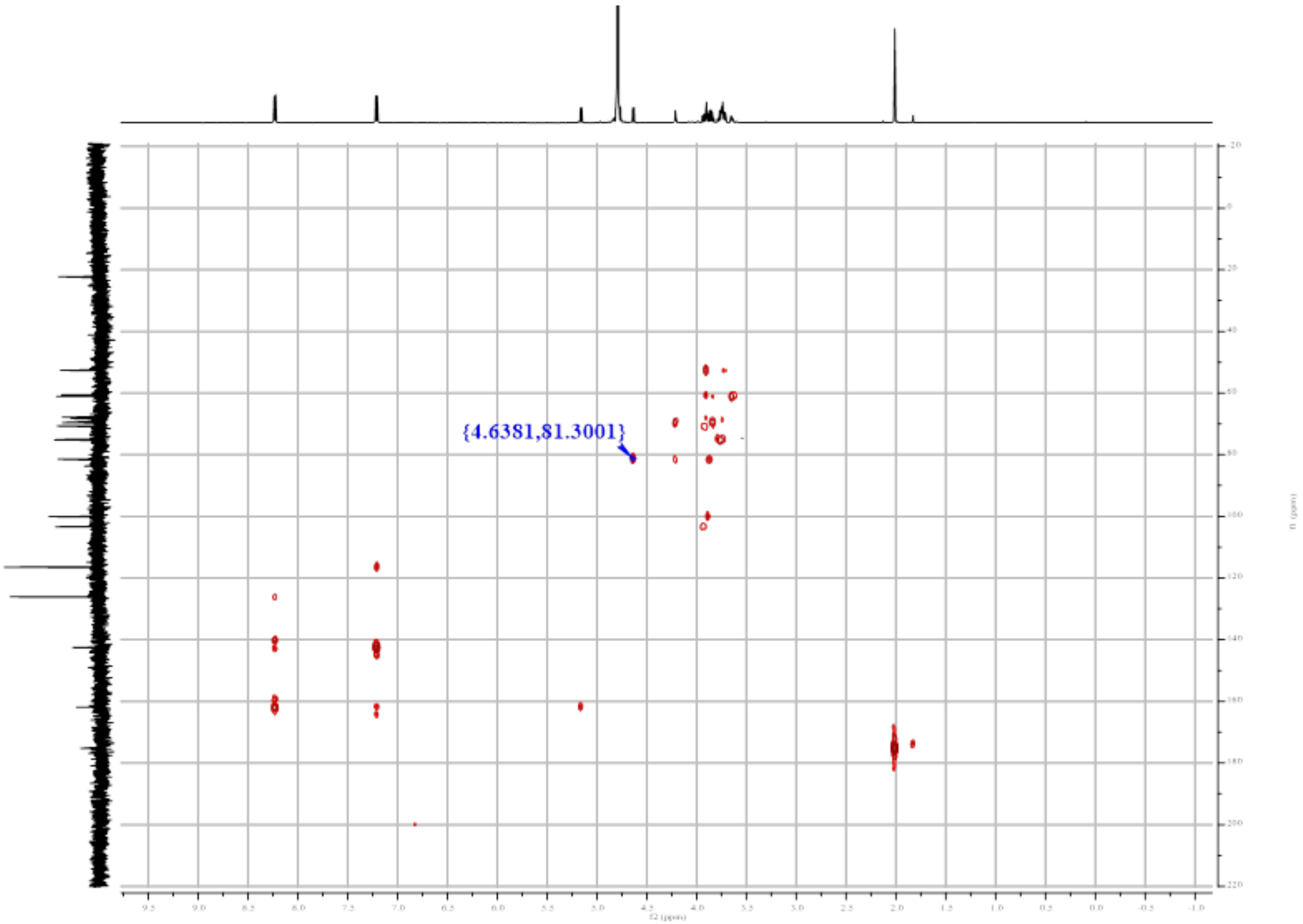

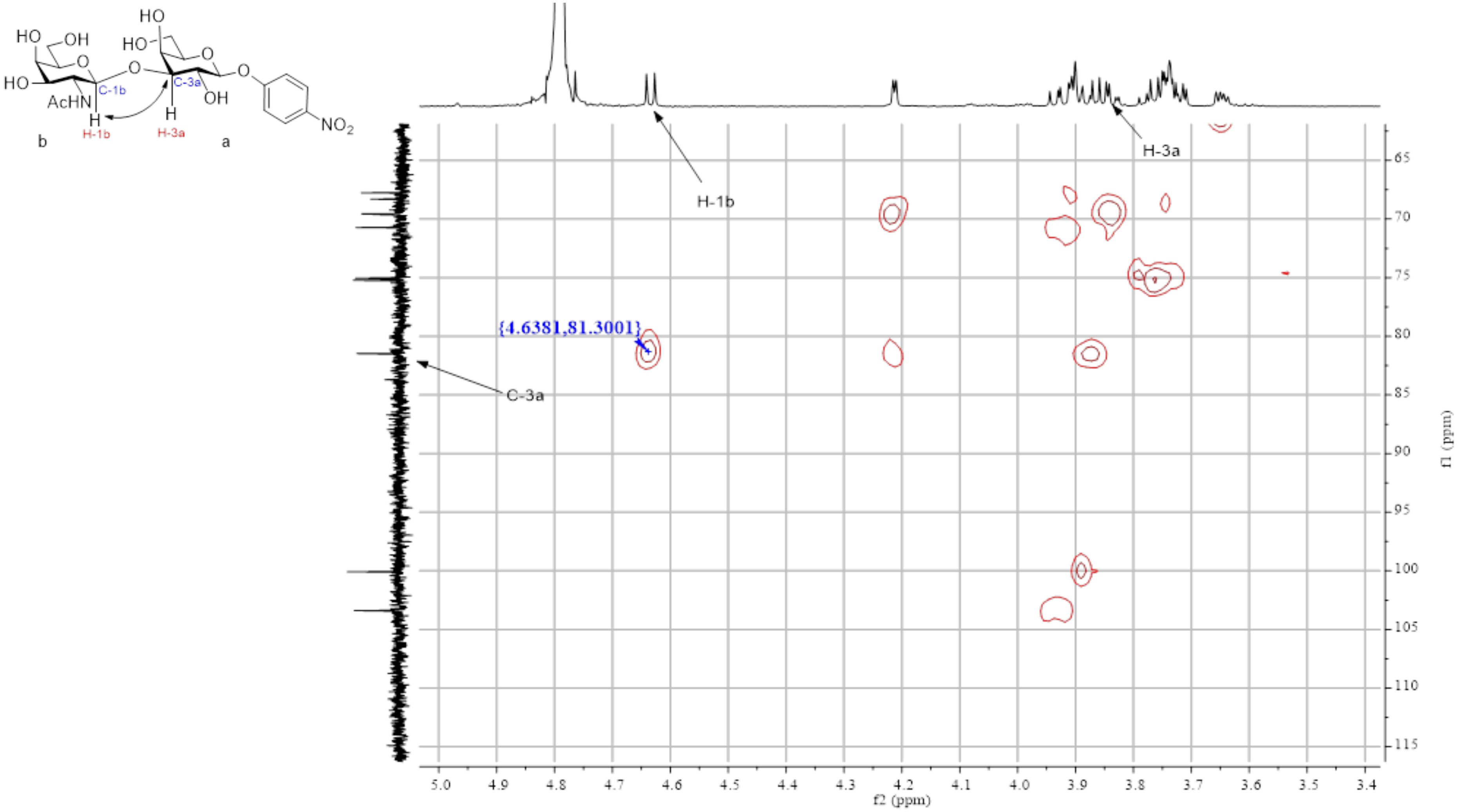
HO
OH
HO
HO
O
O
O
O
HO
AcHN
C-1b
C-3a
OH
H
H
H-1b
H-3a
b
a
NO2
H-1b
H-3a
{4.6381,81.3001}
C-3a
f2 (ppm)
f1 (ppm)

***p*-Nitrophenyl 2-acetamido-2-deoxy-β-D-glucopyranosyl-(1→6)[-α-D-glucopyranosyl-(1→4)]-β-D-glucopyranoside T1**, ^{1}H NMR (400 MHz, D_2O)

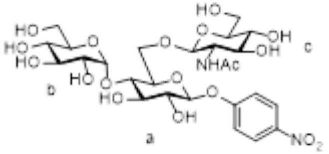

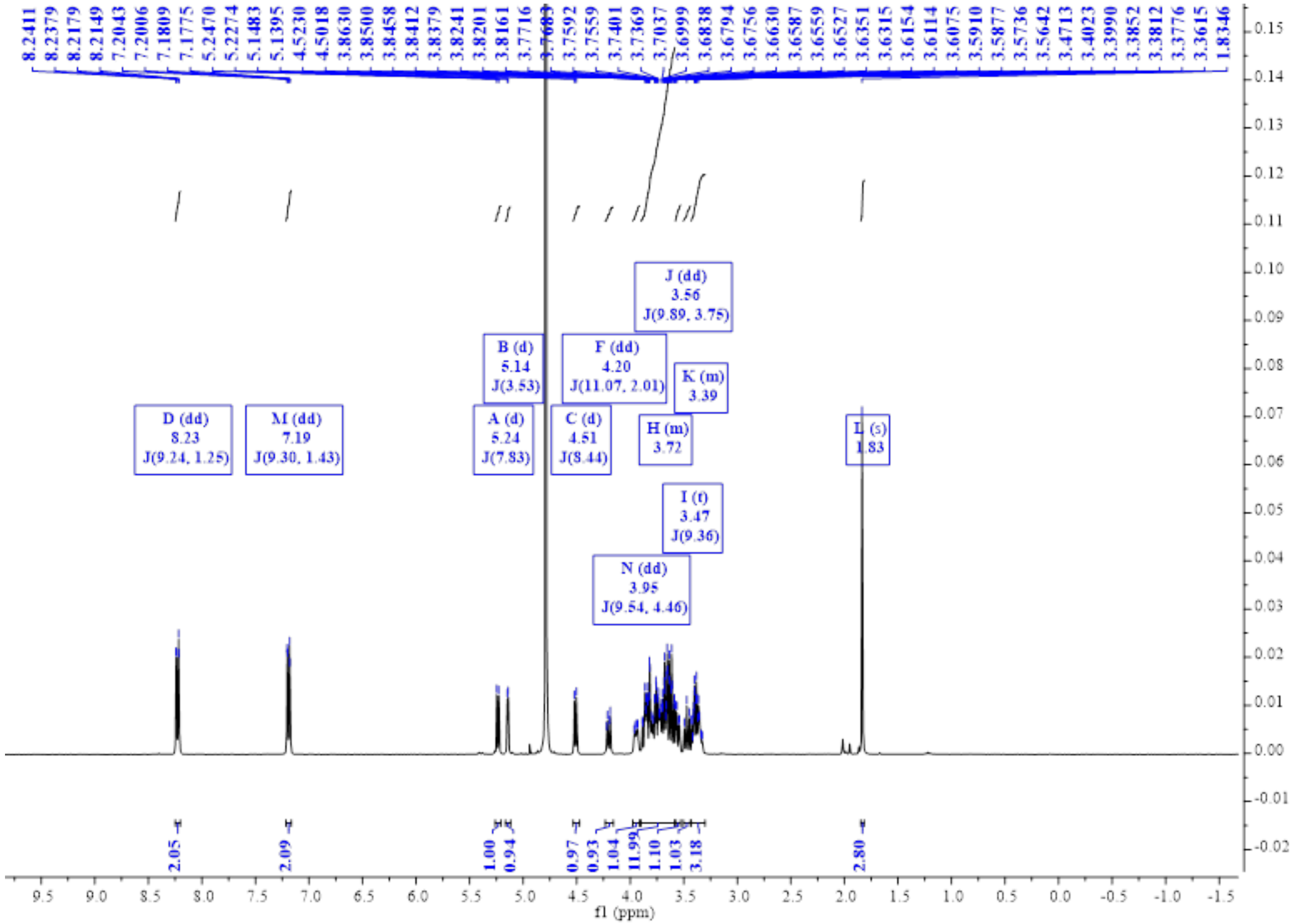

***p*-Nitrophenyl 2-acetamido-2-deoxy-β-D-glucopyranosyl-(1→6)[-α-D-glucopyranosyl-(1→4)]-β-D-glucopyranoside T1**, ^{13}C NMR (150 MHz, D_2O)

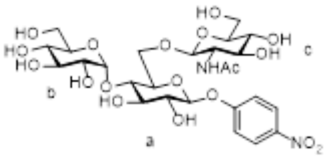

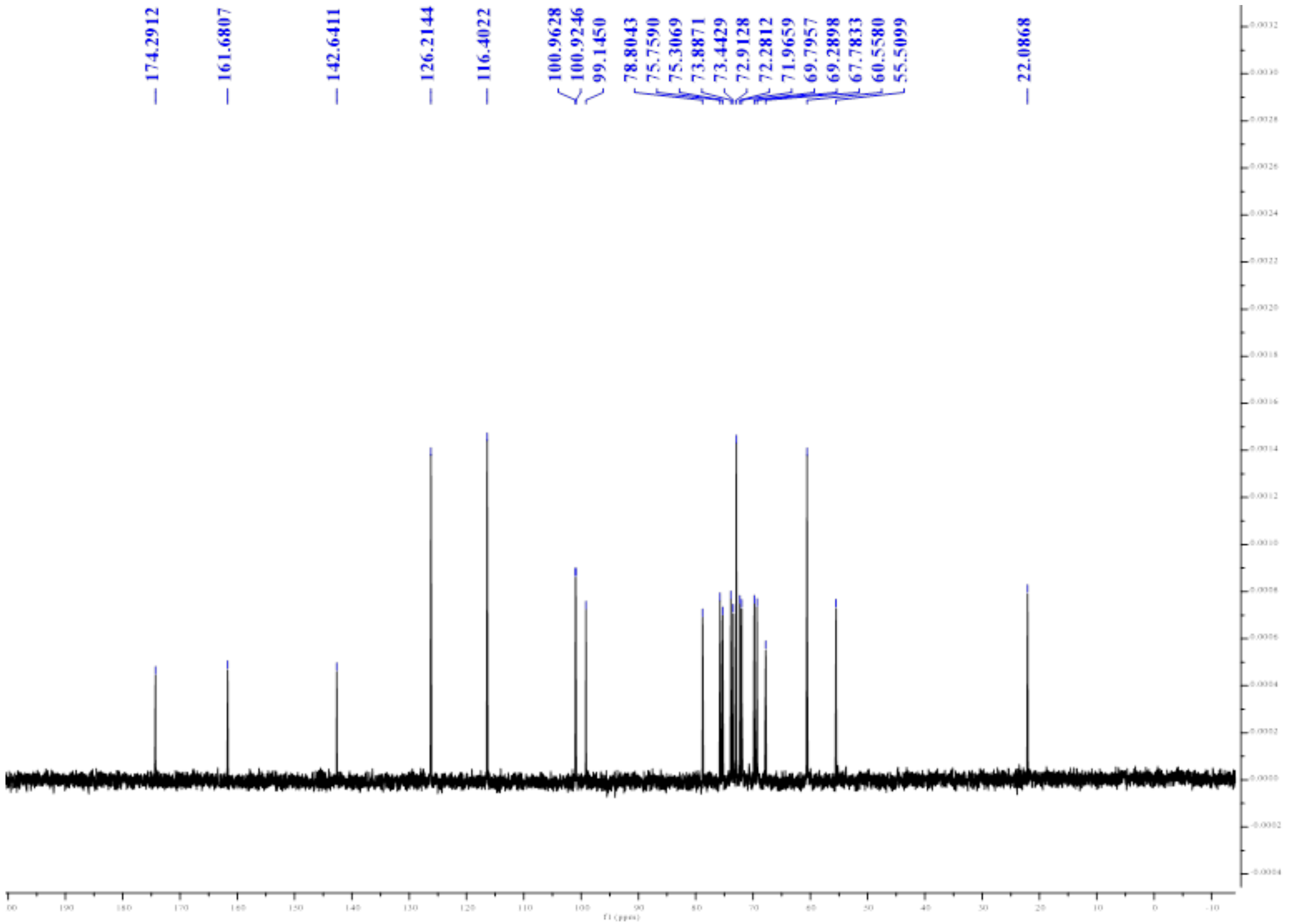

***p*-Nitrophenyl 2-acetamido-2-deoxy-β-D-glucopyranosyl-(1→6)[-α-D-glucopyranosyl-(1→4)]-β-D-glucopyranoside T1**, DEPT 135 (150 MHz, D_2O)

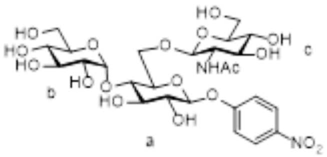

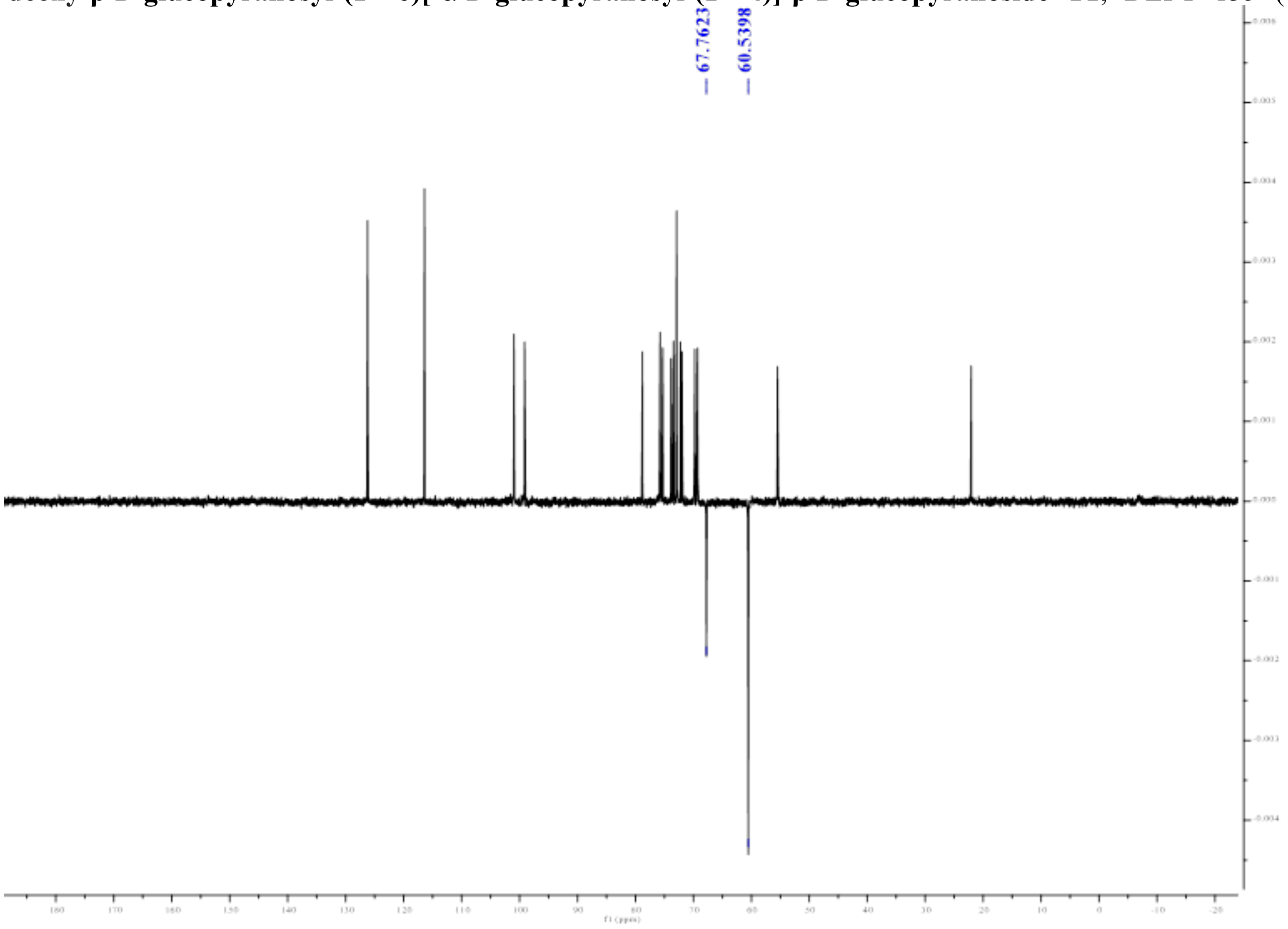

***p*-Nitrophenyl 2-acetamido-2-deoxy-β-D-glucopyranosyl-(1→6)[-α-D-glucopyranosyl-(1→4)]-β-D-glucopyranoside T1**, HMQC (600 MHz, D_2O)

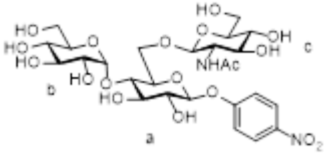

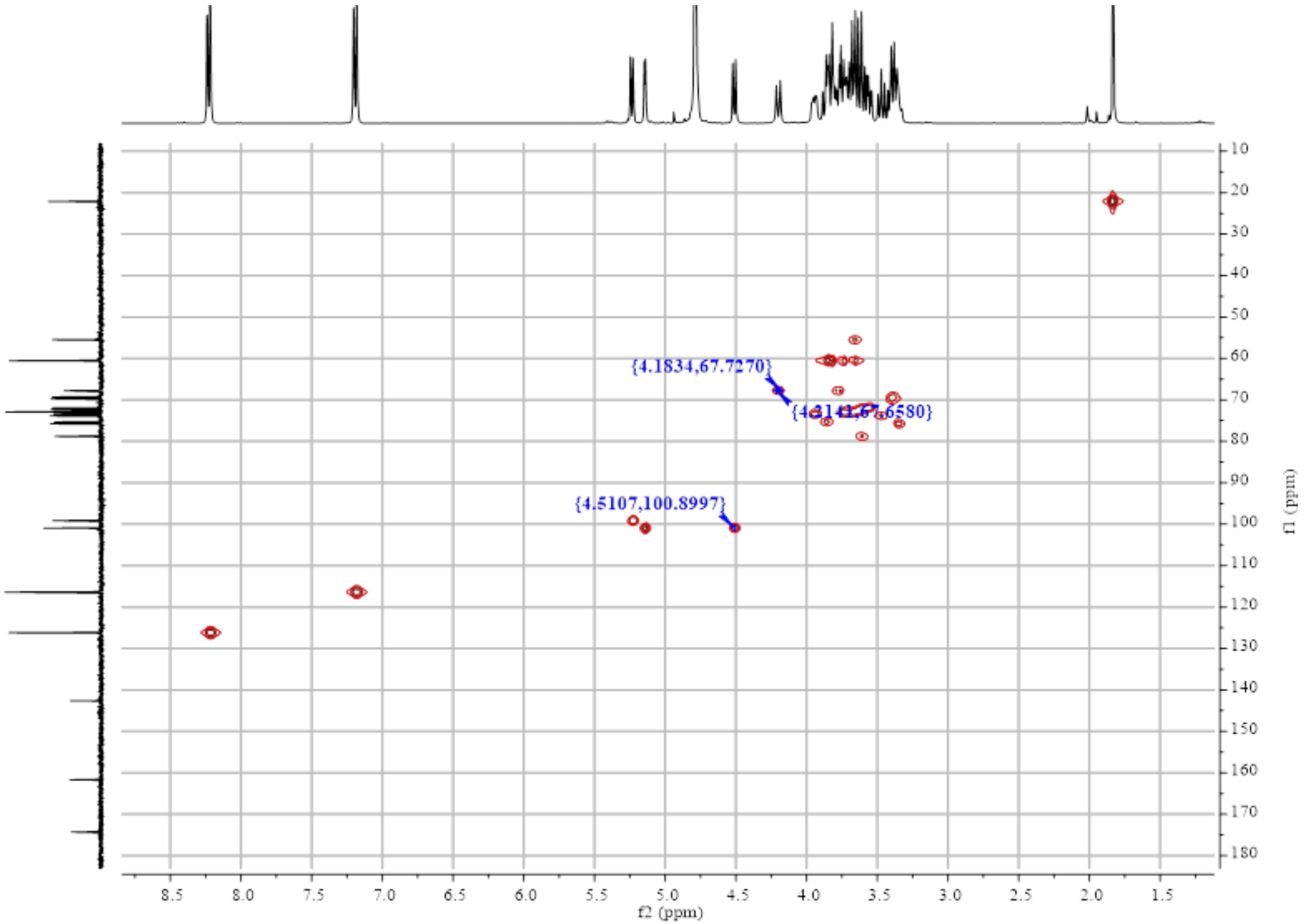

***p*-Nitrophenyl 2-acetamido-2-deoxy-β-D-glucopyranosyl-(1→6)[-α-D-glucopyranosyl-(1→4)]-β-D-glucopyranoside T1**, HMBC (600 MHz, D_2O)

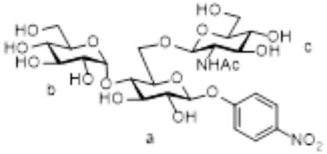

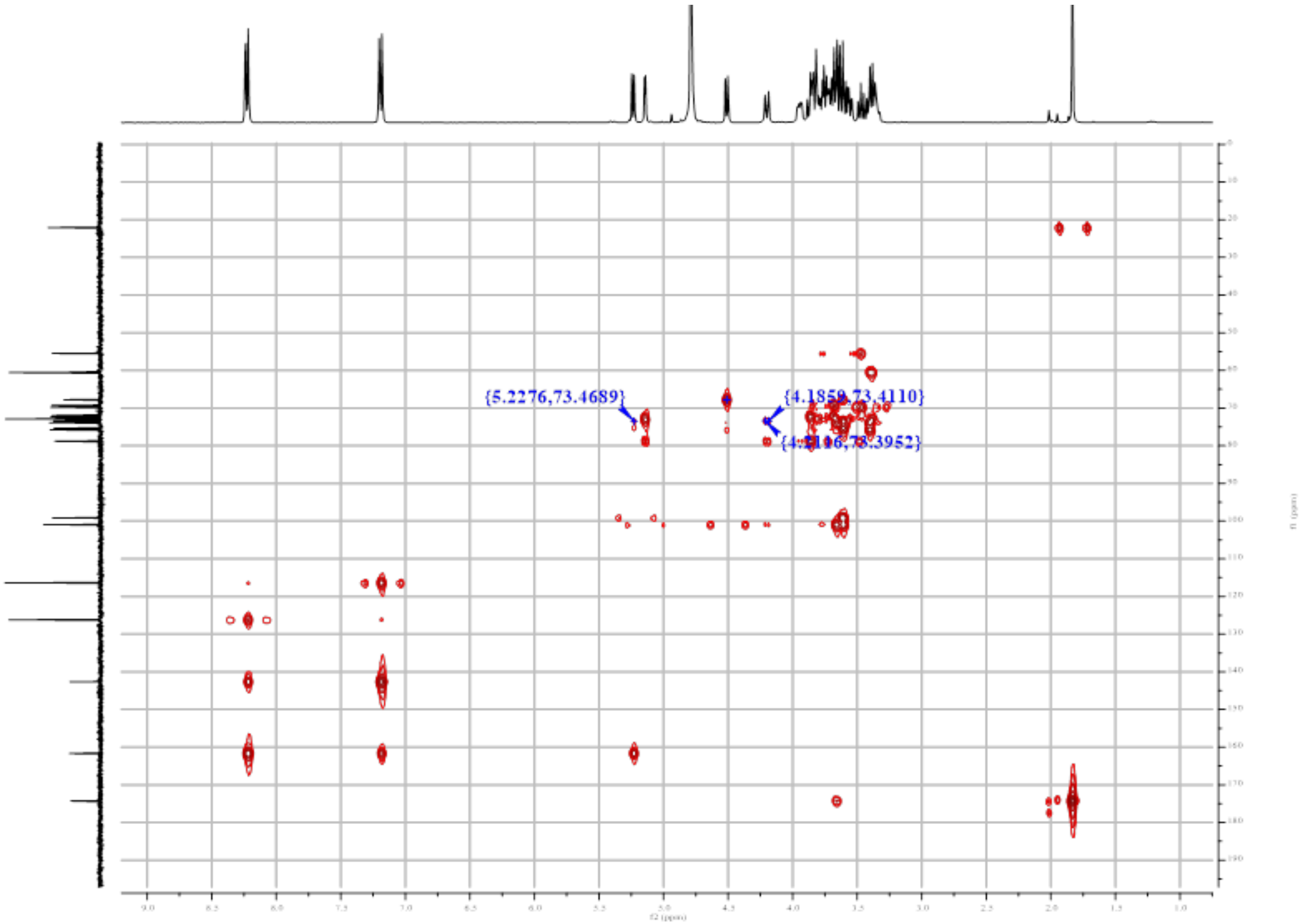

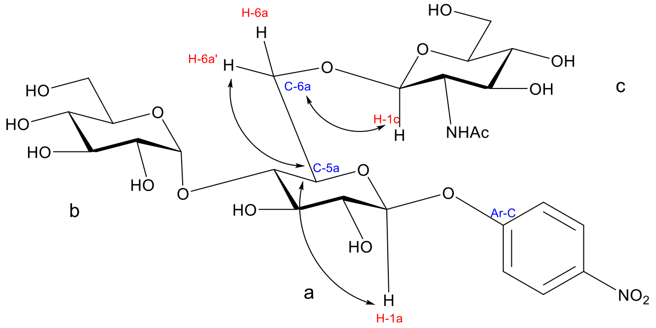
H-6a
H-6a'
C-6a
H-1c
C-5a
Ar-C
H-1a
NHAc
NO2
a
b
c

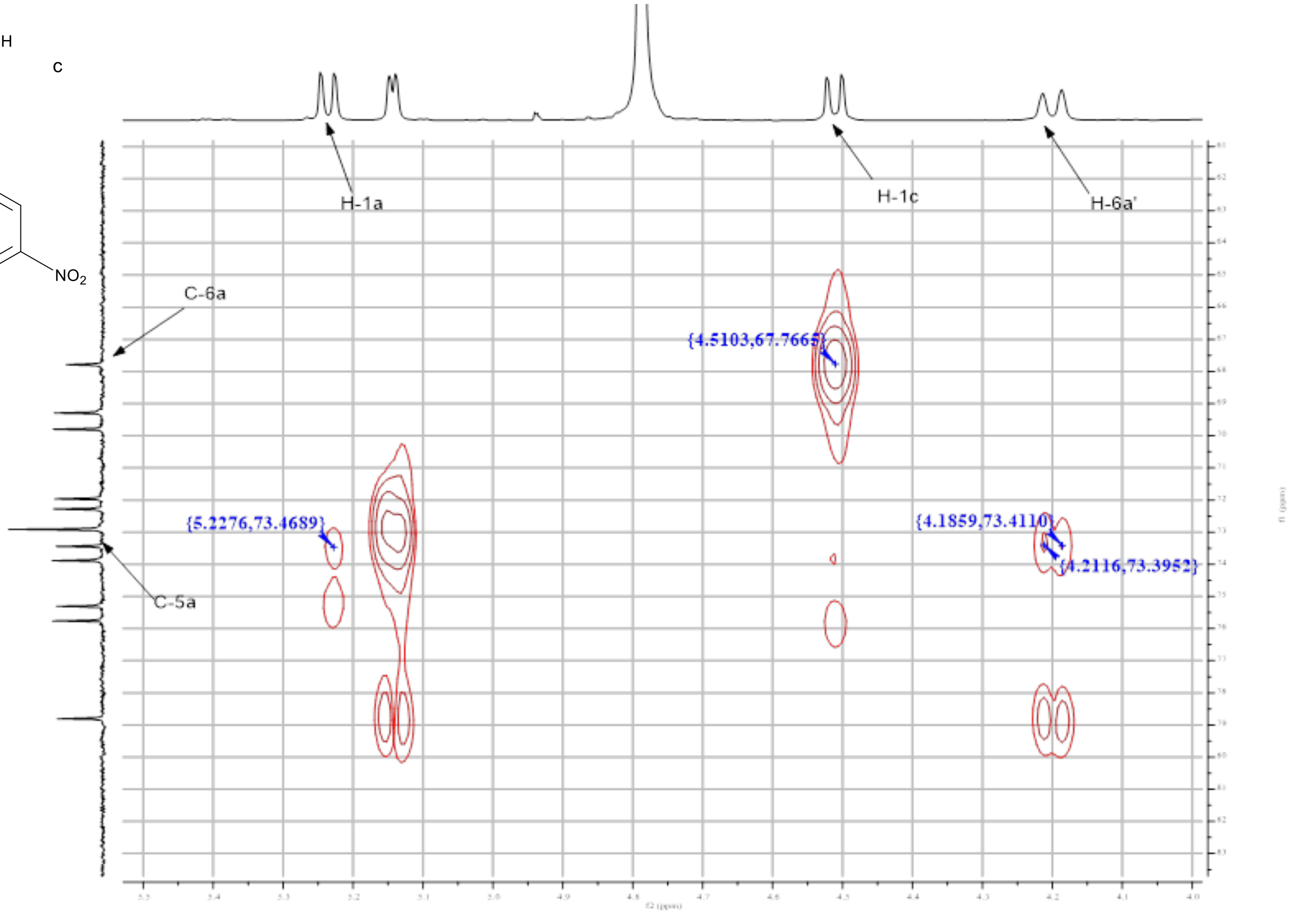
H-1a
H-1c
H-6a'
C-6a
C-5a
{4.5103,67.7665}
{5.2276,73.4689}
{4.1859,73.4110}
{4.2116,73.3952}

***p*-Nitrophenyl 2-acetamido-2-deoxy-β-D-glucopyranosyl-(1→6)-α-D-glucopyranosyl-(1→4)-β-D-glucopyranoside T2**, ^{1}H NMR (400 MHz, D_2O)

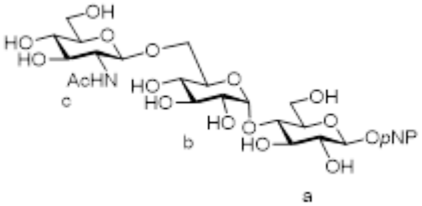

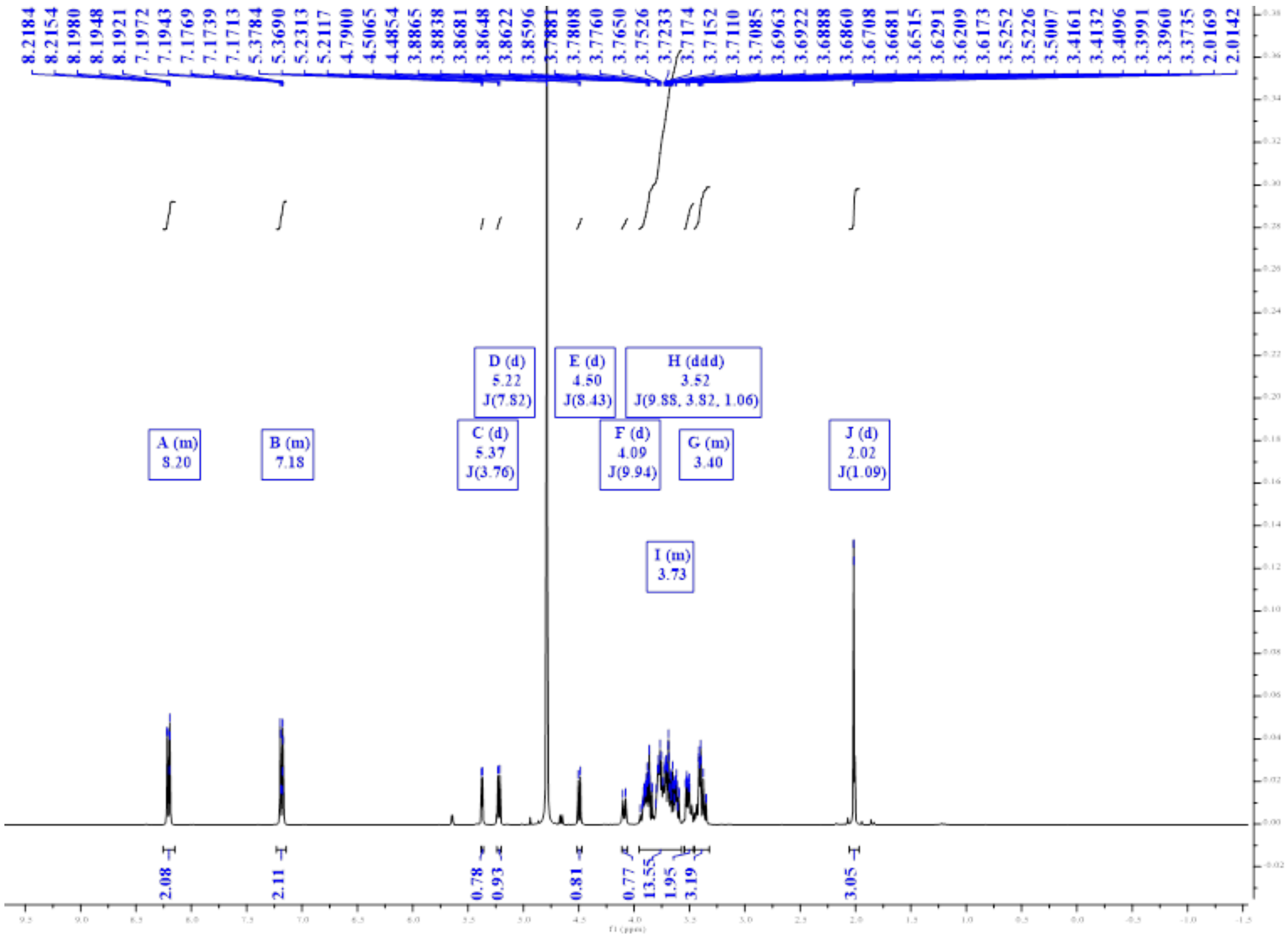

***p*-Nitrophenyl 2-acetamido-2-deoxy-β-D-glucopyranosyl-(1→6)-α-D-glucopyranosyl-(1→4)-β-D-glucopyranoside T2**, ^{13}C NMR (150 MHz, D_2O)

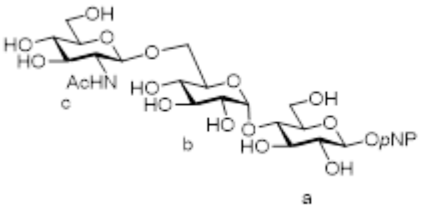

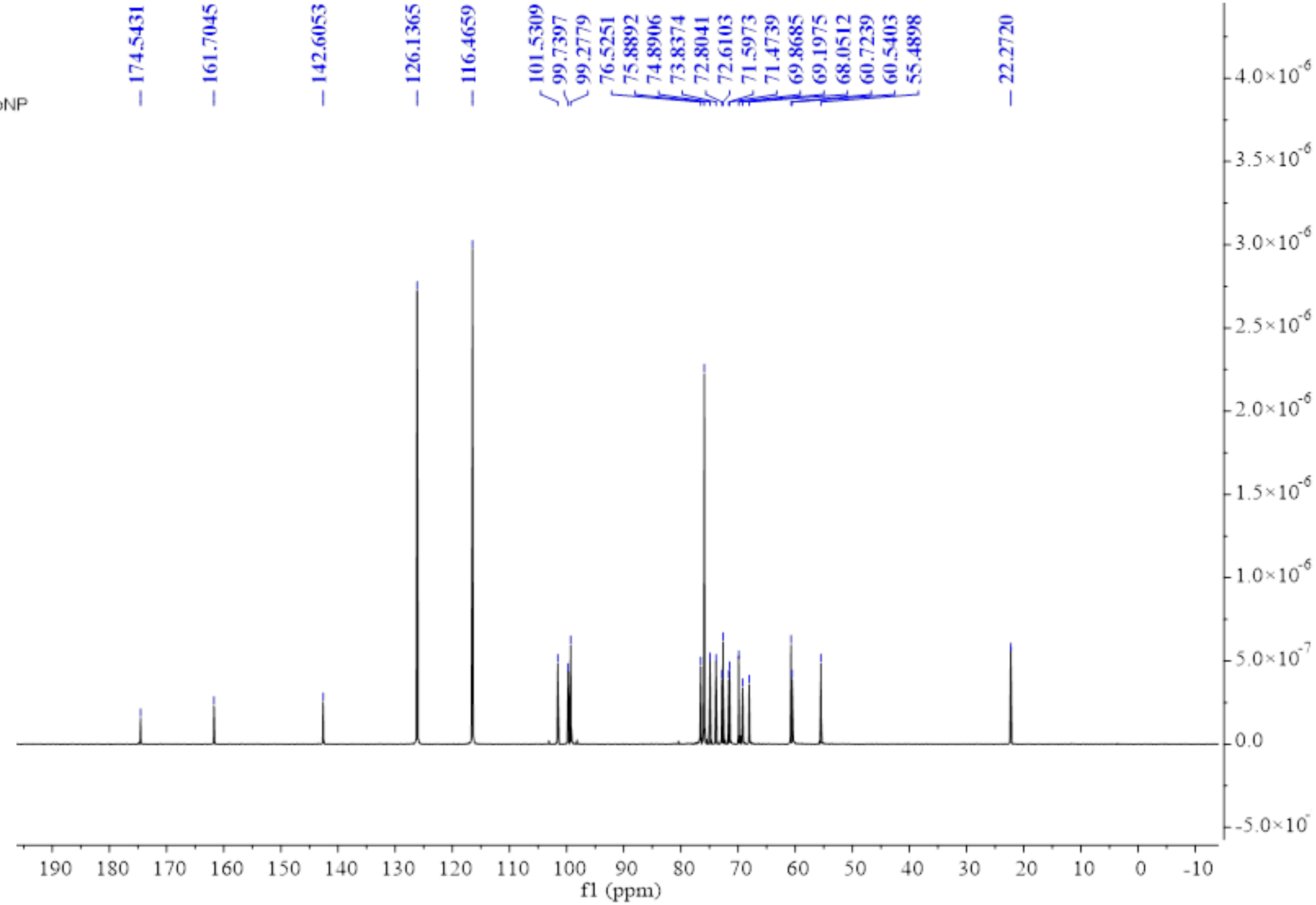

***p*-Nitrophenyl 2-acetamido-2-deoxy-β-D-glucopyranosyl-(1→6)-α-D-glucopyranosyl-(1→4)-β-D-glucopyranoside T2**, DEPT 135 (150 MHz, D_2O)

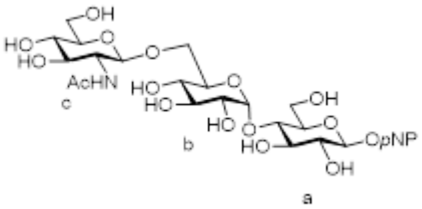

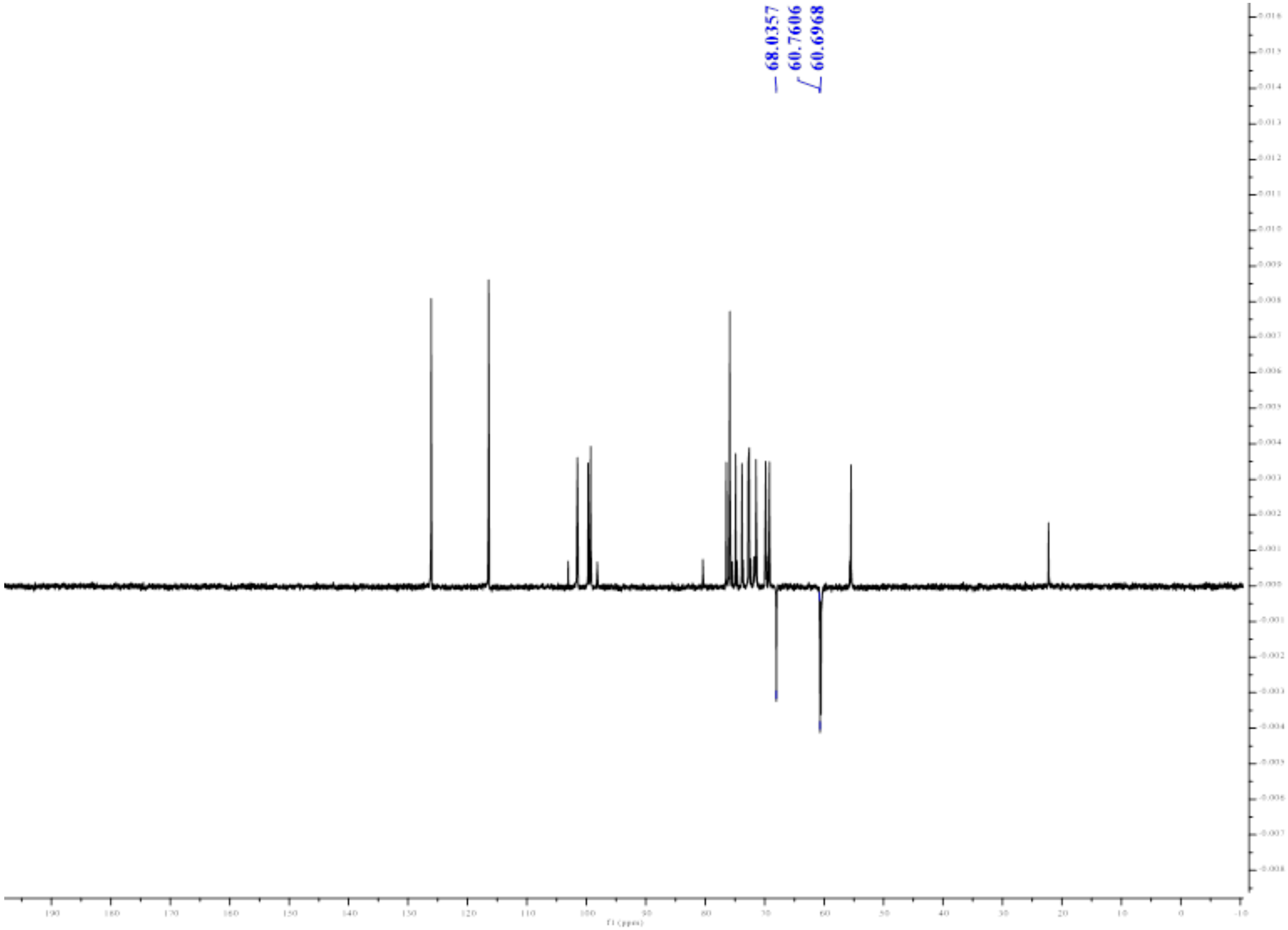

***p*-Nitrophenyl 2-acetamido-2-deoxy-β-D-glucopyranosyl-(1→6)-α-D-glucopyranosyl-(1→4)-β-D-glucopyranoside T2**, HMQC (600 MHz, D_2O)

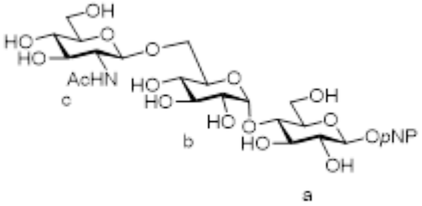

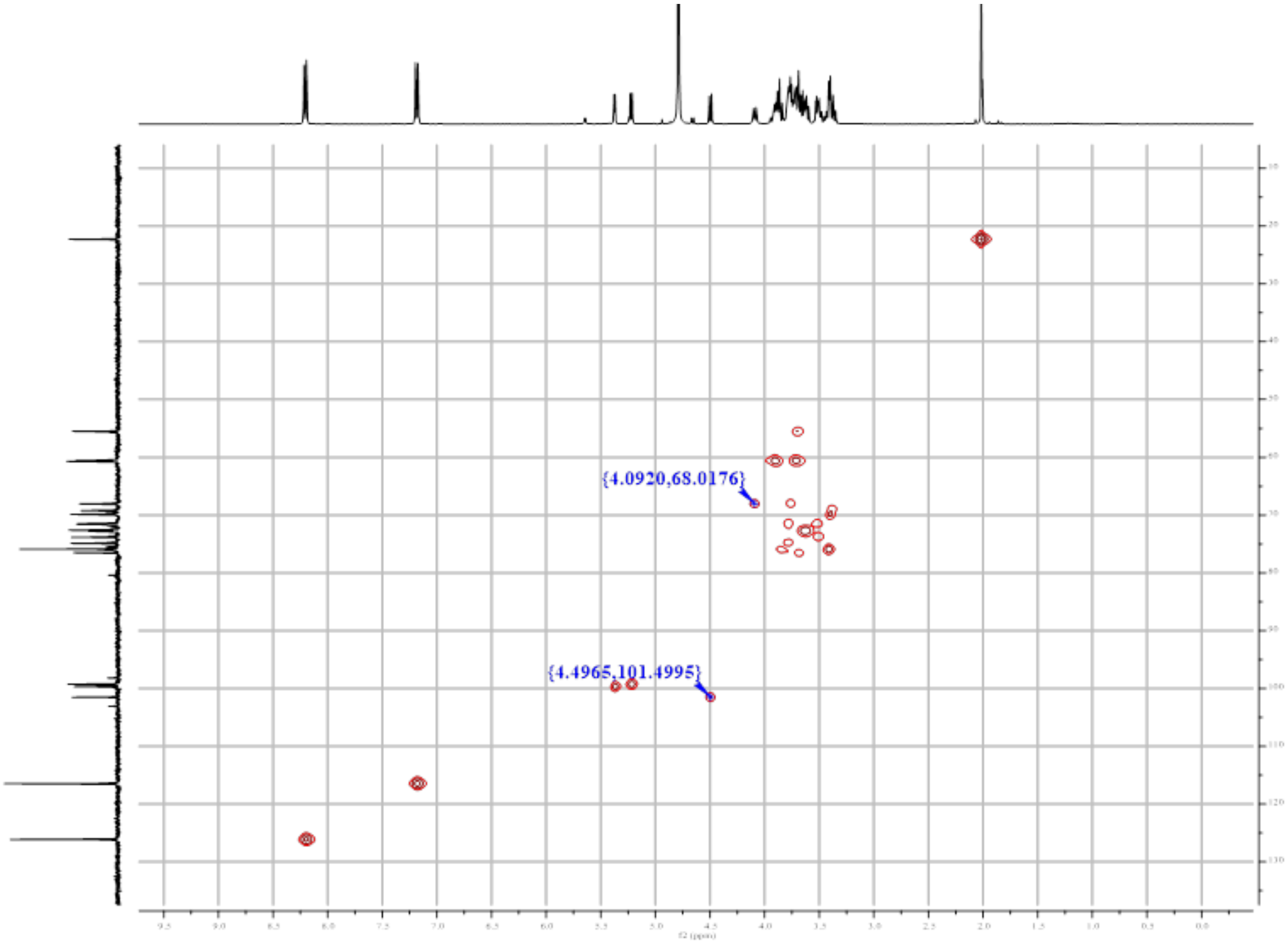

***p*-Nitrophenyl 2-acetamido-2-deoxy-β-D-glucopyranosyl-(1→6)-α-D-glucopyranosyl-(1→4)-β-D-glucopyranoside T2**, HMBC (600 MHz, D_2O)

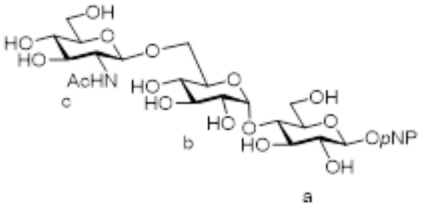

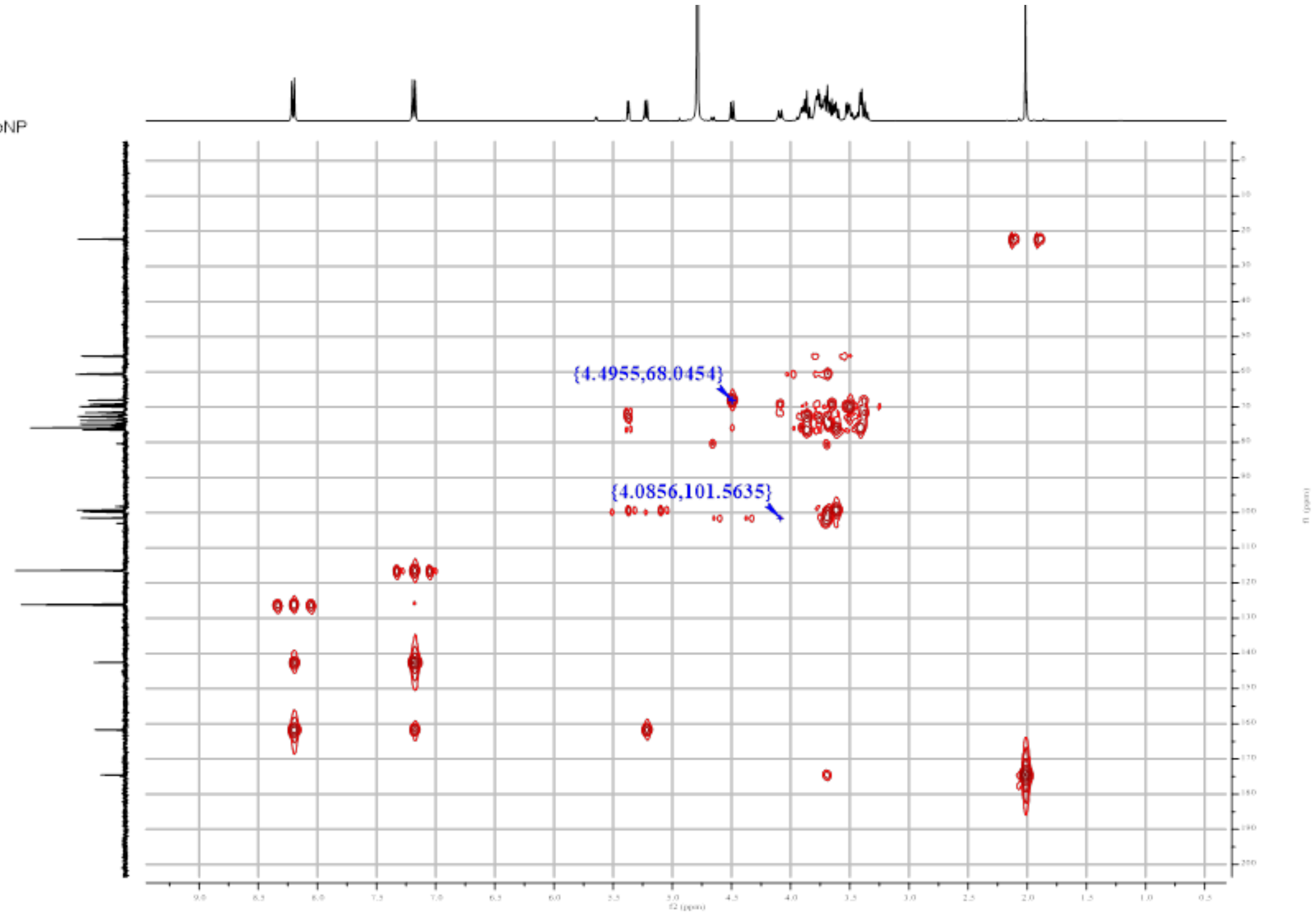

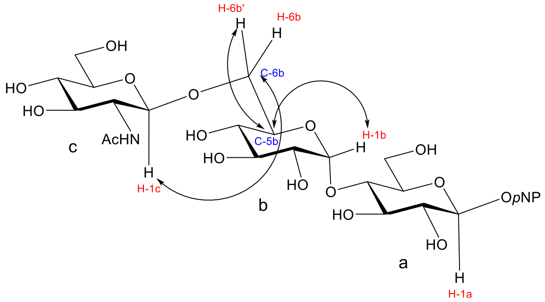

The correlation between C-5b and H-1b overlaps with other signals, making this evidence less supportive to the hypothesis that the signal assigned as H-6b' instead of H-6a'. However, none of the carbons that correlated with the signal (assigned as H-6b') had any correlation with H-1a, which means the newly formed glycosidic linage was not in the same sugar ring with the *p*NP-glucose residue, as further evidence of the linear structure.

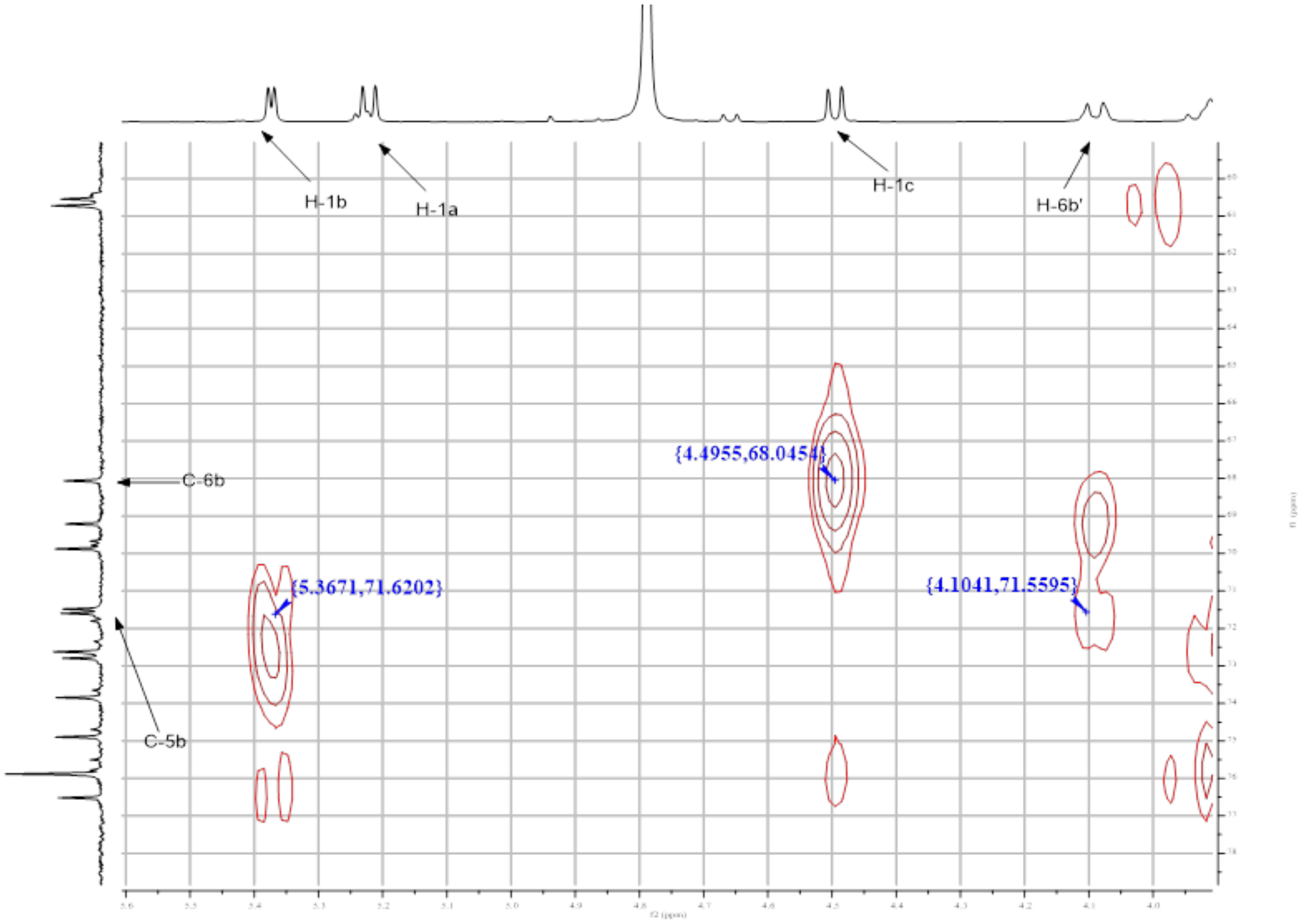

Supplement: SC-013-D2SC00222A-s001 [file SC-013-D2SC00222A-s001.pdf]
